# Comprehensive analysis of the role of ICOS ( CD278 ) in pan-cancer prognosis and immunotherapy

**DOI:** 10.1186/s12885-023-10564-4

**Published:** 2023-02-28

**Authors:** Xiashuang Zhao, Yongfeng Wang, Xianglai Jiang, Bangqian Mo, Chenyu Wang, Mingzheng Tang, Yao Rong, Guiqian Zhang, Ming Hu, Hui Cai

**Affiliations:** 1 The First Clinical Medical College of Gansu, University of Chinese Medicine (Gansu Provincial Hospital), 730000 Lanzhou, Gansu China; 2 General Surgery Clinical Medical Center, Gansu Provincial Hospital, 730000 Lanzhou, Gansu China; 3 Graduate School, Ning Xia Medical University, 750004 Yinchuan, Ningxia China; 4 Key Laboratory of Molecular Diagnostics and Precision Medicine for Surgical Oncology in Gansu Province, Gansu Provincial Hospital, 730000 Gansu, China; 5 NHC Key Laboratory of Diagnosis and Therapy of Gastrointestinal Tumor, Gansu Provincial Hospital, 730000 Lanzhou, China; 6 The First Clinical Medical College of Lanzhou University, 204 Donggang West Road, 730000 Lanzhou, Gansu China; 7 Gansu Provincial Hospital, 730000, Lanzhou, Gansu, China

**Keywords:** ICOS, Tumor immune, Immune checkpoint, Prognosis, Pan-cancer

## Abstract

**Background:**

The immunological checkpoint known as Inducible T Cell Costimulatory Factor (ICOS, Cluster of Differentiation, CD278) is activated and expressed on T cells. Both somatic cells and antigen-presenting cells expressed its ligand, ICOSL (including tumor cells in the tumor microenvironment).It is important for immunosuppression. Uncertainty surrounds the function of ICOS in tumor immunity.

**Methods:**

Several bioinformatics techniques were employed by us to thoroughly examine the expression and prognostic value of ICOS in 33 cancers based on data collected from TCGA and GTEx. In addition, ICOS was explored with pathological stage, tumor-infiltrating cells, immune checkpoint genes, mismatch repair (MMR) genes, DNA methyltransferases (DNMTs), microsatellite instability (MSI),and tumor mutation burden (TMB).In addition,To ascertain the level of ICOS expression in various cells, qRT-PCR was employed.

**Results:**

The findings revealed that ICOS expression was up regulation in most cancer types. The high expression of ICOS in tumor samples was related to the poor prognosis of UVM and LGG; The positive prognosis was boosted by the strong expression of ICOS in OV, SARC, SKCM, THYM, UCEC, and HNSC. The result is that the expression of malignancy was revealed by the immune cells’ invasion.profile of ICOS in different types of cancer. Different ways that ICOS expression is connected to immune cell infiltration account for variations in patient survival. Additionally, the TMB, MSI, MMR, and DNMT genes as well as ICOS expression are linked in many cancer types.The results of PCR showed that it is highly expressed in gastric, breast, liver and renal cell carcinoma cell lines compared with normal cells.

**Conclusion:**

This study suggests that ICOS may be a potential tumor immunotherapy target and prognostic marker.

**Supplementary Information:**

The online version contains supplementary material available at 10.1186/s12885-023-10564-4.

## Introduction

According to statistics, cancer is the first or second cause of death before the age of 70 in 183 countries, ranking third or fourth in 183 countries. In the world, the leading cause of death is cancer [1]. The first-line treatment for most cancer patients is still surgery, chemotherapy, and radiotherapy. Immune checkpoint blockade therapy for cancer, however, has become a crucial component of treatment since its breakthrough [2]. Immunotherapy is mainly for the body ' s immune status, by regulating immune function so as to achieve a method of treating diseases. Compared with other treatment schemes, it has better effects, such as eliminating tumors and improving immunity, preventing tumor recurrence, so as to achieve the purpose of curing diseases. In addition, the side effects of immunotherapy are small, which can prolong the survival time of patients and make great progress in the treatment of tumors.

Cancer development depends largely on immune dysfunction [3–5]. TME is composed of immune cells, stromal cells, and other cells. In TME, tumor cells and immune cells interact dynamically, which determines the characteristics and heterogeneity of cancer [6–8]. In the case of long-term exposure to antigen, T cells deteriorate and exhibit increased expression of IRS, ICOS, and the programmed cell death receptor (PD-1) [9, 10]. Immunotherapy, such as blocking of immune checkpoints (ICBs), has made great progress and shown great potential, especially for patients resistant to radiotherapy and chemotherapy [11–14]. However, clinical options for immunotherapy are lacking [15]. Therefore, it is crucial to investigate and confirm more efficient immune-related targets.

Inducible T cell costimulatory factor (ICOS, cluster of differentiation, CD278) is an activated costimulatory immune checkpoint expressed on T cells. Its ligand ICOSL was expressed in antigen-presenting cells and somatic cells (including tumor cells in the tumor microenvironment) [16, 17]. As one of the most common targeted immune checkpoints, ICOS is considered a possible way to develop new immunotherapy [18]. Some clinical studies shown that blocking ICOS alone can inhibit tumor growth and proliferation, as well as TIGIT, CTLA4, and PD-1 [15, 19]. Numerous malignancies, including colon adenocarcinoma (COAD), endocervical adenocarcinoma (CESC), and lung adenocarcinoma(LUAD), have been linked to the upregulation of ICOS. There are several ways that ICOS inhibits T cells in the tumor microenvironment (TME). ICOS is therefore considered a promising prognostic biomarker and a target for developing new immunotherapy [20, 21]. However, the function of ICOS(CD278) in pan-cancer remains unknown.

In this study, the expression profiles of ICOS, prognostic, immune-related level, tumor mutation burden (TMB), microsatellite instability (MSI), mismatch repair (MMR) genes, and DNA methyltransferases(DNMTS) were thoroughly analyzed at the pan-cancer level using data sets from the TCGA, GEO, and GTEx [22].

## Method

### Data source and processing

The ICOS expression data, clinicopathological, and survival data of 33 cancer genome maps were obtained by the TCGA database(https://portal.gdc.cancer.gov/). To compare the expression level of ICOS in healthy tissues, RNA sequences were taken from the GTEx database. (http://commonfund.nih.gov/GTEx/) database to compare the expression level of ICOS in normal tissues.

### Based on the level of ICOS expression, analyses of survival, ROC curves, and clinicopathological association were performed

After gathering the information on 33 tumor types from the TCGA and 33 tumor types from the normal GTEx samples, the correlation between ICOS expression and clinical prognosis was further examined.Total survival (OS), disease-specific survival (DSS), disease-free interval (DFI), and progression-free interval are the key markers for our accurate assessment (PFI).The patients were split into two groups: those at high and low risk, based on the amount of ICOS expression. The prognosis of the two groups was compared using Kaplan-Meier (KM) analysis.Additionally, we conducted a COX analysis to look at the connection between ICOS expression and the prognosis for all types of cancer. As a cutoff, a COX P- value of 0.05 was chosen. Additionally, based on the TCGA’s gene expression study,we evaluated the correlation between ICOS expression and stage of cancer.

Adopt R-package “survival ” and “ forest map ” were mainly used to design forest map, and R-package “limma ” and “ ggpubr ” were mainly used for clinicopathological correlation analysis. R package “ pROC ” was used to visualize the RNAseq data of TCGA and GTEx and calculate the area under the ROC curve ( AUC ) to determine their diagnosis and prognosis.

### Immunity and ICOS expression are correlated

We investigated the prevalence of immune cells harboring tumors in 33 malignancies. The expression level of ICOS was correlated with the abundance of ICOS in CD4 + T cells, CD8 + T cells, and macrophages. The correlation between ICOS expression and T cell subtype characteristics was analyzed.

We also used the estimation algorithm in R-package “estimation” and “limma” to estimate the matrix score and immune score of stromal cells and immune cells, and further analyzed the correlation between ICOS expression and these two scores (P < 0.001 as a cut-off value).

### Association between ICOS(CD278) expression on TMB, MSI, MMR, and DNMT in pan-cancer

We determined the TMB score and MSI score by removing the patients’ mutation spectrum from TCGA, and we examined the connection with ICOS expression. Radar maps were designed using the R-package “fmsb” to visualize both indicators. We also analyzed Spearman’s correlation analysis between ICOS expression and the MMR gene, DNMTs, respecively. And create these graphics with the R-packages “reshape2” and “RColorBrewer” to see the outcomes.

### Gene set enrichment analysis

The Kyoto Encyclopedia of Genes and Genomes (KEGG) gene set (https://www.kegg.jp/kegg/) [23–25]and the Gene Ontology (GO) gene set were acquired from the Gene Enrichment Analysis (GSEA) weibsite (http://www.gsea-msigdb.org/gsea/index.jsp). Functional annotations and enrichment pathways of ICOS were analyzed by GO and KEGG.These data are combined and analyzed using the R packages org.Hs.eg.db, clusterProfiler, and enrichplot.

### Drug sensitivity of ICOS in Pan-Cancer

Download NCI-60 compound activity data and RNA-seq expression files with CallMinerTM to analyze and visualize ICOS chemosensitivity in pan-cance (https://discover.nci.nih.gov/cellminer/home.do). We mainly selected some FAD or clinically approved drugs for analysis.

### Cell culture

Human normal gastric epithelial cell GES-1, human gastric cancer cell lines AGS, MKN-45 and HGC-27 (from our research group, preserved in Gansu Provincial People ‘s Central Laboratory) ; human normal liver cell L02 and human liver cancer cell SMMC-7721 (from our research group, preserved in Gansu Provincial People ‘s Central Laboratory) ; human normal mammary epithelial cells MCF-10A, human breast cancer cells MCF-7 and MDA-MB-231 (General Hospital of Ningxia Medical University Laboratory Gift) ; human renal proximal tubular epithelial cells HK-2, human renal clear cell carcinoma CAKI-2 (General Hospital of Ningxia Medical University Laboratory Gift). The cells were cultured in RPMI-1640 medium containing 10% fetal bovine serum ( FBS ) and 1% double antibody ( penicillin and streptomycin ) at 37°C.

### RNA isolation and qRT-PCR

Total RNA was extracted according to the instructions of the M5 Universal RNA Mini Kit kit. The absorbance values at 260 nm and 280 nm were measured by spectrophotometer to ensure that the RNA concentration and purity were consistent. RNA was reverse transcribed into cDNA according to the instructions of M5 Sprint qPCR RT kit with gDNA remover reverse transcription kit. We used cDNA as a template and 2 × M5 HiPer SYBR Premix EsTaq(with Tli RNaseH) qRT-PCR detection. ICOS primers were designed and synthesized by Bioengineering (Shanghai) Co. The primer sequences are as follows : forward, 5 ' -GGGCACAATTCCCTCTC-3 ' ; reverse, 5 ' -TTGCATCGACATTGGC.

### Statistical analysis

The Wilcox test was used to examine the gene expression data from TCGA and GTEx. The connection between ICOS expression and immune cell score was assessed using Spearman correlation analysis.To illustrate the findings, the R package is used for all calculations. Statistics were judged significant at P < 0.05.

## Results

### Pan-cancer expression landscape of ICOS

**Fig. 1:**
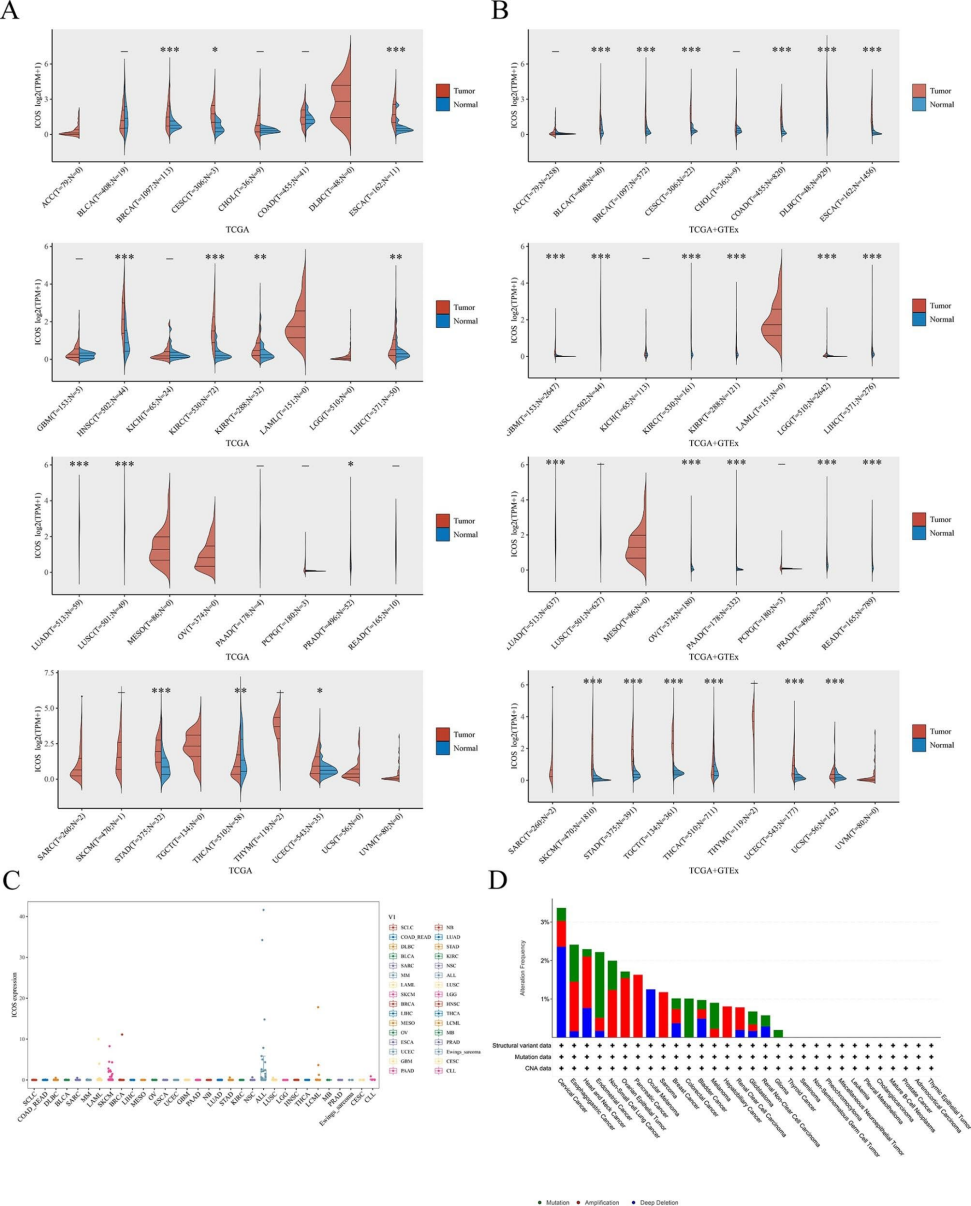
**The expression levels of ICOS in different types of human cancers. (A)**. Based on the TCGA database, the expression levels of ICOS in 33 tumor tissues and normal tissues were compared. **(B)**. The expression level of ICOS in normal tissues and tumor tissues is based on TCGA and GTEx data sets. **(C)** From the TCGA data, the expression levels of ICOS genes in different types of cancer. **(D)** The expression distribution of ICOS in different tumor tissues. *P<0.05, **P<0.01, ***P<0.001

Based on the TCGA and GTEx datasets, the expression of ICOS in normal and malignant samples is compared. According to the TCGA dataset, the expressions of ICOS in the following tissues were up-regulated in the two comparisons: BRCA, CESC, ESCA, HNSC, KIRC, KIRP, LIHC, LUAD, LOSC, PRAD, STAD, and UCEC. In THCA, the expression of ICOS was less tightly controlled (Fig. 1A). On the other hand, according to the TCGA and GTEx datasets, ICOS expression in THCA tissues was noticeably higher than in healthy tissues. In addition to THCA, the expression of ICOS in BLCA, COAD, DLBC, GBM, LGG, OV, PAAD, READ, SKCM, TGCT, and UCS patients in the comprehensive database was also significantly increased (Fig. 1B). By comparison, the expression levels of ICOS in BRCA, CESC, ESCA, HNSC, KIRC, KIRP, LIHC, LUAD, LOSC, PRAD, STAD, and UCEC tumor samples were significantly higher than those in normal tissues, as shown in the figure. According to the integrated database, compared with normal tissues, the expression levels of ICOS in BLCA, COAD, DLBC, GBM, LGG, OV, PAAD, READ, SKCM, TGCT, and UCS were also significantly increased (Fig. 1A, B).

We looked more closely at ICOS expression changes as reported by the cBioPortal database. According to the findings, depth loss was related with the highest mutation rate, which was then followed by mutation and amplification. Cervical cancer changes the most frequently of all cancers (Fig. 1C). Based on CCLE data, Fig. 1D shows the relative levels of ICOS expression in several cell lines.The tumor and adjacent normal tissues for ICOS across all TCGA tumors were shown in supplementary file.1.

### Correlation between the expression level of ICOS and the overall survival of tumor patients

**Fig. 2:**
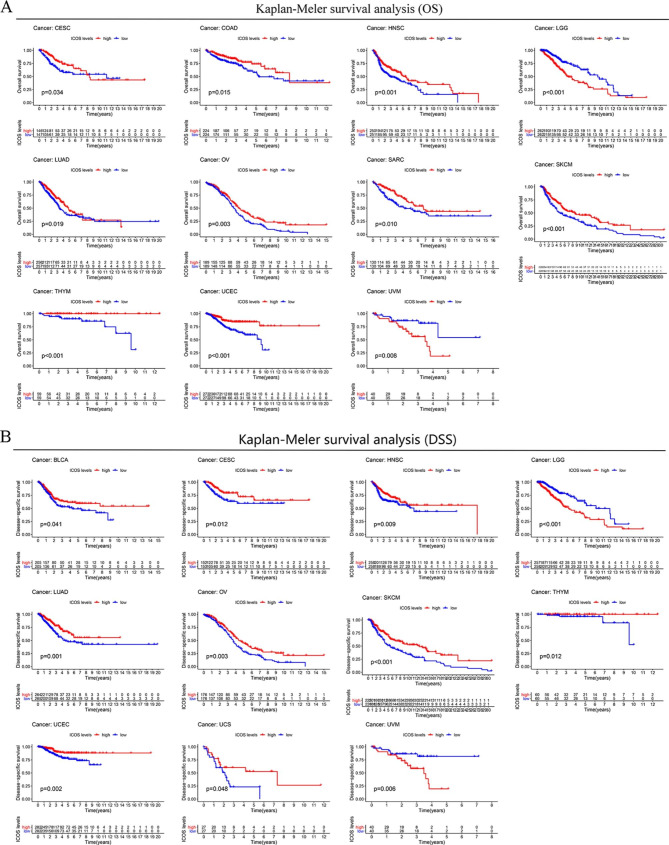
**Survival curve analysis of ICOS gene expression in different tumor types. (A)**: OS of CESC, COAD, HNSC, LGG, LUAD, OV, SARC, SKCM, THYM, UCEC, UVM. **(B)**: DSS of BLCA, CESC, HNSC, LGG, LUAD, OV, SKCM, THYM, UCEC, UCS, UVM. Bladder Urothelial Carcinoma (BLCA), Cervical squamous cell carcinoma and endocervical adenocarcinoma (CESC), Colon adenocarcinoma (COAD), Head and Neck squamous cell carcinoma (HNSC), Brain Lower Grade Glioma (LGG), Lung adenocarcinoma (LUAD), Ovarian serous cystadenocarcinoma (OV), Sarcoma (SARC), Skin Cutaneous Melanoma (SKCM), Thymoma (THYM), Uterine Corpus Endometrial Carcinoma (UCEC), Uterine Carcinosarcoma (UCS), Uveal Melanoma (UVM).

We used different databases to explore the prognostic value of ICOS for pan-cancer. It is worth noting that the Kaplan-Meier cumulative curve shows that in the TCGA database, the expression of ICOS(CD278) is related to the prognosis of several cancers. In eleven distinct cancer types, including LUAD, OV, SARC, SKCM, THYM, UCEC, CESC, COAD, HNSC, BLCA, UCS, and ACC, ICOS played a protective function. In this situation, patients with higher ICOS expression outlived those with lower ICOS expression by a greater margin (Fig. 2, Supplementary file.2). In contrast, ICOS had a negative effect on patients with LGG, UVM, and GBM cancers, where higher ICOS expression was associated with a lower survival rate than low ICOS mRNA levels (Fig. 2, Supplementary file.2).

**Fig. 3:**
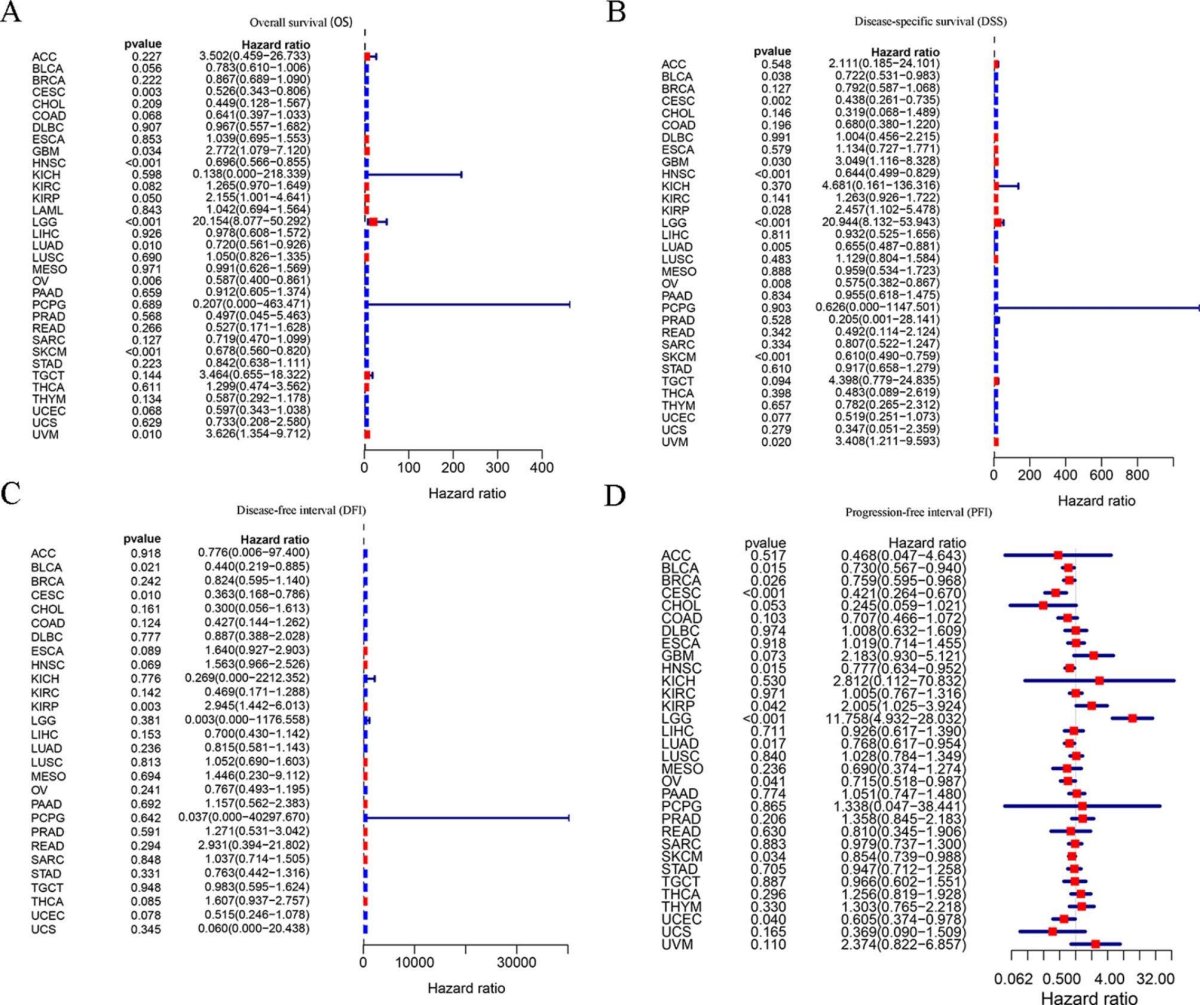
**Correlation analysis of ICOS expression with survival using the COX method for different types of cancers in TCGA. (A)** OS. **(B)** DSS. **(C)** DFI. **(D)** PFI.

Through COX analysis, we looked more closely at ICOS-related survival (OS, DSS, DFI, and PFI) (Fig. 3). Because of this, we discovered that ICOS is a protective prognosis factor in CESC, HNSC, LUAD, OV, SKCM, and UCEC; however in UVM, LGG and KIRP had a negative prognostic role.

Since the two methods are different, the results are different. For example, the results of two analyses showed that SARC, THYM, COAD, BLAC, UCS, and ACC were significant in COX analysis, but not in K-M analysis.

### Correlation analysis of ICOS ( CD278 ) expression with age and cancer stage

**Fig. 4:**
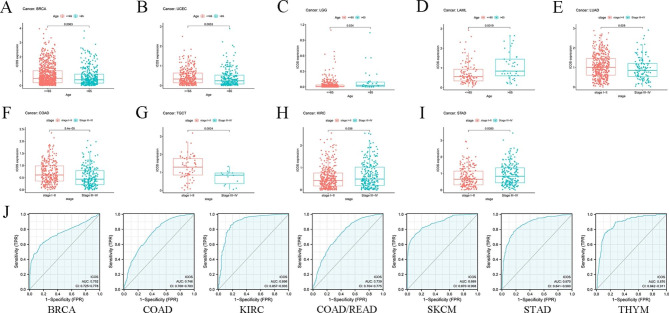
**Correlation analysis of ICOS ( CD278 ) expression with age and cancer stage** ICOS gene expression associated with age in BRAC **(A)**, UCEC **(B)**, LGG **(C)**, and LAML **(D)**. ICOS gene expression is related to the stage in LUAD **(E)**, TGCT **(F)**, COAD **(G)**, KIRC **(H)**, STAD **(I).** ROC analysis of ICOS genes in TCGA database**(J)**.Breast invasive carcinoma (BRCA), Uterine Corpus Endometrial Carcinoma (UCEC), Brain Lower Grade Glioma (LGG), Acute Myeloid Leukemia (LAML), Lung adenocarcinoma (LUAD), Testicular Germ Cell Tumors (TGCT), Colon adenocarcinoma (COAD), Kidney renal clear cell carcinoma (KIRC), Stomach adenocarcinoma (STAD), Head and Neck squamous cell carcinoma (HNSC), Lung adenocarcinoma (LUAD), and Testicular Germ Cell Tumors(TGCT).

Several tumors’ age and stage are related to the expression of ICOS. Particularly, patients who are 65 years of age or younger have significant levels of ICOS expression. However, in LGG and LAML (Fig. 4A-D), Individuals over 65 had higher levels of ICOS expression than patients who were younger or older. In contrast, ICOS was low expressed in individuals over 65, especially in BRCA and UCEC. ICOS was also significantly expressed in stage I-II patients and lowly expressed in stage III-IV patients with LUAD, TGCT, and COAD (Fig. 4E-G); similarly, ICOS of KIRC and STAD (Fig. 4H, I) were highly expressed in stage III-IV patients and lowly expressed in stage I-II patients. Moerover, the receiver operating characteristic (ROC) curve was emplored to explore the diagnostic value of ICOS in different cancers. As shown in Fig. 4J, the ICOS had a moderate diagnostic accuracy of BRCA, COAD, COAD/READ, KIRC, SKCM, STAD, and THYM. (AUCs were above 0.7 and even 0.8). Taken together, these analyses suggest that ICOS is a meaningful biomarker in a variety of cancers.

### Study on the correlation between the expression of ICOS and immune infiltration

**Fig. 5:**
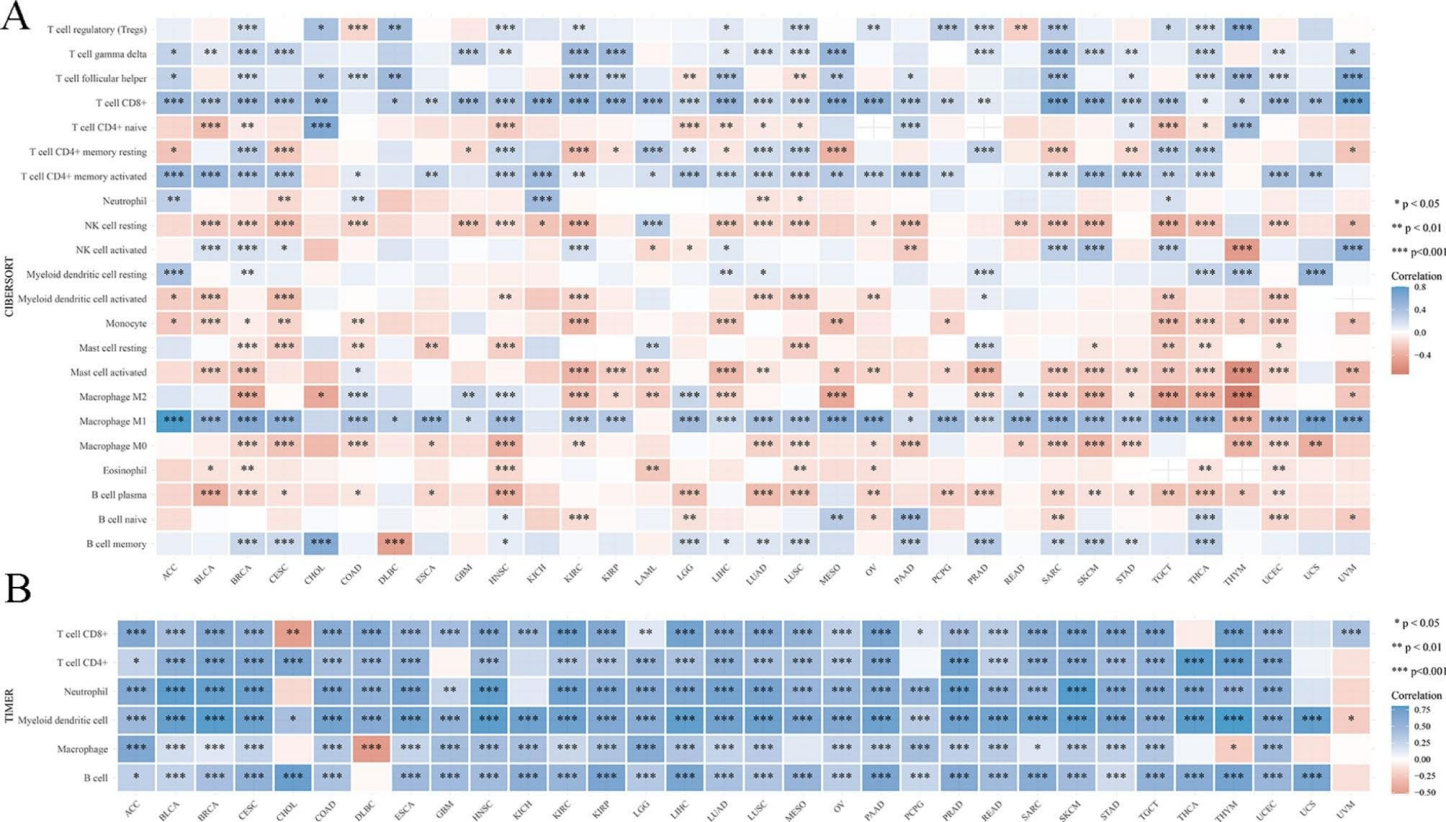
The relationship between the expression of the ICOS gene and the level of immune cell infiltration in 33 types of cancer was analyzed by CIBEROR**(A)** and TIMER**(B)** databases. * *P*<0.05, **P<0.01, ***P<0.001

In addition, we used CIBEROR and TIMER methods to analyze the correlation of immune infiltrating cells in 33 cancers.Results from the CIBEROR and TIMER databases revealed a correlation between different immune cells and the expression of ICOS in 33 malignancies (Fig. 5A,B).

Using CIBEROR techniques, we investigated the potential relationship between ICOS expression and 22 immune cell infiltration cells in various cancer types (Detailed information is provided in supplementary file.3). The aforementioned findings demonstrated that immune cell infiltration in tumors revealed the expression profile of ICOS in various cancer types. To explain the potential variations in patient survival, this gene expression is primarily linked to the infiltration of immune cells in various ways.

**Fig. 6:**
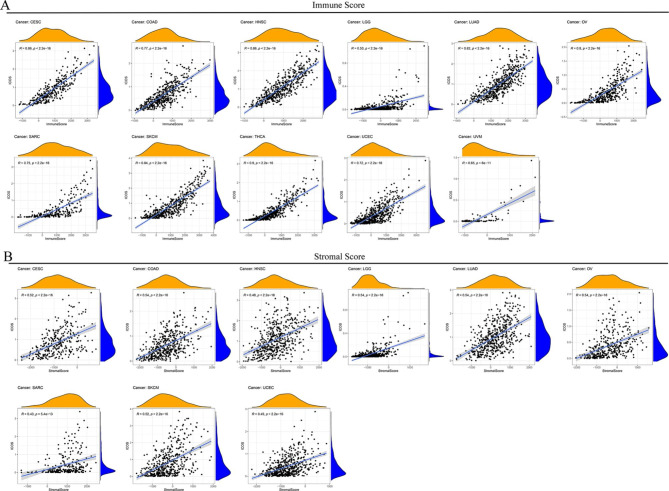
Analysis of ICOS gene expression and interstitial score **(A)** and immune score **(B)** of cancer

In order to determine the relationship between the expression of ICOS and the immune and matrix scores in various cancer types, we estimated the immune score and matrix score. In the following cells: CESC, COAD, HNSC, LGG, LUAD, OV, SARC, SKCM, THCA, UCEC, and UVM, the expression of ICOS was substantially linked with stromal and immunological scores (P<0.001). These findings showed that ICOS expression increased along with an increase in the number of stromal cells and immune cells (Fig. 6A, B).

**Fig. 7:**
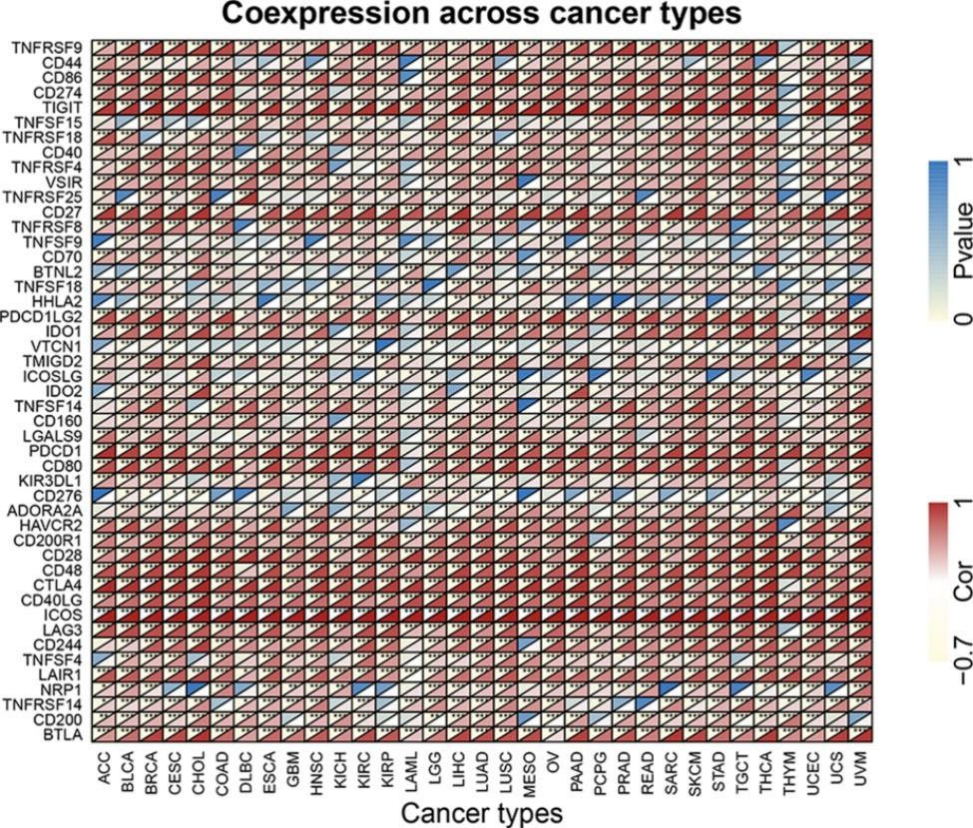
The correlation between ICOS expression and immune checkpoint gene expression in 33 cancer types. *P<0.05,**P<0.01, and***P<0.001

To further study the potential mechanism of immunosuppression of the ICOS signaling pathway, in 33 different cancer types, we looked at the relationship between ICOS expression and immunological checkpoint markers.(Fig. 7). In general, the results show that in different T cells, the expression of ICOS is significantly correlated with many immune checkpoints. For instance, ICOS has positive relationships with PDCD1, TIGIT, CD274, and CTLA4 in the majority of cancer types, indicating a broad co-expression landscape.

### Correlation analysis on TMB, MSI, MMR, and DNMT

**Fig. 8:**
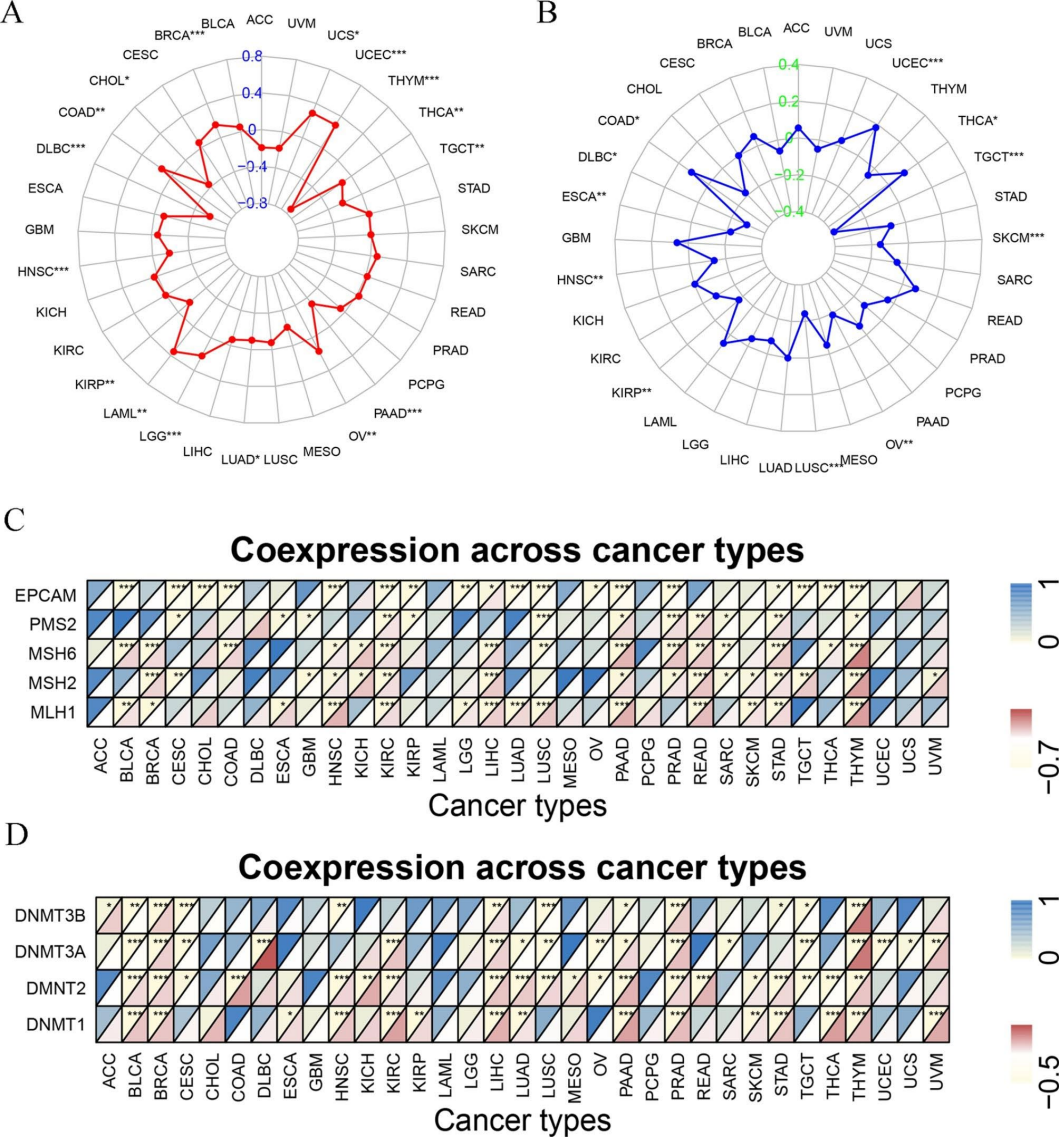
**Relationships between ICOS gene expression and TMB, MSI in Pan-cancer, and the correlation between ICOS expression and five mismatch repair genes.** (**A**) Radar map showed the expression relationship between TMB and ICOS in different cancers. Red curve represents correlation coefficient and blue value represents range. (**B**) Radar map showed the expression relationship between MSI and ICOS in different cancers. Blue curve represents correlation coefficient and green value represents range. **(C)**, and the correlation between ICOS expression and DNA methyltransferase **(D)** across 33 cancer types. * *P*<0.05,** *P*<0.01,and *** *P*<0.001

In addition, we evaluated the relationship between TMB, MSI, and ICOS expression. We found that ICOS expression was positively correlated with TMB in BRCA, COAD, LAML, LGG, OV, UCEC, and UCS, and negatively correlated with TMB in CHOL, DLBC, HNSC, KIRP, LUAD, PAAD, TGCT, and THCA (Fig. 8A). In COAD, THCA, and VCEC, ICOS expression correlated favorably with MSI and negatively with DLBC, ESCA, HNSC, KIRP, LUSC, OV, SKCM, and TGCT (Fig. 8B).

In addition, we analyzed the relationship between ICOS and the expression of MMR genes and MSH2 upstream gene EPCAM. In 17 of the 33 different malignancies, there was a positive correlation between ICOS and the expression of at least one MMR gene. ICOS had a bad correlation with the expression of the MMR gene in the 7 tumors (Fig. 8C). We also looked at the relationship between ICOS and DNMT expression. ICOS was positively correlated with at least one DNMTs expression in 19 cancers, and negatively correlated with DNMT expression in 5 cancers (Fig. 8D).

### Functional analysis by GSEA

**Fig. 9:**
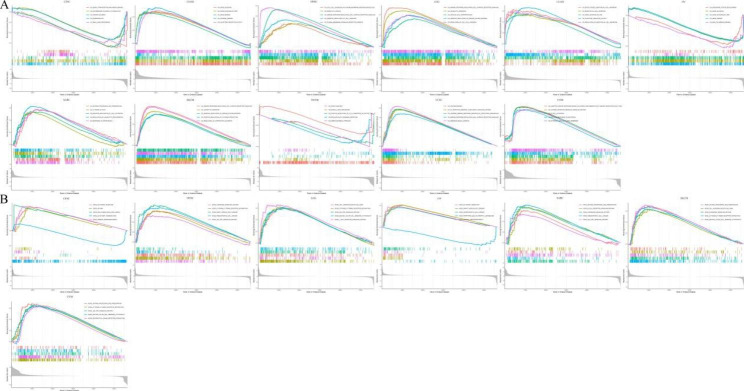
**Functional enrichment analysis of GO and KEGG on ICOS by GSEA. (A)** GO functional annotation of ICOS gene in CESC, COAD, HNSC, LGG, LUAD, OV, SARC, SKCM, THYM, UCEC, and UVM. **(B)** KEGG pathway analysis of ICOS gene in CESC, HNSC, LGG, OV, SARC, SKCM, and UVM. Different color curves showed that ICOS gene regulated different functions or pathways of different cancers. The upward peak of the curve indicated positive regulation, and the downward peak of the curve indicated negative regulation

We then carried out GO functional annotation and KEGG pathway analysis of ICOS in various cancer types by GSEA. According to GO functional analysis, in the biological processes of gene silencing, RNA silencing, and mRNA binding in OV, CESC, and THYM, ICOS acts in a negative manner. ICOS is positively regulated in COAD, HNSC, LGG, LUAD, SARC, SKCM, UVM, and UCEC, and provides multiple immune-related functions. Its functions include cell immune response regulation, endocytosis, negative regulation of cell adhesion, plasma membrane signal receptor complex, cell activation, and immune response(Fig. 9A). KEGG pathway analysis results show that ICOS can regulate: the T cell receptor signaling pathway and chemokine signaling pathway(Fig. 9B).

### Drug sensitivity analysis of ICOS

**Fig. 10:**
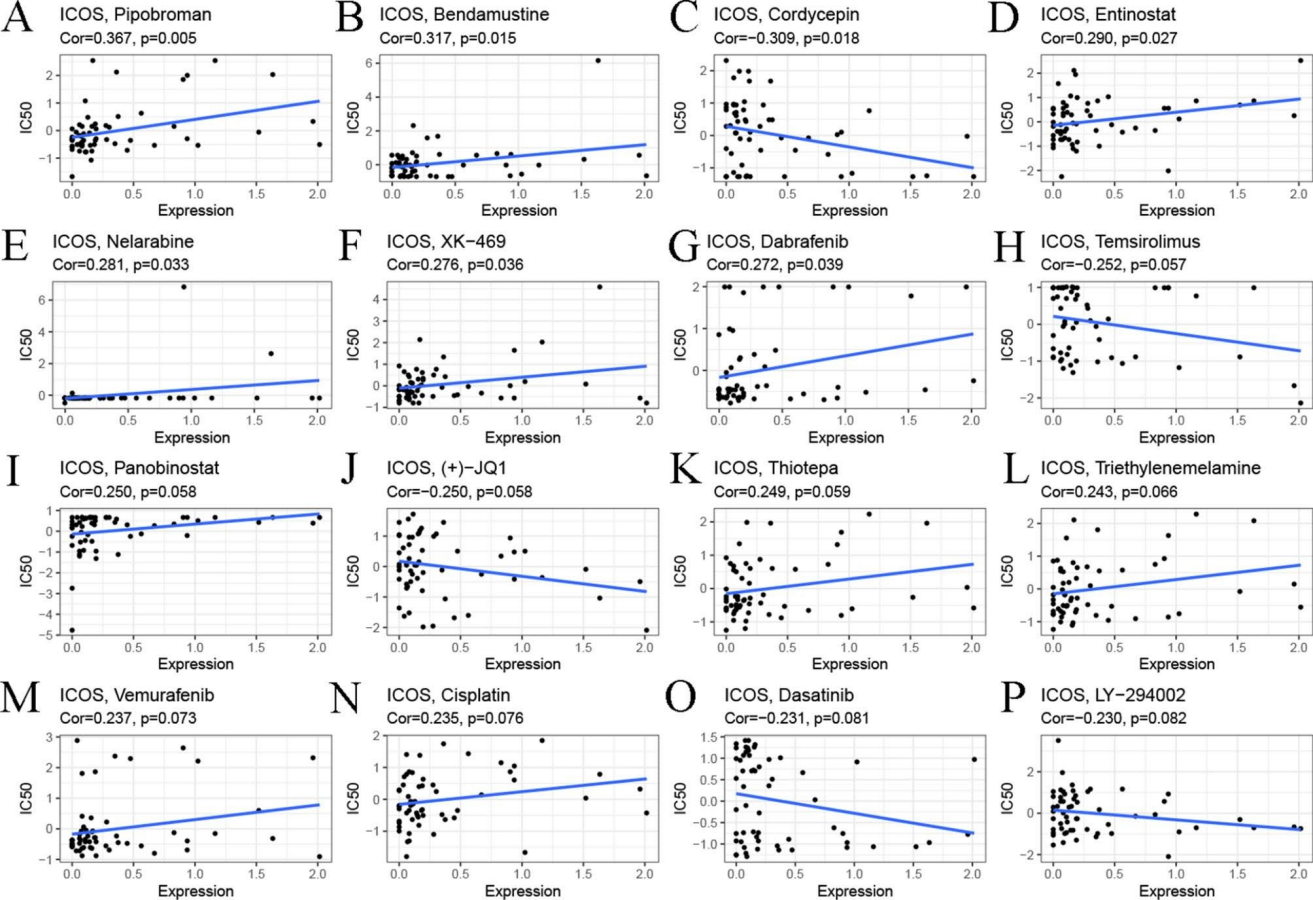
**Drug sensitivity analysis of ICOS.** The expression of ICOS was associated with the sensitivity of pipobroman **(A)**, bendamustine **(B)**, cordycepin **(C)**, entinostat **(D)**, nelarabine **(E)**, XK-469 **(F)**, dabrafenib **(G)**, temsirolimus** (H)**, panobinostat **(I)**, (+)-JQ-1 **(J)**, thiotepa **(K)**, triethylenemelamine **(L)**, vemurafenib **(M)**, cisplatin **(N)**, dasatinib **(O)**, and LY-294,002 **(P)**

We further investigated the potential correlation analysis between drug sensitivity and ICOS expression using the CellMiner™ database. Notably, ICOS expression was negatively correlated with drug sensitivity of cordycepin, temsirolimus, (+)-JQ1, dasatinib, and LY-294,002 (Fig. 10C,H,J,O,P). Our results exhibited that ICOS expression was positively associated with pipobroman, bendamustine, entinostat, nelarabine, XK-469, dabrafenib, panobinostat, thiotepa, triethylenemelamine, vemurafenib, and cisplatin sensitivity (Fig. 10A,B,D-G,I,K-N). In summary, we found that ICOS may be associated with chemoresistance to certain drugs. It is speculated that it may be related to the involvement of ICOS in RNA silencing, mRNA binding endocytosis and cellular immune response regulation.

### Expression of ICOS in different cancers

**Fig. 11:**
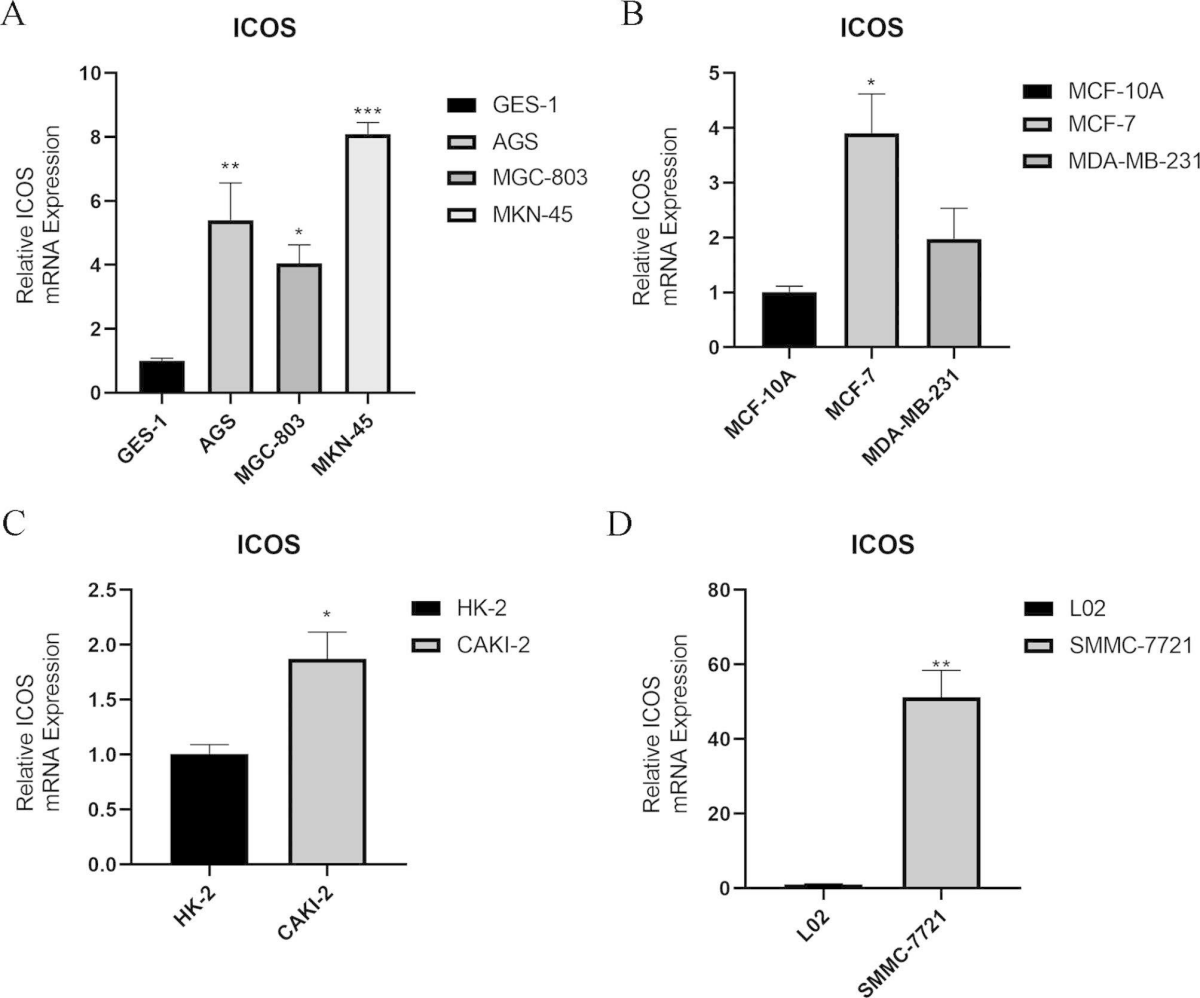
**The expression of ICOS in different cancer cell lines. ( A )** The expression of ICOS in gastric cell lines GES-1, AGS, MKN-45, MGC-803 ; **( B )** The expression of ICOS in breast cell lines MCF-10 A, MCF-7 and MDA-MB-231 ; **( C )** The expression of ICOS in renal cell lines HK-2 and CAKI-2; **( D )** Expression of ICOS in liver cell lines L02 and SMMC-7721. * *P*<0.05,** *P*<0.01,and *** *P*<0.001

The results showed that ICOS was highly expressed in gastric cancer cells AGS, MKN-45 and MGC-803 compared to normal cells ( Fig. 11A ) ; it was highly expressed in breast cancer cells MCF-7 and MDA-MB-231 compared to normal cells ( Fig. 11B ). CAKI-2 was highly expressed in renal carcinoma cells compared with normal cells ( Fig. 11C ). Hepatocellular carcinoma SMMC-7721 cells were highly expressed compared with normal cells ( Fig. 11D ).Genes are selectively expressed in different time and space, so that cells have different physiological functions and morphological structures. The occurrence of tumor is under the influence of various factors, resulting in local tissue cells in the relevant gene mutations, aberrant DNA methylation and immune escape a series of changes. Therefore, the same gene in different cancer cells express different, different functions.

## Discussion

The recent success of checkpoint inhibitors in cancer treatment provides new therapeutic prospects for cancer treatment. Inducible costimulator (ICOS) is a costimulatory receptor for T cell enhancement. The ICOS / ICOSL axis has dual effects, and it may be involved in anti-tumor T cell response and tumor promotion response due to its association with regulatory T cells (Tregs) inhibitory activity. Therefore, both antagonists and agonist antibodies may be targeted for cancer therapy. In the study, it was demonstrated that an ICOS agonist monoclonal antibody improved inhibitory checkpoint blocking. On the other hand, immunosuppressive Tregs can also be inhibited by an anti-ICOS monoclonal antibody, in addition to lymphatic tumor cells that express ICOS [26]. Therefore, a key problem in tumor immunology and immunotherapy is increasing, extending, and forecasting the clinical success of the treatment.

In this study, a pan-cancer analysis workflow was carried out, and the role of ICOS in cancer was studied in depth. The results show that the prognostic impact of ICOS on different types of cancer. The expression of ICOS mediates infiltration of immune cells and is positive correlated with the expression of PDCD1, CTLA4, and TIGIT in most cancer types [27]. In a variety of cancers, the expression of ICOS is also related to TMB, MSI, DNMTS, and MMR genes.

This study shows that ICOS has great prognostic value in different types of cancer. According to LGG [28, 29] and UVM [30]reports, ICOS expression is upregulated and is associated with a bad prognosis, which is consistent with the findings of our study. The findings of our study also show a relationship between ICOS and the pathological phases of LGG and the predictive significance of UVM. The findings demonstrate that ICOS has the potential to be employed as a prognostic biomarker in a variety of cancer types [31–33]. According to the study, the expression of ICOS was up-regulated with the activation of T cells and NK cells [34, 35]. Additionally, during prolonged antigen stimulation, T cells deplete or become dysfunctional, and several IRS expressions—including PD1, CTLA-4, TIGIT, and ICOS are also increased [7, 12, 27, 36, 37], which are consistent with the results of our study, that is, the expression of ICOS is positively correlated with the effector T cells and Treg. Tumor tissues with up-regulated ICOS expression also showed abnormal immune characteristics [38–41]. Our GSEA analysis revealed that ICOS also demonstrated the capacity to negatively regulate immune-related processes and pathways, including cytokine-cytokine receptor interaction, chemokine signaling pathway, cytotoxicity mediated by natural killer cells, interferon-gamma reaction, and JAK-STAT3 signaling pathway.

Our findings demonstrated that among cancer types with poor prognoses, ICOS was positively linked with TIICs. Studies have reported that high TIIC status may lead to a poor prognosis. It may explain that some infiltrating immune cells, such as macrophages, can promote the development, and metastasis, especially in an immune microenvironment [42, 43], which can confirm our research results, that is, the overexpression of ICOS is related to the poor prognosis of some cancers. Further studies are needed, for example, different correlation analysis results may be due to the heterogeneity between tumors [44], and different types show different TME, tumor immunogenicity, TMB, and microenvironment [45, 46]. In summary, the immunosuppressive effect of ICOS is likely to lead to the survival and escape of tumor cells, affecting the occurrence and development of cancer and the prognosis of patients.

We examined the connections between ICOS and TMB, MSI, MMR genes, and DNMTs in order to further investigate the likely mechanism underlying the association between ICOS and tumor. MSI is a typical MMR gene mutation phenomenon [47, 48]. According to recent data, the majority of tumors with MSI-H/dMMR status have elevated TMB [49]. These features are associated with increased new antigens that affect tumor-infiltrating lymphocytes and responses to ICB, thus independently predicting responses to immunotherapy [50]. Our results showed that ICOS in COAD was positively correlated with MSI / TMB. At the pan-cancer level, the expression of ICOS in other types of cancer was more correlated with MSI / TMB. However, the expression of ICOS in THCA and OA is not consistent with TMB and MSI in some same types of cancer, which can be explained for two reasons. First of all, reports on the integration of MSI and TMB to predict ICB responses have been made, despite the fact that some studies have indicated that TMB is elevated in MSI tumors. This is because the link between MSI and TMB is still nonstationary. There is a lack of research on the relationship between ICOS and TMB in tumors, which needs more study. Second, distinct relationships between ICOS and TMB, and MSI in the same kind of cancer may result from the usage of datasets and the characteristics of the collecting process. In addition to gene mutation, epigenetic changes also profoundly affect tumor growth, proliferation, metastasis, and immunosuppression. One of the epigenetic control mechanisms is DNA methylation. Immune evasion and abnormal DNA methylation are linked to the development of tumors [51]. In diverse types of cancer patients, our investigation discovered a connection between DNMTs and ICOS expression, and DNA methylation may also be involved in the regulation of ICOS. The mechanism is related to the decreased expression of tumor suppressor and anti-tumor immune-related genes caused by DNA hypermethylation and the overexpression of tumor suppressor and immune-related genes caused by DNA hypomethylation. In conclusion, different methylation models affect various cancer types and their immune microenvironment mechanisms. This is a complex issue that will require additional investigation in the future.The correlation between DNMTs and ICOS also suggest that it is possible to target these checkpoints by methylation regulation or to improve the response rate by combining methylation regulators with ICB. We hypothesized that malignancies, activated T cells, and NK cells by immune cells were caused by aberrant genetic and epigenetic alterations [33]. Additionally, some T cells and NK cells up-regulate the expression of ICOS and suppress immune activity, creating an immunological milieu in tumors that encourages the growth and spread of malignancies and has a bad prognosis [34, 35].

In this study, we demonstrated the analysis of pancarcinoma with abnormal expression of ICOS in different tumors. Our findings will enable us to further study ICOS functionality. For certain cancers, the clinical use of ICOS blocking offers new therapeutic alternatives for cancer patients. Our research has several limitations. Firstly, more data from other public datasets are needed to support and validate our results. Second, although we found that ICOS expression is associated with tumor immune cell infiltration and patient survival, it may affect the survival of patients through immune infiltration cells.

## Conclusion

According to this study, ICOS may be used as a cancer biomarker for prognosis and a possible therapeutic target. utilized to enhance prognosis, find possible targets for cancer treatments, and improve cancer detection. Therefore, a tailored prognosis and more therapy options are offered to patients by combining routine clinical tests with an assessment of ICOS expression.

## Electronic supplementary material

Below is the link to the electronic supplementary material.

Supplementary Material 1: The expression of ICOS in pan cancer was analyzed using paired tumor/normal samples from TCGA and GTEx databases.

Supplementary Material 2: Survival curve analysis of ICOS gene expression in different tumor types.

Supplementary Material 3: The relationship between ICOS gene expression and immune cell infiltration.

## Data Availability

The data generated and analysed during the current study are available in the TCGA Research Network(https://www.cancer.gov/about-nci/organization/ccg/research/structural-genomics/tcga), GTEx (http://commonfund.nih.gov/GTEx/), and GEO (https://www.ncbi.nlm.nih.gov/geo/).

## Abbreviations

ICOS(CD278): Inducible T-cell costimulator
ICOSL: Inducible T-cell co-stimulator ligand
TCGA: The Cancer Genome Atlas
GTEx: Genotypic Tissue Expression Project
TMB: Tumor mutation burden
MSI: Microsatellite instability
MMR: Mismatch repair
DNMTs: DNA methyltransferases
GO: Gene Ontology
KEGG: Kyoto Encyclopedia of Genes and Genomes
GSEA: Gene set enrichment analysis
TIGIT: T cell immune receptor with Ig and ITIM domains
CTLA4: Cytotoxic T Iymphocyte associate protein-4
PD-1: PDCD1
OS: Overall survival
DFS: Disease-free survival
ACC: Adrenocortical Carcinoma
BLCA: Bladder Urothelial Carcinoma
BRCA: Breast invasive carcinoma
CESC: Cervical squamous cell carcinoma and endocervical adenocarcinoma
CHOL: Cholangiocarcinoma
COAD: Colon adenocarcinoma
DLBC: Lymphoid Neoplasm Diffuse Large B-cell Lymphoma
ESCA: Esophageal carcinoma
GBM: Glioblastoma multiforme
HNSC: Head and Neck squamous cell carcinoma
KICH: Kidney Chromophobe
KIRC: Kidney renal clear cell carcinoma
KIRP: Kidney renal papillary cell carcinoma
LAML: Acute Myeloid Leukemia
LGG: Lower Grade Glioma
LIHC: Liver hepatocellular carcinoma
LUAD: Lung adenocarcinoma
LUSC: Lung squamous cell carcinoma
MESO: Mesothelioma
OV: Ovarian serous cystadenocarcinoma
PAAD: Pancreatic adenocarcinoma
PCPG: Pheochromocytoma and Paraganglioma
PRAD: Prostate adenocarcinoma
READ: Rectum adenocarcinoma
SARC: Sarcoma
SKCM: Skin Cutaneous Melanoma
STAD: Stomach adenocarcinoma
TGCT: Testicular Germ Cell Tumors
THCA: Thyroid carcinoma
THYM: Thymoma
UCEC: Uterine Corpus Endometrial Carcinoma
UCS: Uterine Carcinosarcoma
UVM: Uveal Melanoma
COAD/READ: Colon and rectal cancer
