# Supplementary material for: Comprehensive analysis of the role of ICOS ( CD278 ) in pan-cancer prognosis and immunotherapy
Source: BMC Cancer. 2023 Feb 28;23:194. doi: 10.1186/s12885-023-10564-4 (PMC9971684; doi:10.1186/s12885-023-10564-4)
Supplement: Supplementary file 1 — Supplementary Material 1: The expression of ICOS in pan cancer was analyzed using paired tumor/normal samples from TCGA and GTEx databases. [file 12885_2023_10564_MOESM1_ESM.pdf]

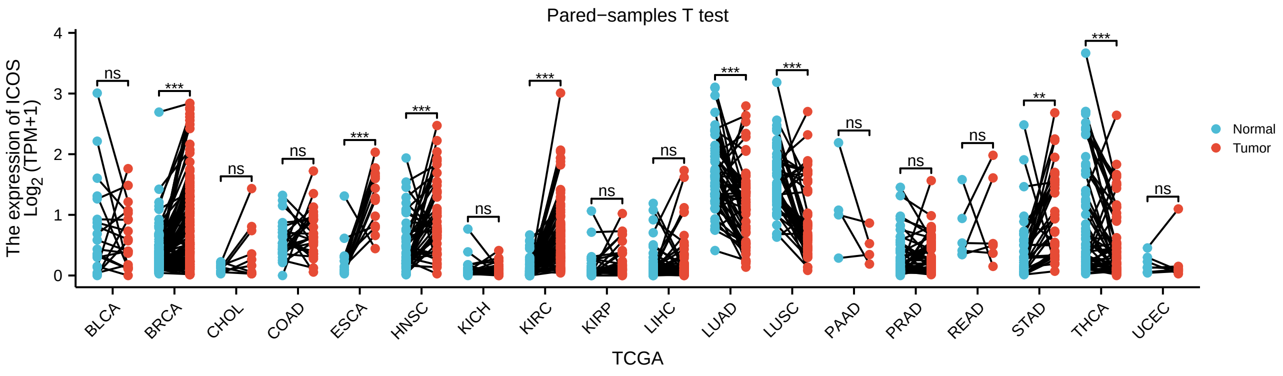

Pared−samples T test
The expression of ICOS
Log2 (TPM+1)
0
1
2
3
4
ns
***
ns
ns
***
***
ns
***
ns
ns
***
***
ns
ns
ns
**
***
ns
BLCA
BRCA
CHOL
COAD
ESCA
HNSC
KICH
KIRC
KIRP
LIHC
LUAD
LUSC
PAAD
PRAD
READ
STAD
THCA
UCEC
TCGA
Normal
Tumor

Supplementary Figure 1.The expression of ICOS in pan cancer was analyzed using paired tumor normal samples from TCGA and GTEx databases.
