# Supplementary material for: Comprehensive analysis of the role of ICOS ( CD278 ) in pan-cancer prognosis and immunotherapy
Source: BMC Cancer. 2023 Feb 28;23:194. doi: 10.1186/s12885-023-10564-4 (PMC9971684; doi:10.1186/s12885-023-10564-4)
Supplement: Supplementary file 2 — Supplementary Material 2: Survival curve analysis of ICOS gene expression in different tumor types. [file 12885_2023_10564_MOESM2_ESM.pdf]

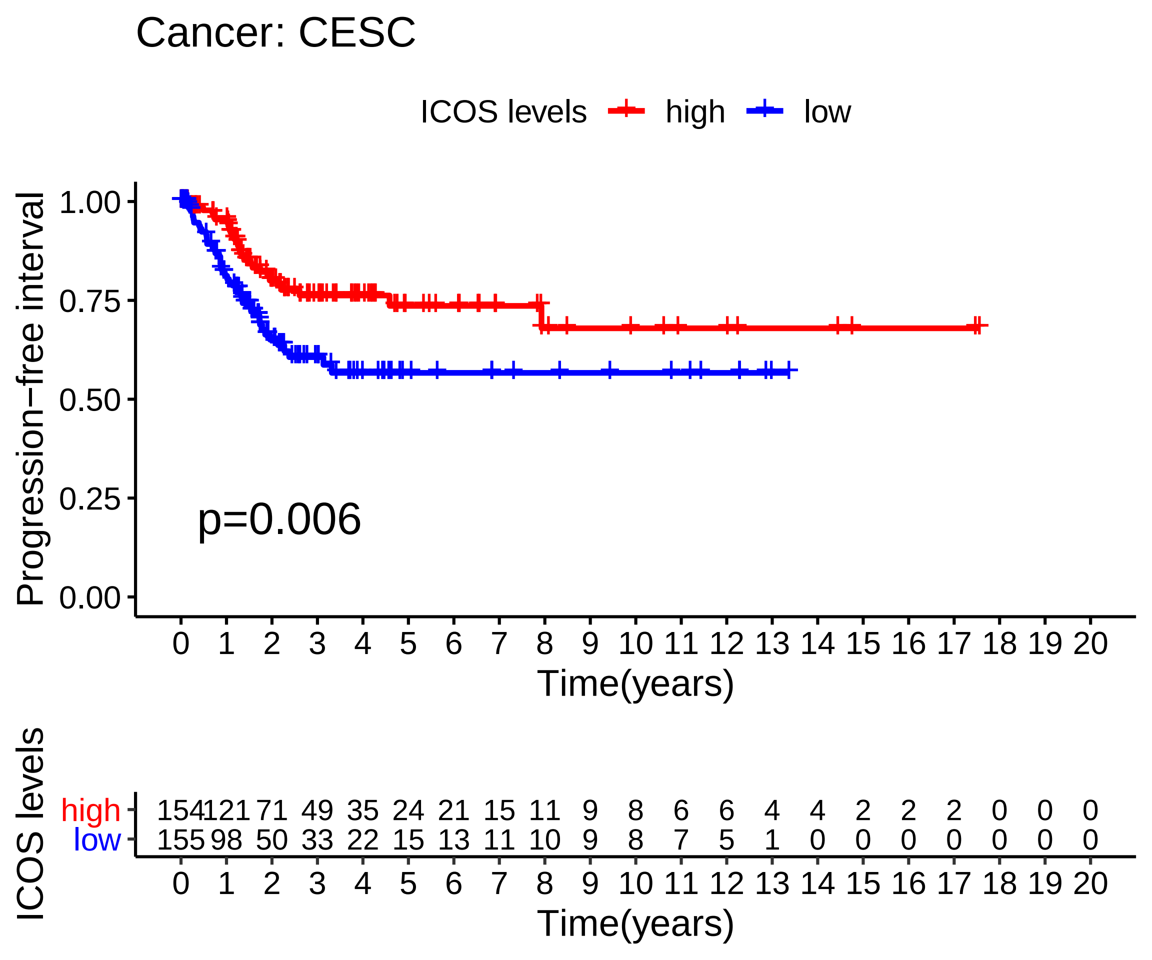
Cancer: CESC
ICOS levels
high
low
Progression−free interval
1.00
0.75
0.50
0.25
0.00
p=0.006
0 1 2 3 4 5 6 7 8 9 10 11 12 13 14 15 16 17 18 19 20
Time(years)
ICOS levels
high 154 121 71 49 35 24 21 15 11 9 8 6 6 4 4 2 2 2 0 0 0
low 155 98 50 33 22 15 13 11 10 9 8 7 5 1 0 0 0 0 0 0 0
0 1 2 3 4 5 6 7 8 9 10 11 12 13 14 15 16 17 18 19 20
Time(years)

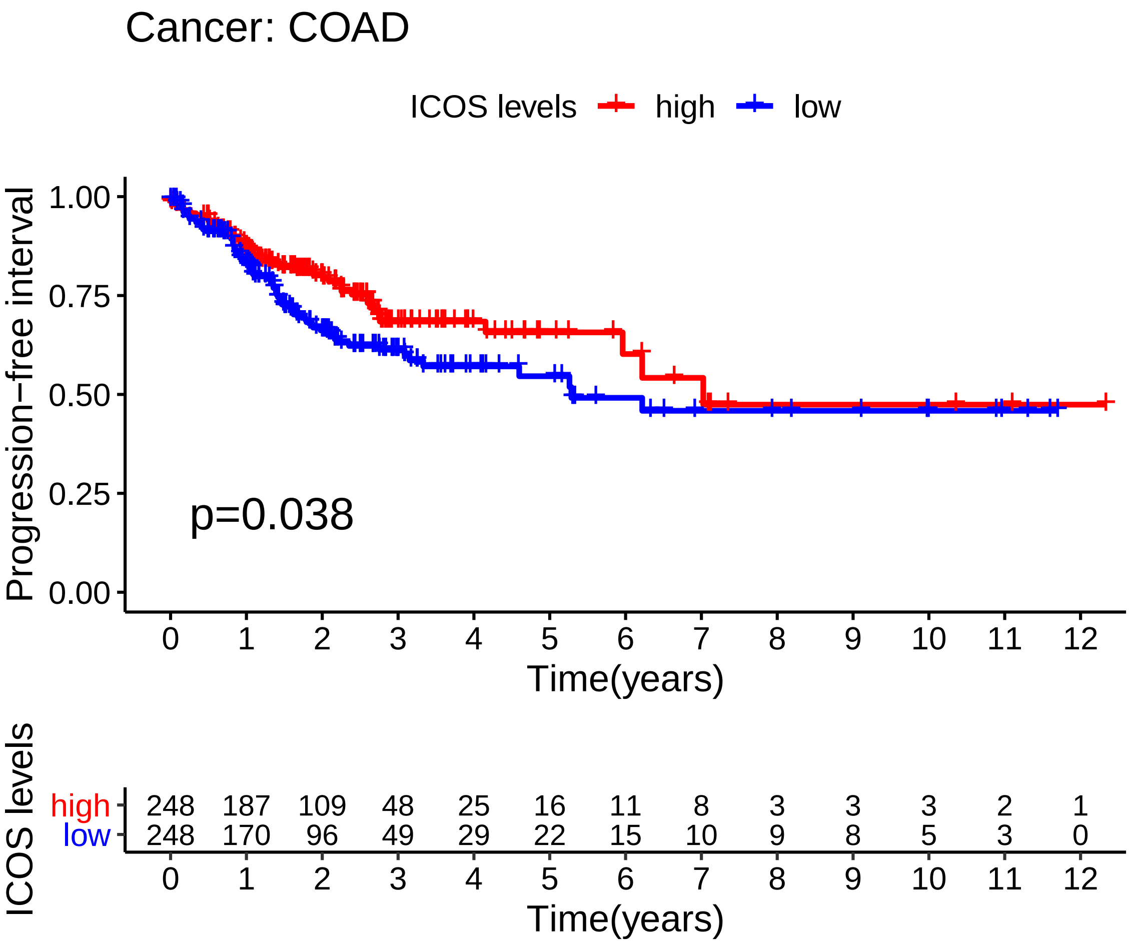
Cancer: COAD
ICOS levels
high
low
Progression−free interval
1.00
0.75
0.50
0.25
0.00
p=0.038
0
1
2
3
4
5
6
7
8
9
10
11
12
Time(years)
ICOS levels
high 248 187 109 48 25 16 11 8 3 3 3 2 1
low 248 170 96 49 29 22 15 10 9 8 5 3 0
Time(years)

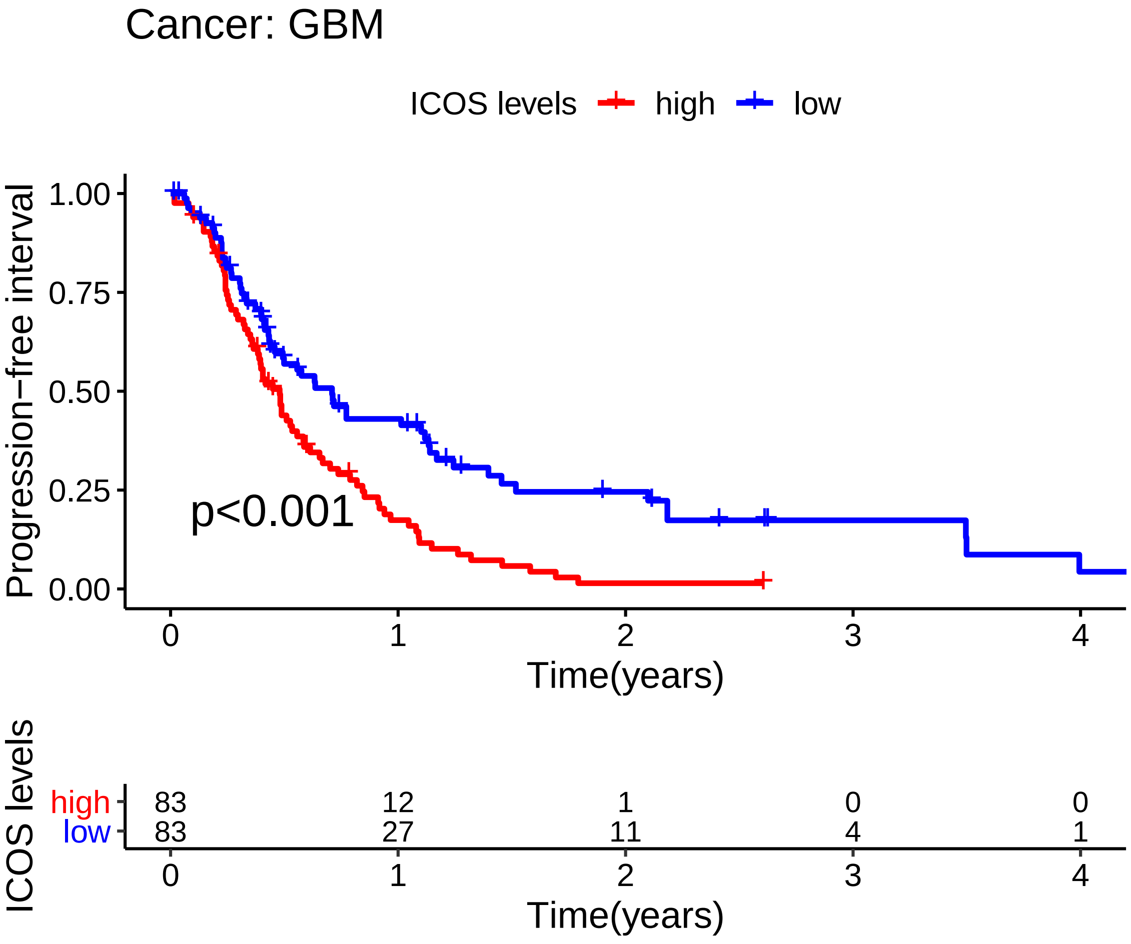

Cancer: GBM
ICOS levels
high
low
Progression−free interval
1.00
0.75
0.50
0.25
0.00
p<0.001
0
1
2
3
4
Time(years)
ICOS levels
high 83 12 1 0 0
low 83 27 11 4 1
Time(years)

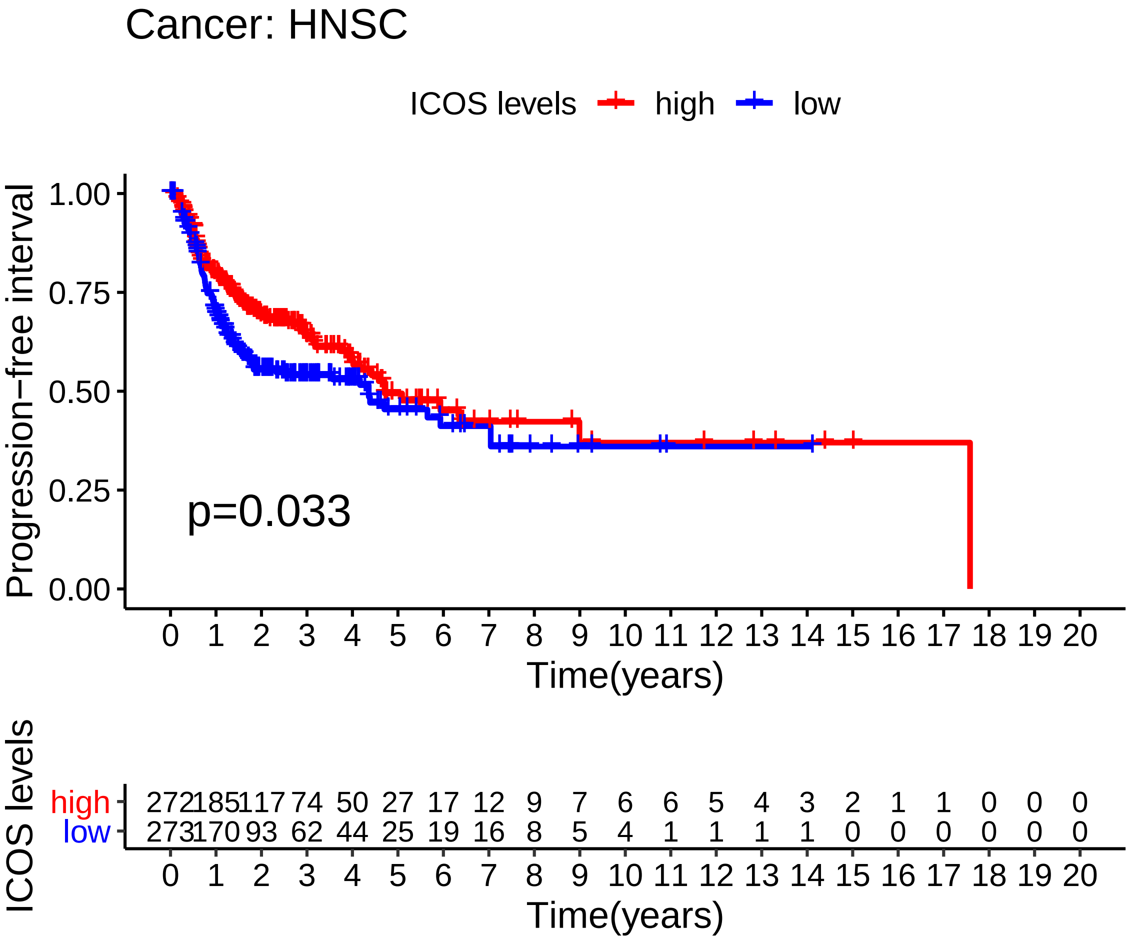

Cancer: HNSC
ICOS levels
high
low
Progression−free interval
1.00
0.75
0.50
0.25
0.00
p=0.033
0 1 2 3 4 5 6 7 8 9 10 11 12 13 14 15 16 17 18 19 20
Time(years)
ICOS levels
high 272 185 117 74 50 27 17 12 9 7 6 6 5 4 3 2 1 1 0 0 0
low 273 170 93 62 44 25 19 16 8 5 4 1 1 1 1 0 0 0 0 0 0
0 1 2 3 4 5 6 7 8 9 10 11 12 13 14 15 16 17 18 19 20
Time(years)

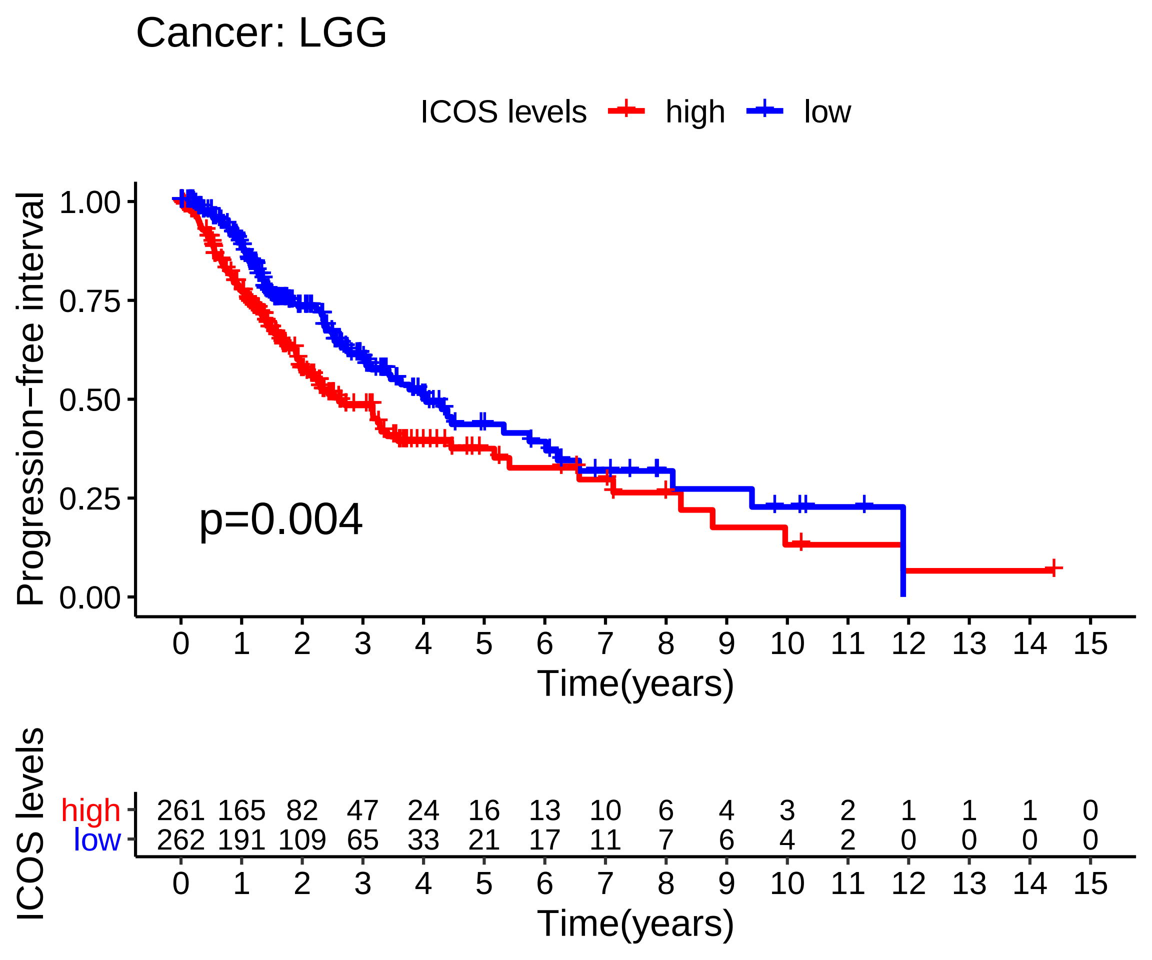

Cancer: LGG
ICOS levels
high
low
Progression−free interval
1.00
0.75
0.50
0.25
0.00
p=0.004
0 1 2 3 4 5 6 7 8 9 10 11 12 13 14 15
Time(years)
ICOS levels
high 261 165 82 47 24 16 13 10 6 4 3 2 1 1 1 0
low 262 191 109 65 33 21 17 11 7 6 4 2 0 0 0 0
0 1 2 3 4 5 6 7 8 9 10 11 12 13 14 15
Time(years)

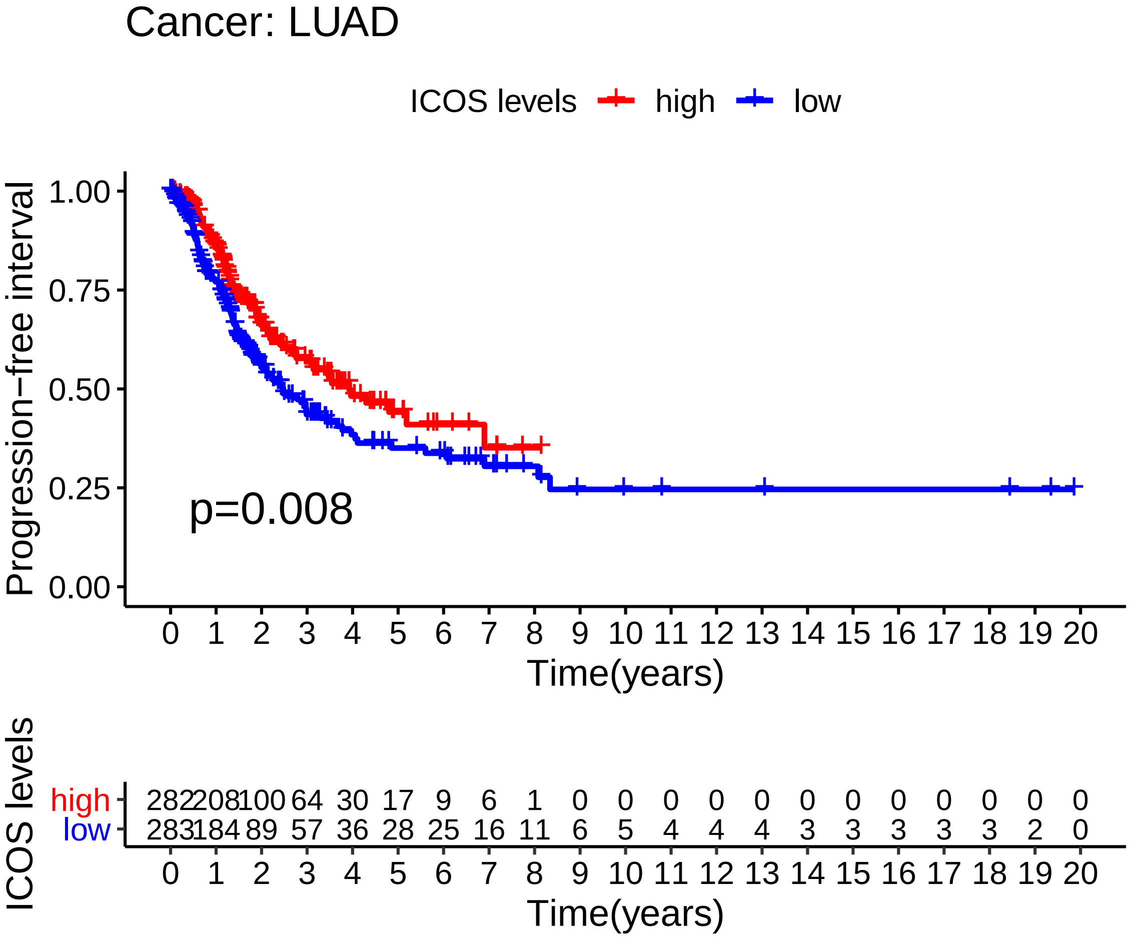
Cancer: LUAD
ICOS levels
high
low
Progression−free interval
1.00
0.75
0.50
0.25
0.00
p=0.008
0 1 2 3 4 5 6 7 8 9 10 11 12 13 14 15 16 17 18 19 20
Time(years)
ICOS levels
high 282 208 100 64 30 17 9 6 1 0 0 0 0 0 0 0 0 0 0 0 0
low 283 184 89 57 36 28 25 16 11 6 5 4 4 4 3 3 3 3 3 2 0
0 1 2 3 4 5 6 7 8 9 10 11 12 13 14 15 16 17 18 19 20
Time(years)

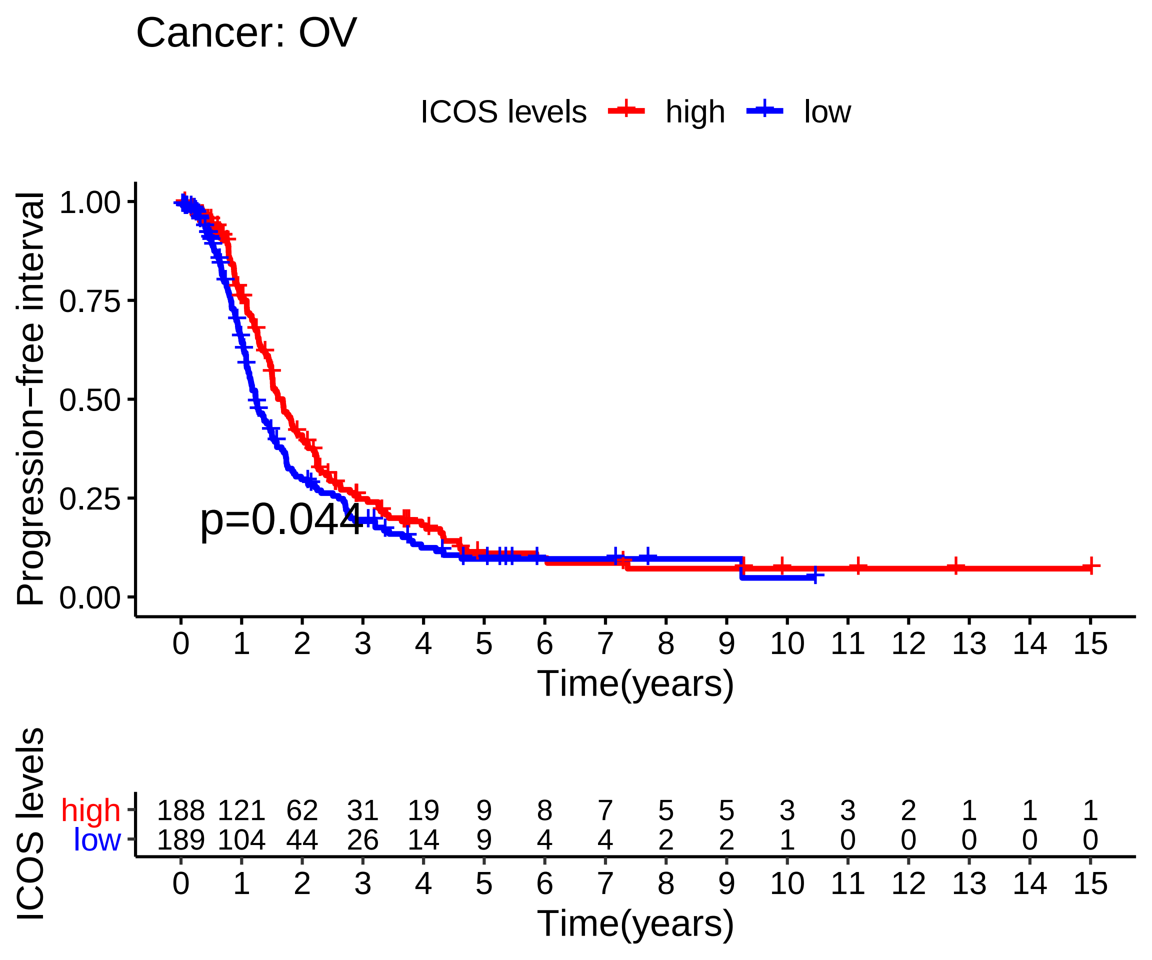
Cancer: OV
ICOS levels
high
low
Progression−free interval
1.00
0.75
0.50
0.25
0.00
p=0.044
0 1 2 3 4 5 6 7 8 9 10 11 12 13 14 15
Time(years)
ICOS levels
high 188 121 62 31 19 9 8 7 5 5 3 3 2 1 1 1
low 189 104 44 26 14 9 4 4 2 2 1 0 0 0 0 0
0 1 2 3 4 5 6 7 8 9 10 11 12 13 14 15
Time(years)

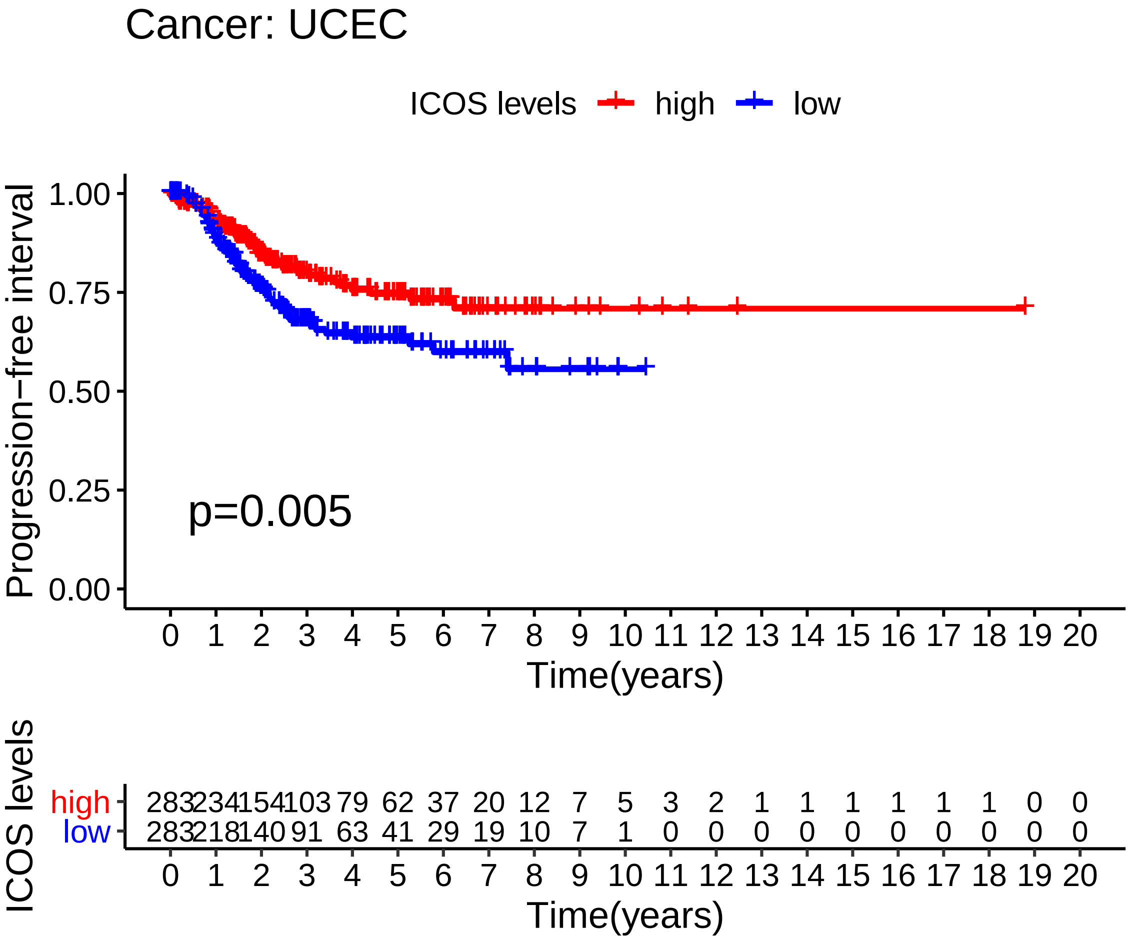
Cancer: UCEC
ICOS levels
high
low
Progression−free interval
1.00
0.75
0.50
0.25
0.00
p=0.005
0 1 2 3 4 5 6 7 8 9 10 11 12 13 14 15 16 17 18 19 20
Time(years)
ICOS levels
high 283 234 154 103 79 62 37 20 12 7 5 3 2 1 1 1 1 1 1 0 0
low 283 218 140 91 63 41 29 19 10 7 1 0 0 0 0 0 0 0 0 0 0
0 1 2 3 4 5 6 7 8 9 10 11 12 13 14 15 16 17 18 19 20
Time(years)

Supplementary Figure 2. Survival curve analysis of ICOS gene expression in different tumor types.
