# Supplementary material for: Comprehensive analysis of the role of ICOS ( CD278 ) in pan-cancer prognosis and immunotherapy
Source: BMC Cancer. 2023 Feb 28;23:194. doi: 10.1186/s12885-023-10564-4 (PMC9971684; doi:10.1186/s12885-023-10564-4)
Supplement: Supplementary file 3 — Supplementary Material 3: The relationship between ICOS gene expression and immune cell infiltration. [file 12885_2023_10564_MOESM3_ESM.pdf]

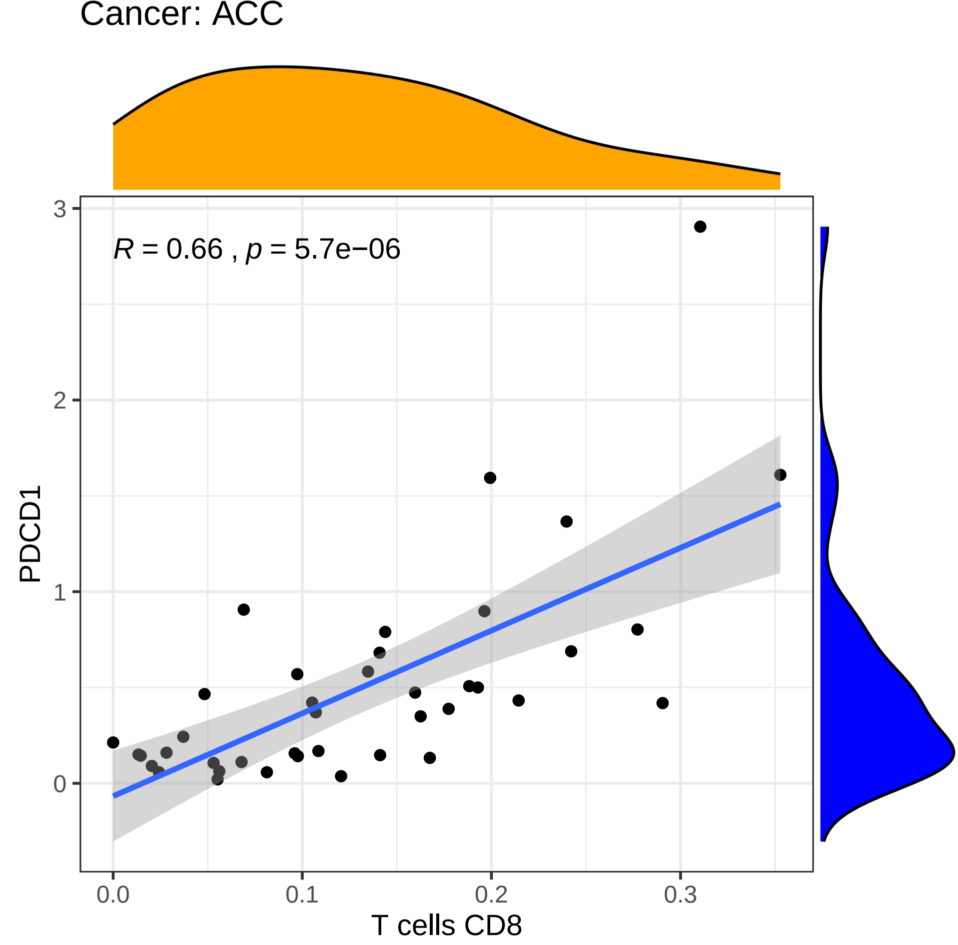

Cancer: ACC
R = 0.66 , p = 5.7e−06
PDCD1
3
2
1
0
0.0
0.1
0.2
0.3
T cells CD8

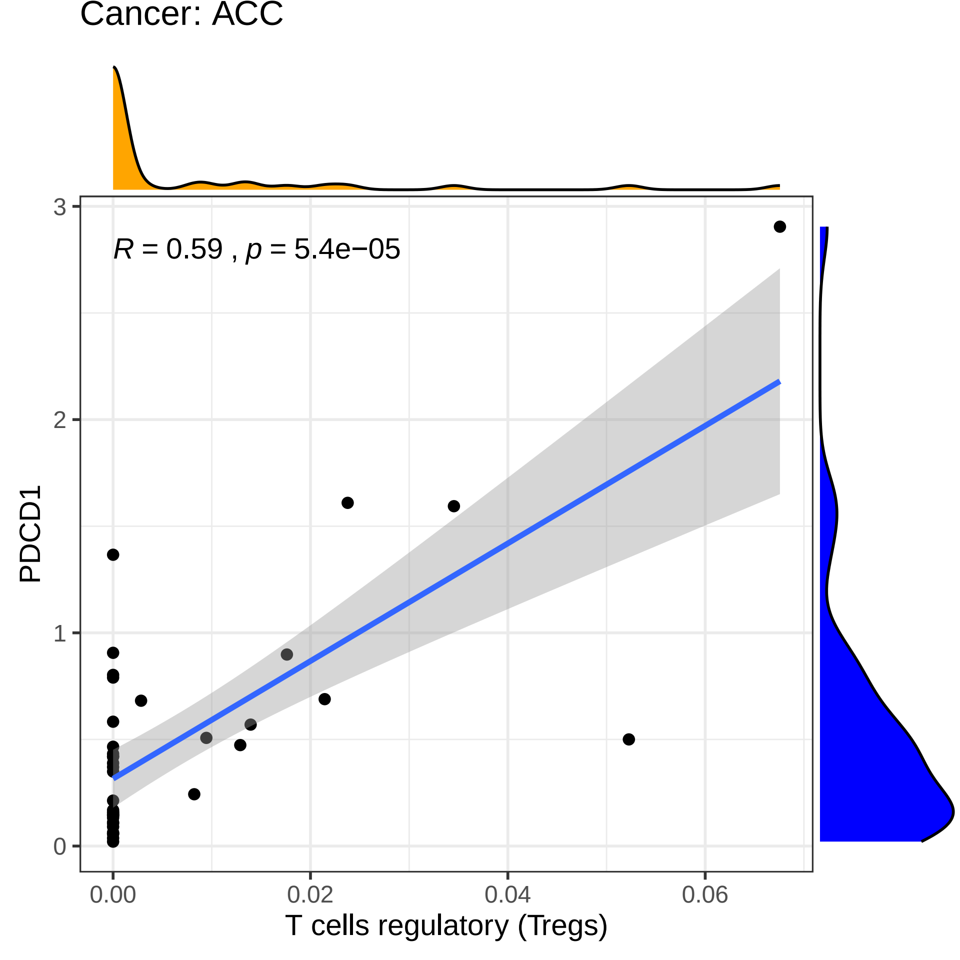

Cancer: ACC
R = 0.59 , p = 5.4e−05
3
2
1
0
0.00
0.02
0.04
0.06
PDCD1
T cells regulatory (Tregs)

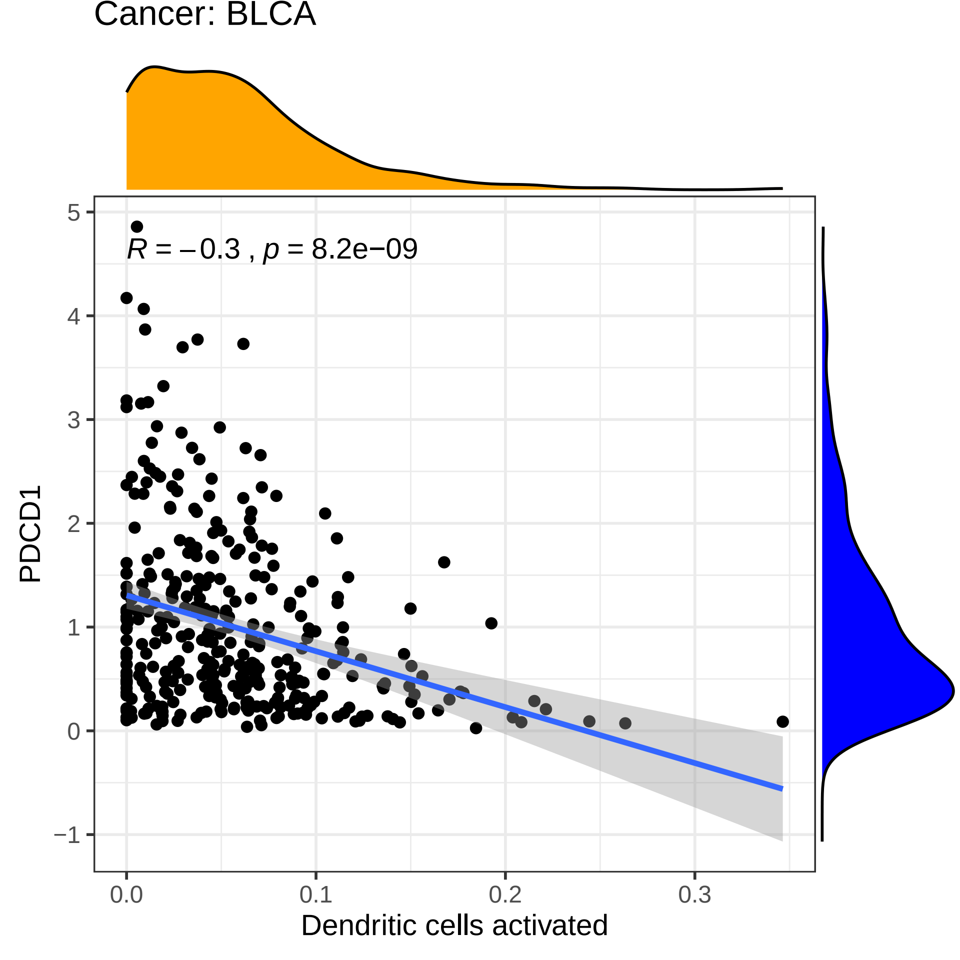

Cancer: BLCA
R = −0.3 , p = 8.2e−09
PDCD1
Dendritic cells activated
5
4
3
2
1
0
−1
0.0
0.1
0.2
0.3

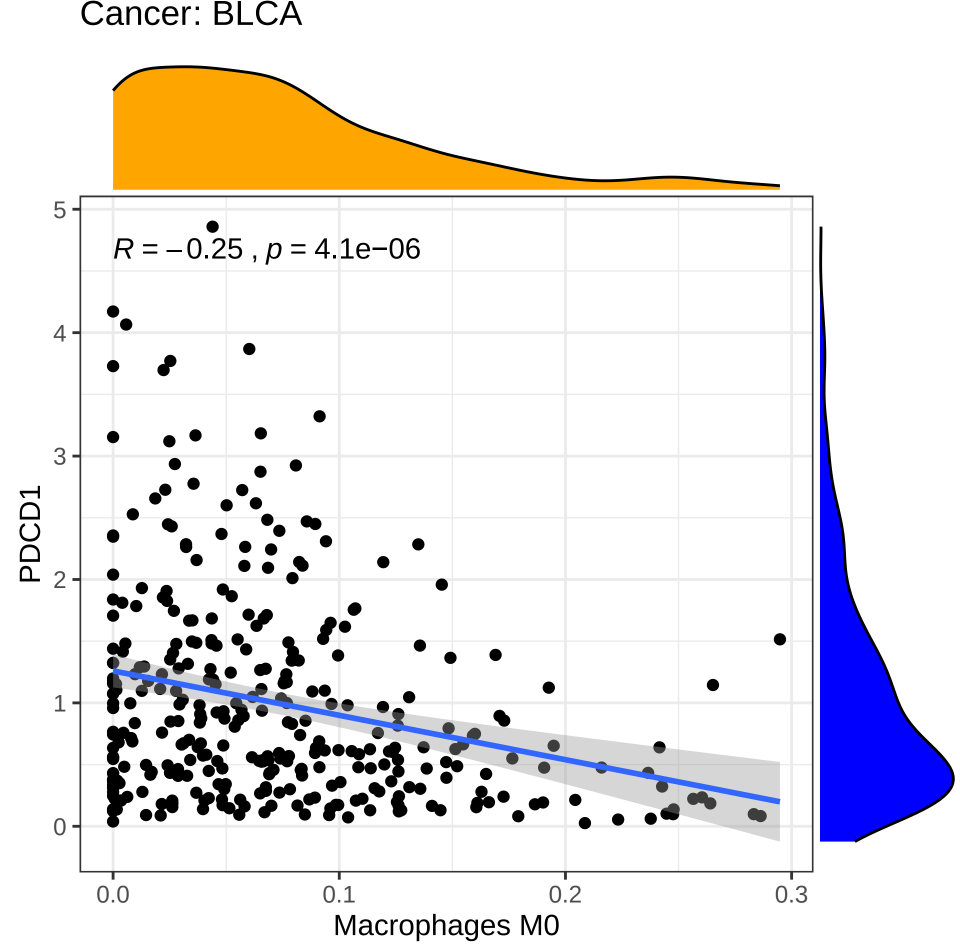

Cancer: BLCA
R = −0.25 , p = 4.1e−06
PDCD1
0
1
2
3
4
5
0.0
0.1
0.2
0.3
Macrophages M0

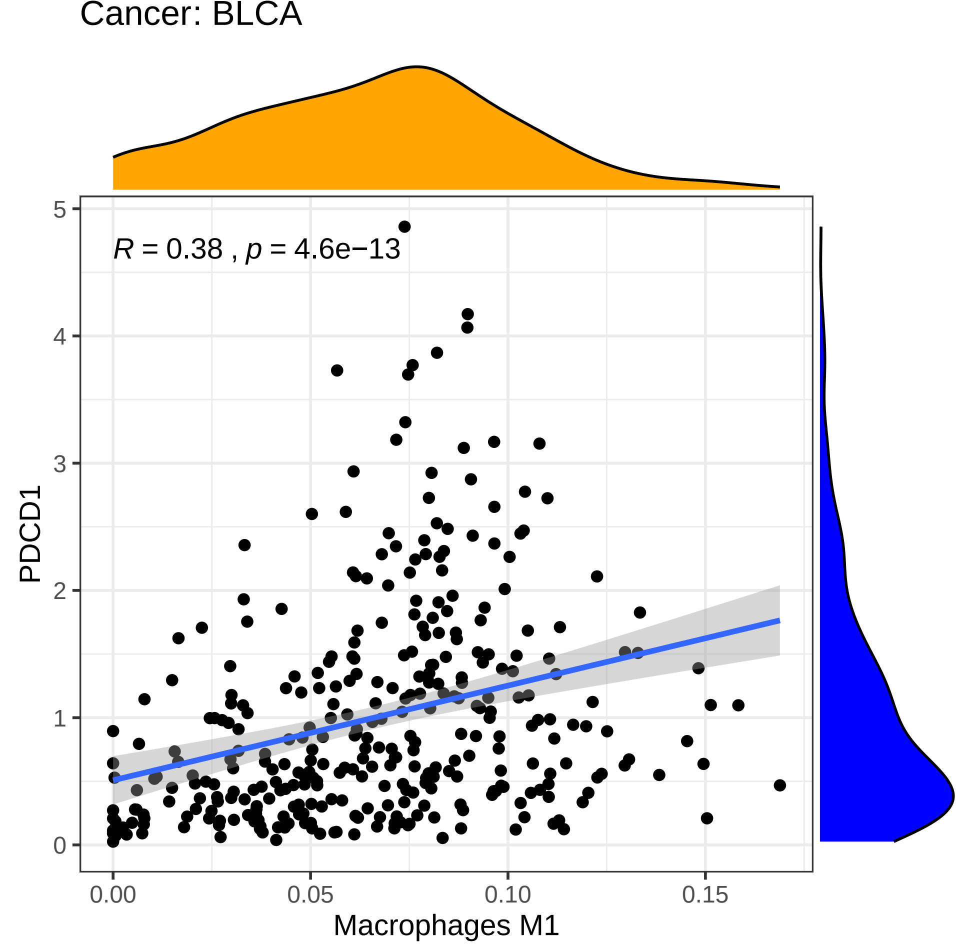
Cancer: BLCA
R = 0.38 , p = 4.6e−13
PDCD1
Macrophages M1
0
1
2
3
4
5
0.00
0.05
0.10
0.15

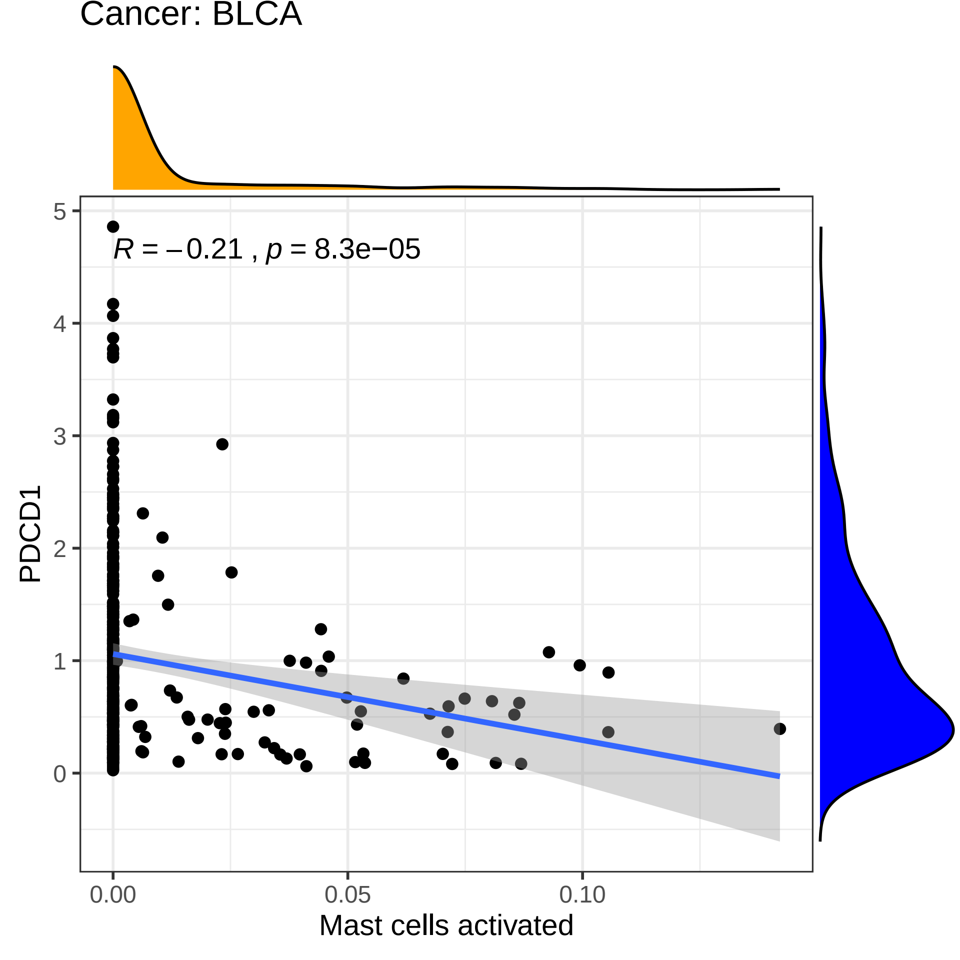

Cancer: BLCA
R = −0.21 , p = 8.3e−05
PDCD1
Mast cells activated
0
1
2
3
4
5
0.00
0.05
0.10

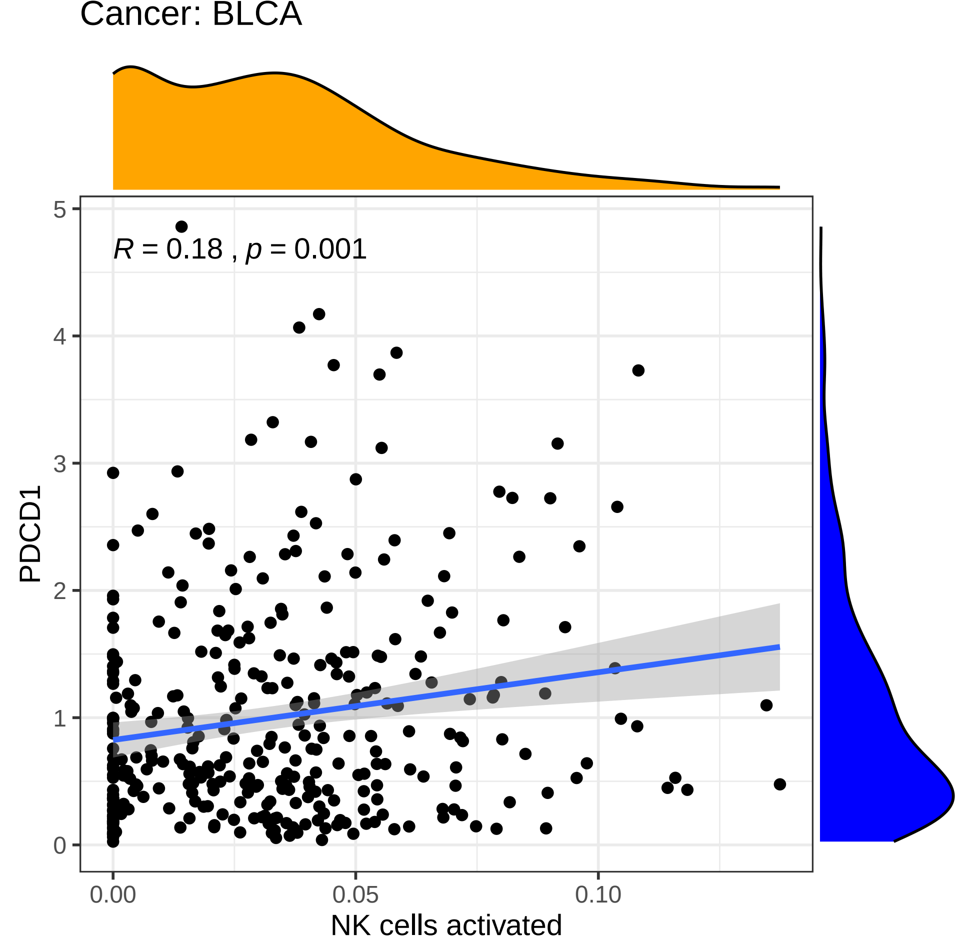

Cancer: BLCA
R = 0.18 , p = 0.001
PDCD1
NK cells activated
0
1
2
3
4
5
0.00
0.05
0.10

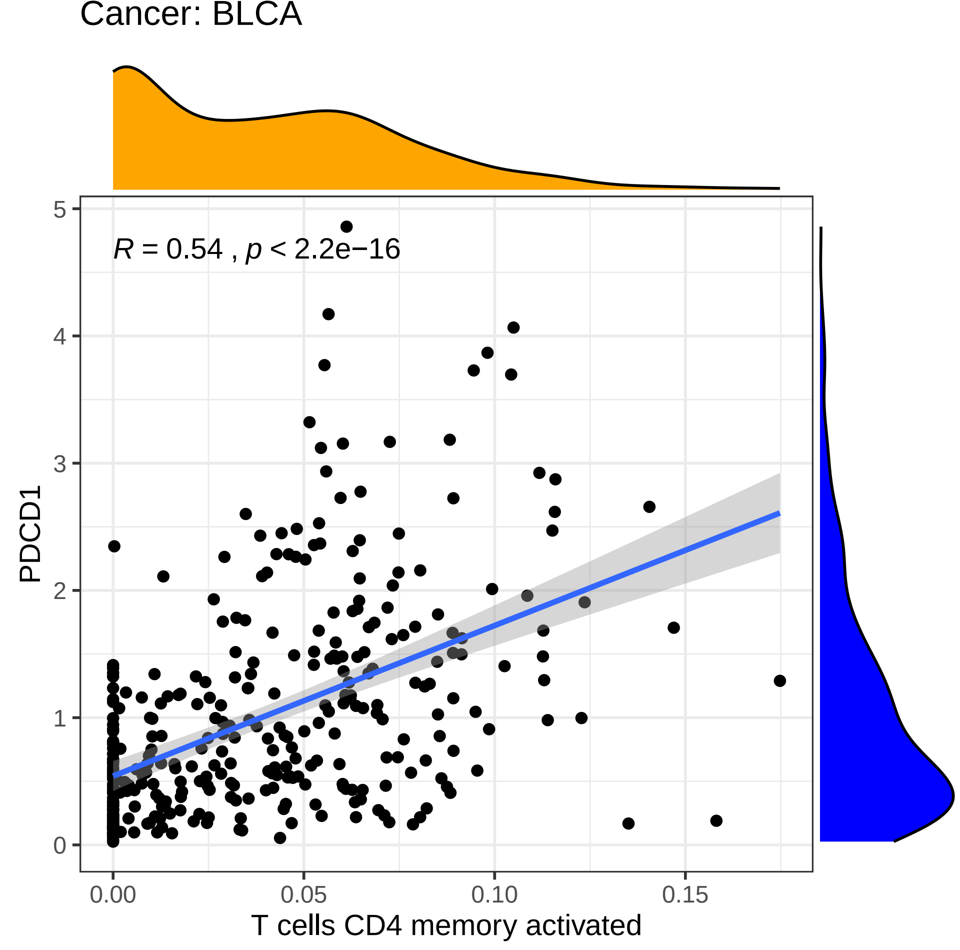
Cancer: BLCA
R = 0.54 , p < 2.2e−16
PDCD1
T cells CD4 memory activated
0
1
2
3
4
5
0.00
0.05
0.10
0.15

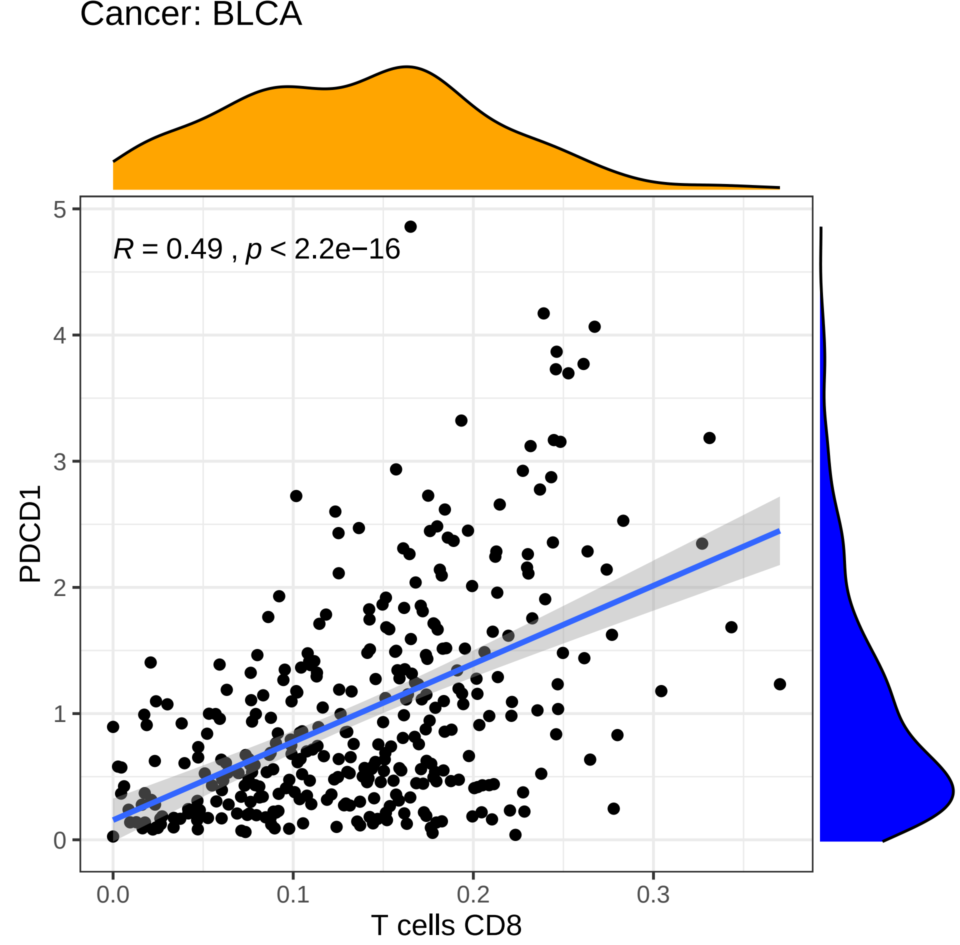
Cancer: BLCA
R = 0.49 , p < 2.2e−16
PDCD1
T cells CD8
0
1
2
3
4
5
0.0
0.1
0.2
0.3

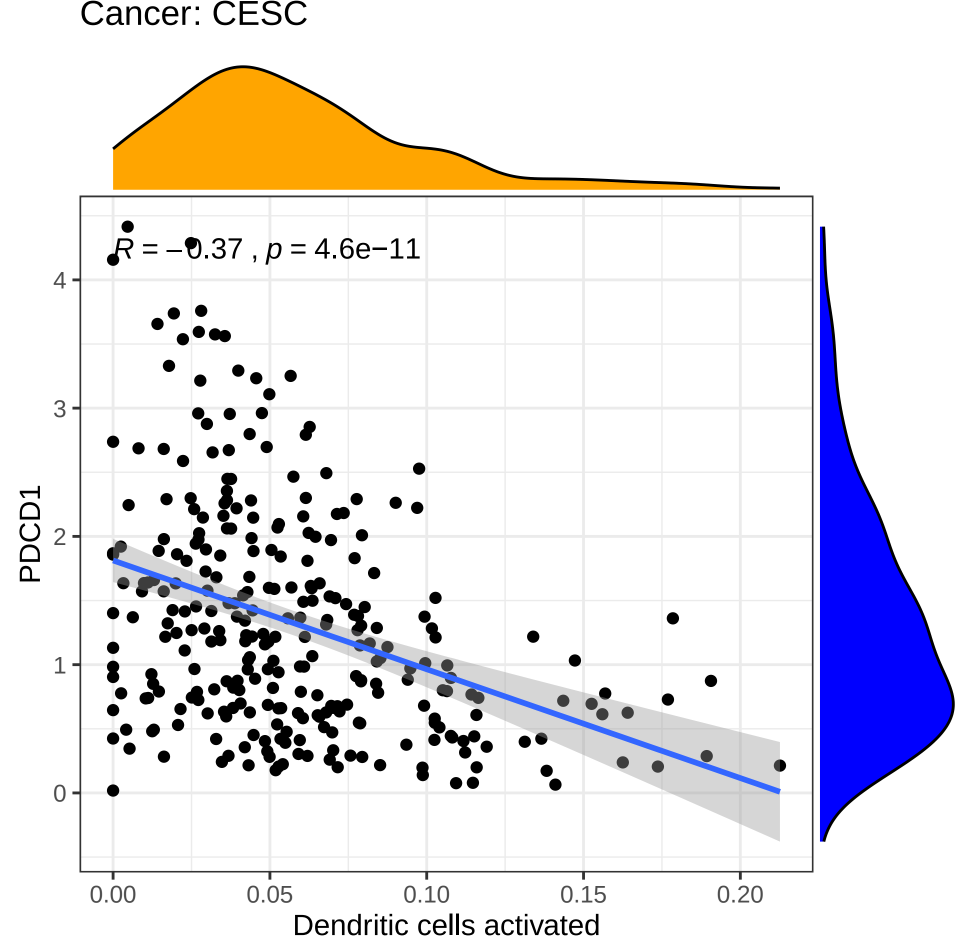

Cancer: CESC
R = −0.37 , p = 4.6e−11
4
3
2
1
0
PDCD1
0.00
0.05
0.10
0.15
0.20
Dendritic cells activated

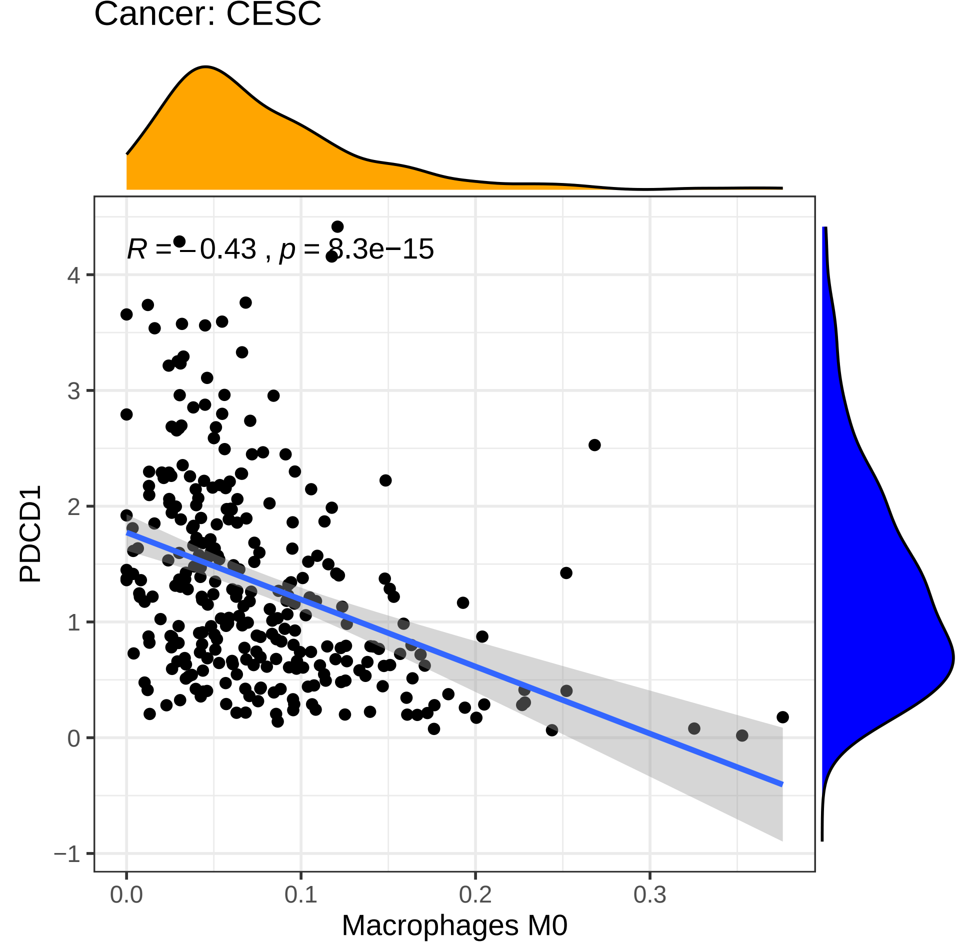

Cancer: CESC
R = −0.43 , p = 8.3e−15
PDCD1
Macrophages M0
4
3
2
1
0
−1
0.0
0.1
0.2
0.3

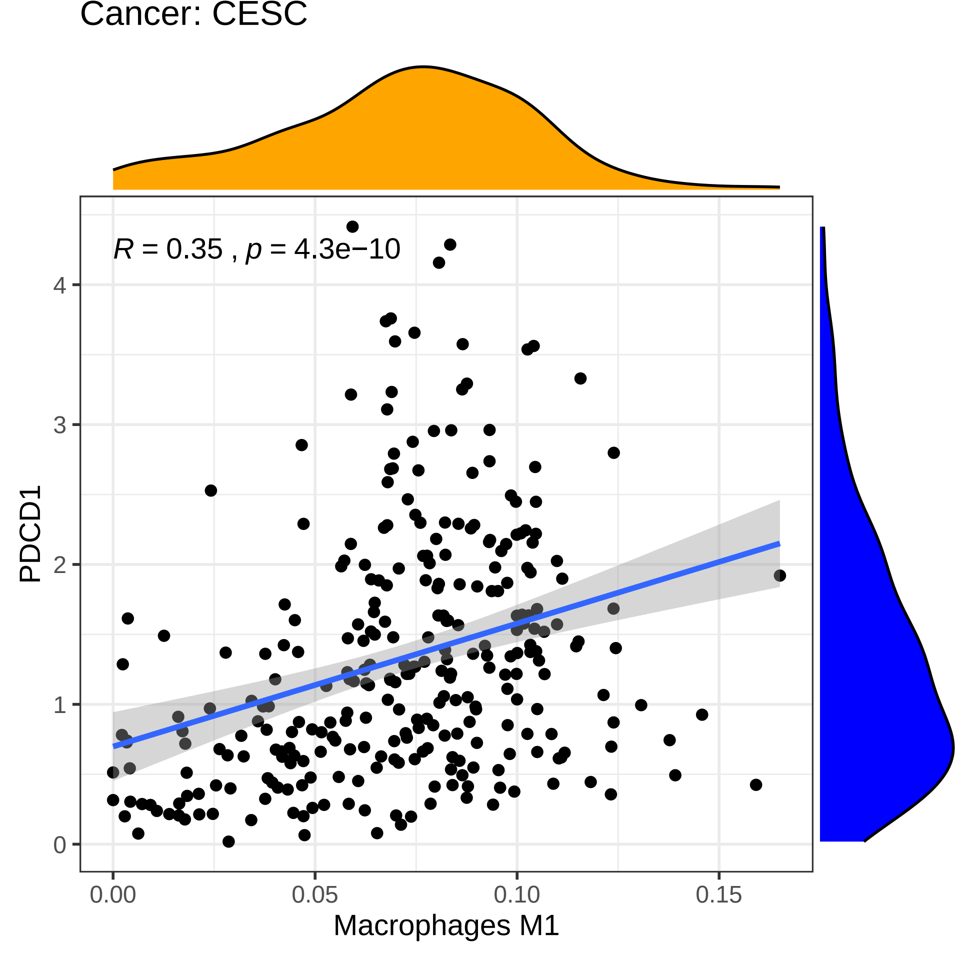

Cancer: CESC
R = 0.35 , p = 4.3e−10
PDCD1
Macrophages M1
0
1
2
3
4
0.00
0.05
0.10
0.15

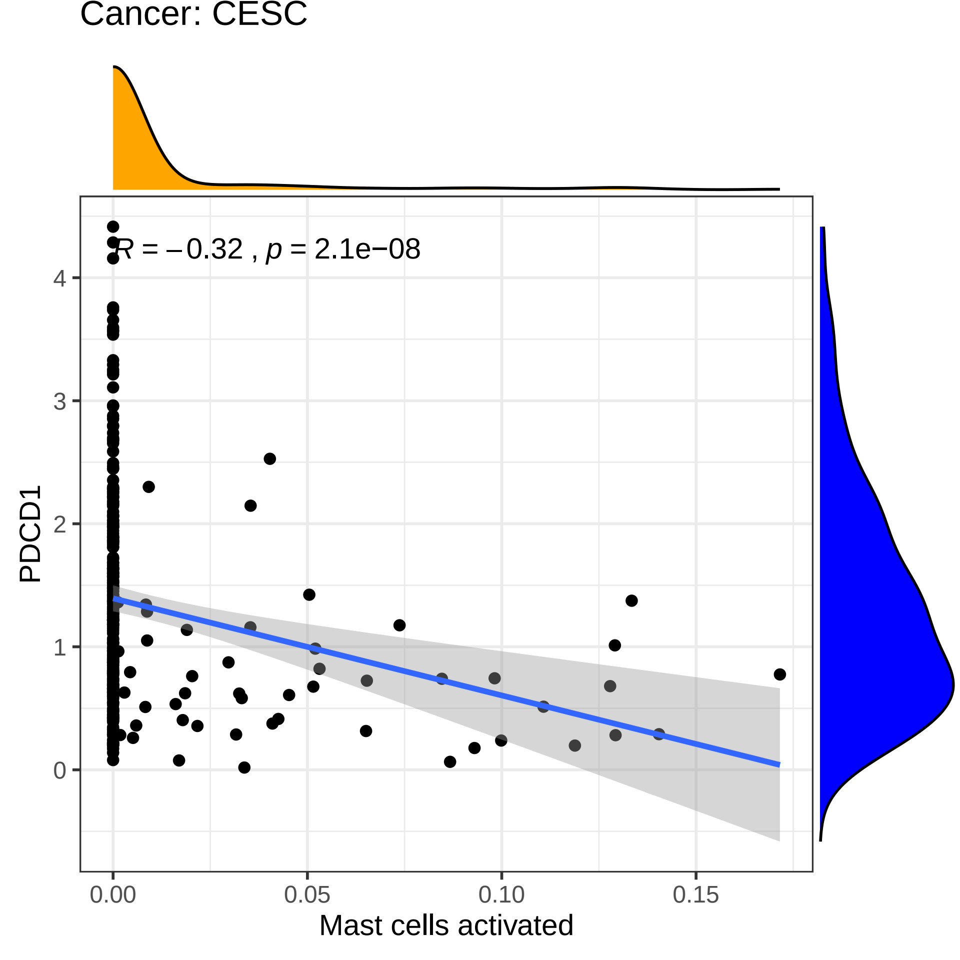

Cancer: CESC
R = −0.32 , p = 2.1e−08
PDCD1
0
1
2
3
4
0.00
0.05
0.10
0.15
Mast cells activated

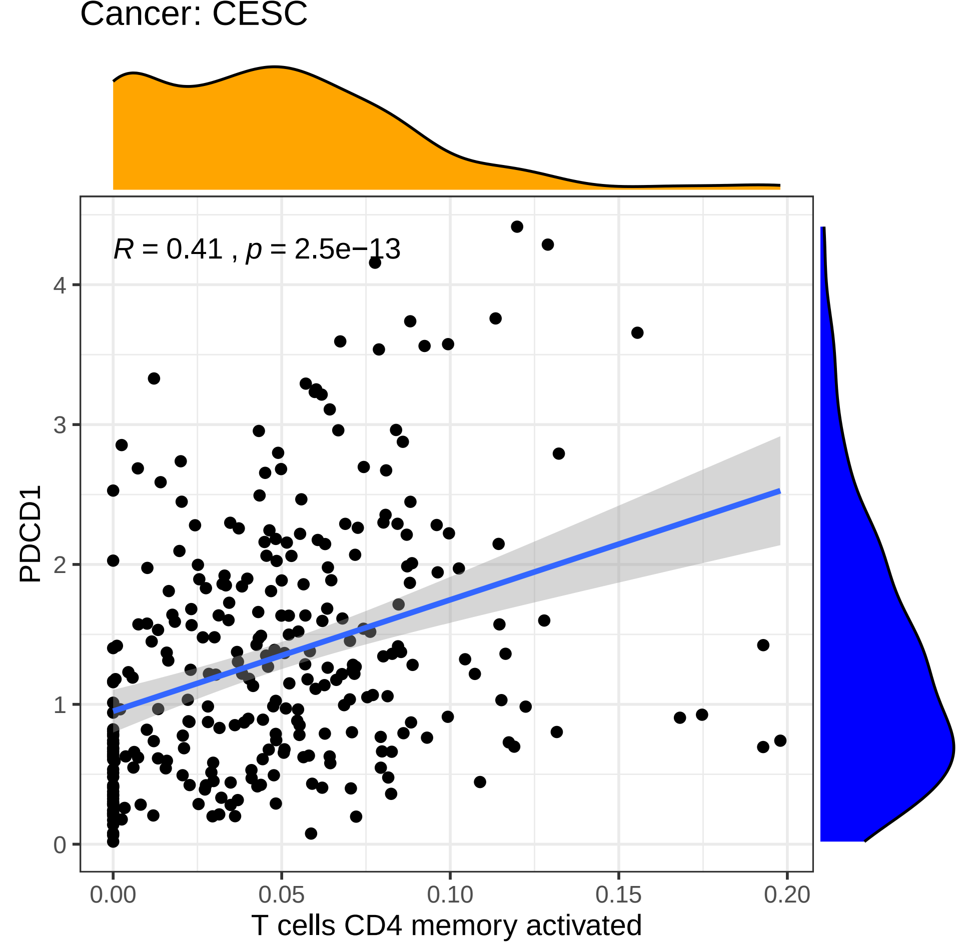

Cancer: CESC
R = 0.41 , p = 2.5e−13
PDCD1
0
1
2
3
4
0.00
0.05
0.10
0.15
0.20
T cells CD4 memory activated

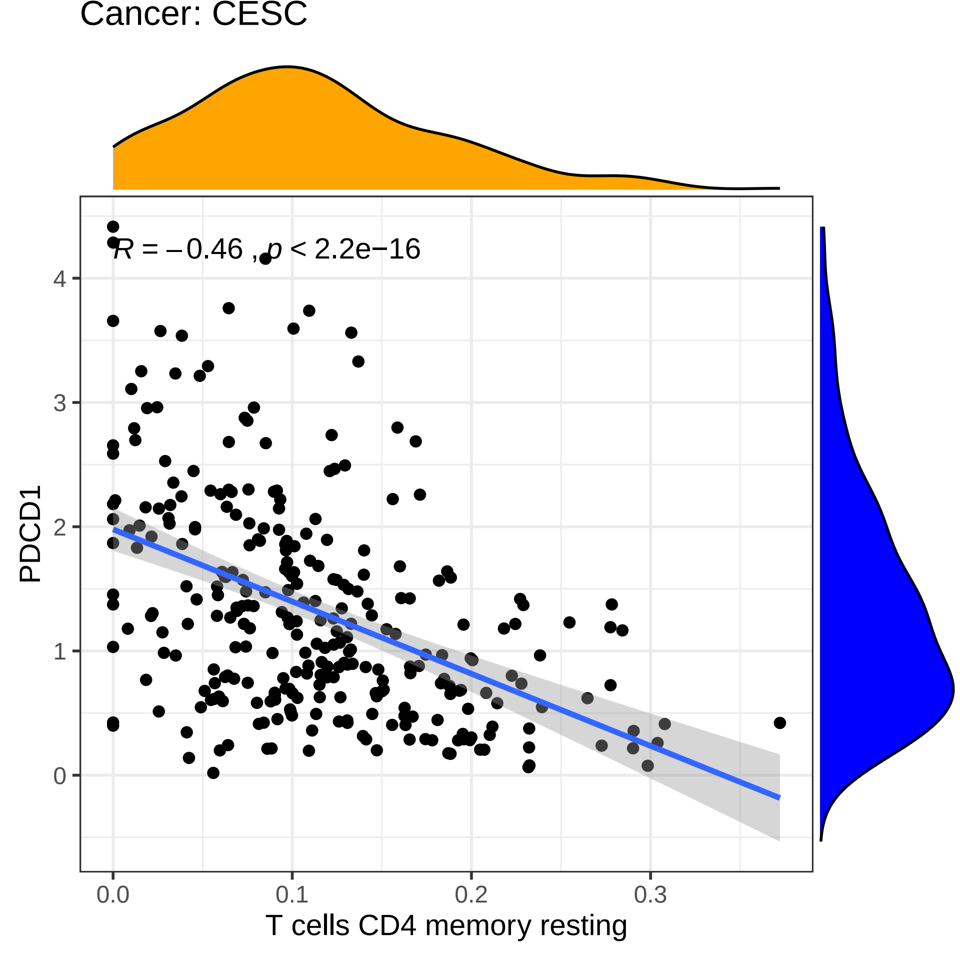

Cancer: CESC
R = −0.46 , p < 2.2e−16
PDCD1
0
1
2
3
4
0.0
0.1
0.2
0.3
T cells CD4 memory resting

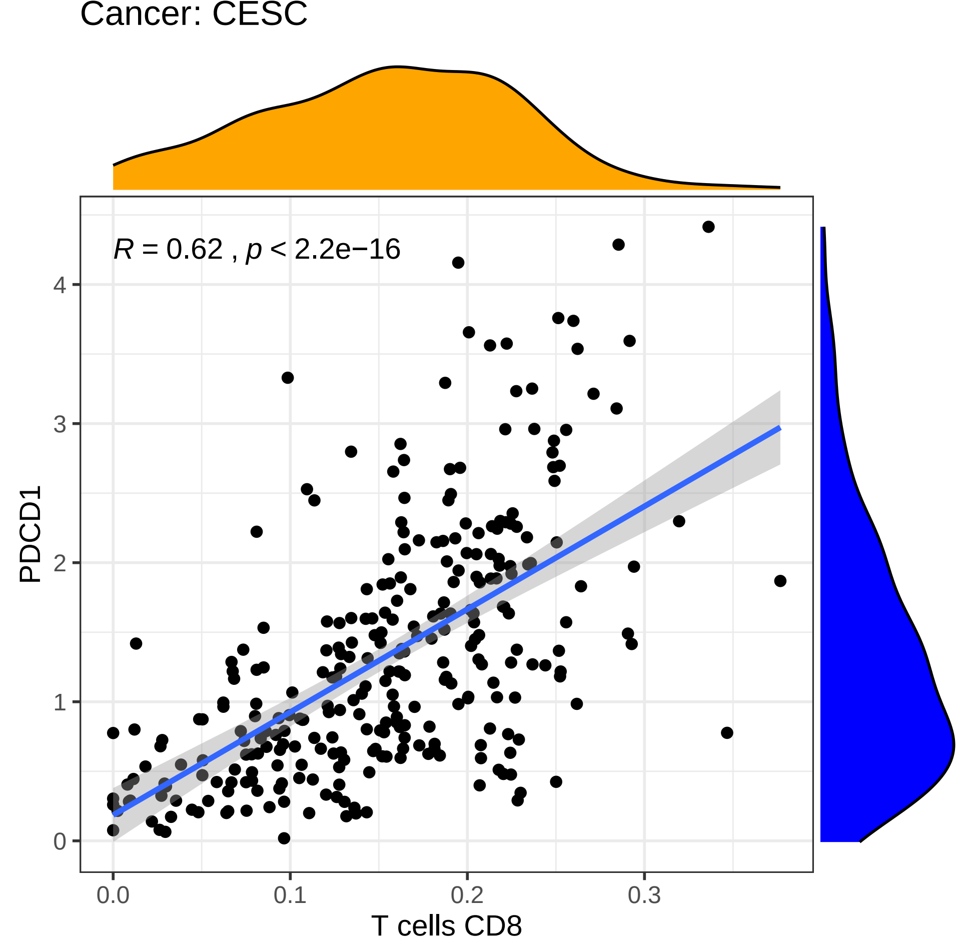

Cancer: CESC
R = 0.62 , p < 2.2e−16
PDCD1
0
1
2
3
4
0.0
0.1
0.2
0.3
T cells CD8

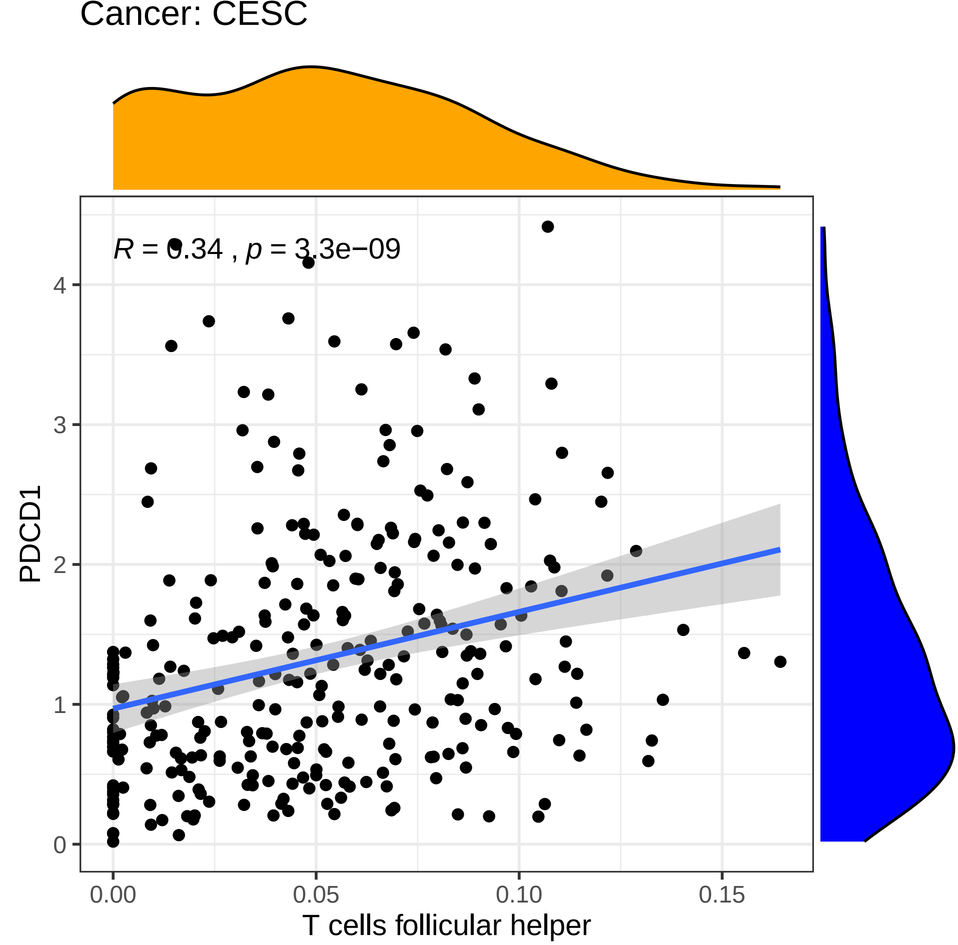

Cancer: CESC
R = 0.34 , p = 3.3e−09
PDCD1
0
1
2
3
4
0.00
0.05
0.10
0.15
T cells follicular helper

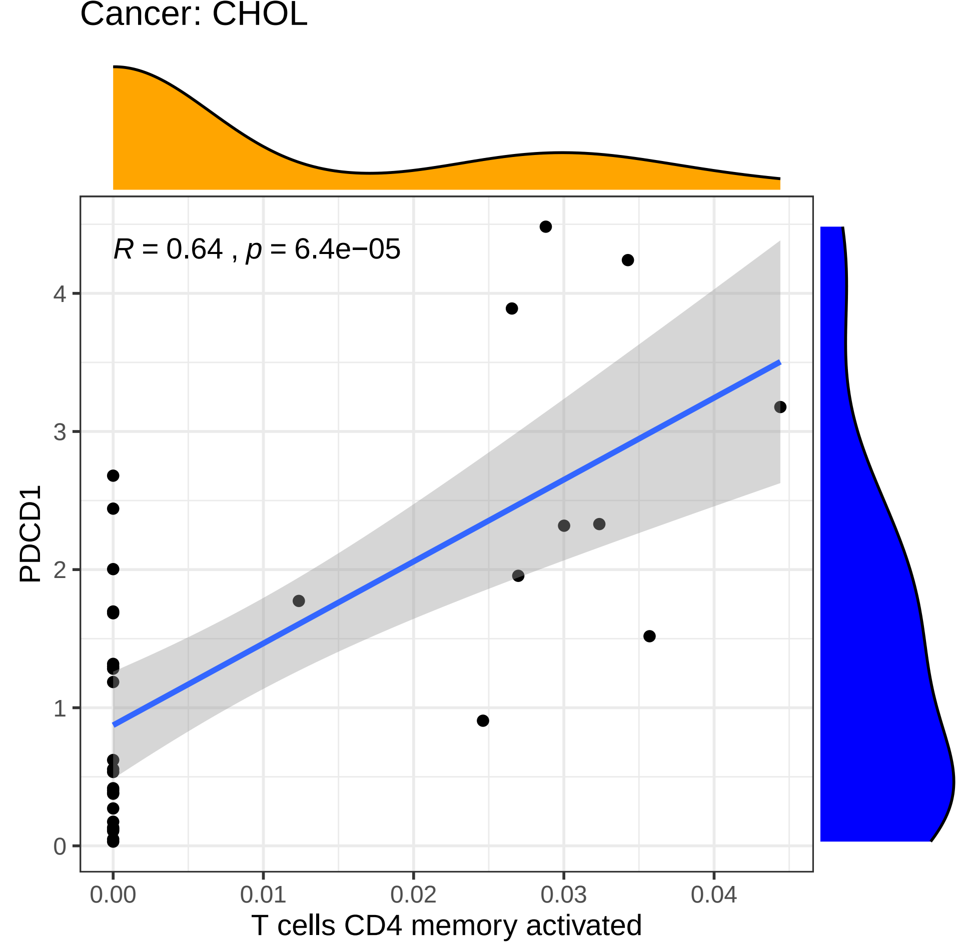

Cancer: CHOL
R = 0.64 , p = 6.4e−05
PDCD1
0
1
2
3
4
0.00
0.01
0.02
0.03
0.04
T cells CD4 memory activated

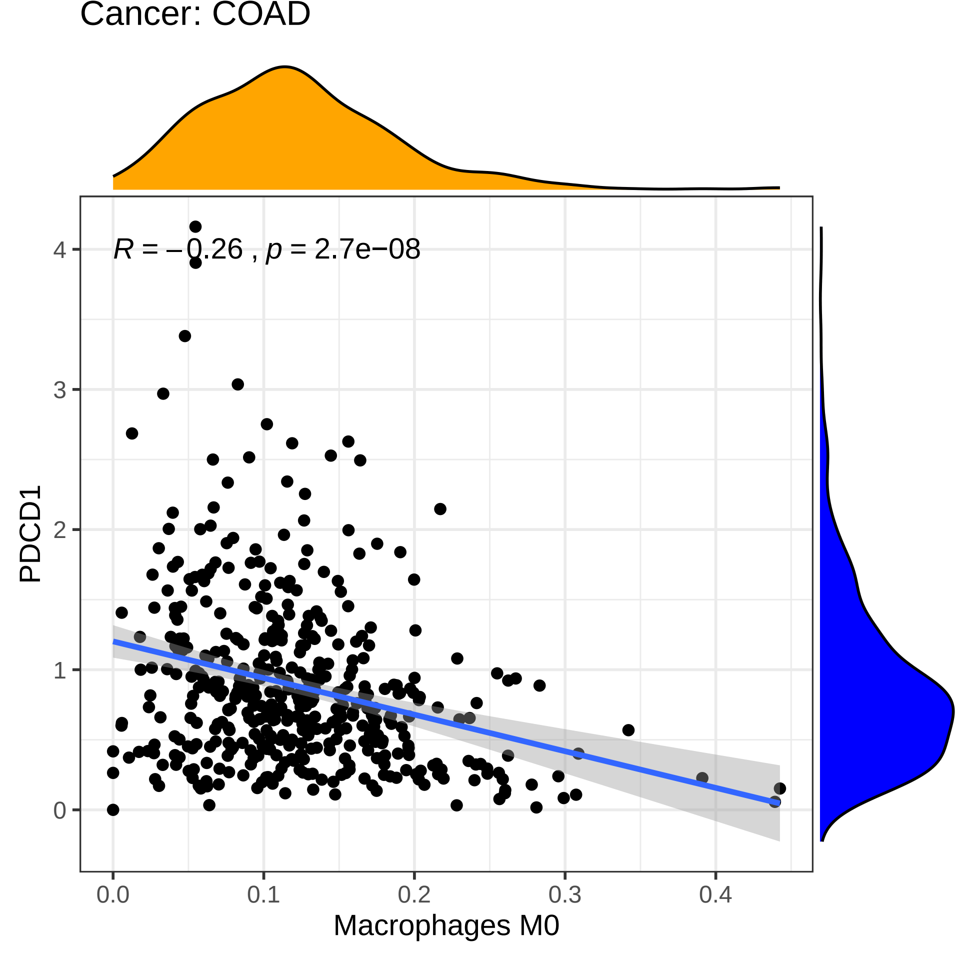
Cancer: COAD
R = −0.26 , p = 2.7e−08
PDCD1
Macrophages M0
0
1
2
3
4
0.0
0.1
0.2
0.3
0.4

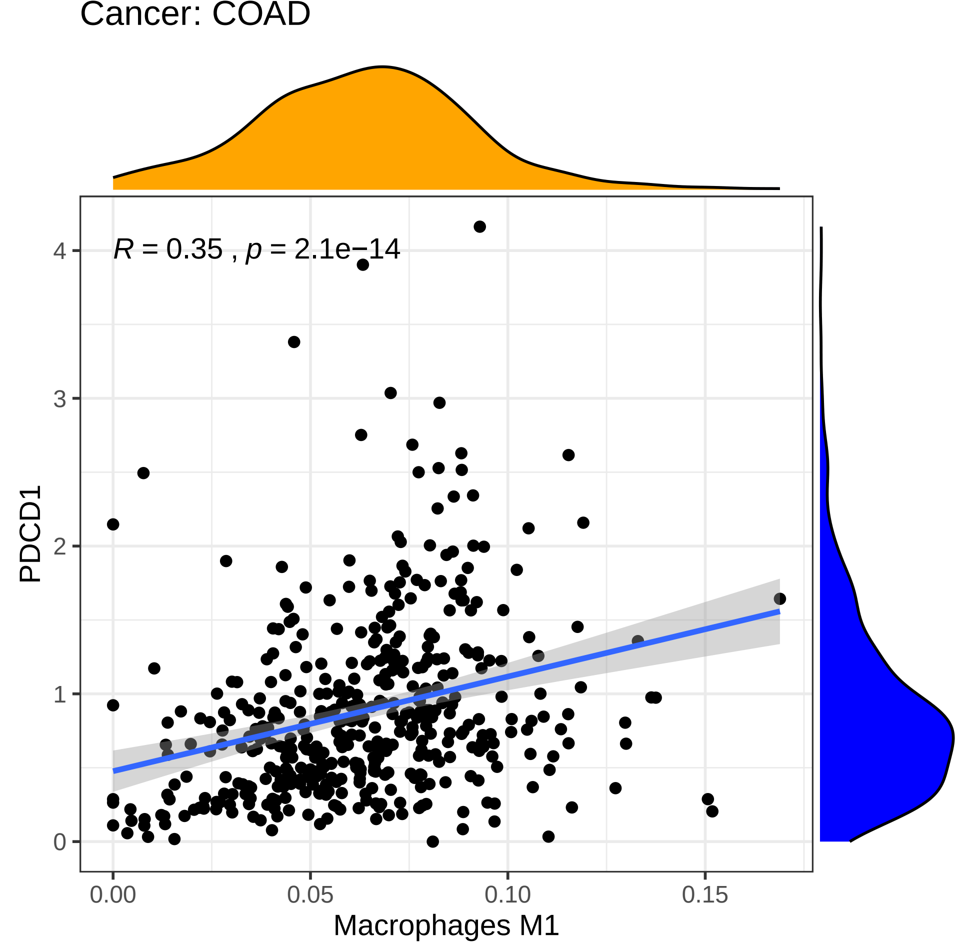

Cancer: COAD
R = 0.35 , p = 2.1e−14
PDCD1
Macrophages M1
0
1
2
3
4
0.00
0.05
0.10
0.15

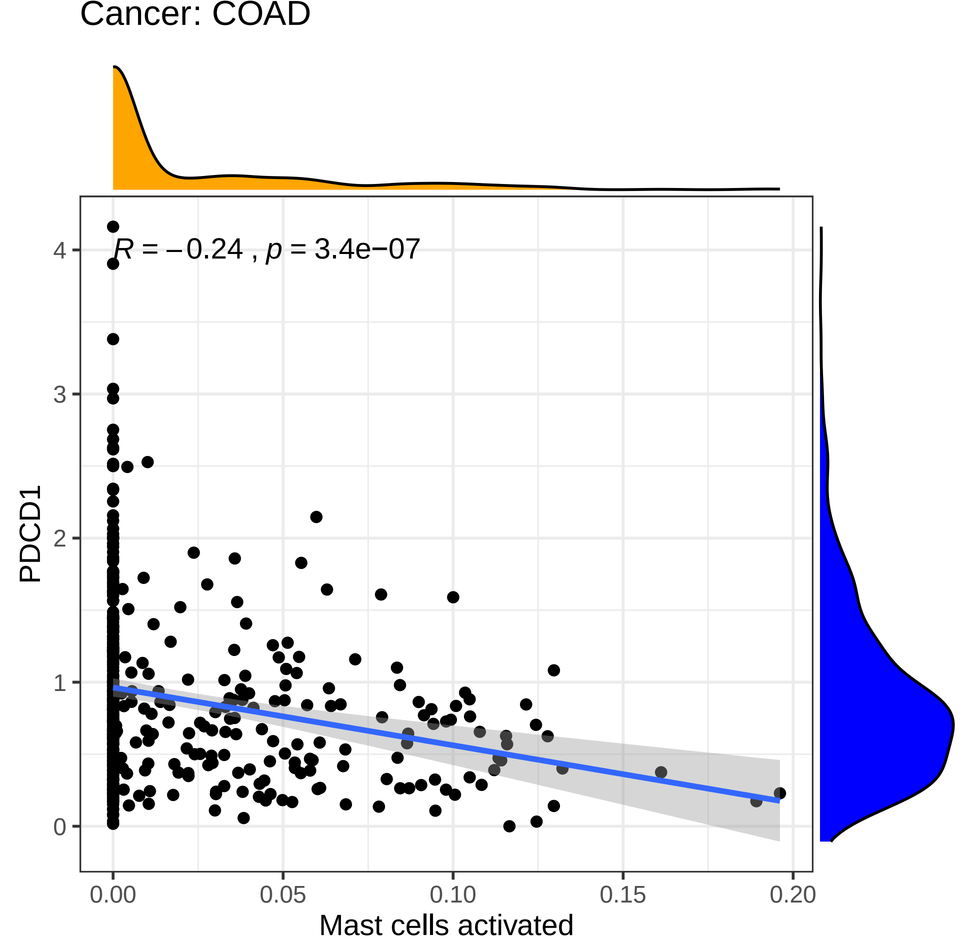

Cancer: COAD
R = −0.24 , p = 3.4e−07
PDCD1
0
1
2
3
4
0.00
0.05
0.10
0.15
0.20
Mast cells activated

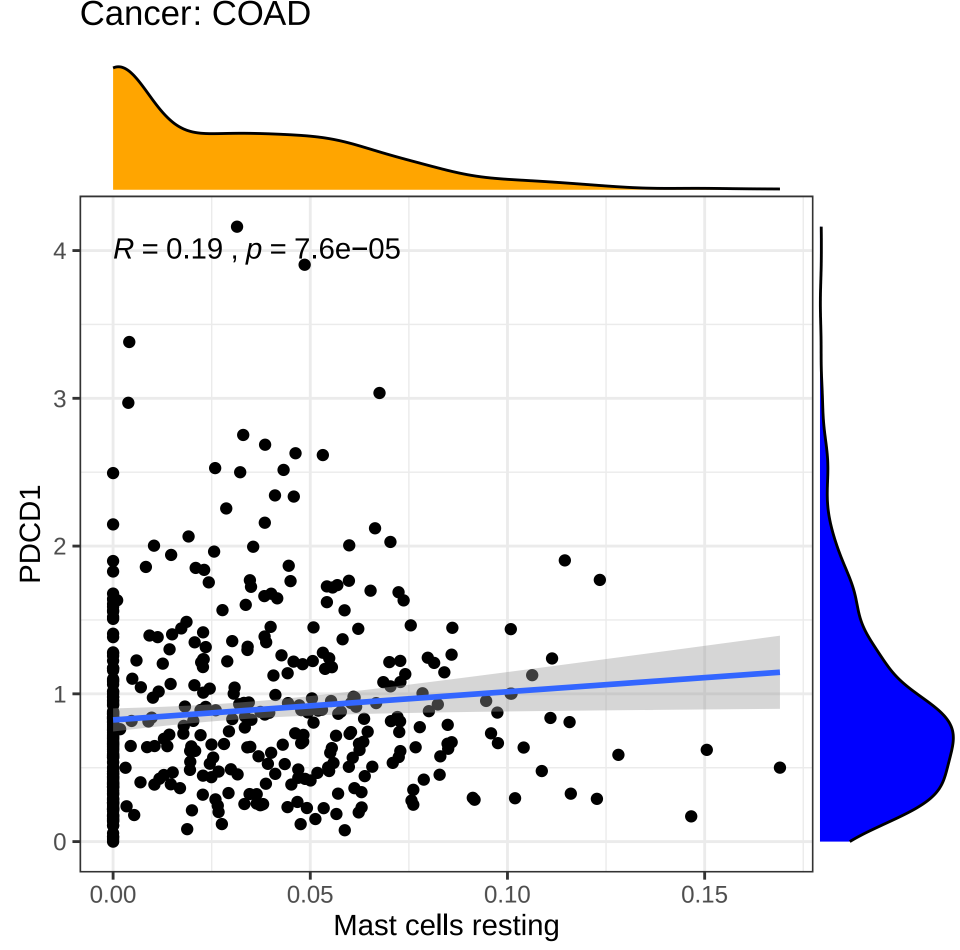

Cancer: COAD
R = 0.19 , p = 7.6e−05
PDCD1
0
1
2
3
4
0.00
0.05
0.10
0.15
Mast cells resting

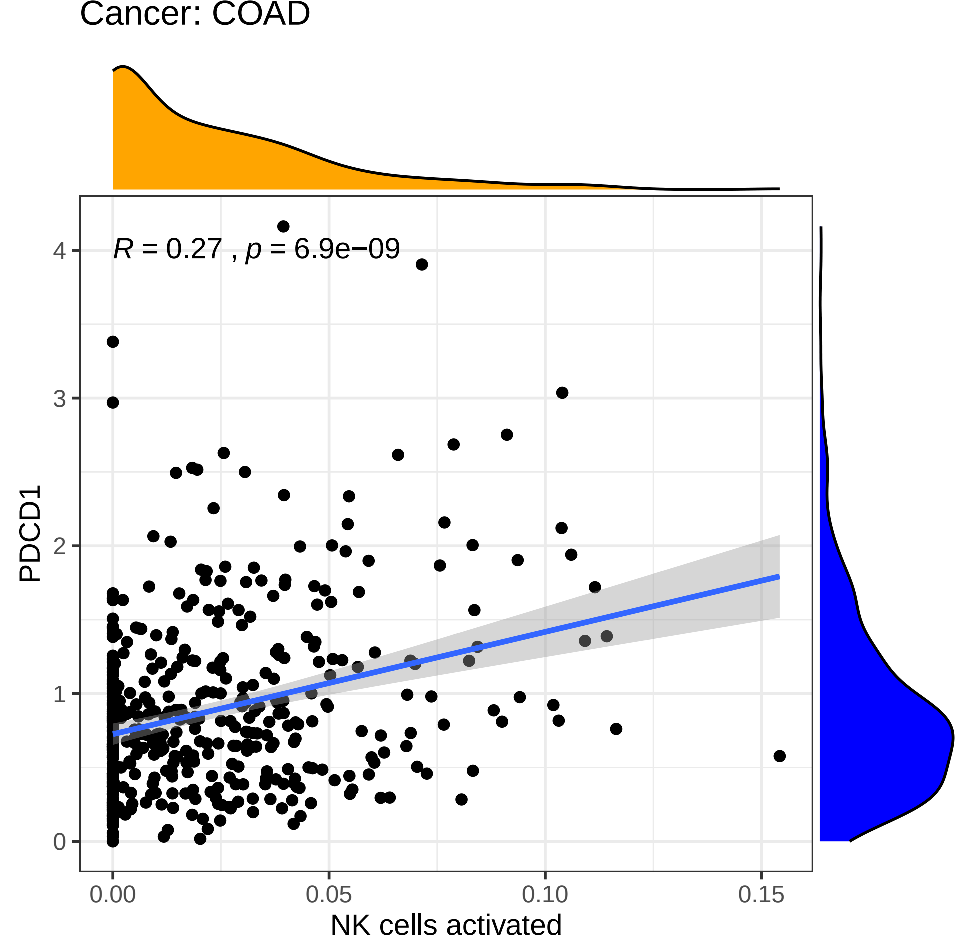

Cancer: COAD
R = 0.27 , p = 6.9e−09
PDCD1
0
1
2
3
4
0.00
0.05
0.10
0.15
NK cells activated

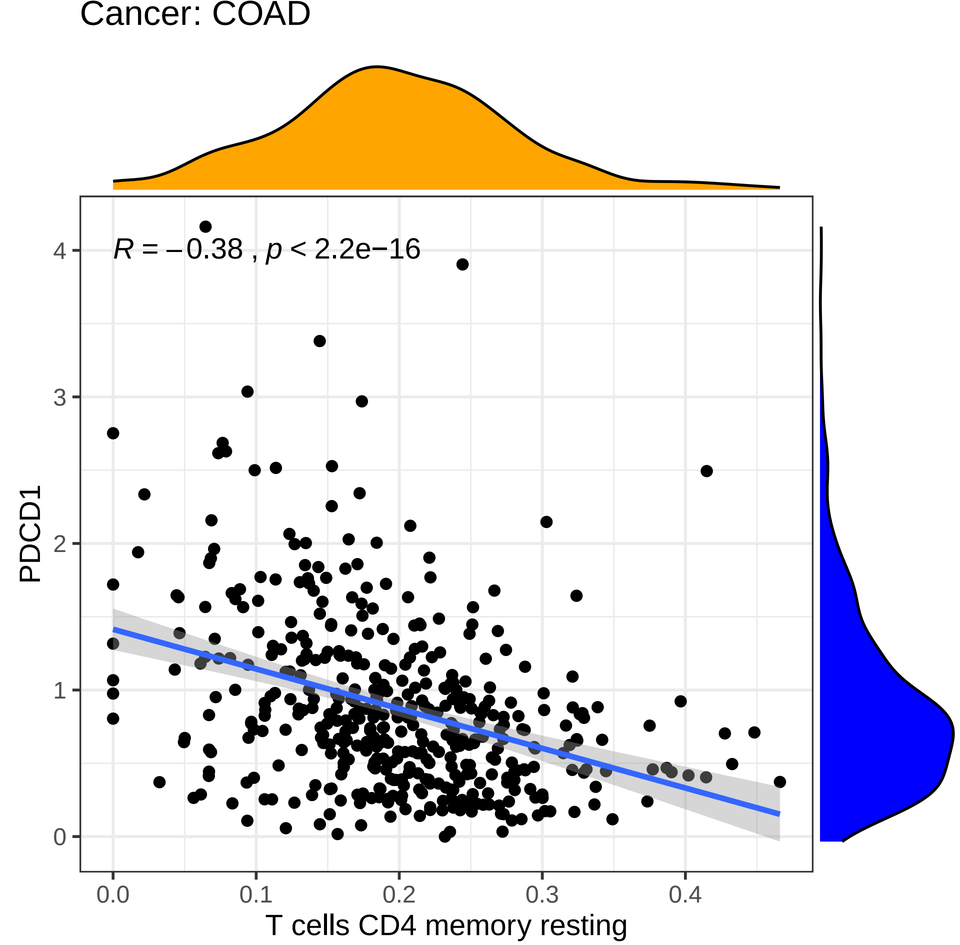
Cancer: COAD
R = −0.38 , p < 2.2e−16
PDCD1
T cells CD4 memory resting
0
1
2
3
4
0.0
0.1
0.2
0.3
0.4

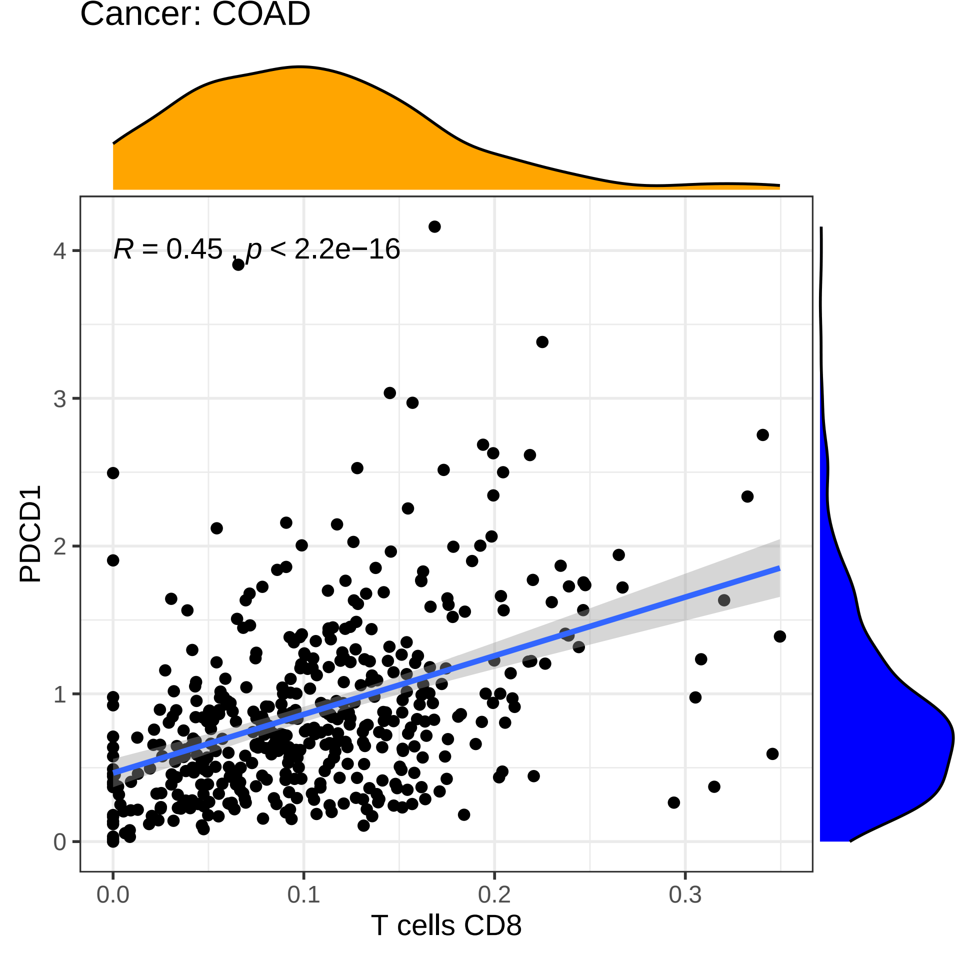

Cancer: COAD
R = 0.45 , p < 2.2e−16
PDCD1
T cells CD8
0
1
2
3
4
0.0
0.1
0.2
0.3

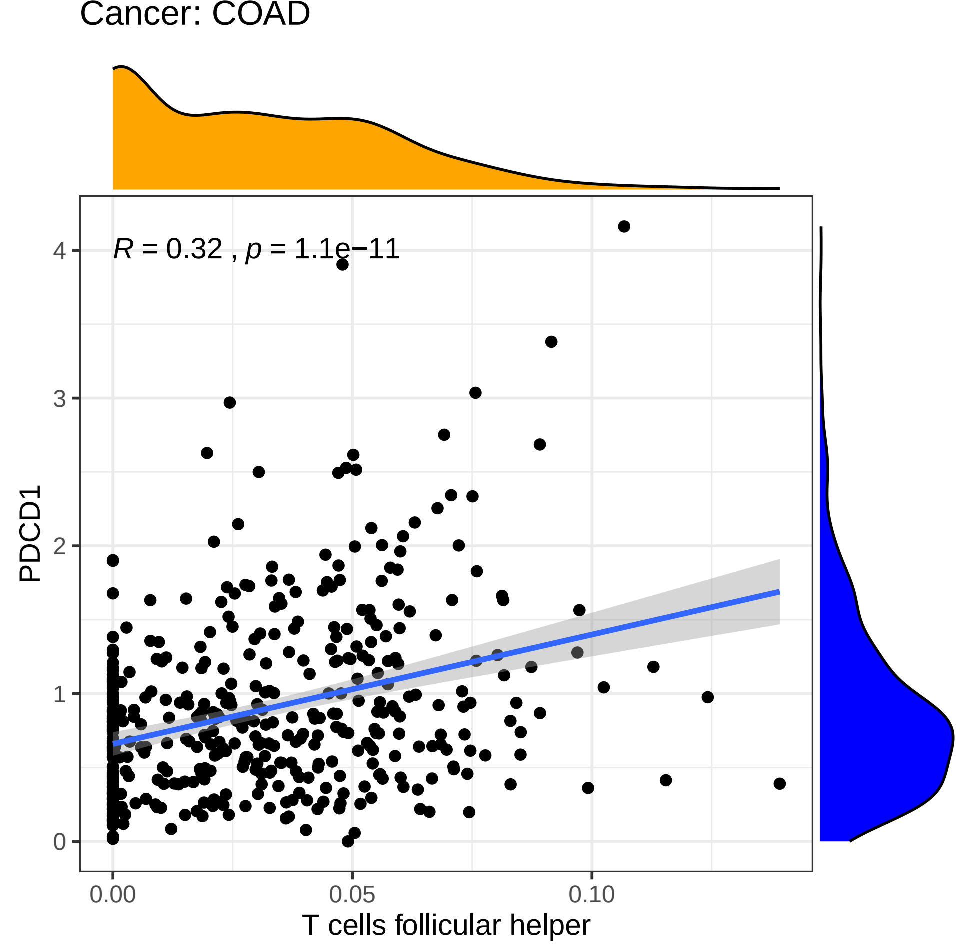
Cancer: COAD
R = 0.32 , p = 1.1e−11
PDCD1
T cells follicular helper
0
1
2
3
4
0.00
0.05
0.10

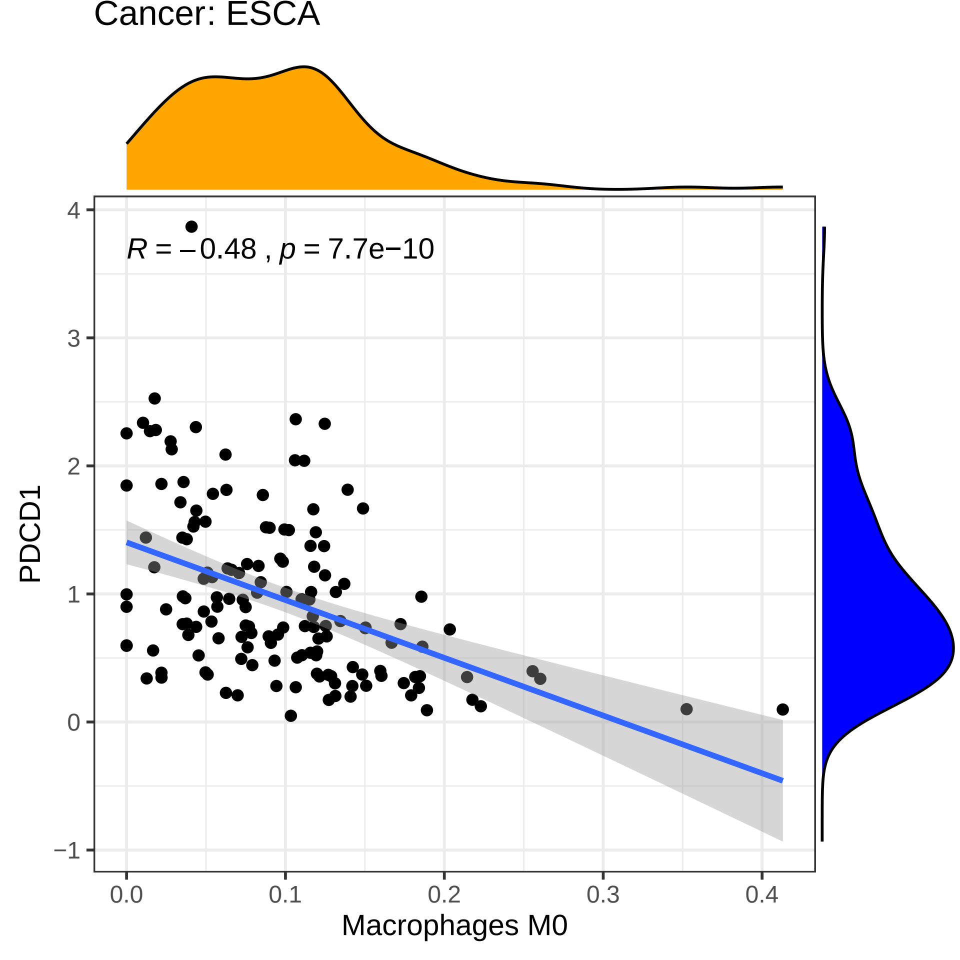

Cancer: ESCA
R = −0.48 , p = 7.7e−10
PDCD1
4
3
2
1
0
−1
0.0
0.1
0.2
0.3
0.4
Macrophages M0

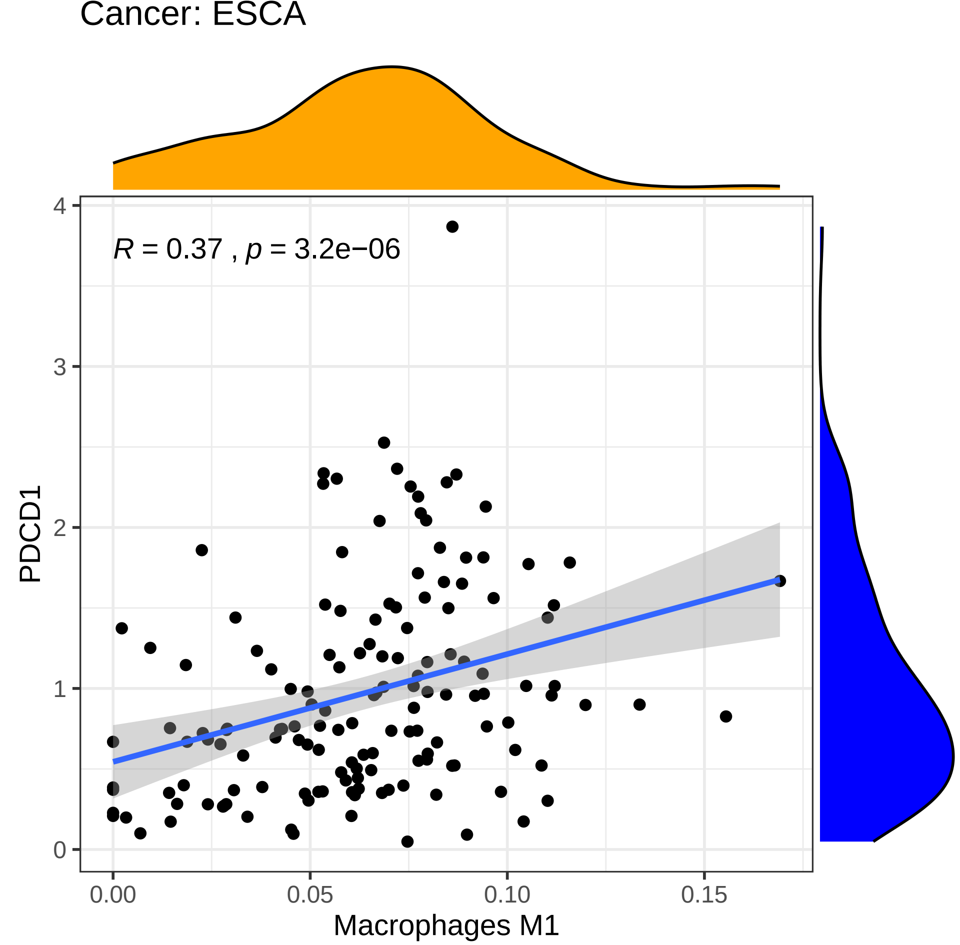

Cancer: ESCA
R = 0.37 , p = 3.2e−06
PDCD1
Macrophages M1
0.00
0.05
0.10
0.15
0
1
2
3
4

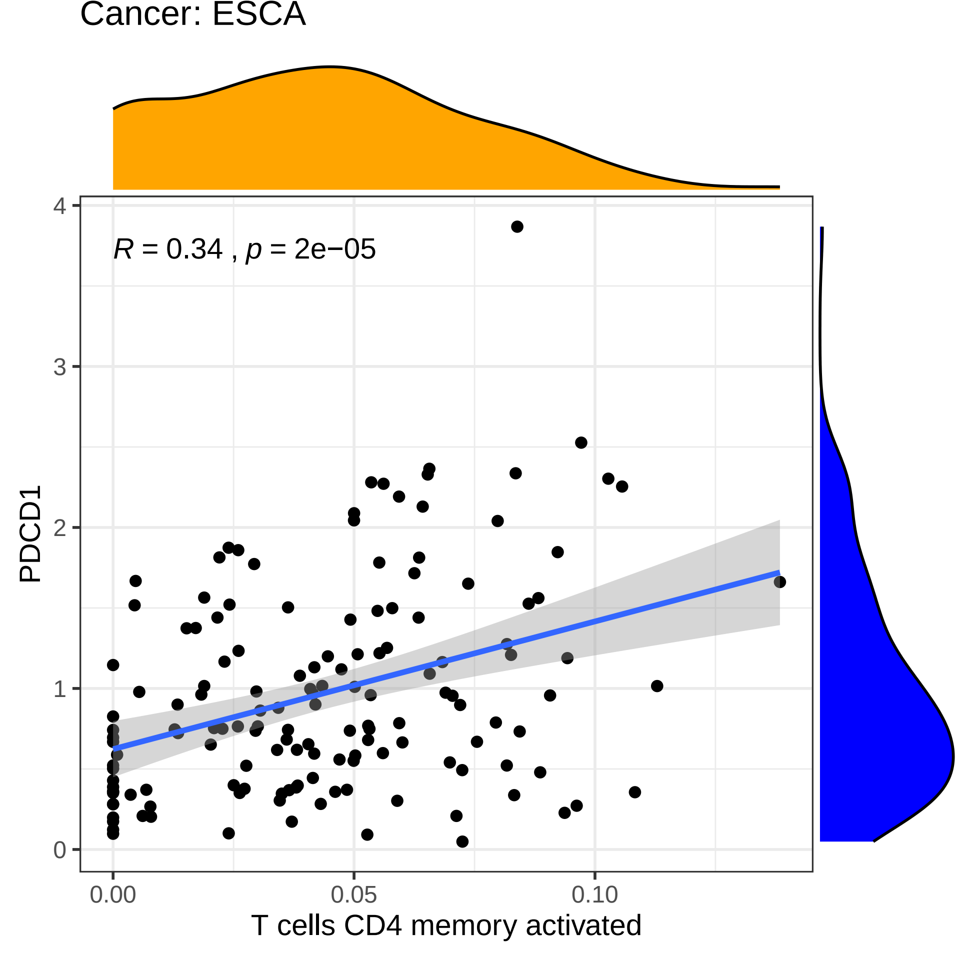

Cancer: ESCA
R = 0.34 , p = 2e−05
4
3
2
1
0
PDCD1
0.00
0.05
0.10
T cells CD4 memory activated

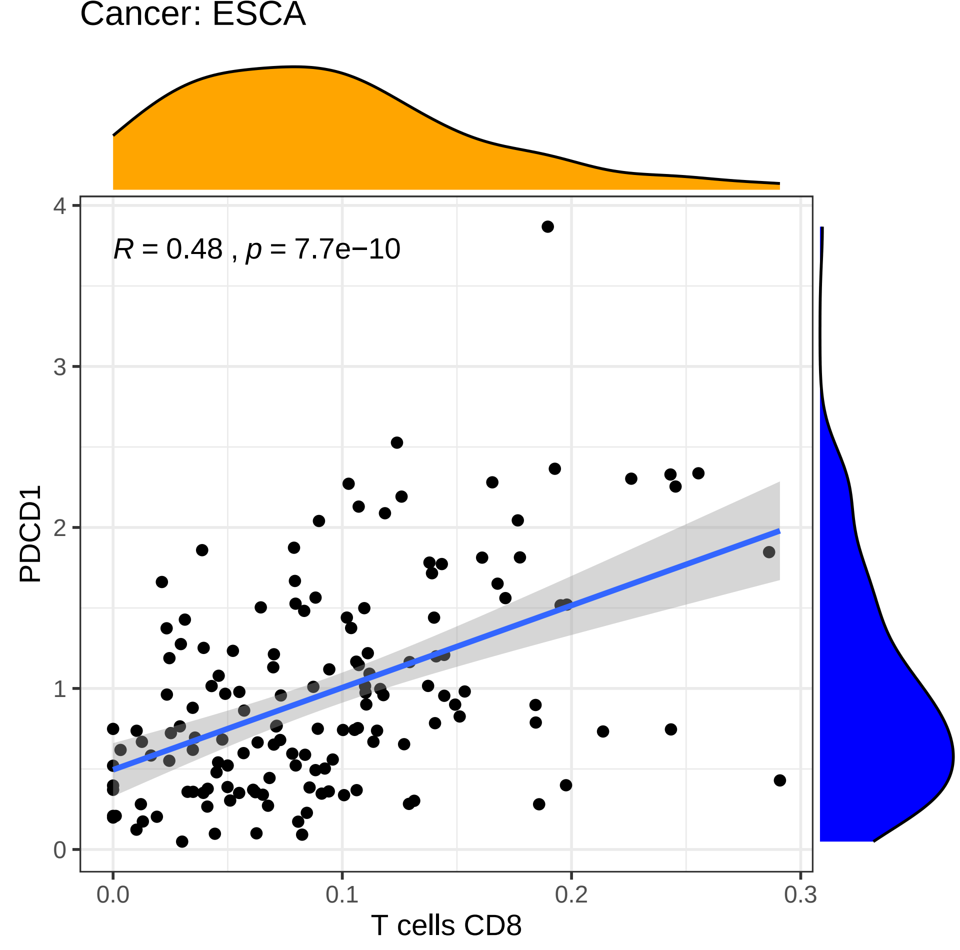
Cancer: ESCA
R = 0.48 , p = 7.7e−10
PDCD1
T cells CD8
0
1
2
3
4
0.0
0.1
0.2
0.3

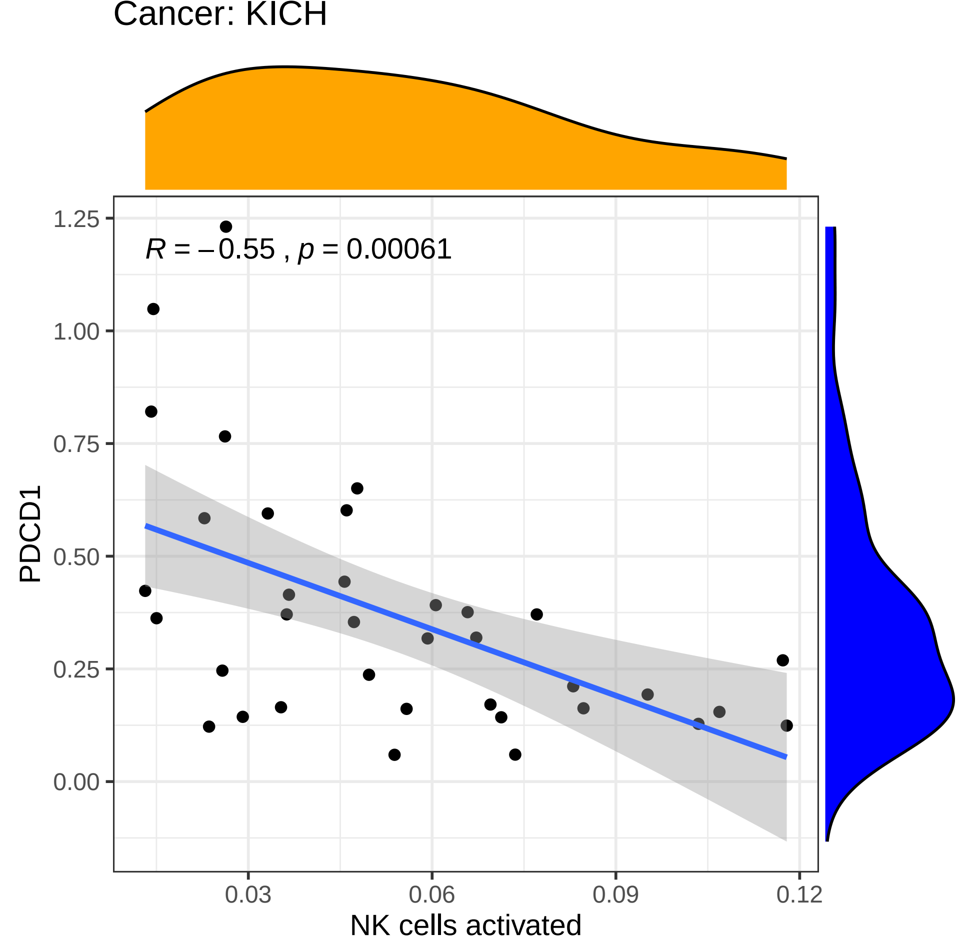

Cancer: KICH
R = −0.55 , p = 0.00061
1.25
1.00
0.75
0.50
0.25
0.00
PDCD1
0.03
0.06
0.09
0.12
NK cells activated

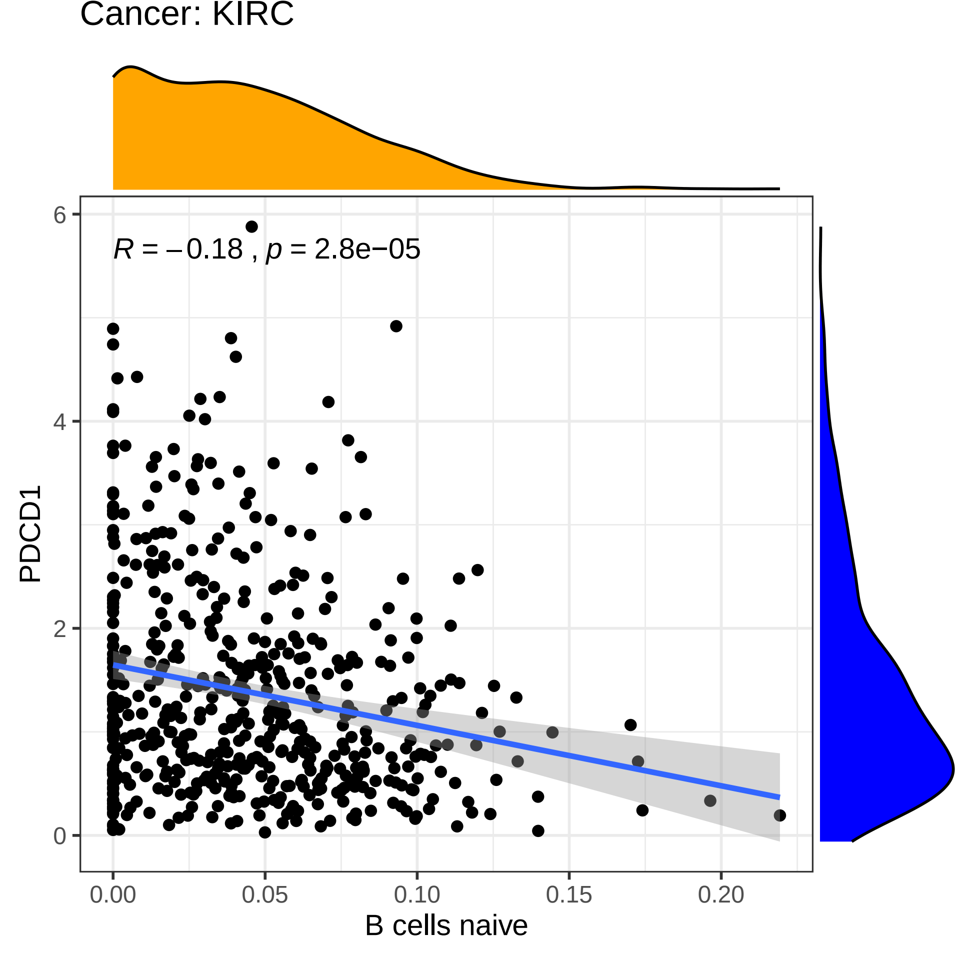
Cancer: KIRC
R = −0.18 , p = 2.8e−05
PDCD1
B cells naive
0
2
4
6
0.00
0.05
0.10
0.15
0.20

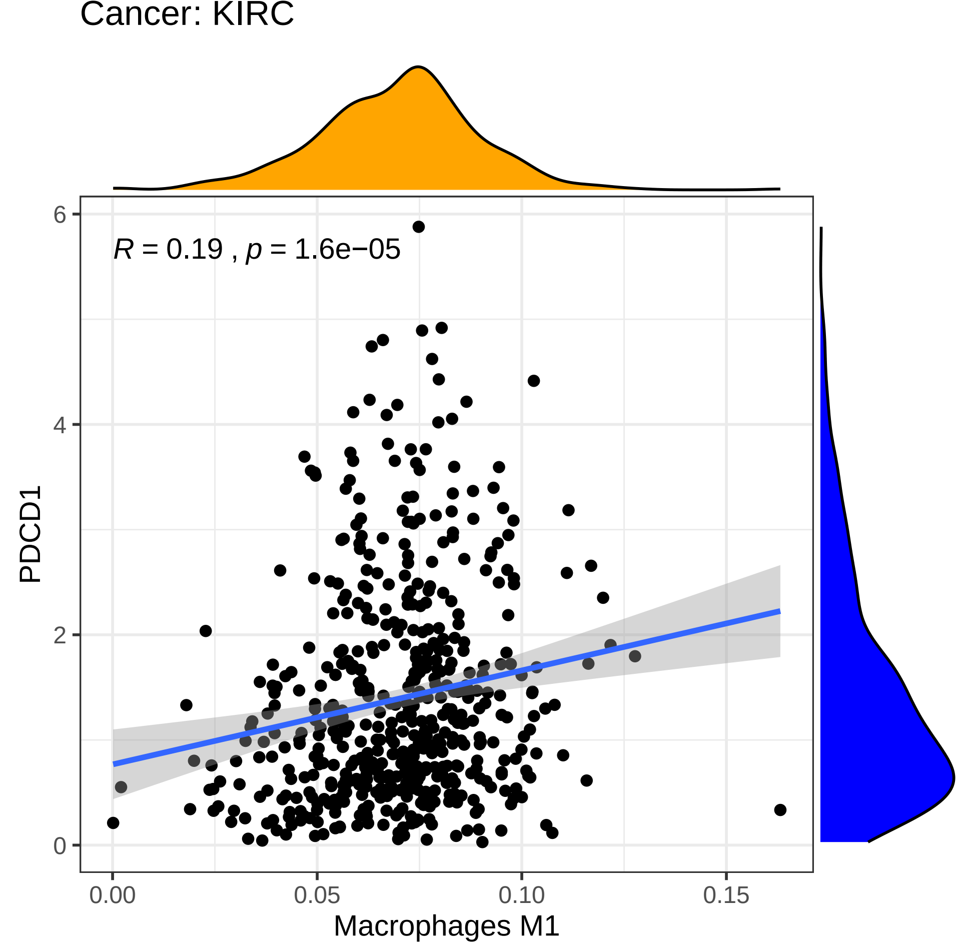
Cancer: KIRC
R = 0.19 , p = 1.6e−05
PDCD1
Macrophages M1
0
2
4
6
0.00
0.05
0.10
0.15

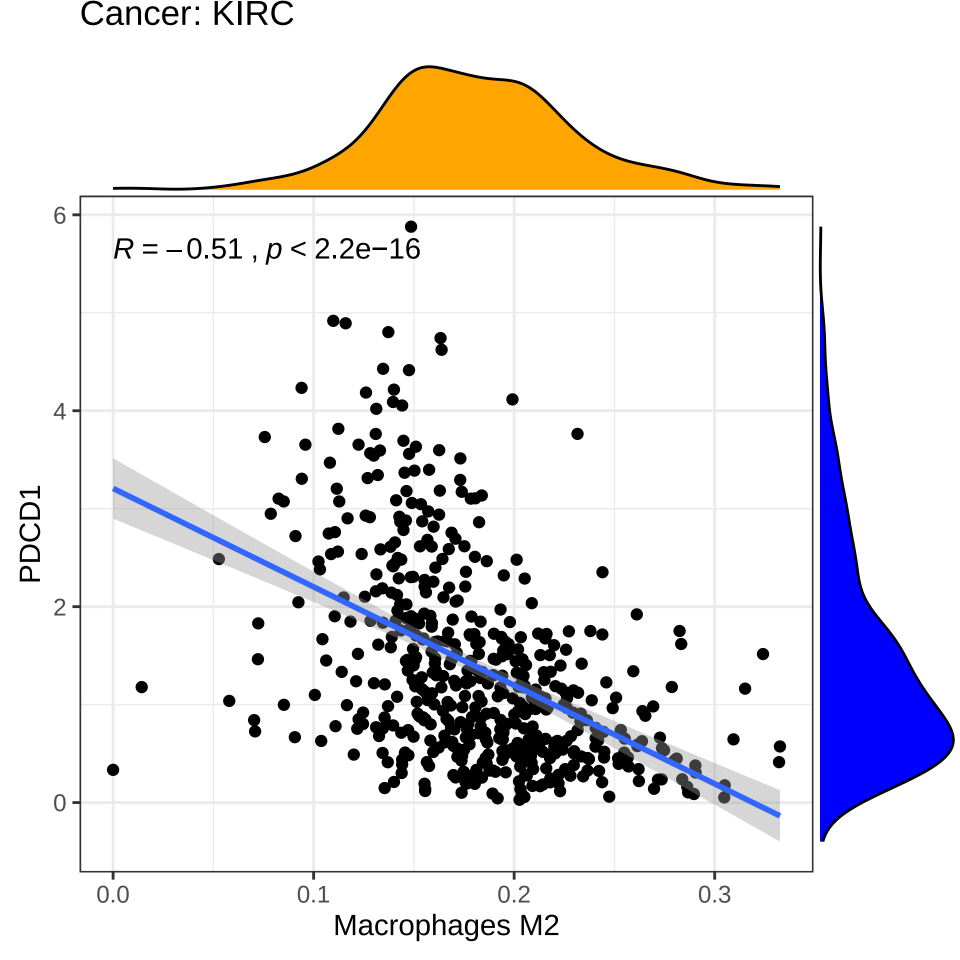

Cancer: KIRC
R = −0.51 , p < 2.2e−16
PDCD1
0
2
4
6
0.0
0.1
0.2
0.3
Macrophages M2

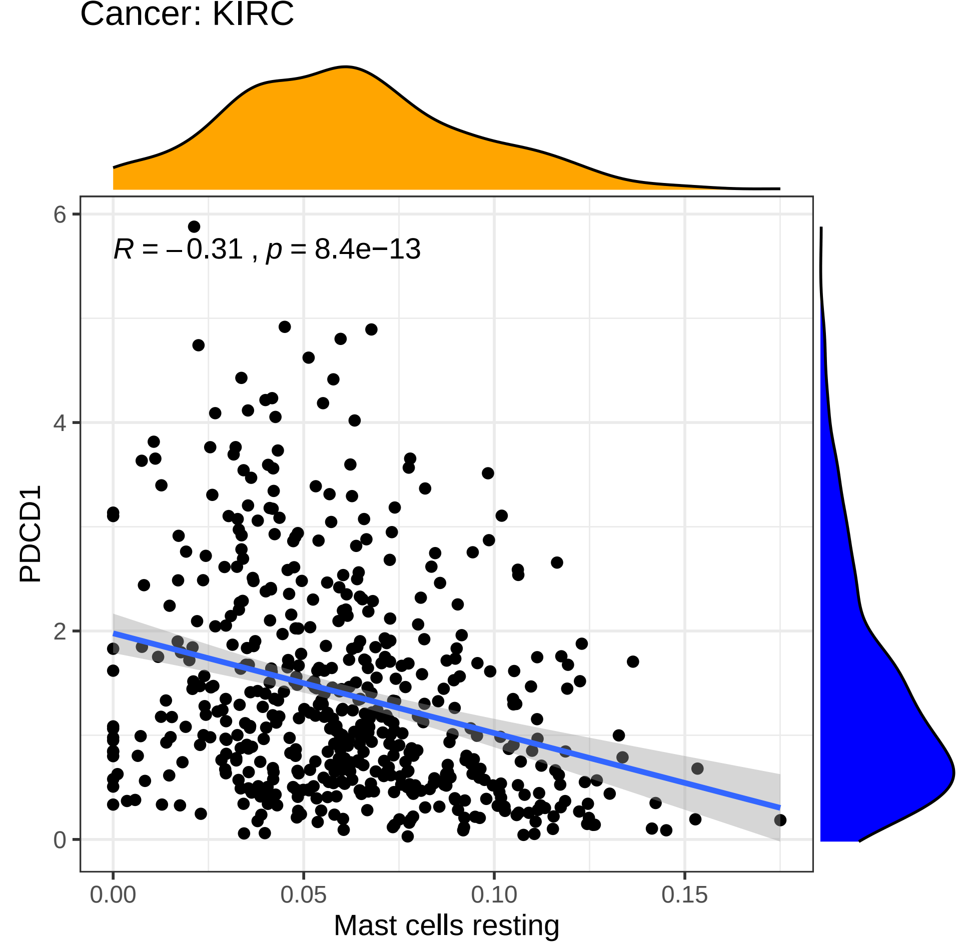

Cancer: KIRC
R = −0.31 , p = 8.4e−13
PDCD1
0
2
4
6
0.00
0.05
0.10
0.15
Mast cells resting

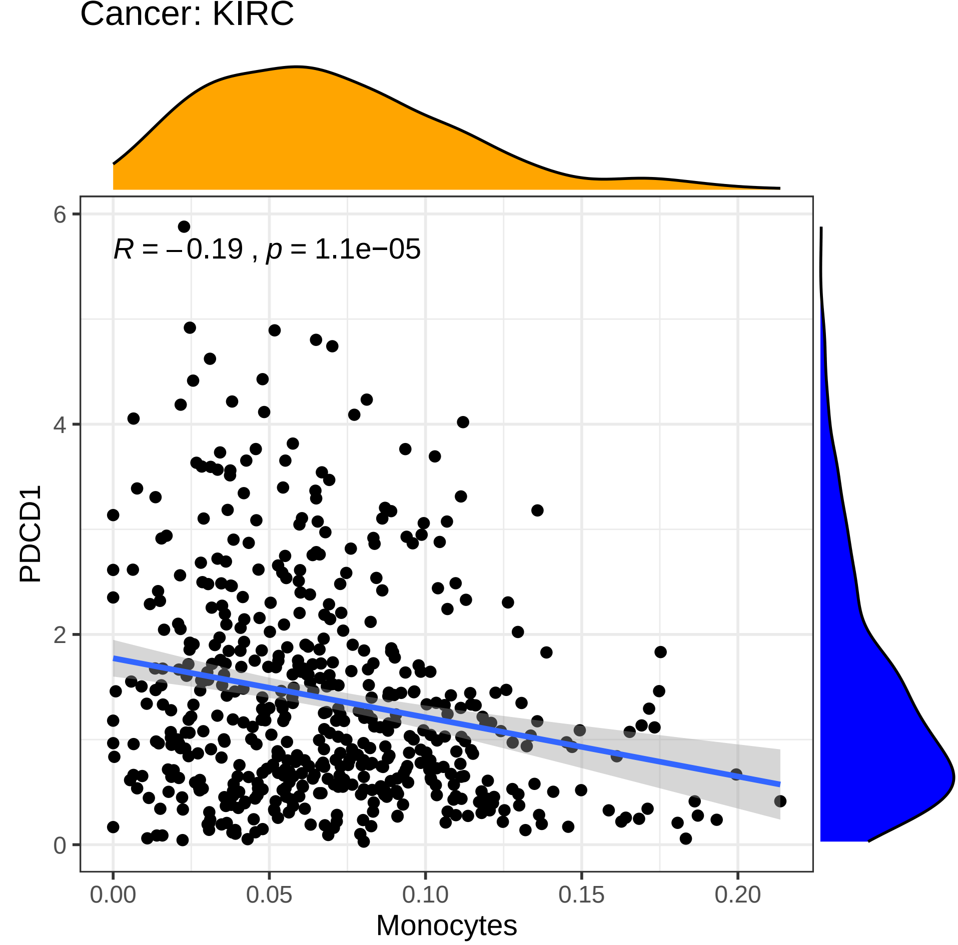

Cancer: KIRC
R = −0.19 , p = 1.1e−05
PDCD1
Monocytes
0
2
4
6
0.00
0.05
0.10
0.15
0.20

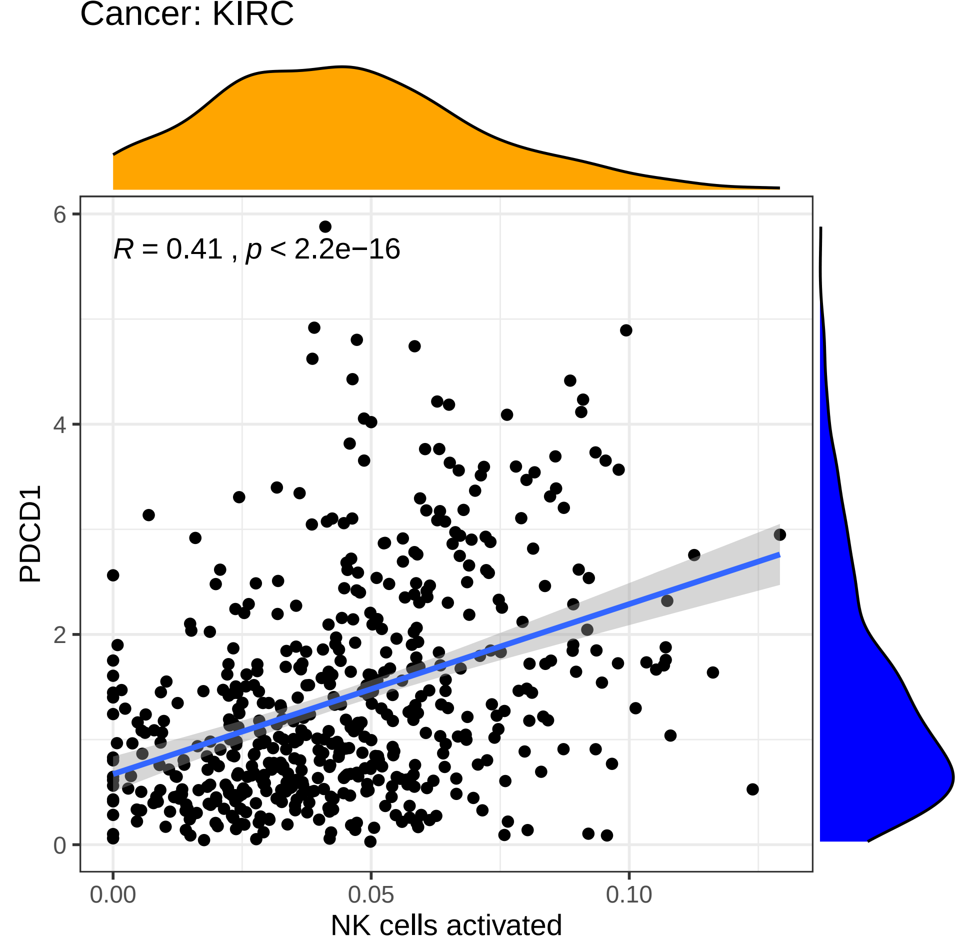
Cancer: KIRC
R = 0.41 , p < 2.2e−16
PDCD1
NK cells activated
0
2
4
6
0.00
0.05
0.10

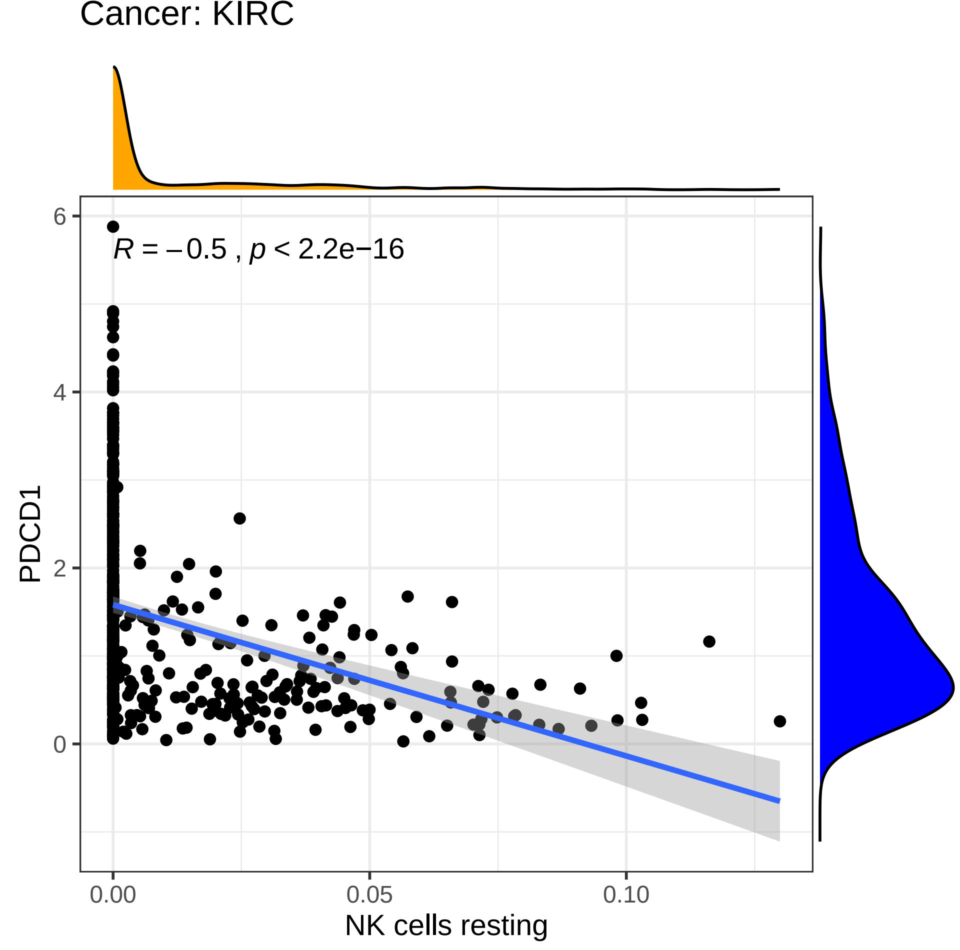

Cancer: KIRC
R = −0.5 , p < 2.2e−16
PDCD1
6
4
2
0
0.00
0.05
0.10
NK cells resting

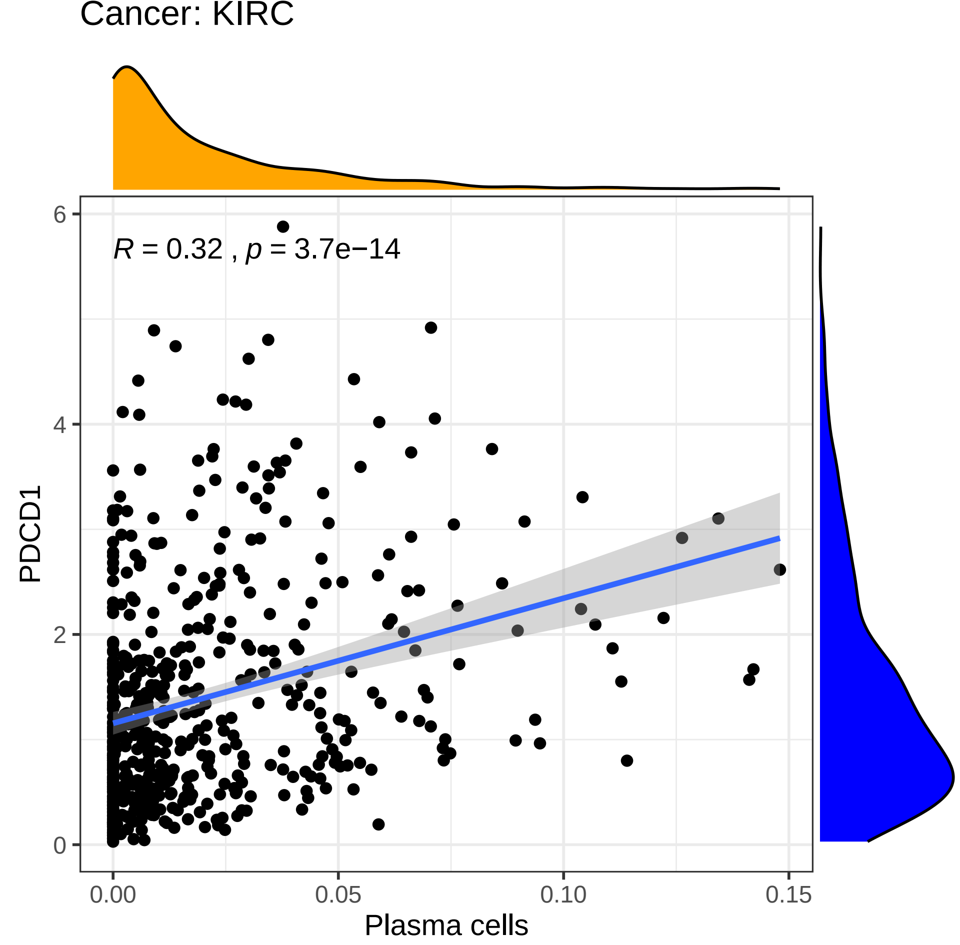

Cancer: KIRC
R = 0.32 , p = 3.7e−14
PDCD1
Plasma cells
0
2
4
6
0.00
0.05
0.10
0.15

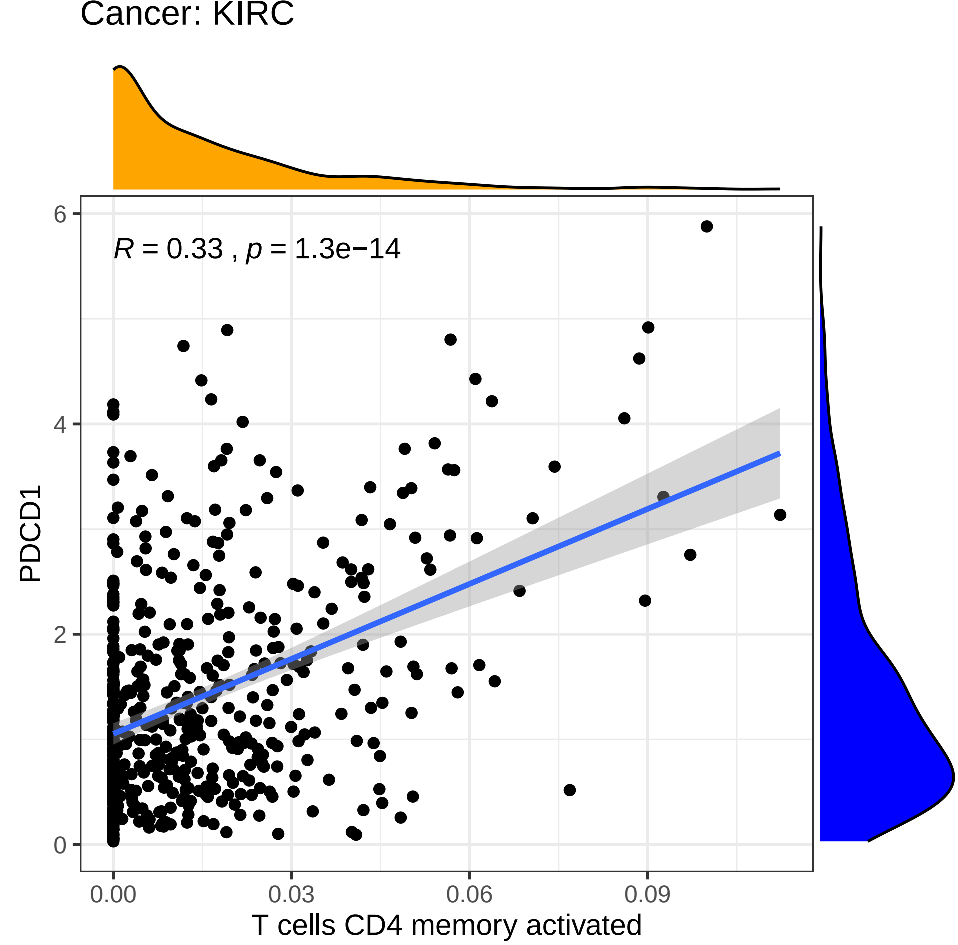

Cancer: KIRC
R = 0.33 , p = 1.3e−14
PDCD1
0
2
4
6
0.00
0.03
0.06
0.09
T cells CD4 memory activated

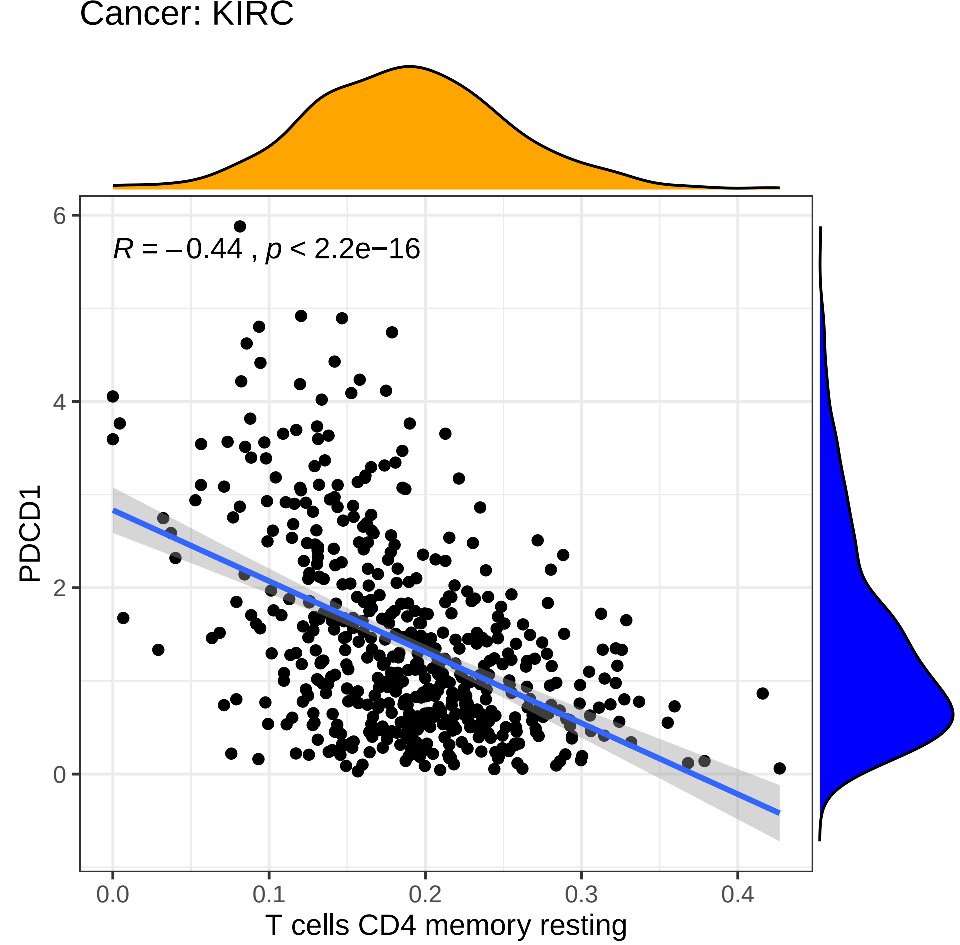

Cancer: KIRC
R = −0.44 , p < 2.2e−16
PDCD1
0
2
4
6
0.0
0.1
0.2
0.3
0.4
T cells CD4 memory resting

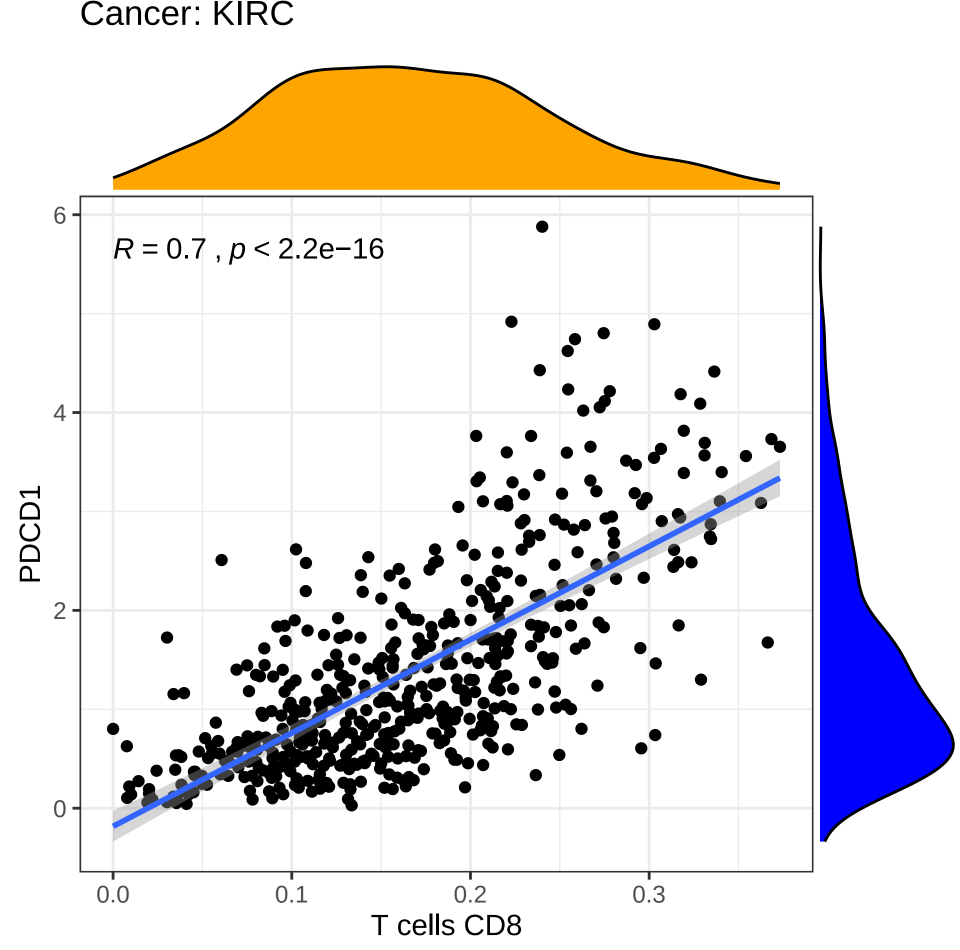
Cancer: KIRC
R = 0.7 , p < 2.2e−16
PDCD1
0
2
4
6
0.0
0.1
0.2
0.3
T cells CD8

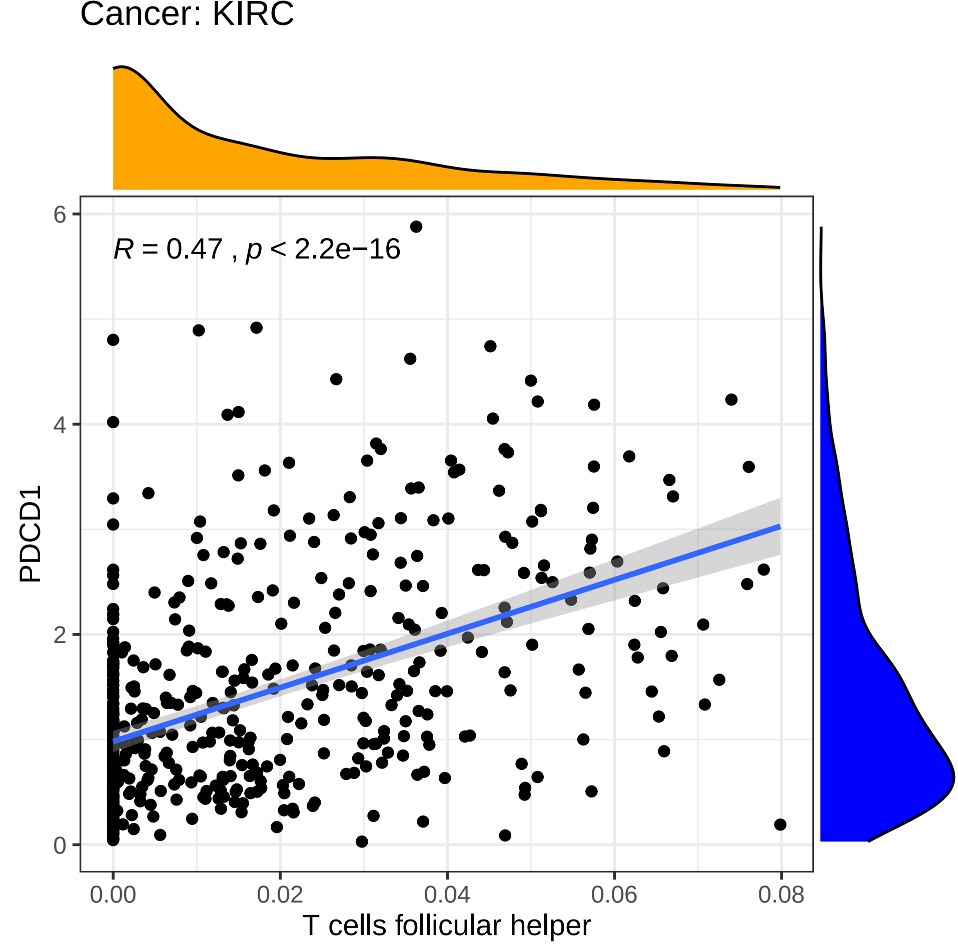

Cancer: KIRC
R = 0.47 , p < 2.2e−16
PDCD1
0
2
4
6
0.00
0.02
0.04
0.06
0.08
T cells follicular helper

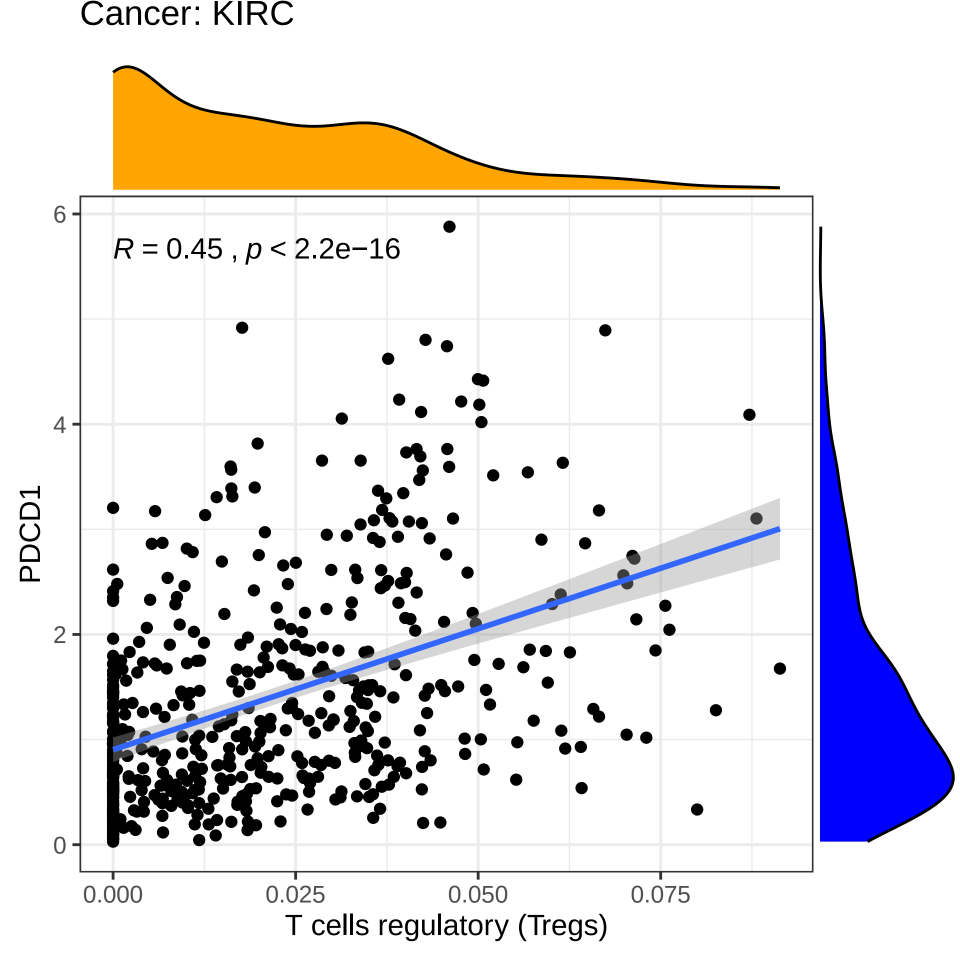

Cancer: KIRC
R = 0.45 , p < 2.2e−16
PDCD1
T cells regulatory (Tregs)
0.000
0.025
0.050
0.075
0
2
4
6

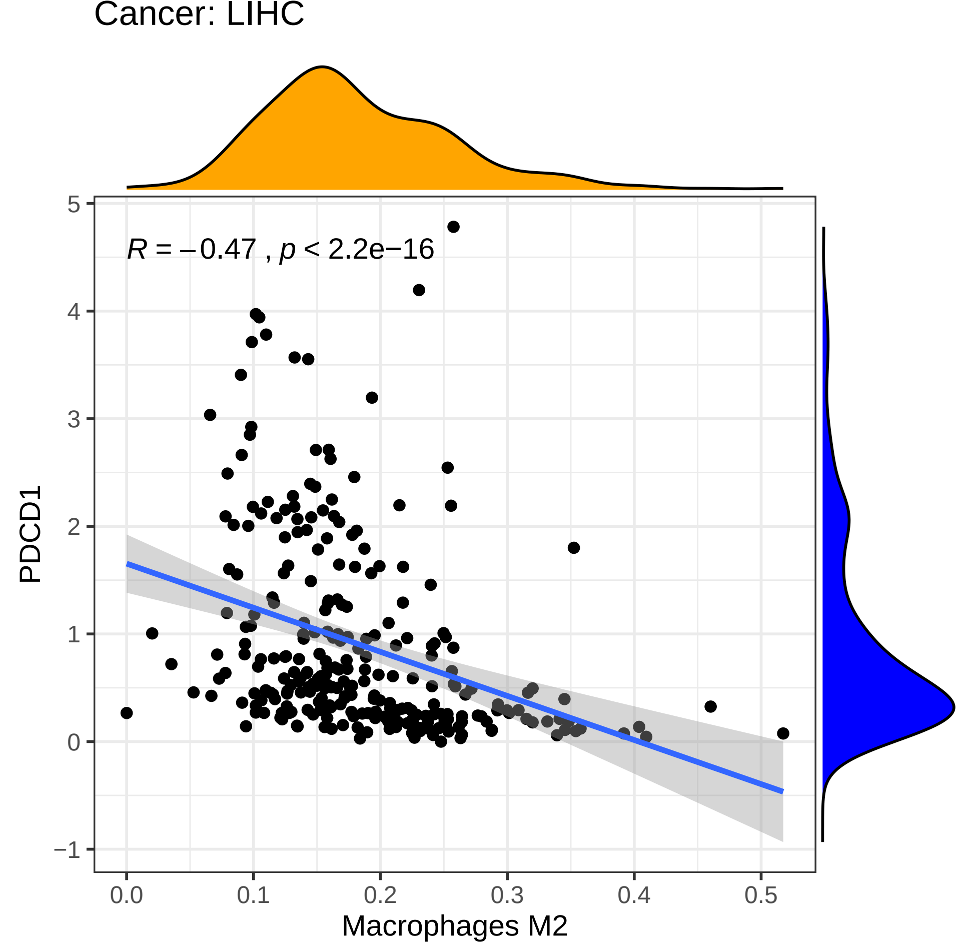

Cancer: LIHC
R = −0.47 , p < 2.2e−16
PDCD1
Macrophages M2
5
4
3
2
1
0
−1
0.0
0.1
0.2
0.3
0.4
0.5

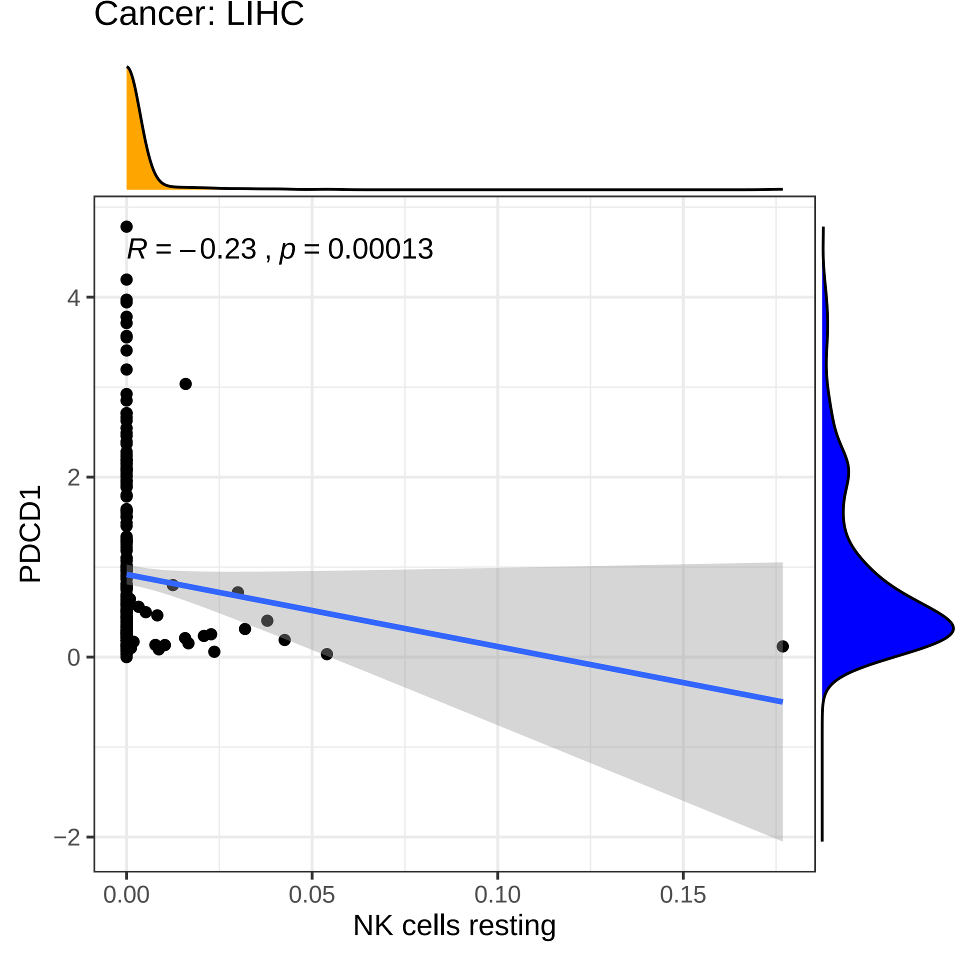

Cancer: LIHC
R = −0.23 , p = 0.00013
PDCD1
4
2
0
−2
0.00
0.05
0.10
0.15
NK cells resting

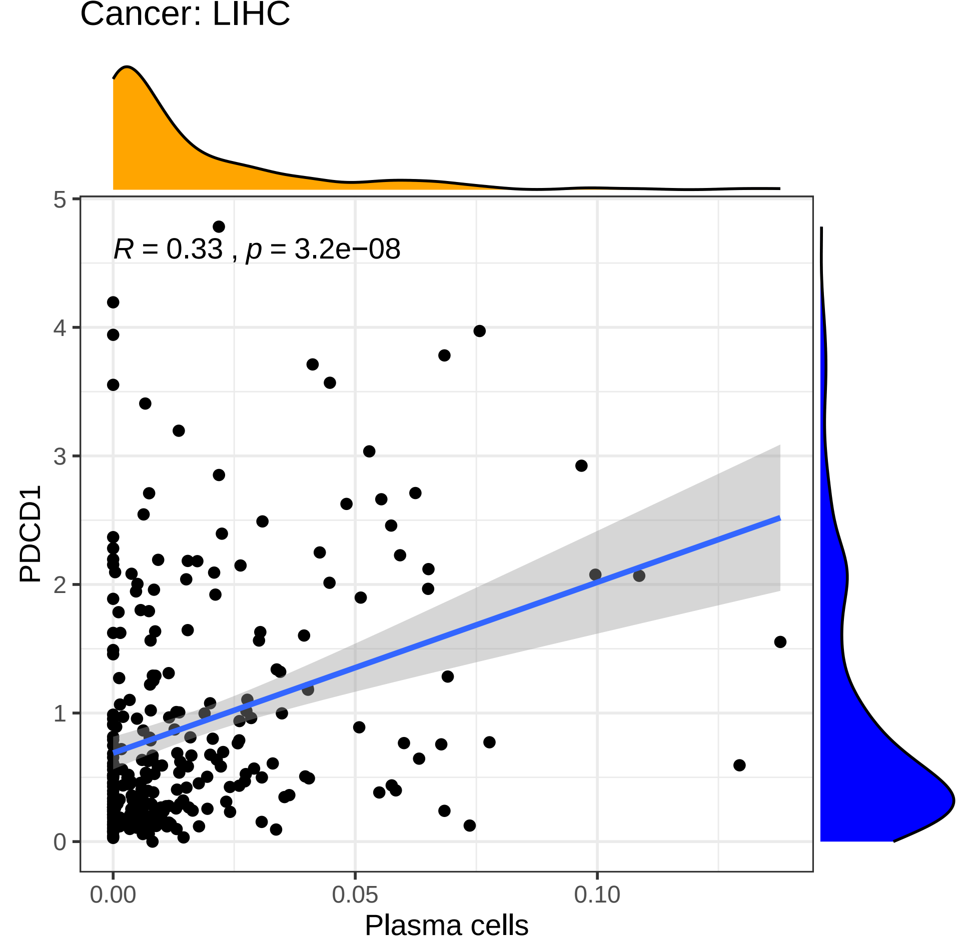

Cancer: LIHC
R = 0.33 , p = 3.2e−08
PDCD1
Plasma cells
0
1
2
3
4
5
0.00
0.05
0.10

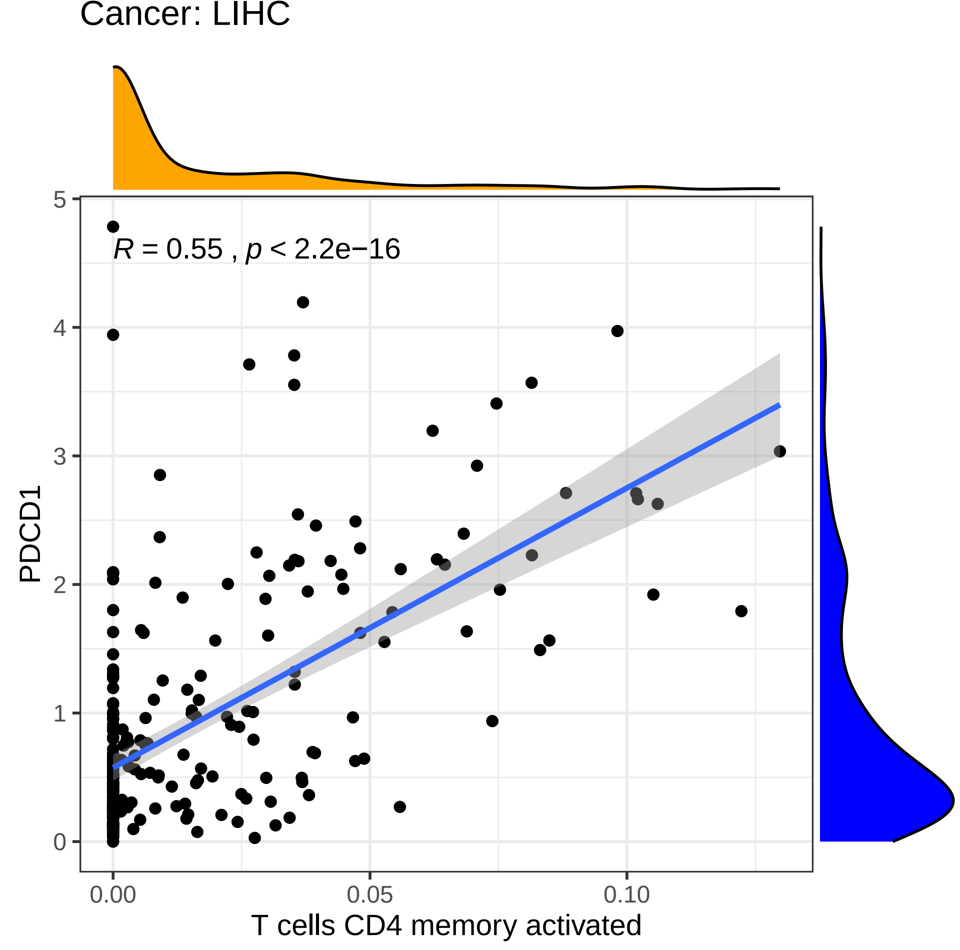

Cancer: LIHC
R = 0.55 , p < 2.2e−16
PDCD1
T cells CD4 memory activated
0
1
2
3
4
5
0.00
0.05
0.10

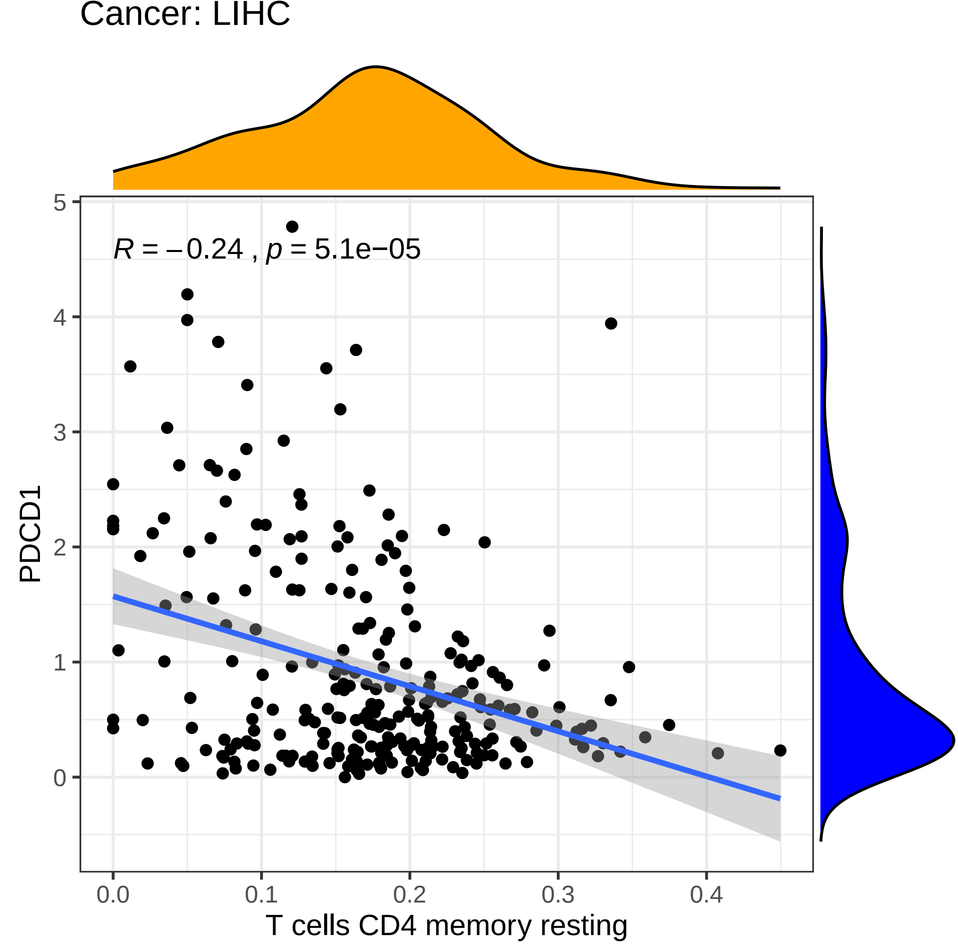

Cancer: LIHC
R = −0.24 , p = 5.1e−05
PDCD1
T cells CD4 memory resting
0
1
2
3
4
5
0.0
0.1
0.2
0.3
0.4

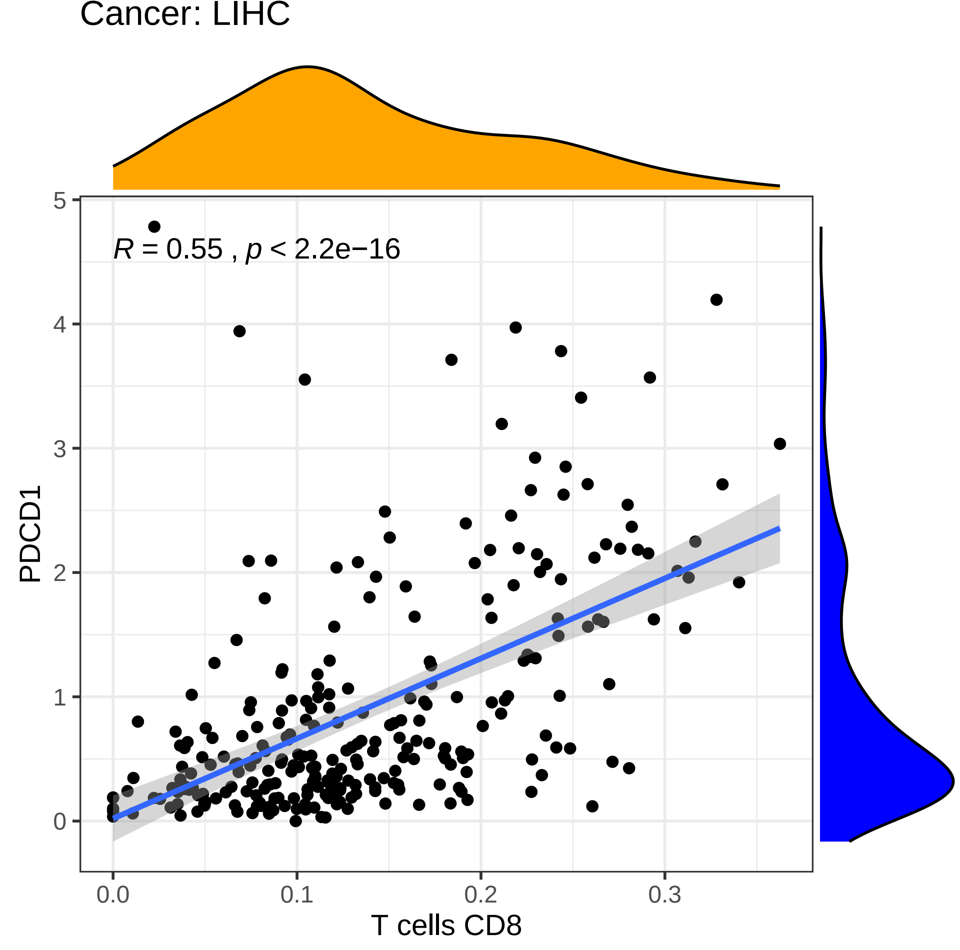

Cancer: LIHC
R = 0.55 , p < 2.2e−16
PDCD1
T cells CD8
0
1
2
3
4
5
0.0
0.1
0.2
0.3

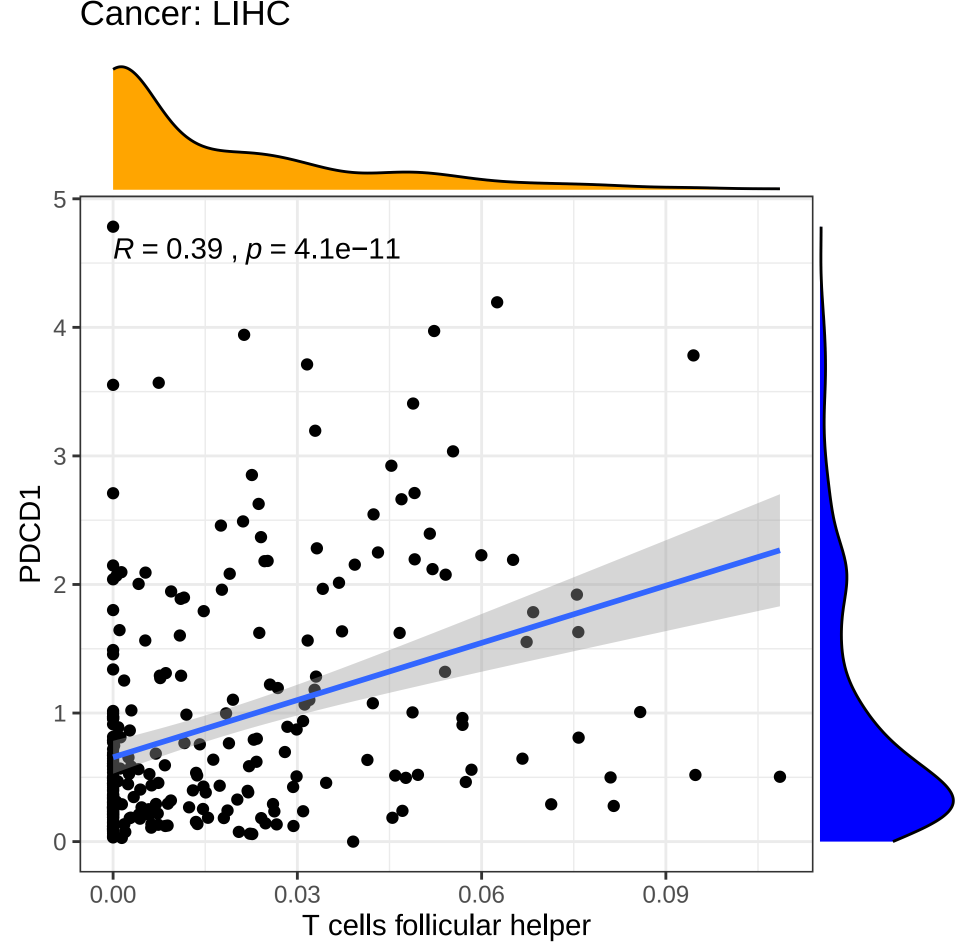

Cancer: LIHC
R = 0.39 , p = 4.1e−11
PDCD1
T cells follicular helper
0
1
2
3
4
5
0.00
0.03
0.06
0.09

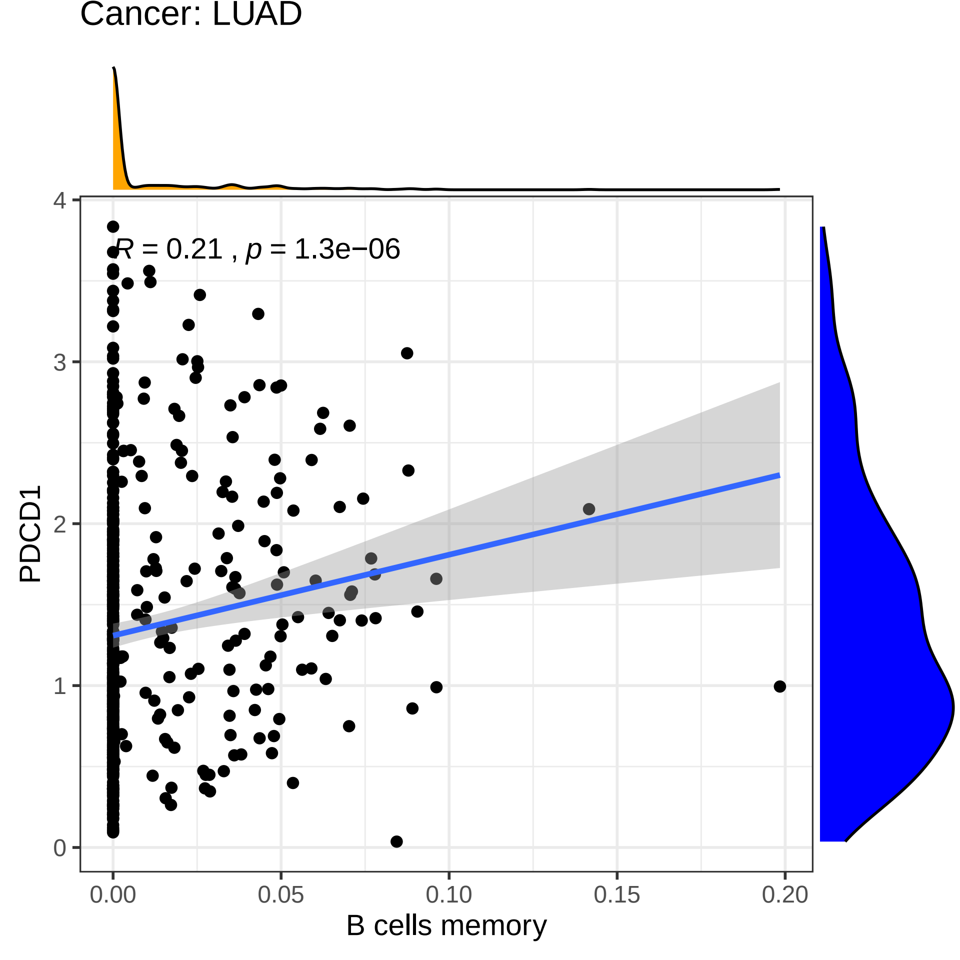

Cancer: LUAD
R = 0.21 , p = 1.3e−06
PDCD1
B cells memory
0
1
2
3
4
0.00
0.05
0.10
0.15
0.20

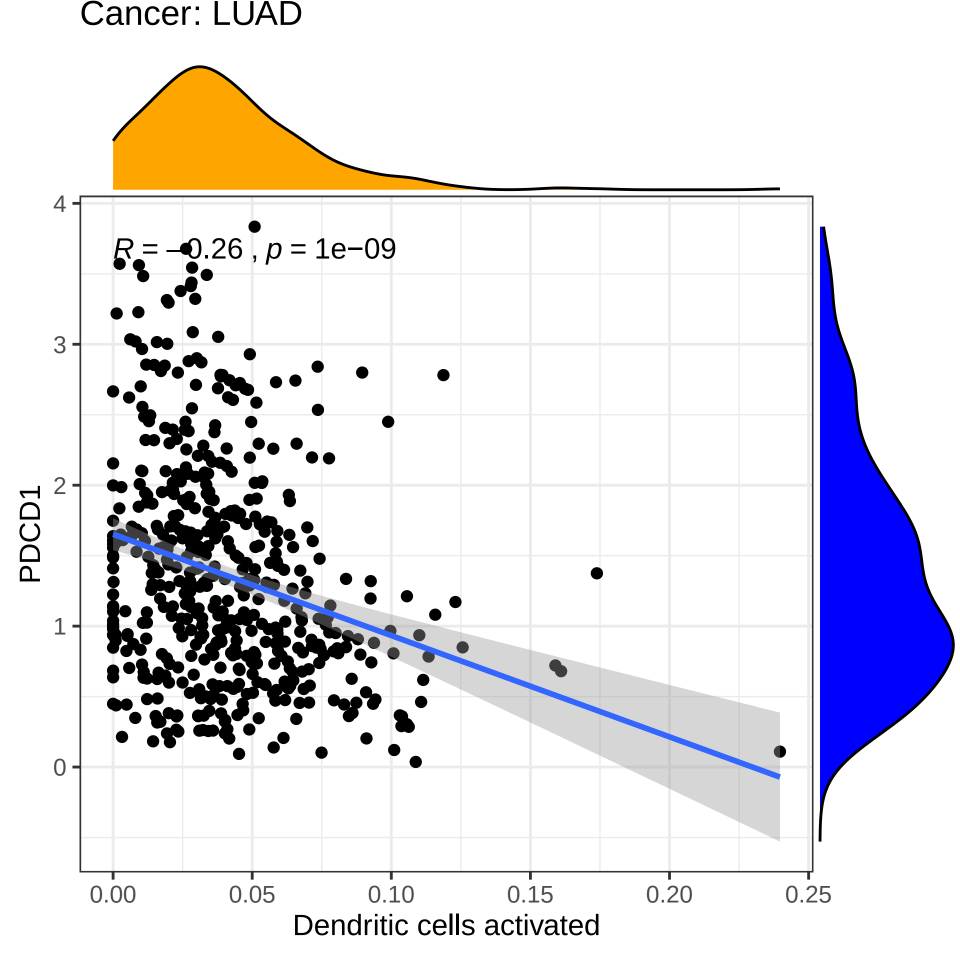

Cancer: LUAD
R = −0.26 , p = 1e−09
4
3
2
1
0
PDCD1
0.00
0.05
0.10
0.15
0.20
0.25
Dendritic cells activated

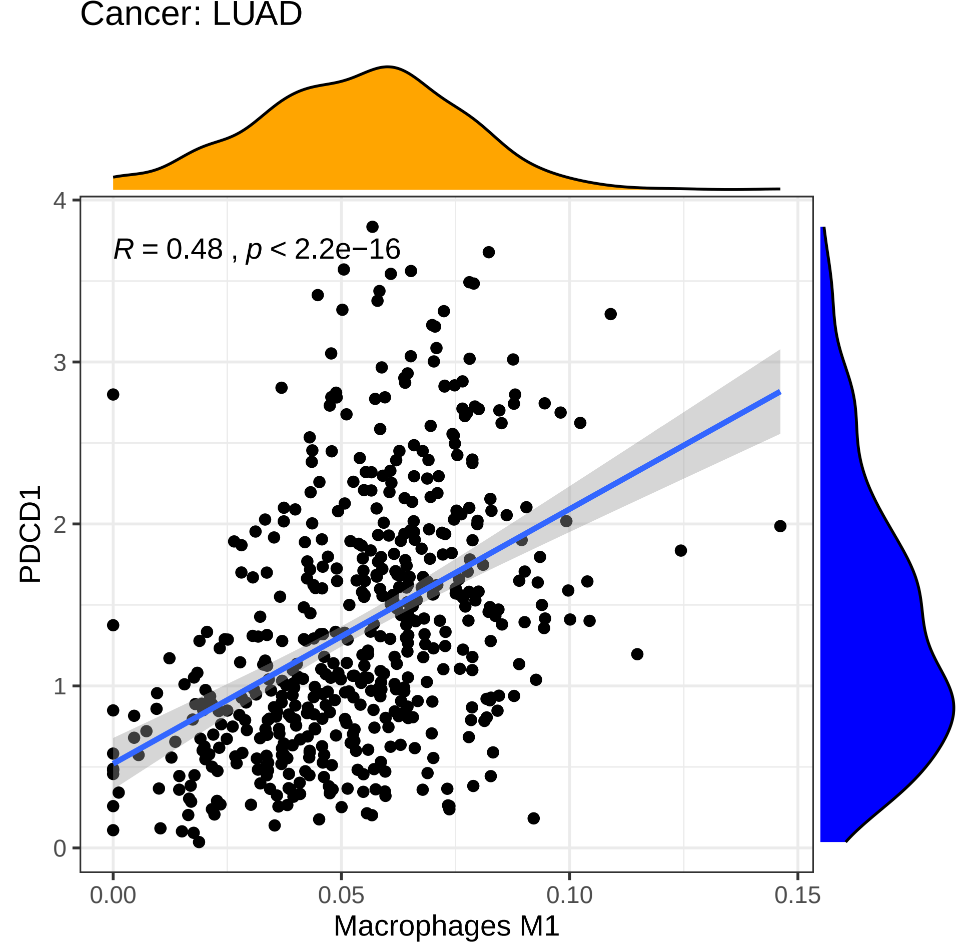

Cancer: LUAD
R = 0.48 , p < 2.2e−16
PDCD1
0
1
2
3
4
0.00
0.05
0.10
0.15
Macrophages M1

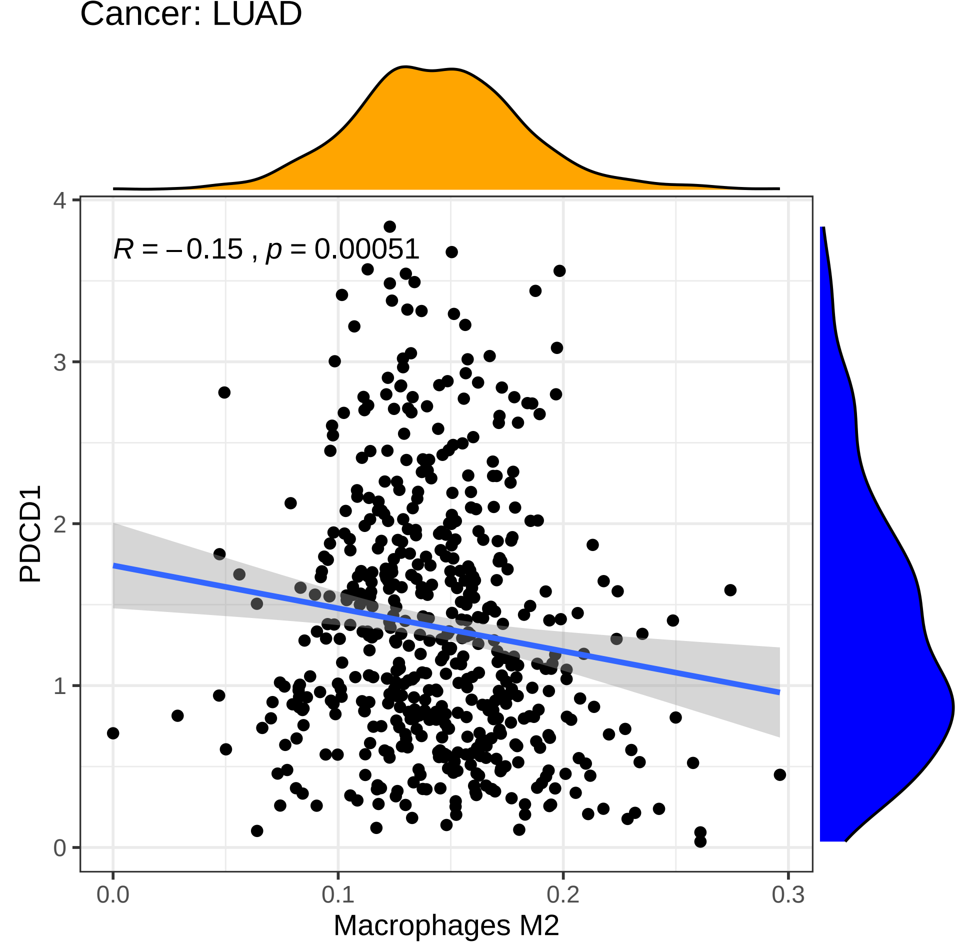

Cancer: LUAD
R = −0.15 , p = 0.00051
PDCD1
Macrophages M2
0
1
2
3
4
0.0
0.1
0.2
0.3

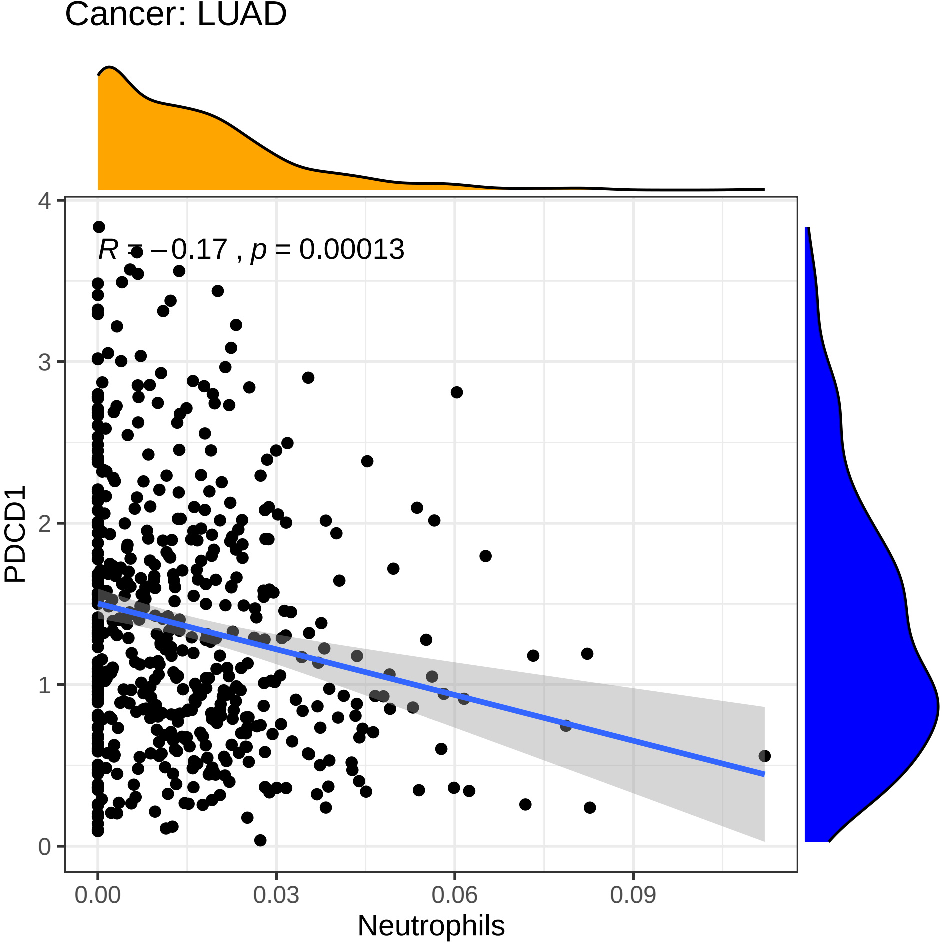

Cancer: LUAD
R = −0.17 , p = 0.00013
PDCD1
4
3
2
1
0
0.00
0.03
0.06
0.09
Neutrophils

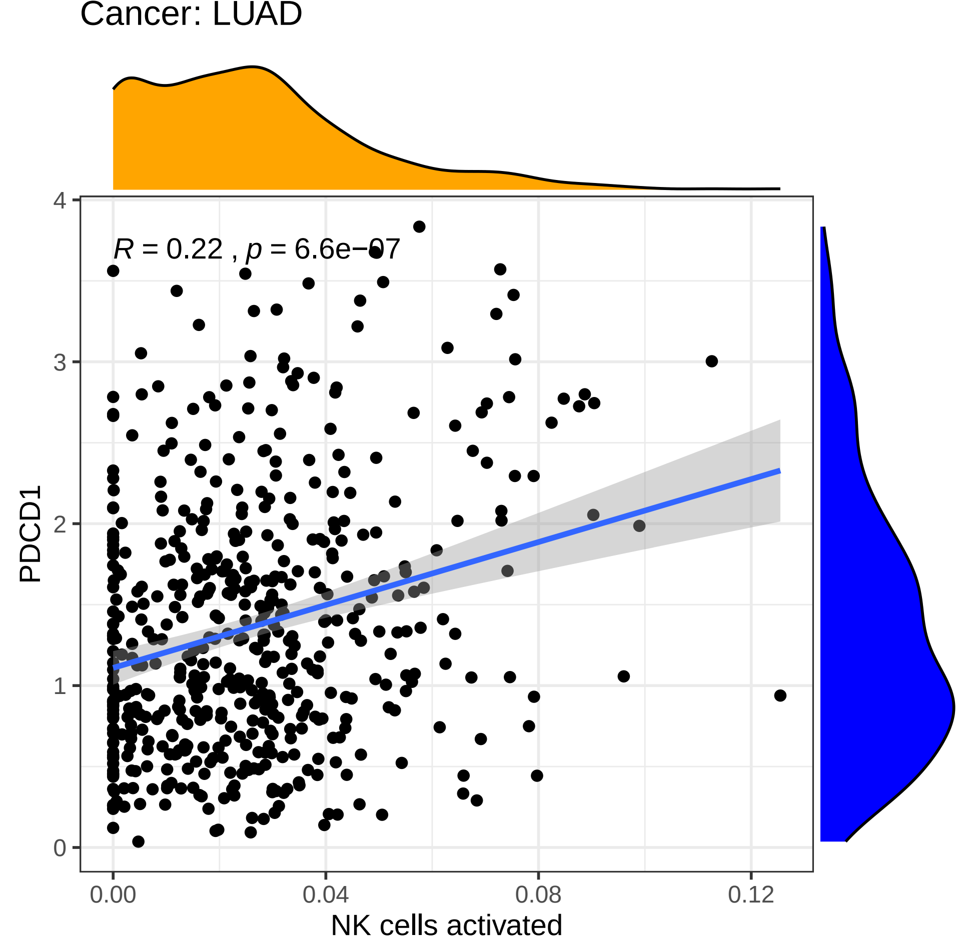
Cancer: LUAD
R = 0.22 , p = 6.6e−07
PDCD1
NK cells activated
0
1
2
3
4
0.00
0.04
0.08
0.12

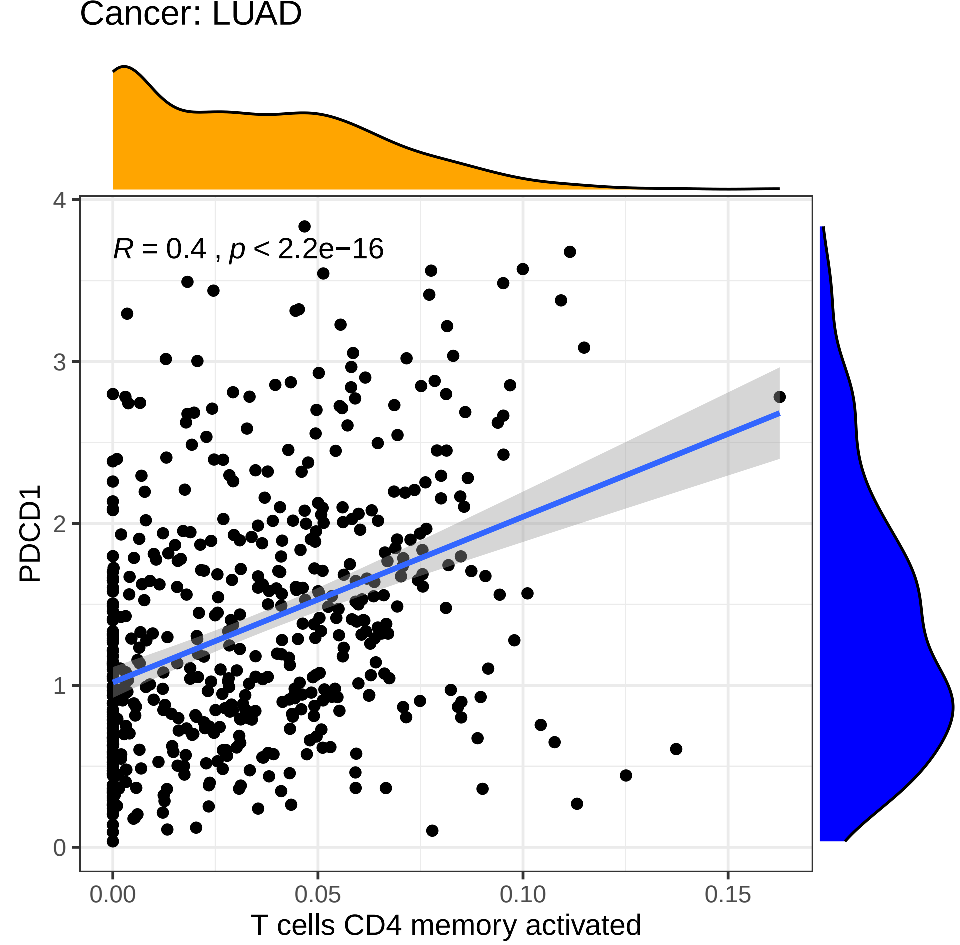
Cancer: LUAD
R = 0.4 , p < 2.2e−16
PDCD1
0
1
2
3
4
0.00
0.05
0.10
0.15
T cells CD4 memory activated

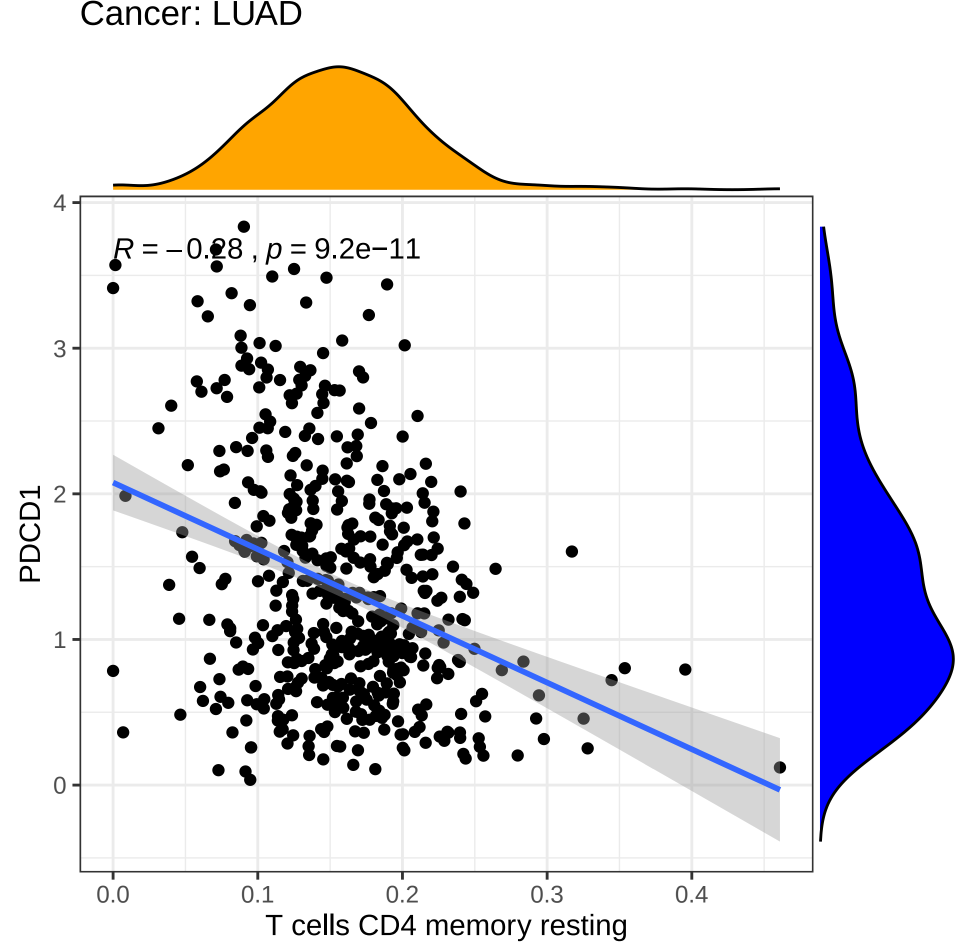

Cancer: LUAD
R = −0.28 , p = 9.2e−11
PDCD1
0
1
2
3
4
0.0
0.1
0.2
0.3
0.4
T cells CD4 memory resting

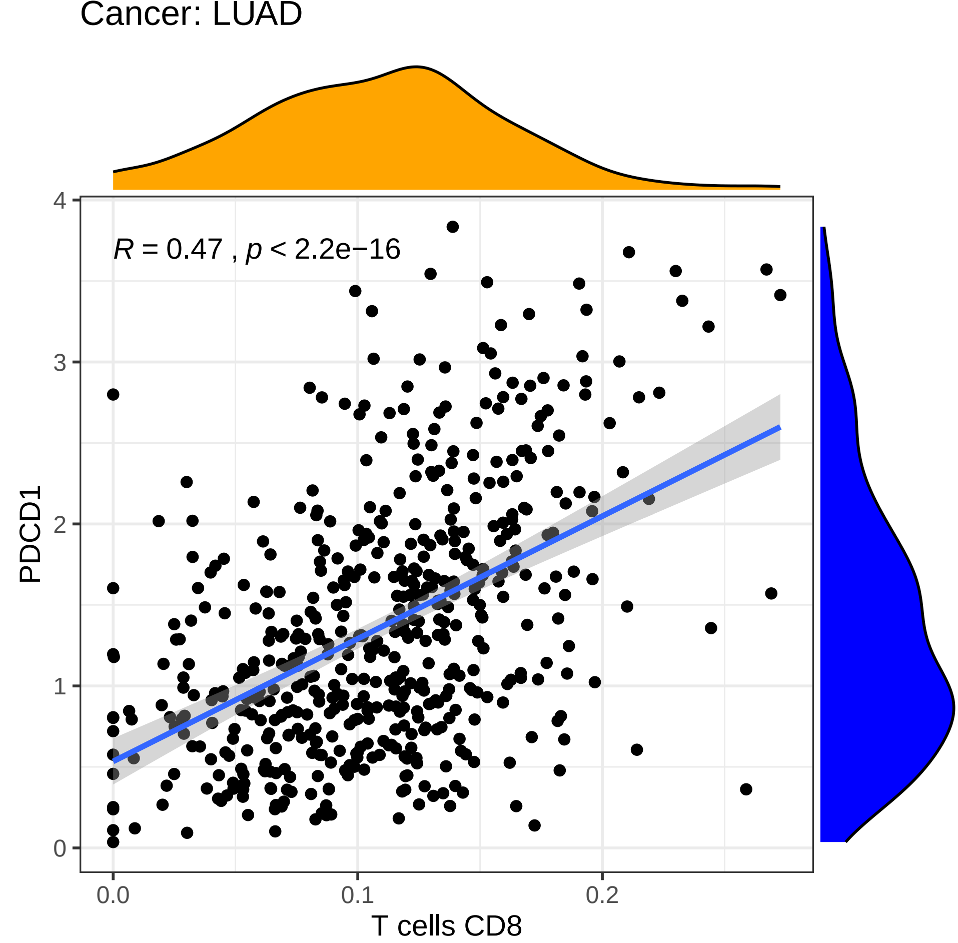

Cancer: LUAD
R = 0.47 , p < 2.2e−16
PDCD1
T cells CD8
0
1
2
3
4
0.0
0.1
0.2

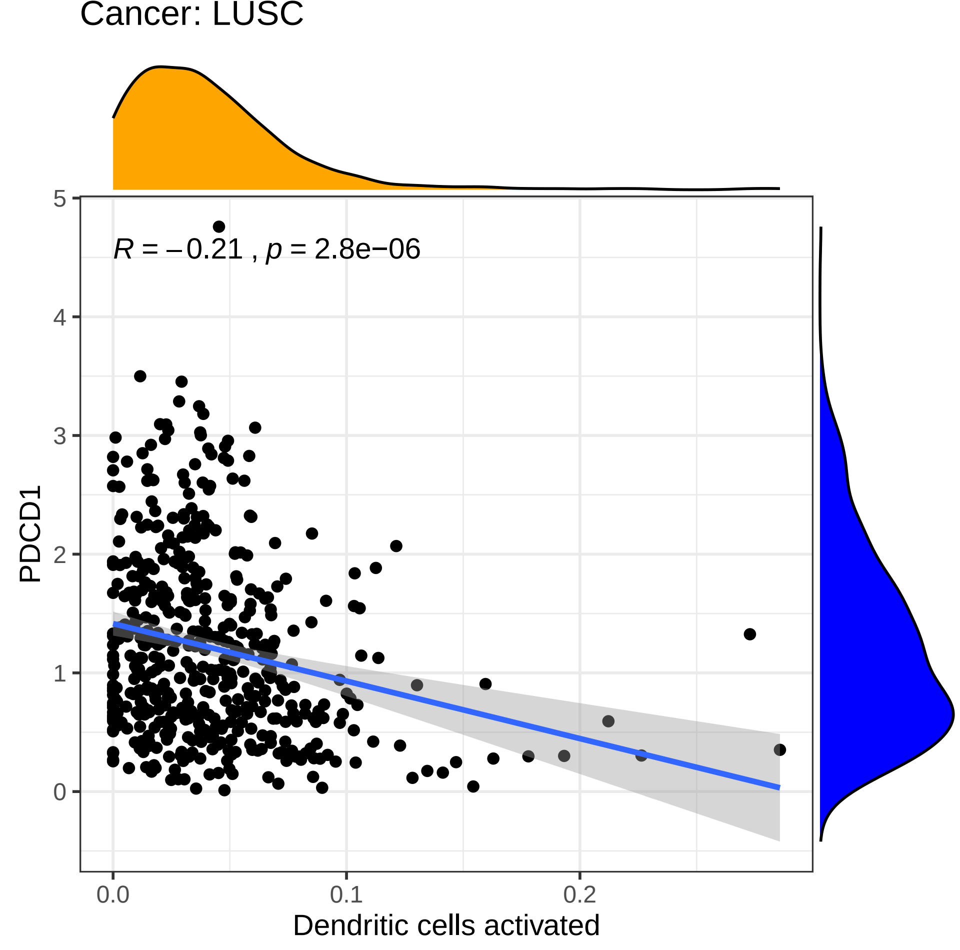

Cancer: LUSC
R = −0.21 , p = 2.8e−06
PDCD1
5
4
3
2
1
0
0.0
0.1
0.2
Dendritic cells activated

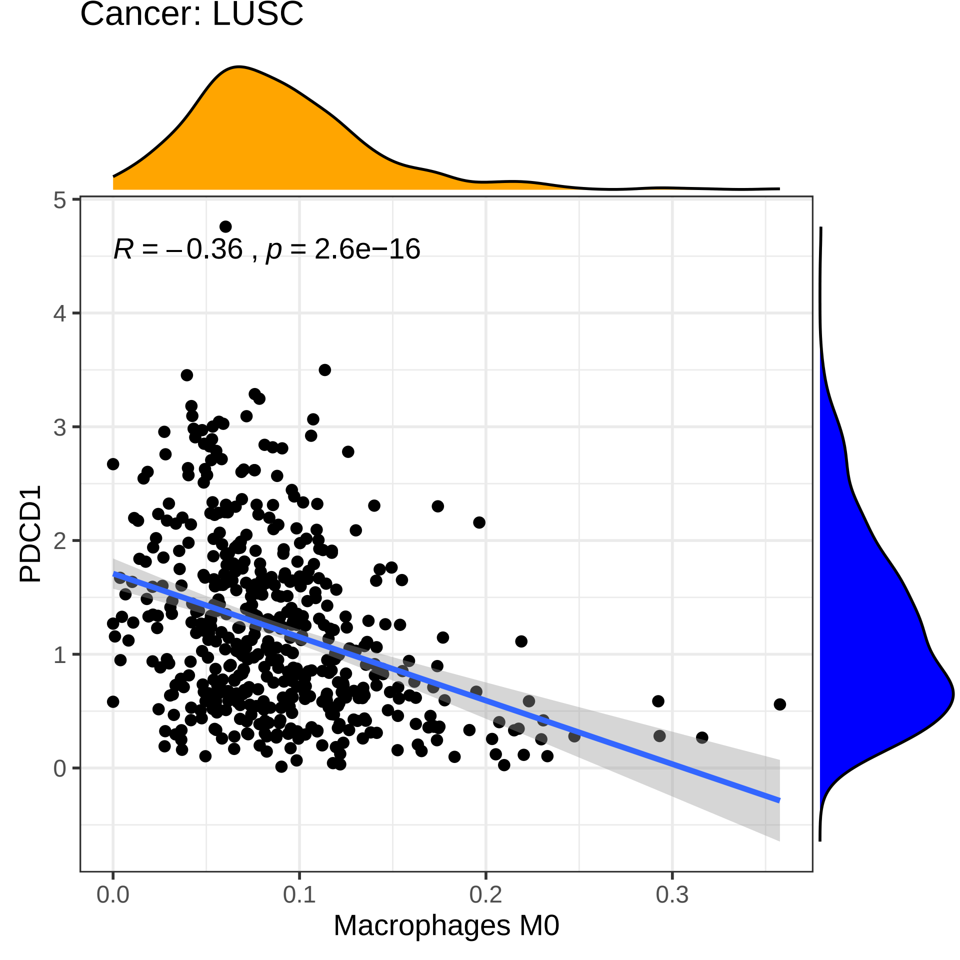

Cancer: LUSC
R = −0.36 , p = 2.6e−16
PDCD1
Macrophages M0
0
1
2
3
4
5
0.0
0.1
0.2
0.3

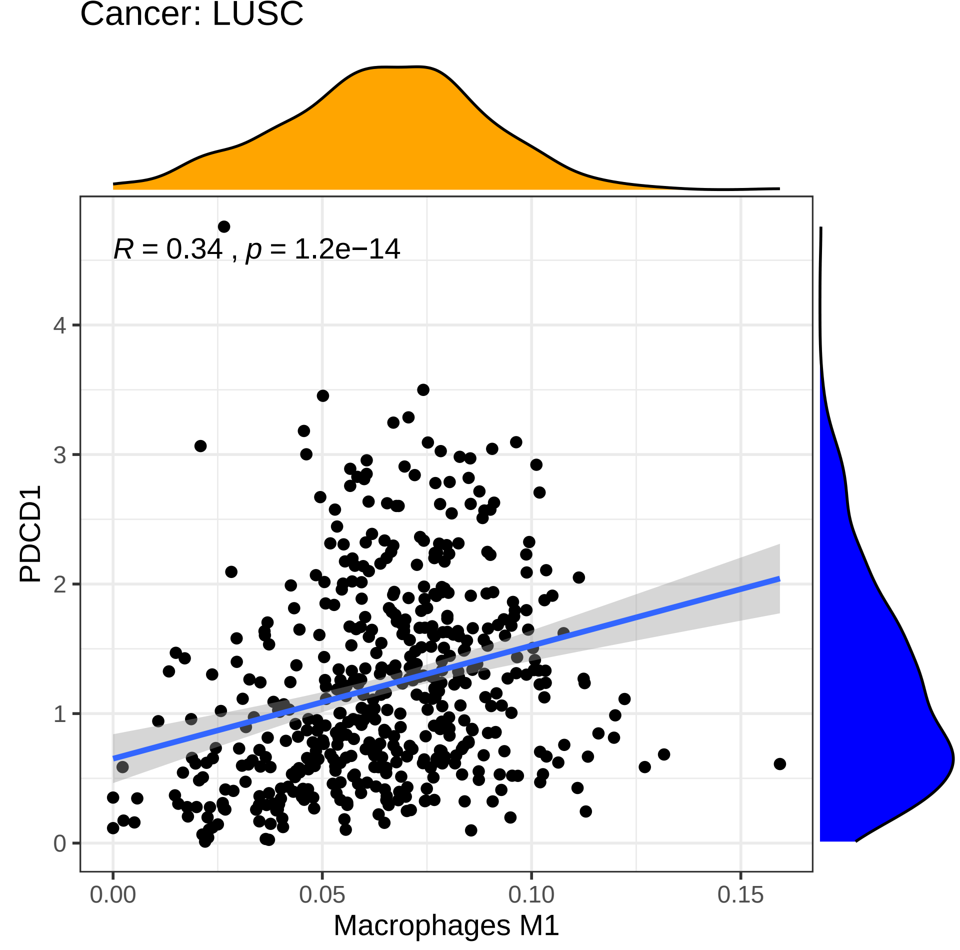
Cancer: LUSC
R = 0.34 , p = 1.2e−14
PDCD1
0
1
2
3
4
0.00
0.05
0.10
0.15
Macrophages M1

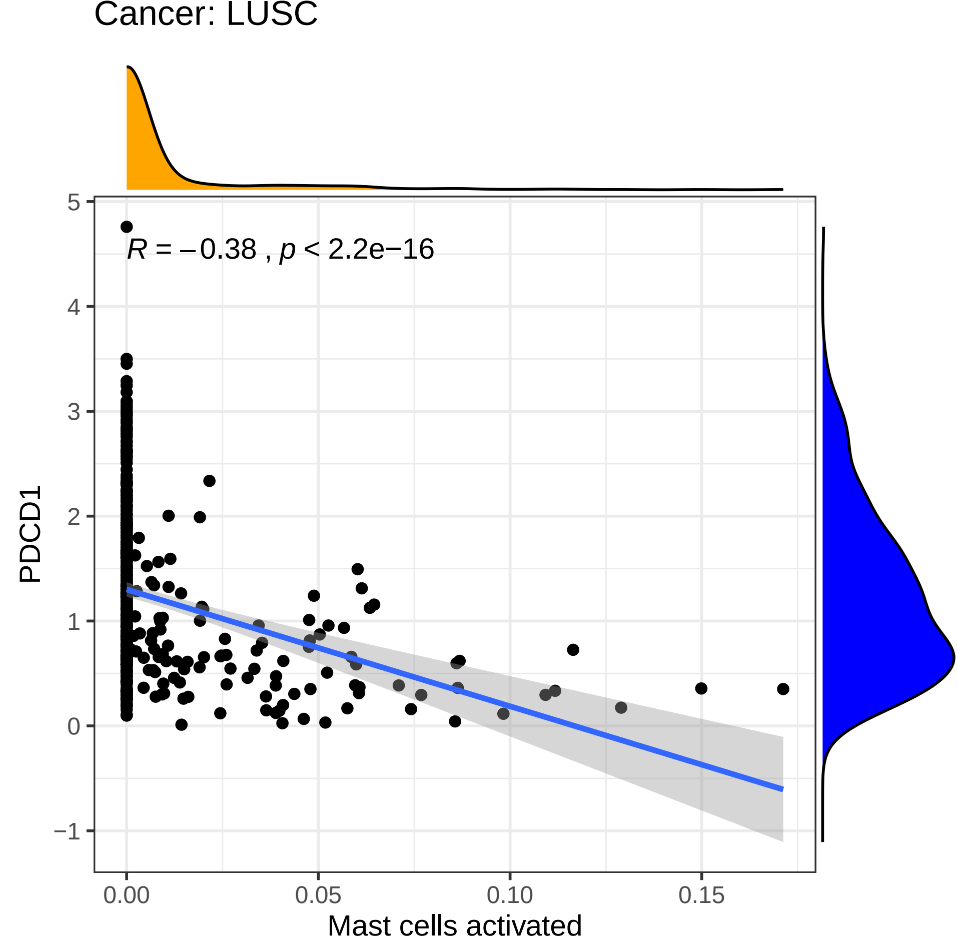

Cancer: LUSC
R = −0.38 , p < 2.2e−16
PDCD1
Mast cells activated
5
4
3
2
1
0
−1
0.00
0.05
0.10
0.15

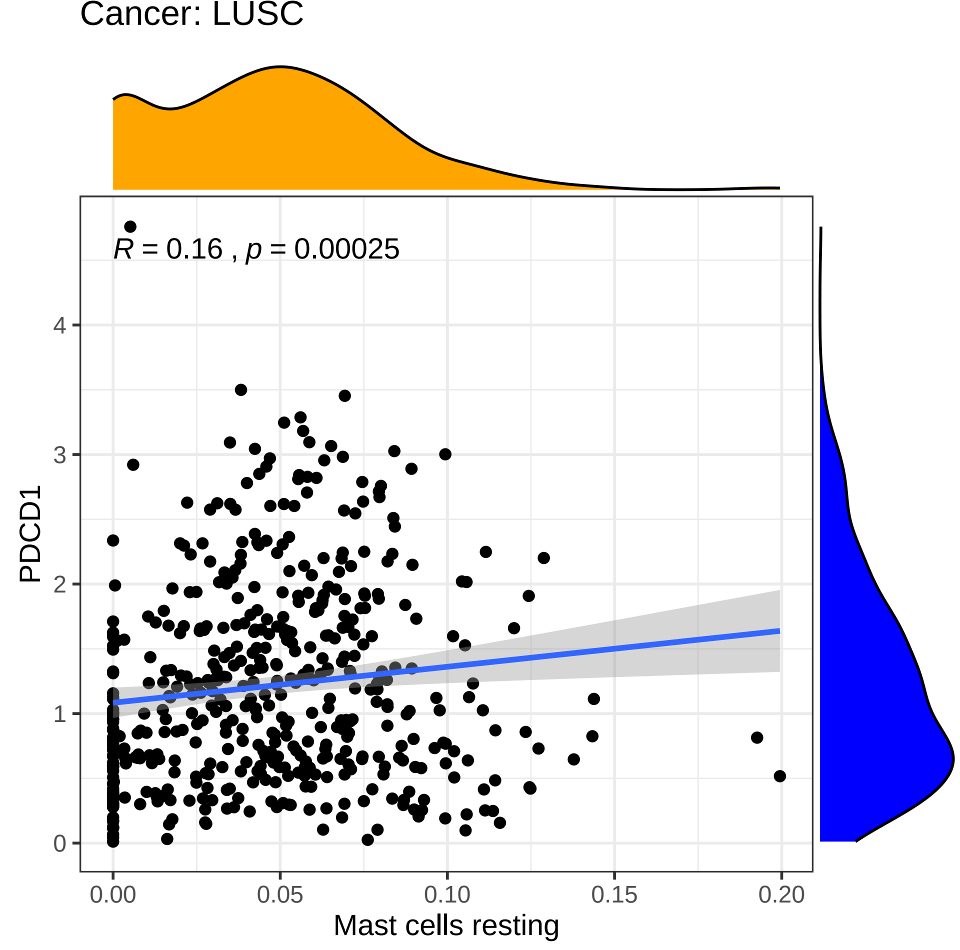

Cancer: LUSC
R = 0.16 , p = 0.00025
PDCD1
0
1
2
3
4
0.00
0.05
0.10
0.15
0.20
Mast cells resting

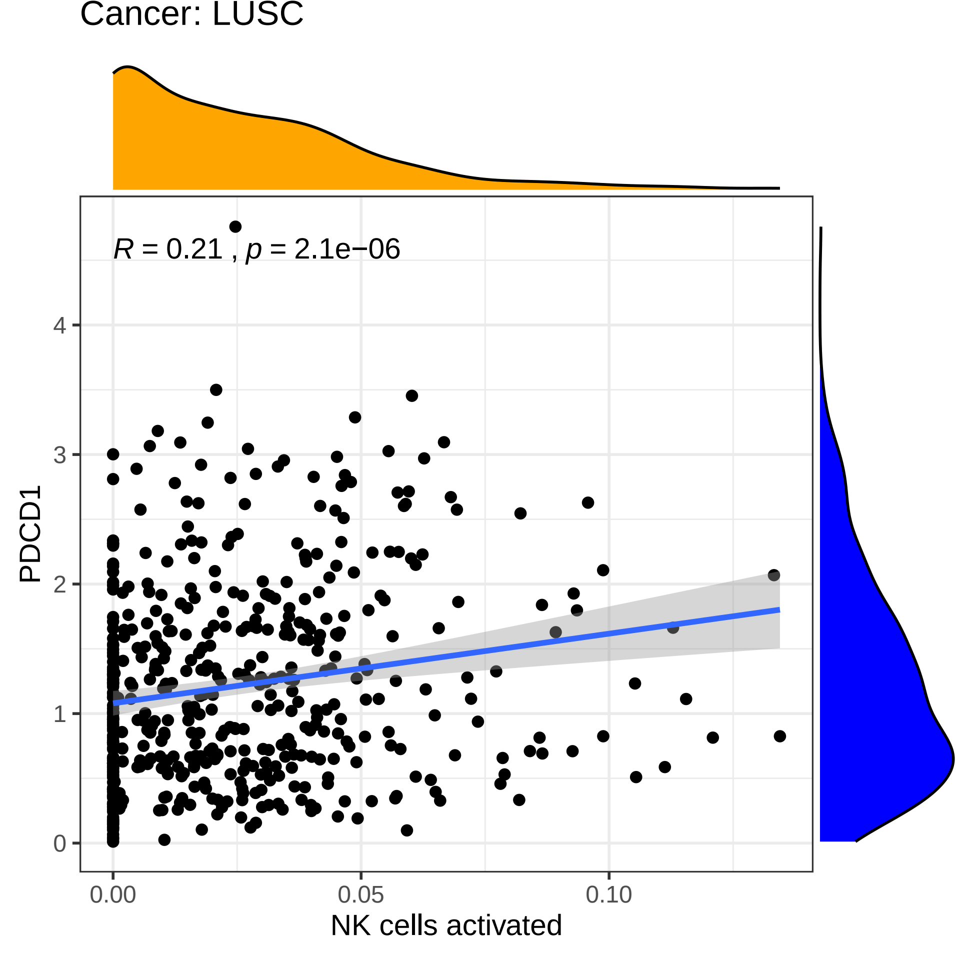

Cancer: LUSC
R = 0.21 , p = 2.1e−06
PDCD1
0
1
2
3
4
0.00
0.05
0.10
NK cells activated

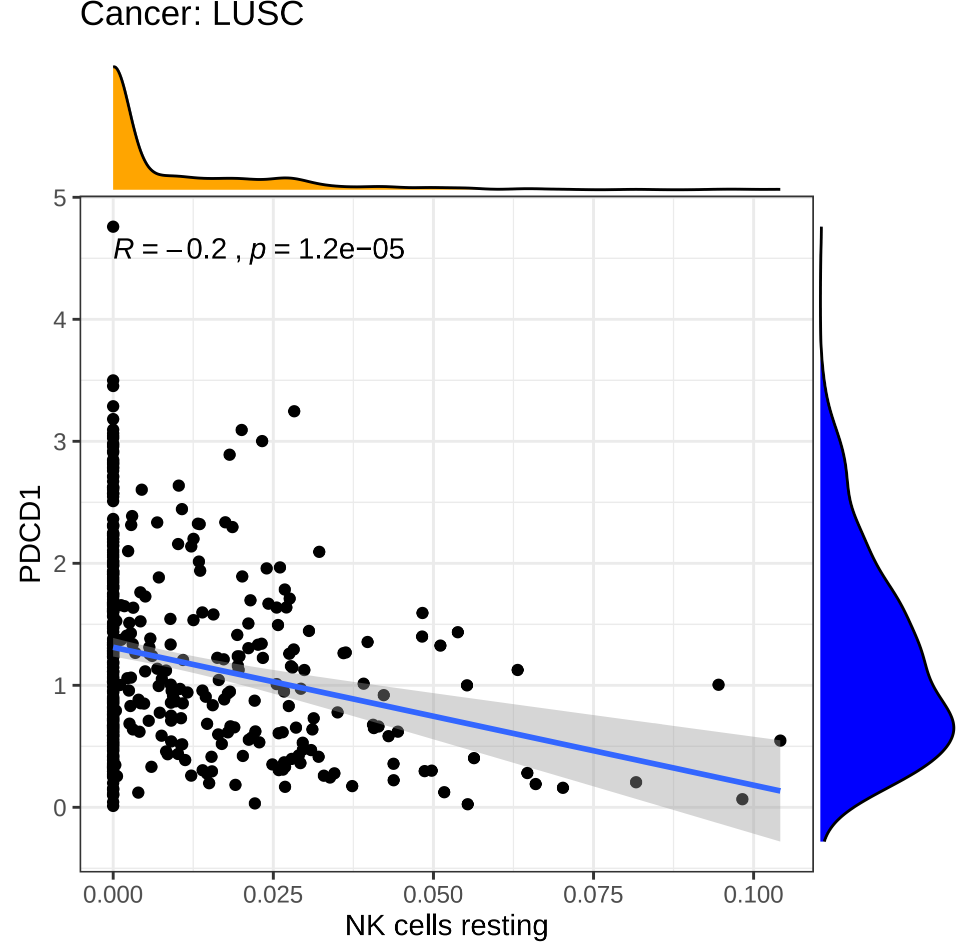

Cancer: LUSC
R = −0.2 , p = 1.2e−05
PDCD1
NK cells resting
0.000
0.025
0.050
0.075
0.100
0
1
2
3
4
5

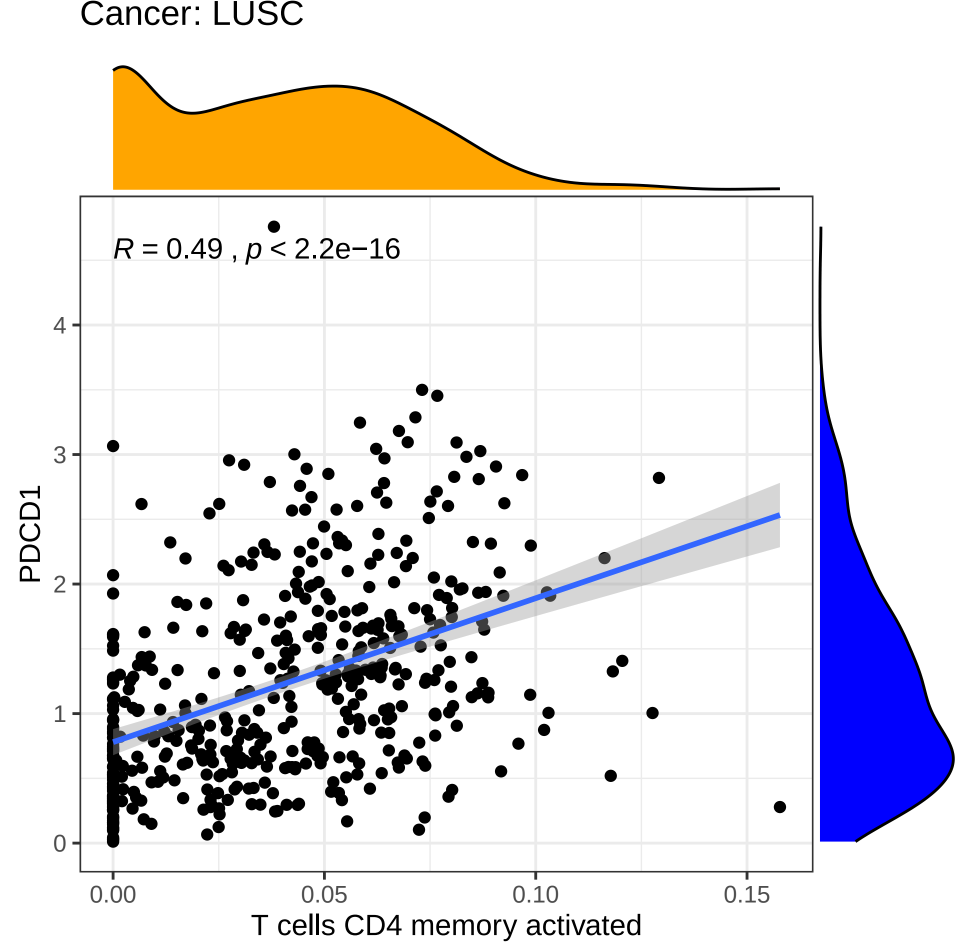
Cancer: LUSC
R = 0.49 , p < 2.2e−16
PDCD1
0
1
2
3
4
0.00
0.05
0.10
0.15
T cells CD4 memory activated

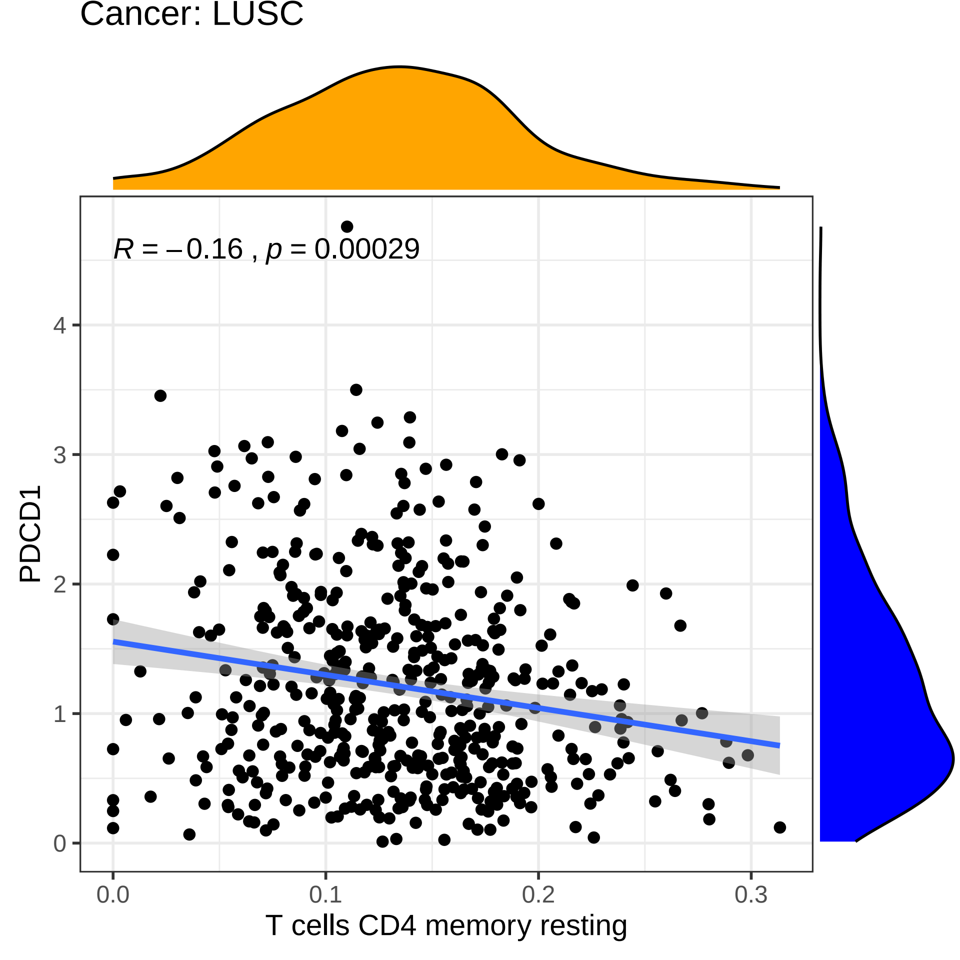

Cancer: LUSC
R = −0.16 , p = 0.00029
PDCD1
0
1
2
3
4
0.0
0.1
0.2
0.3
T cells CD4 memory resting

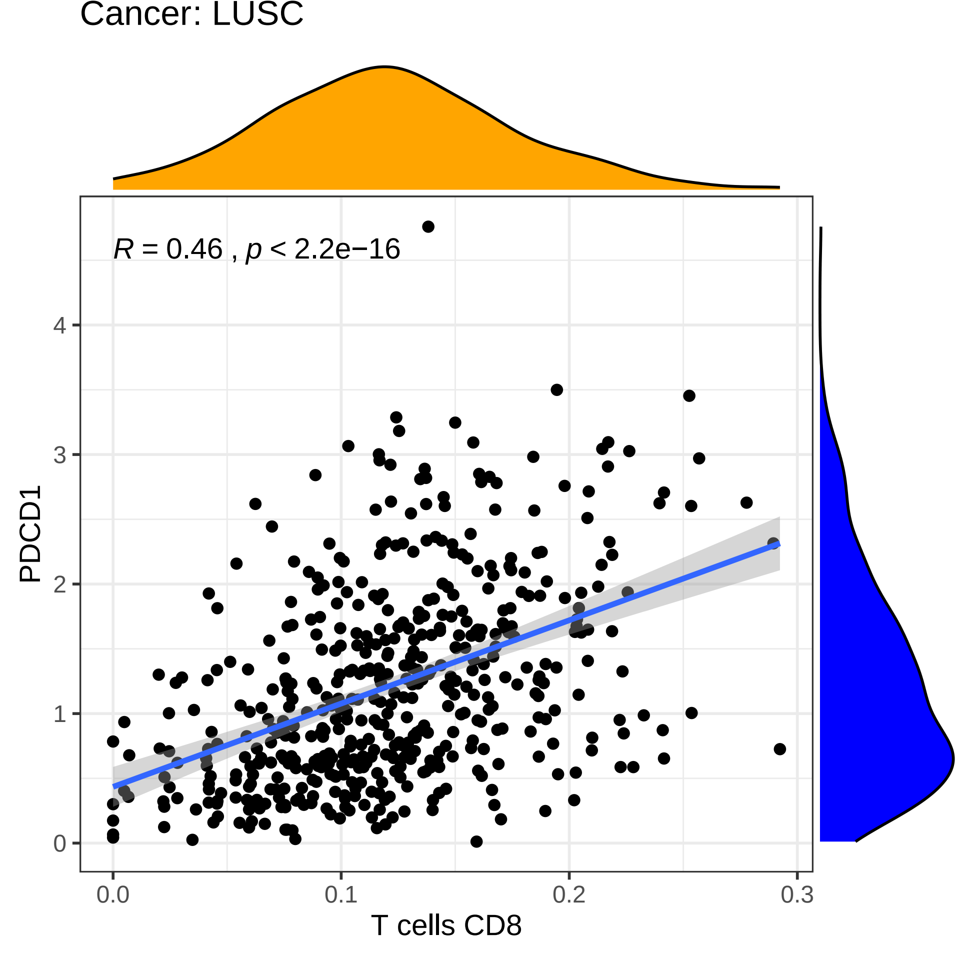

Cancer: LUSC
R = 0.46 , p < 2.2e−16
PDCD1
0
1
2
3
4
0.0
0.1
0.2
0.3
T cells CD8

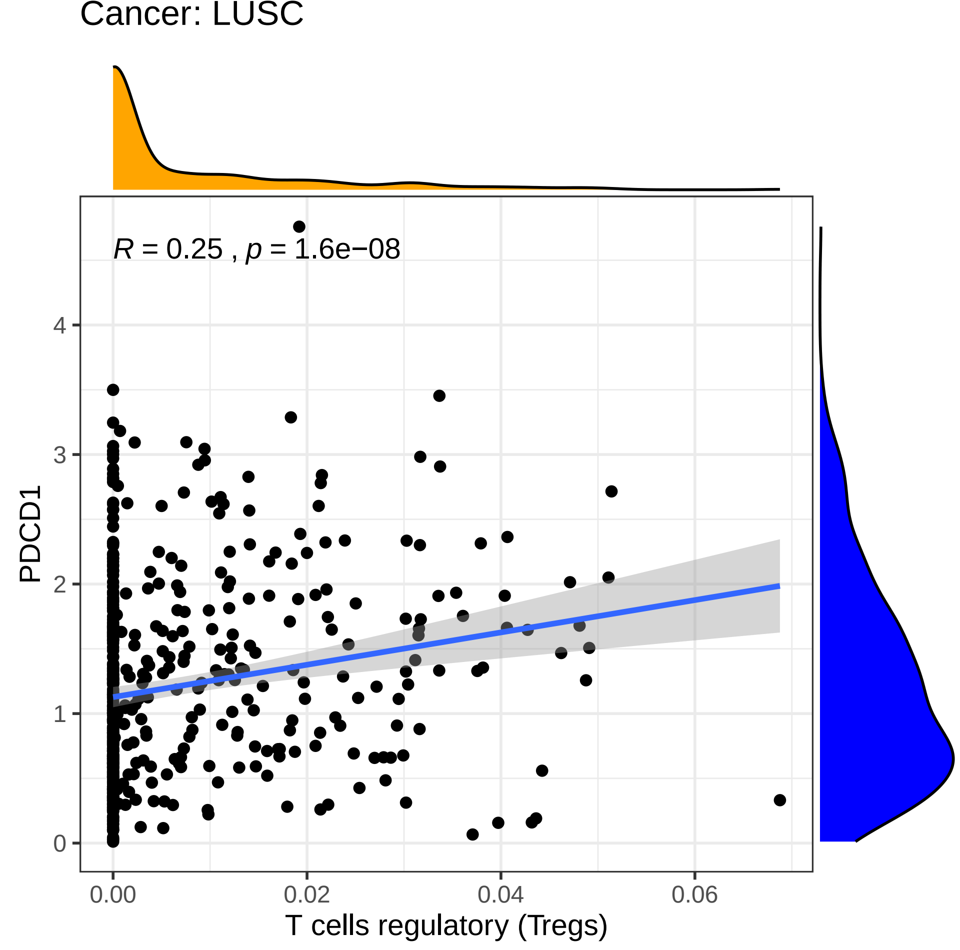
Cancer: LUSC
R = 0.25 , p = 1.6e−08
PDCD1
T cells regulatory (Tregs)
0
1
2
3
4
0.00
0.02
0.04
0.06

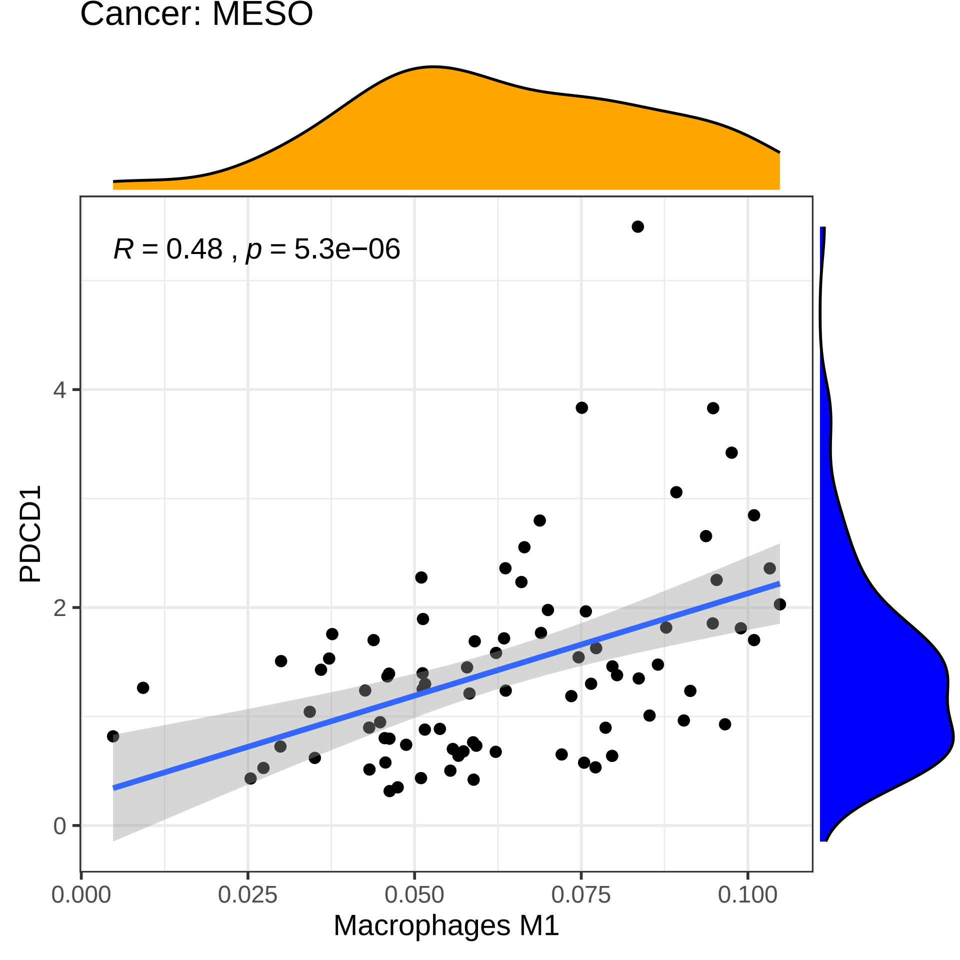
Cancer: MESO
R = 0.48 , p = 5.3e−06
PDCD1
0
2
4
0.000
0.025
0.050
0.075
0.100
Macrophages M1

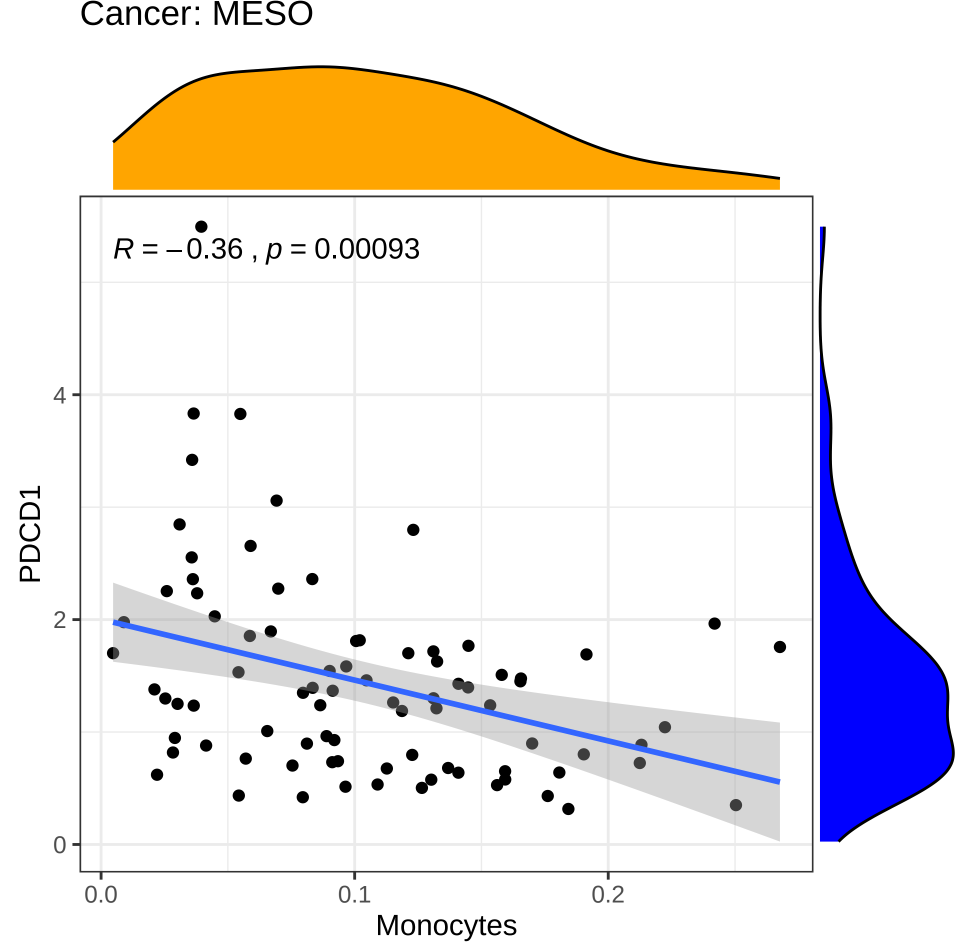

Cancer: MESO
R = −0.36 , p = 0.00093
PDCD1
0
2
4
0.0
0.1
0.2
Monocytes

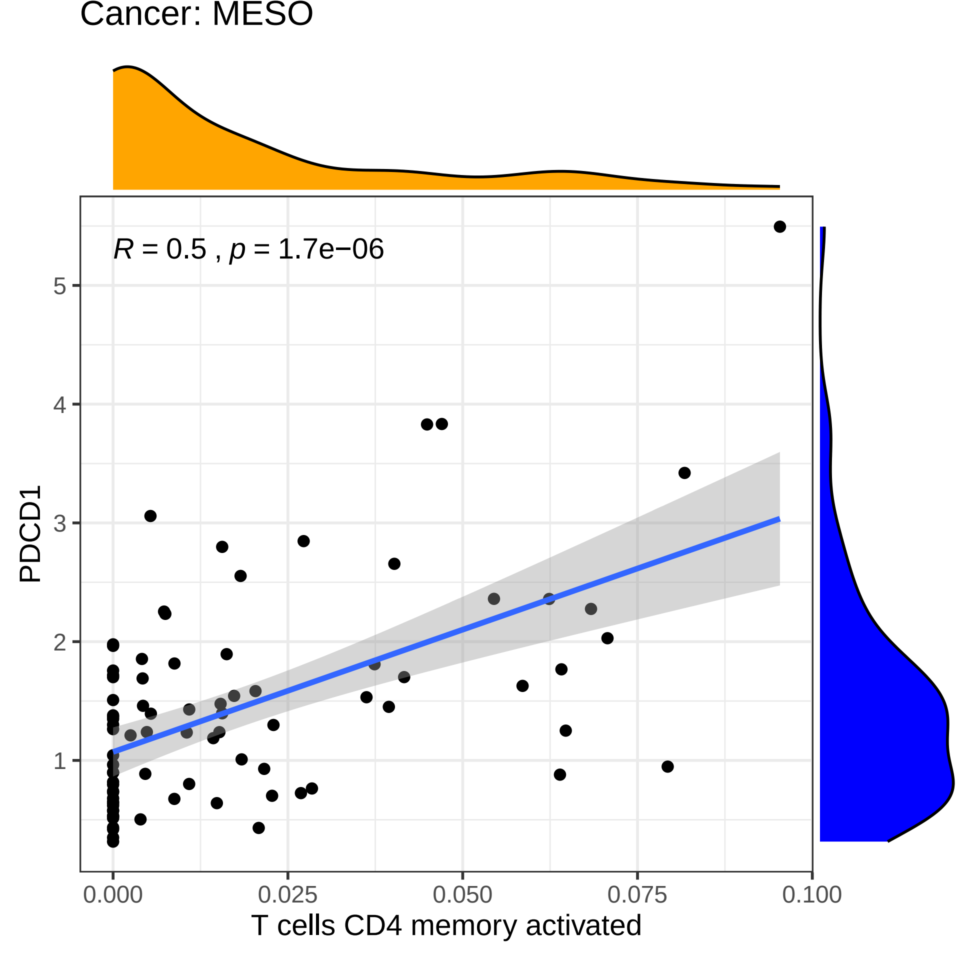

Cancer: MESO
R = 0.5 , p = 1.7e−06
PDCD1
1
2
3
4
5
0.000
0.025
0.050
0.075
0.100
T cells CD4 memory activated

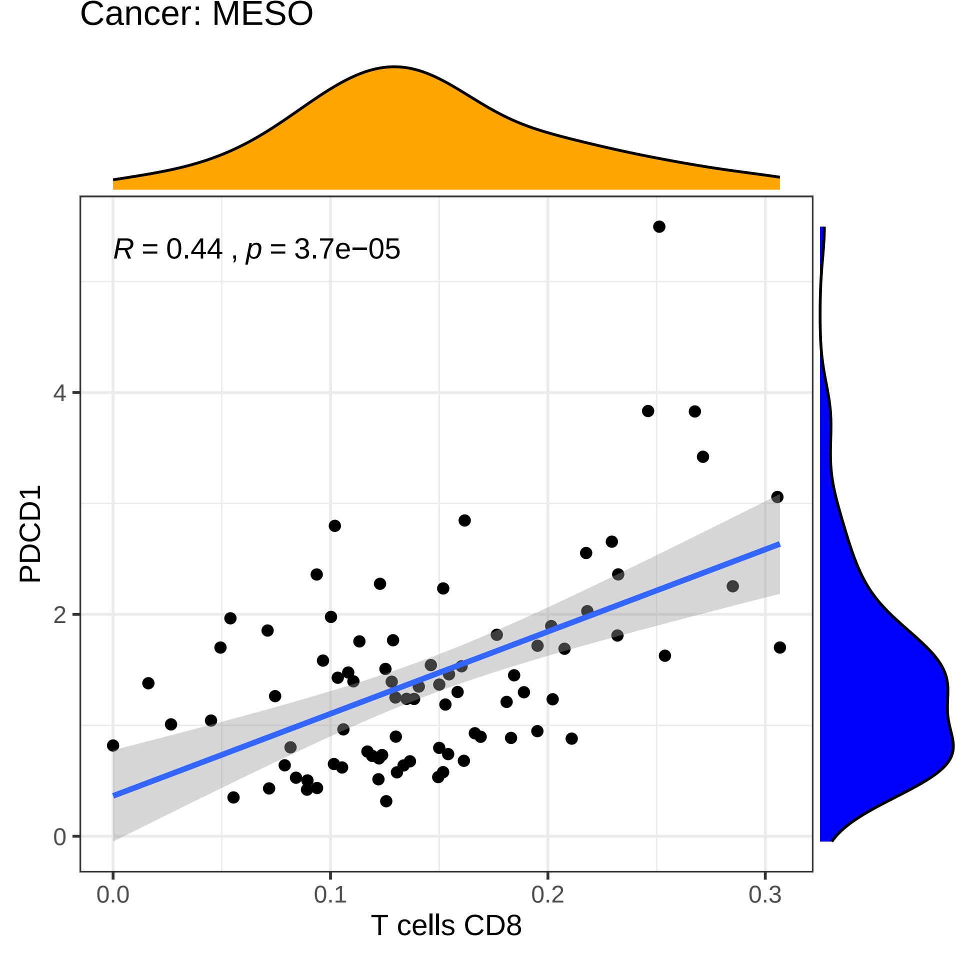

Cancer: MESO
R = 0.44 , p = 3.7e−05
PDCD1
0
2
4
0.0
0.1
0.2
0.3
T cells CD8

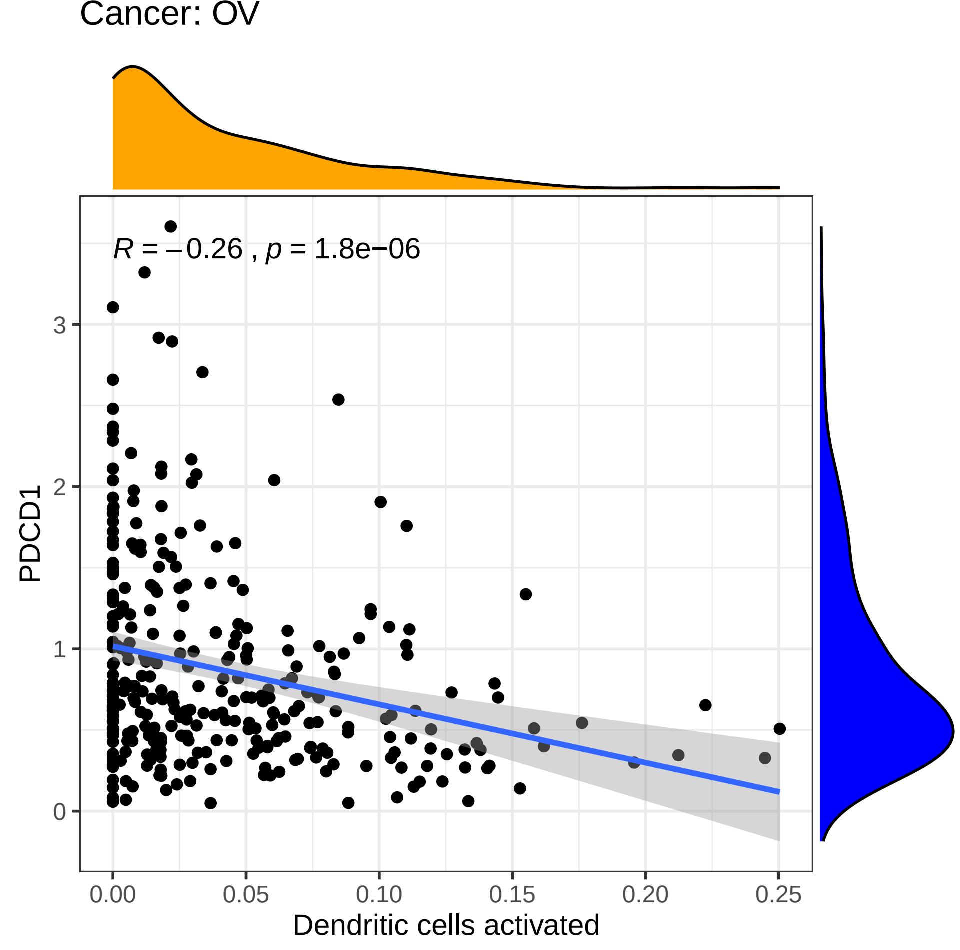
Cancer: OV
R = −0.26 , p = 1.8e−06
PDCD1
0
1
2
3
0.00
0.05
0.10
0.15
0.20
0.25
Dendritic cells activated

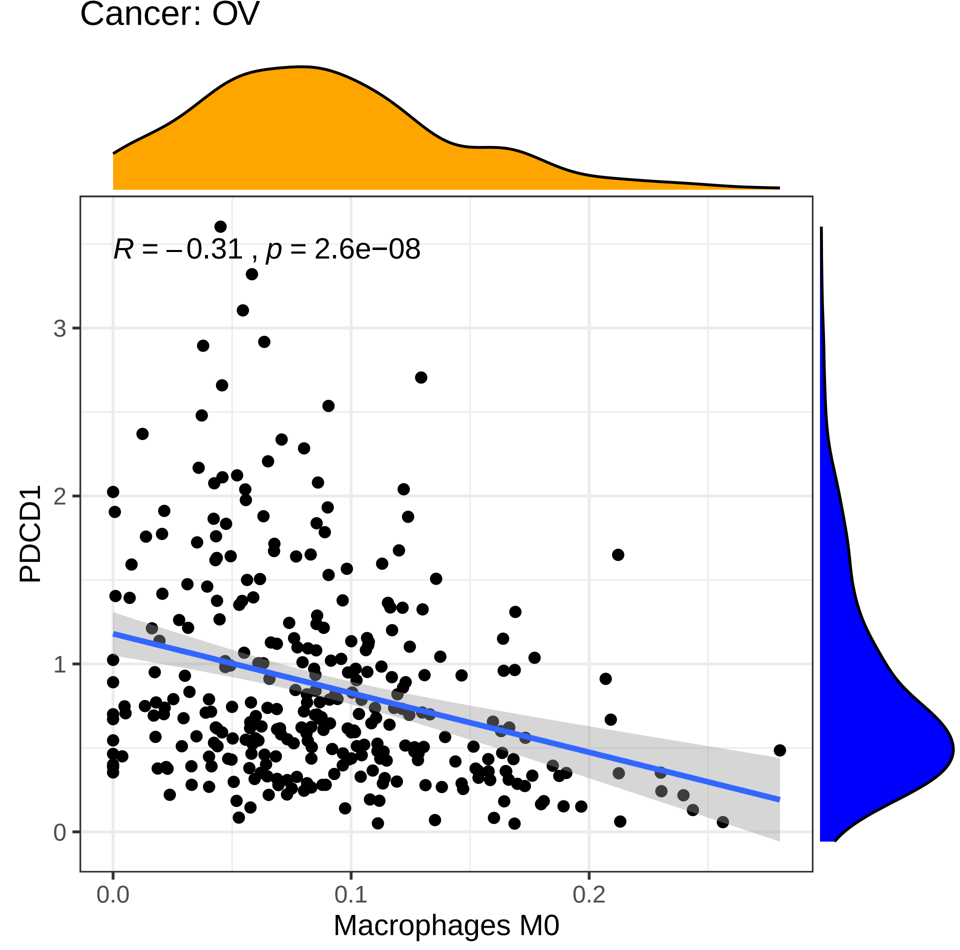

Cancer: OV
R = −0.31 , p = 2.6e−08
PDCD1
0
1
2
3
0.0
0.1
0.2
Macrophages M0

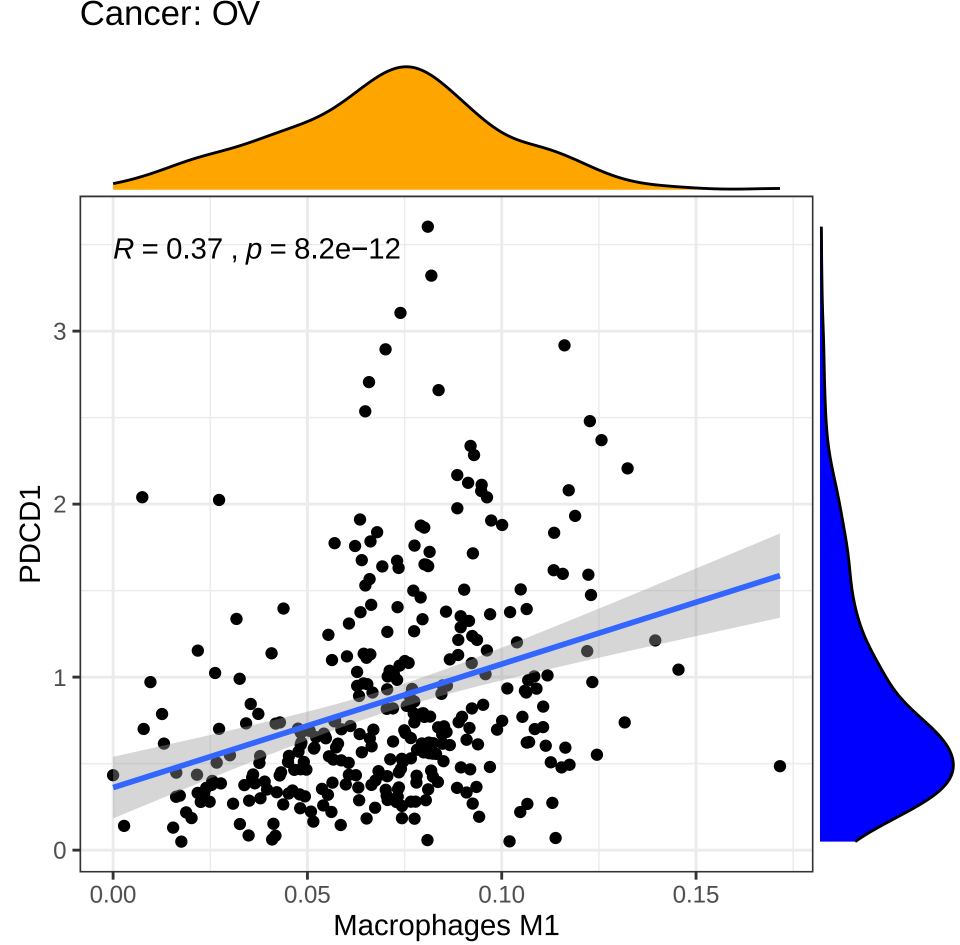

Cancer: OV
R = 0.37 , p = 8.2e−12
PDCD1
0
1
2
3
0.00
0.05
0.10
0.15
Macrophages M1

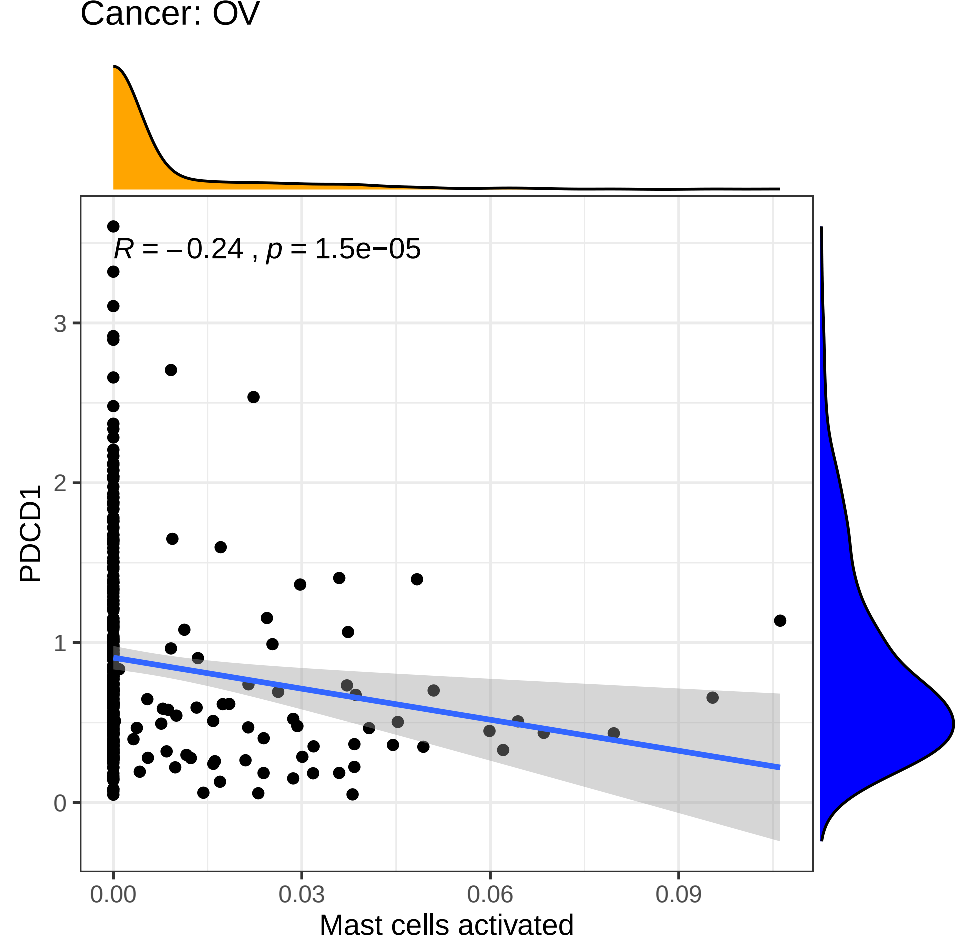

Cancer: OV
R = −0.24 , p = 1.5e−05
PDCD1
0
1
2
3
0.00
0.03
0.06
0.09
Mast cells activated

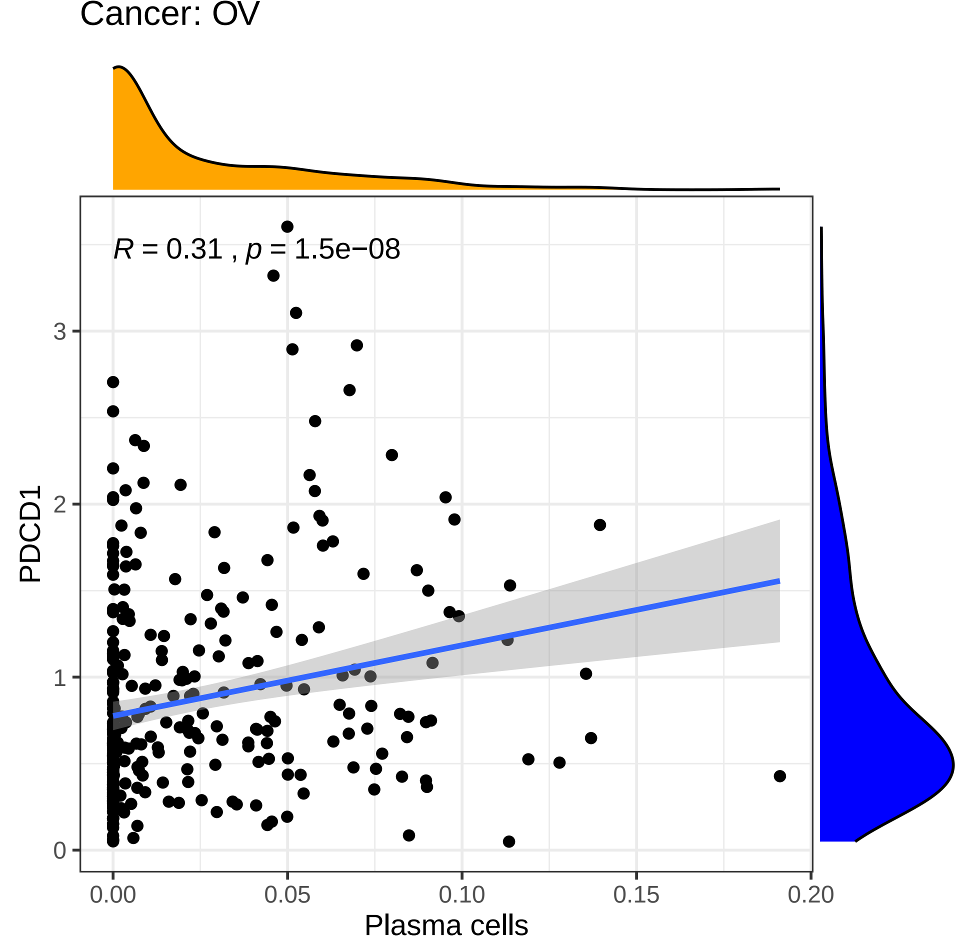
Cancer: OV
R = 0.31 , p = 1.5e−08
PDCD1
Plasma cells
0
1
2
3
0.00
0.05
0.10
0.15
0.20

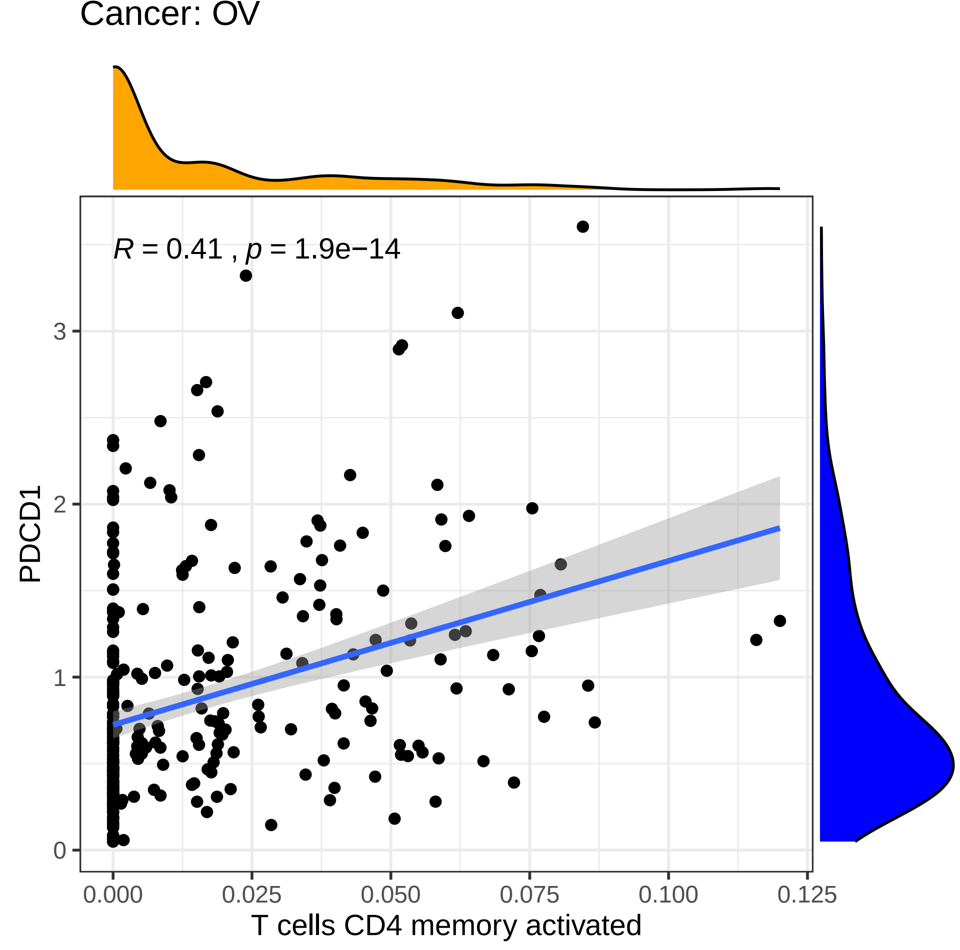

Cancer: OV
R = 0.41 , p = 1.9e−14
PDCD1
0
1
2
3
0.000
0.025
0.050
0.075
0.100
0.125
T cells CD4 memory activated

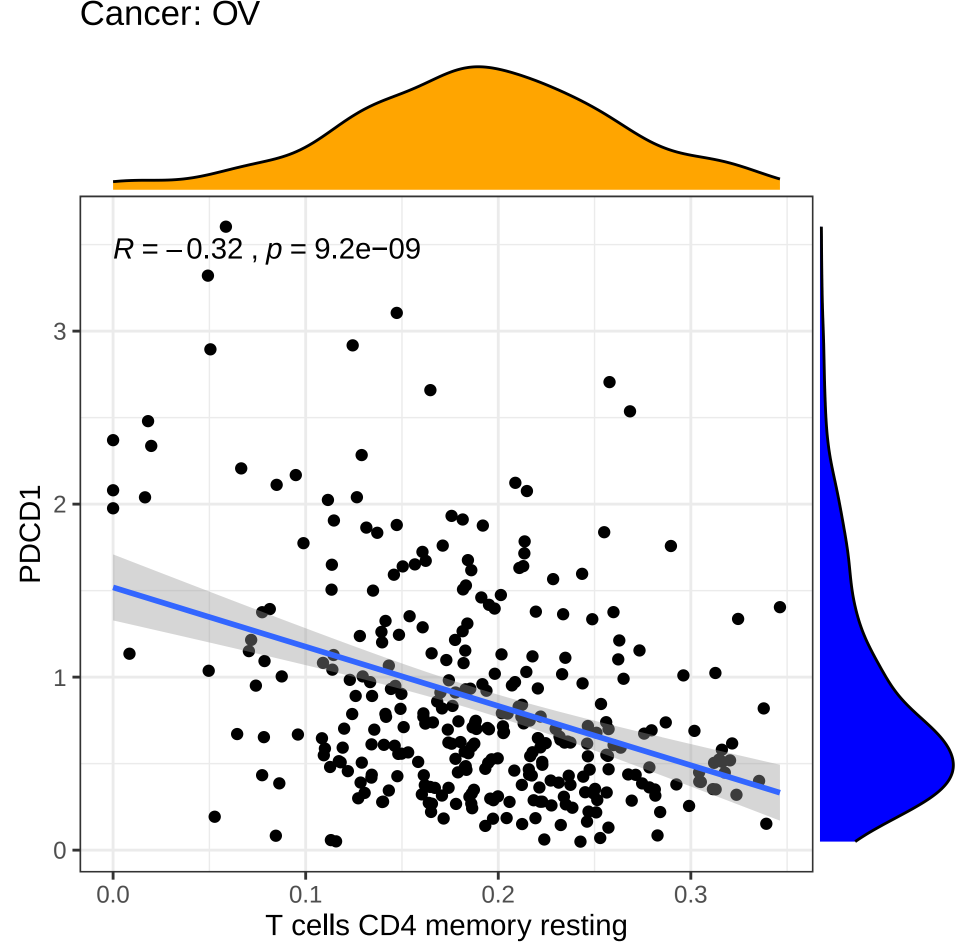
Cancer: OV
R = −0.32 , p = 9.2e−09
PDCD1
0
1
2
3
0.0
0.1
0.2
0.3
T cells CD4 memory resting

Cancer: OV

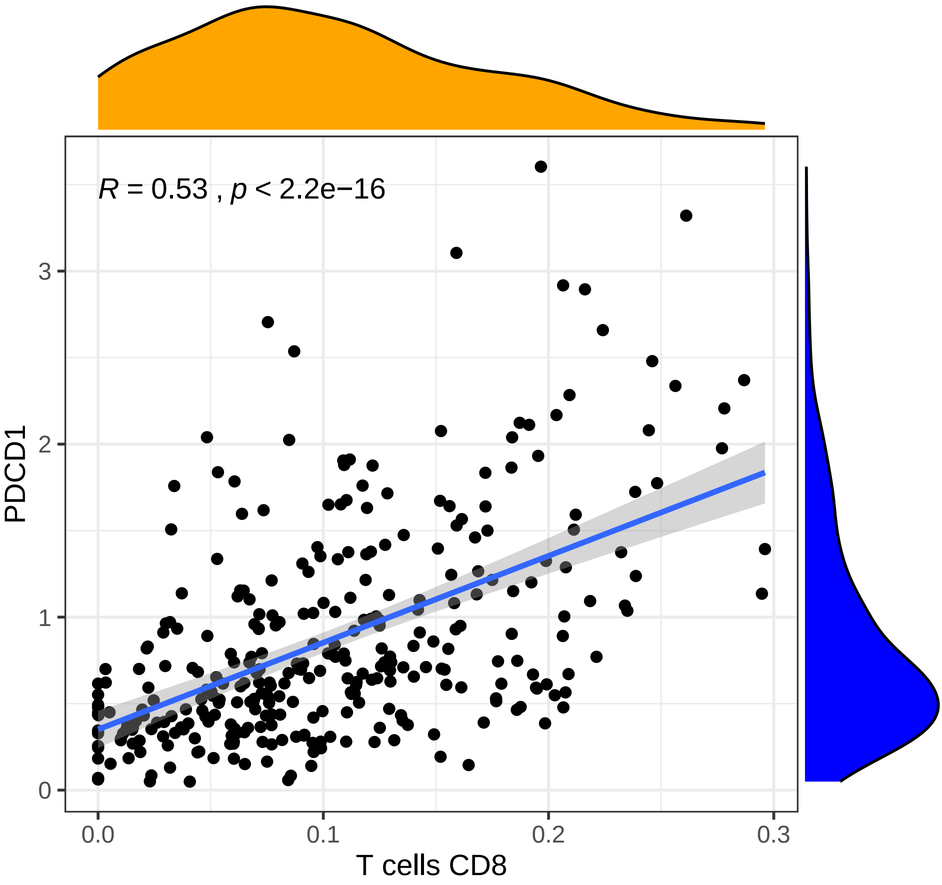

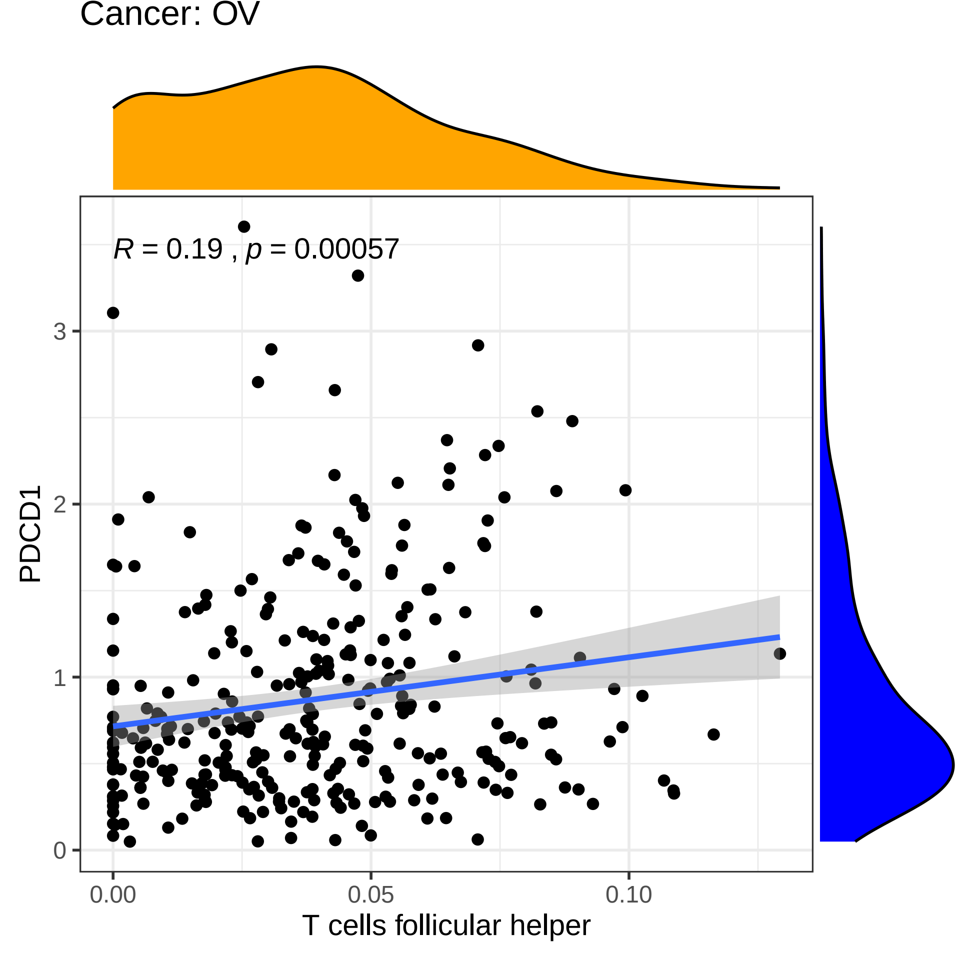
Cancer: OV
R = 0.19 , p = 0.00057
PDCD1
T cells follicular helper
0
1
2
3
0.00
0.05
0.10

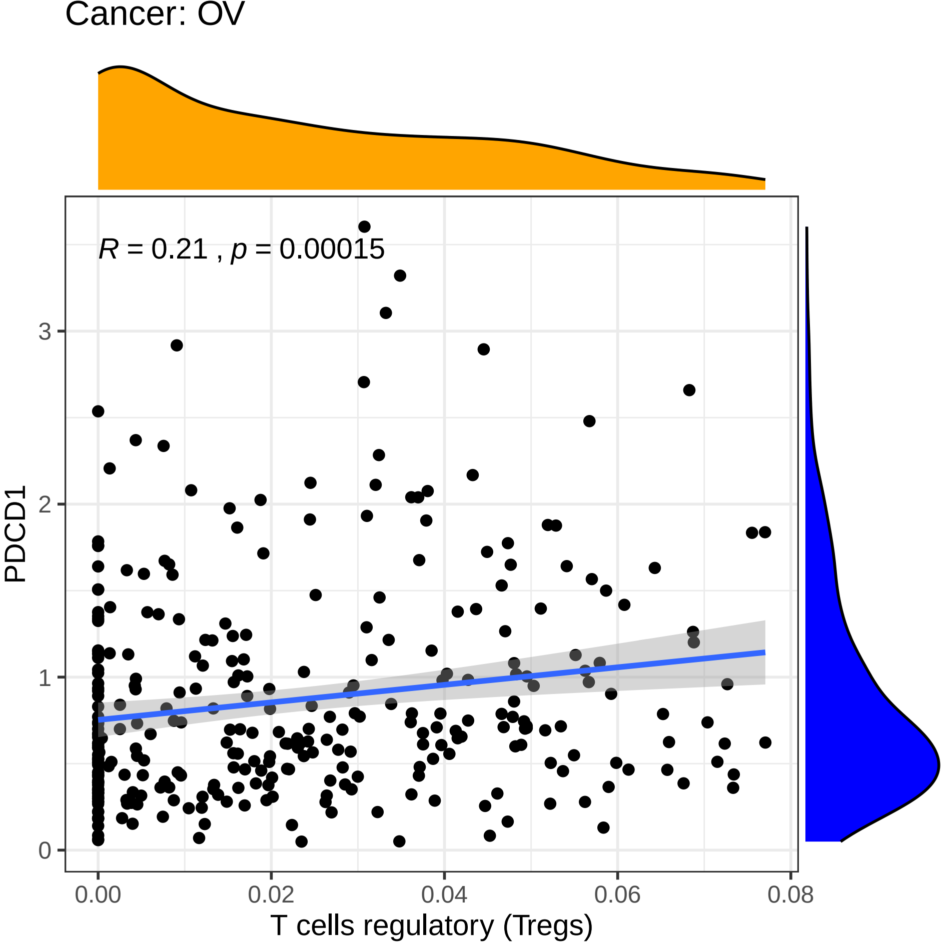
Cancer: OV
R = 0.21 , p = 0.00015
PDCD1
0
1
2
3
0.00
0.02
0.04
0.06
0.08
T cells regulatory (Tregs)

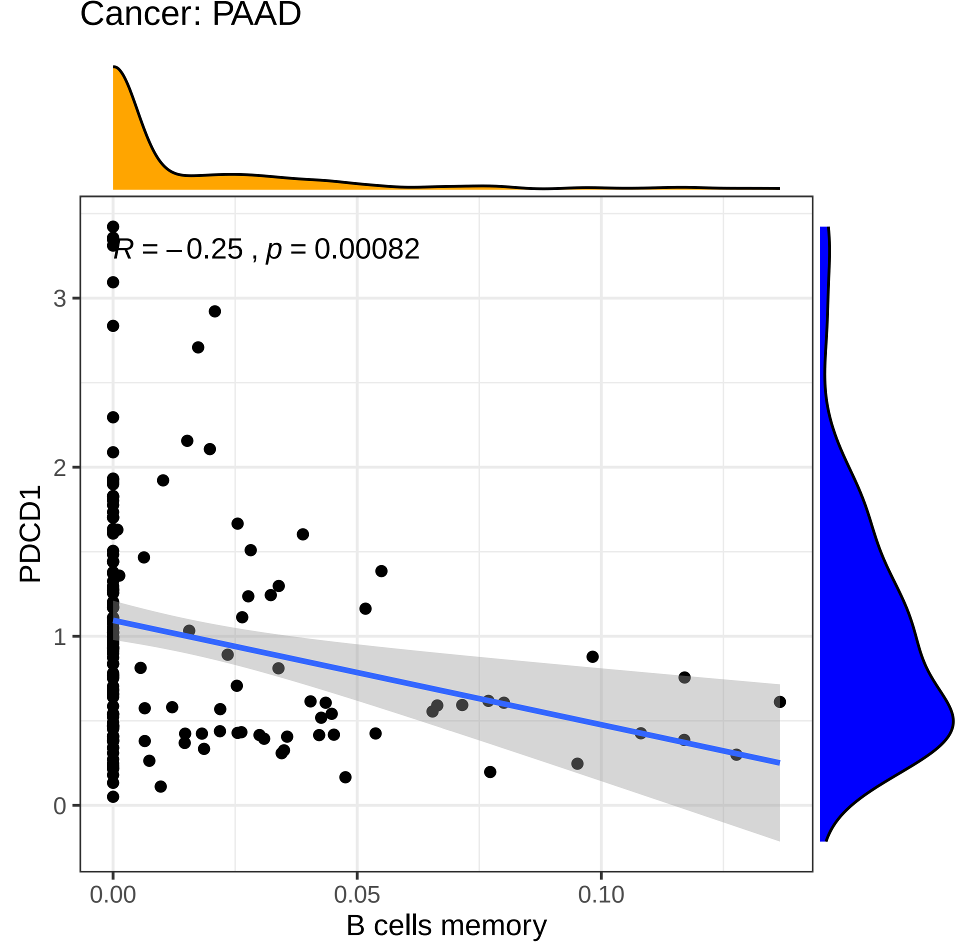

Cancer: PAAD
R = −0.25 , p = 0.00082
PDCD1
B cells memory
0
1
2
3
0.00
0.05
0.10

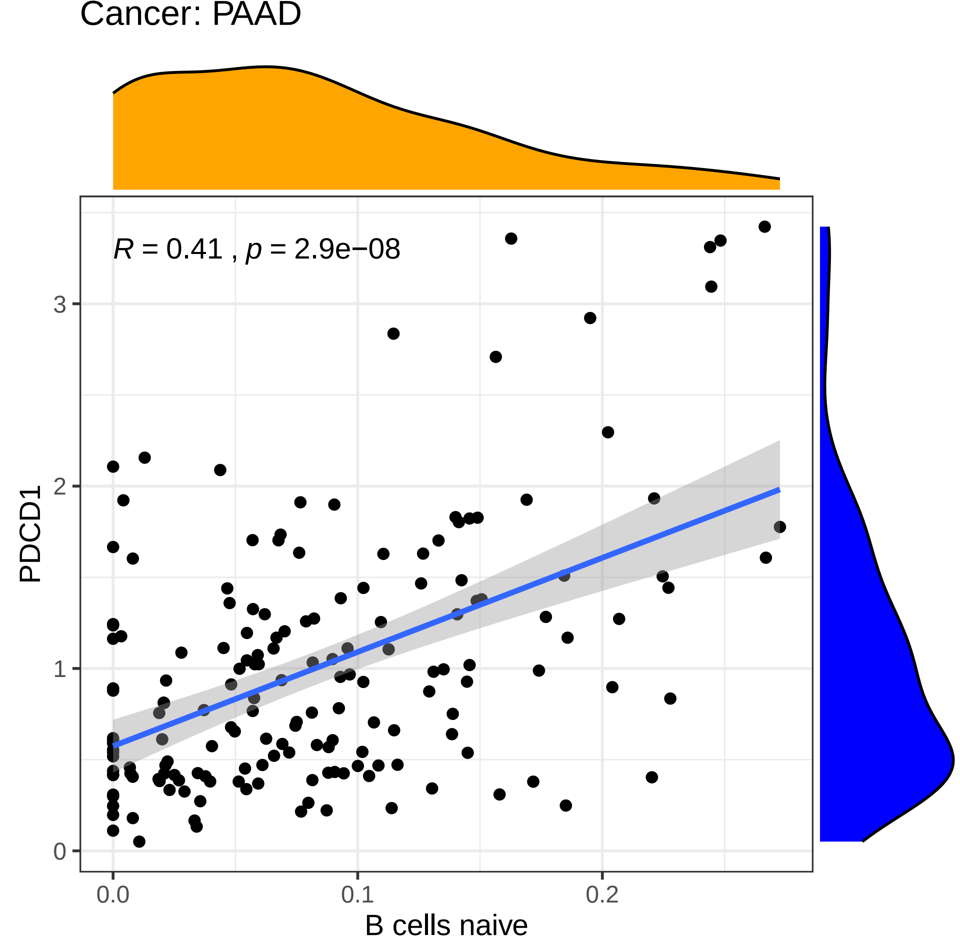

Cancer: PAAD
R = 0.41 , p = 2.9e−08
PDCD1
B cells naive
0
1
2
3
0.0
0.1
0.2

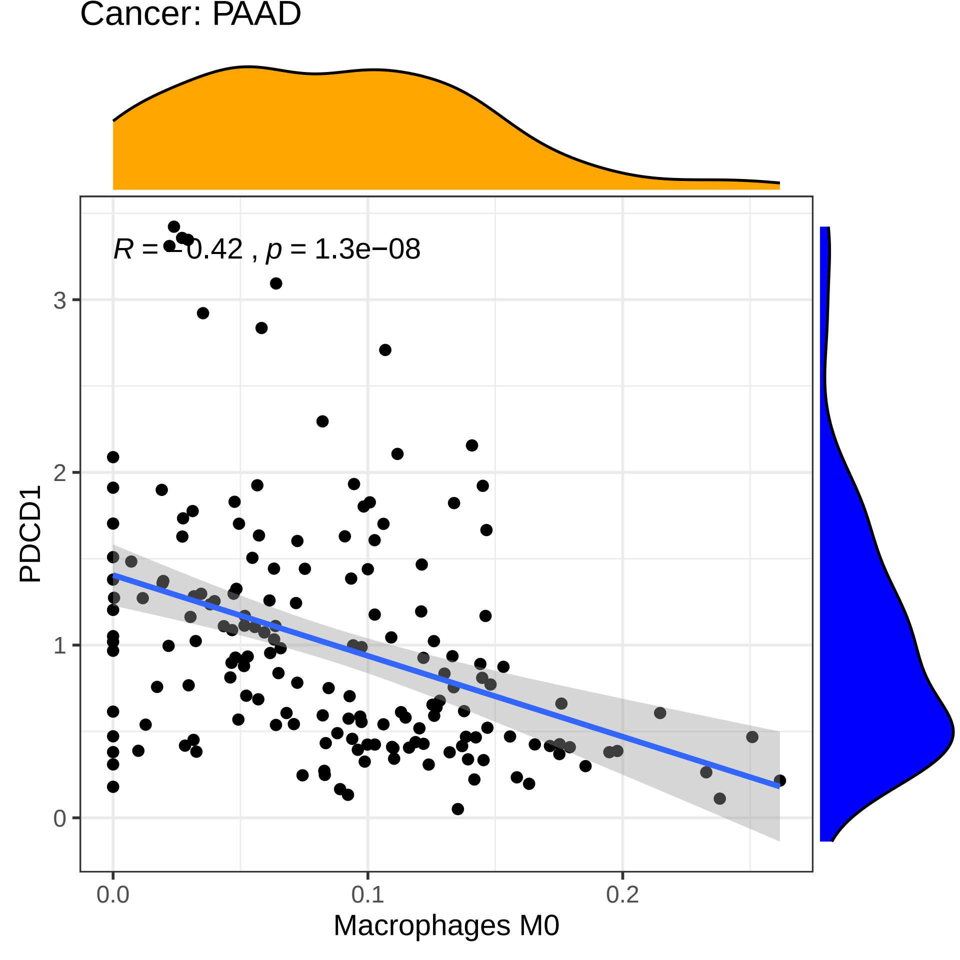

Cancer: PAAD
R = −0.42 , p = 1.3e−08
PDCD1
0
1
2
3
0.0
0.1
0.2
Macrophages M0

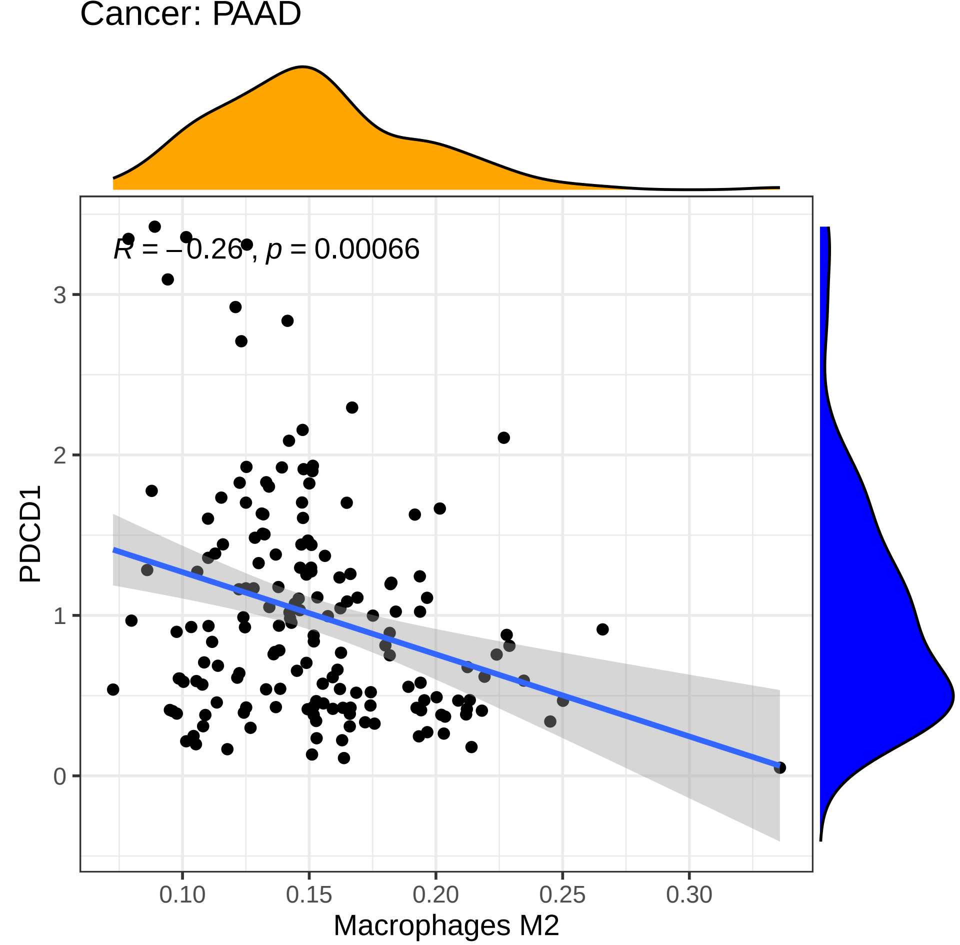
Cancer: PAAD
R = −0.26, p = 0.00066
PDCD1
0
1
2
3
0.10
0.15
0.20
0.25
0.30
Macrophages M2

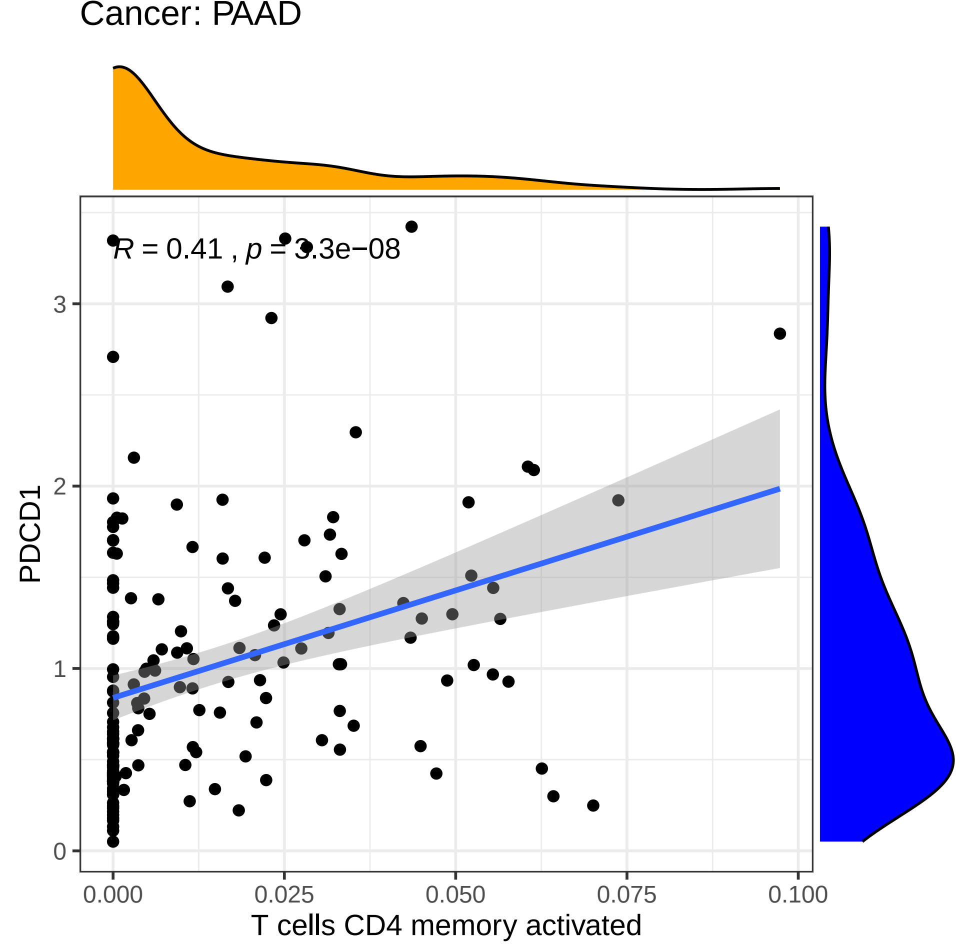

Cancer: PAAD
R = 0.41 , p = 3.3e−08
PDCD1
0
1
2
3
0.000
0.025
0.050
0.075
0.100
T cells CD4 memory activated

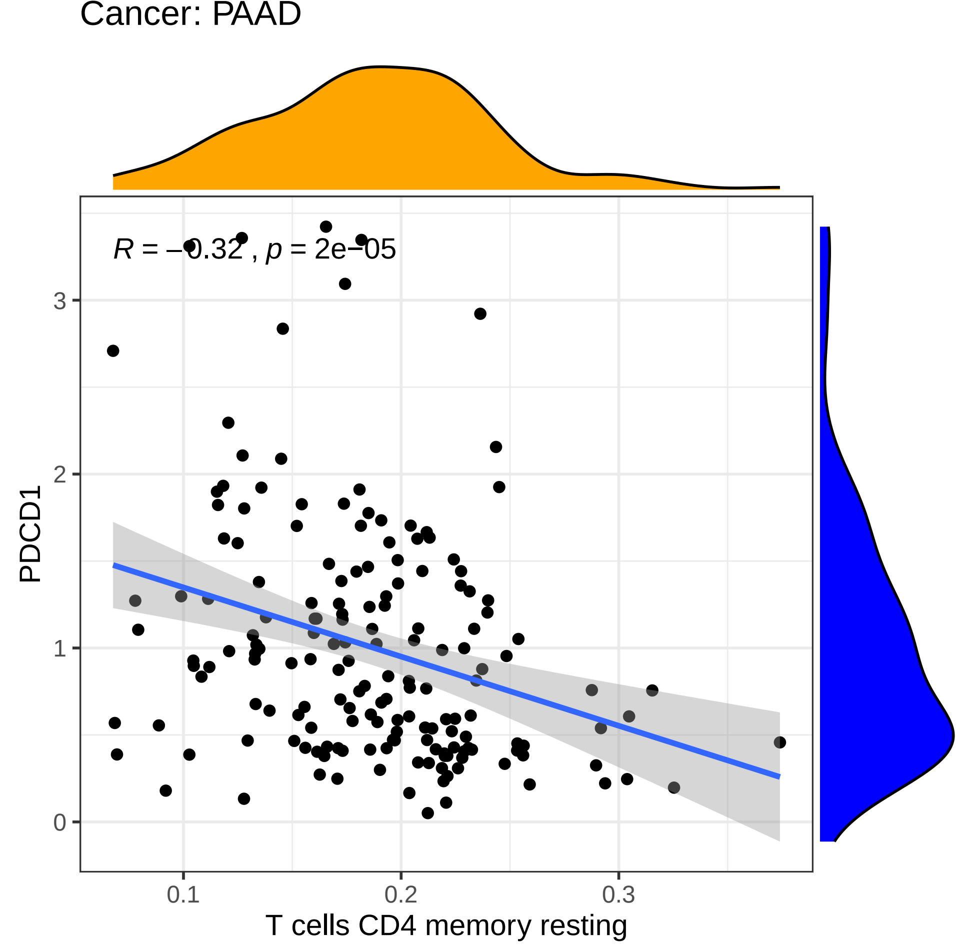

Cancer: PAAD
R = −0.32 , p = 2e−05
PDCD1
0
1
2
3
0.1
0.2
0.3
T cells CD4 memory resting

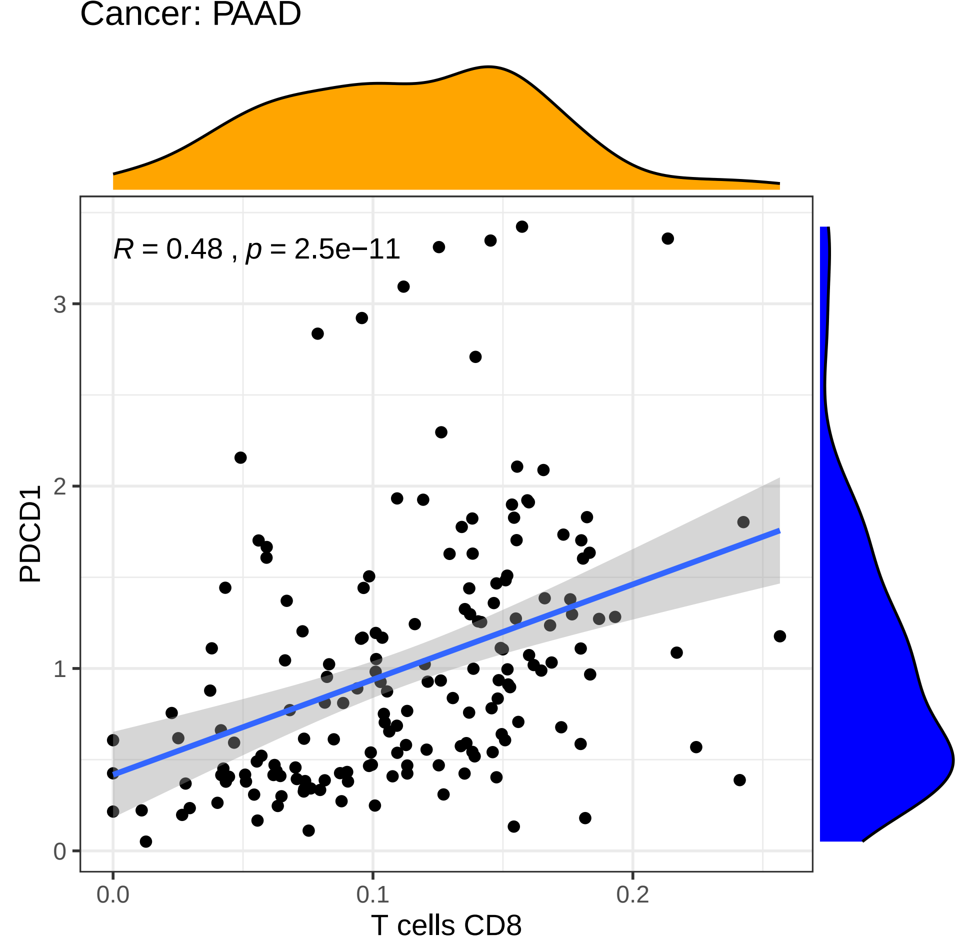

Cancer: PAAD
R = 0.48 , p = 2.5e−11
PDCD1
T cells CD8
0
1
2
3
0.0
0.1
0.2

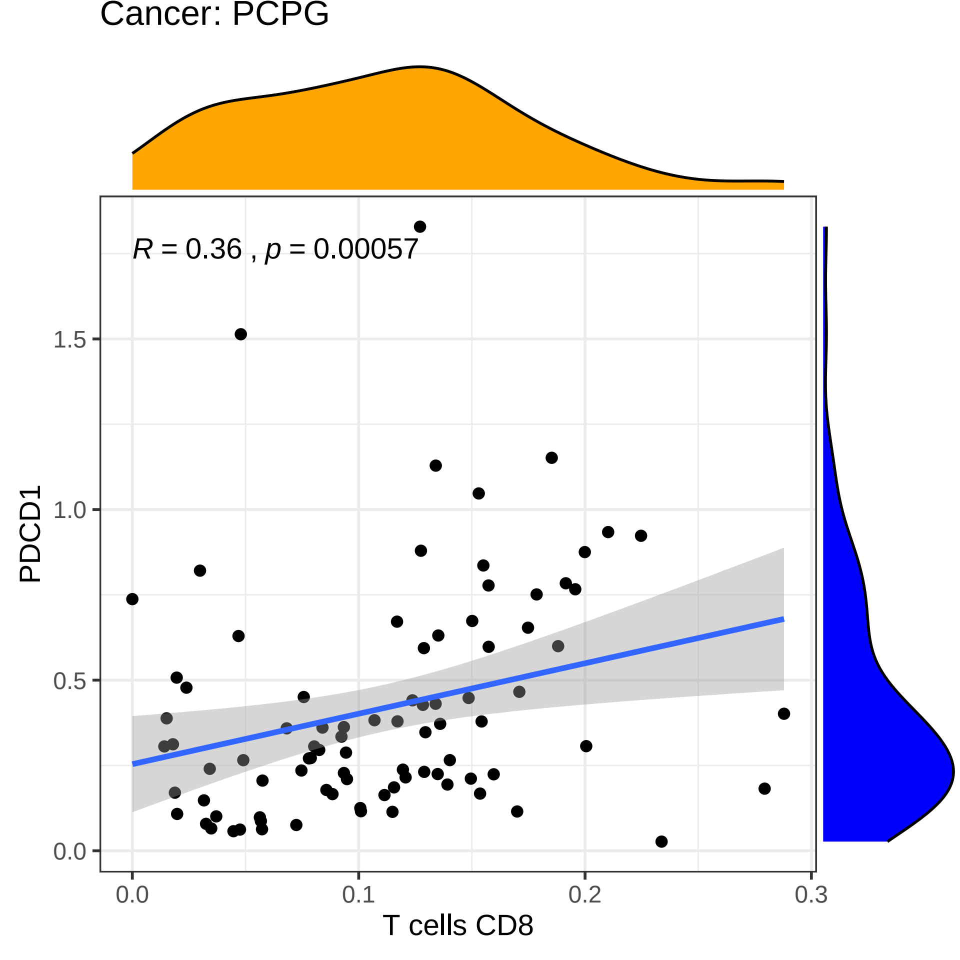

Cancer: PCPG
R = 0.36 , p = 0.00057
PDCD1
0.0
0.5
1.0
1.5
0.0
0.1
0.2
0.3
T cells CD8

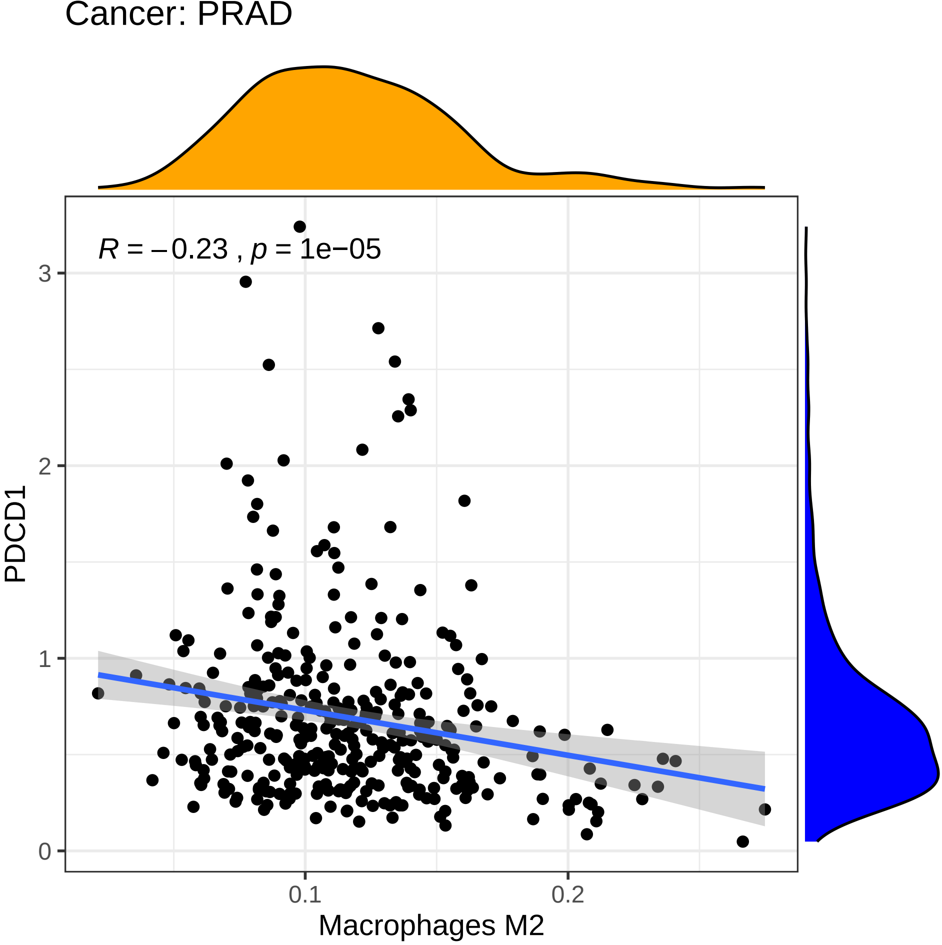

Cancer: PRAD
R = −0.23 , p = 1e−05
PDCD1
0
1
2
3
0.1
0.2
Macrophages M2

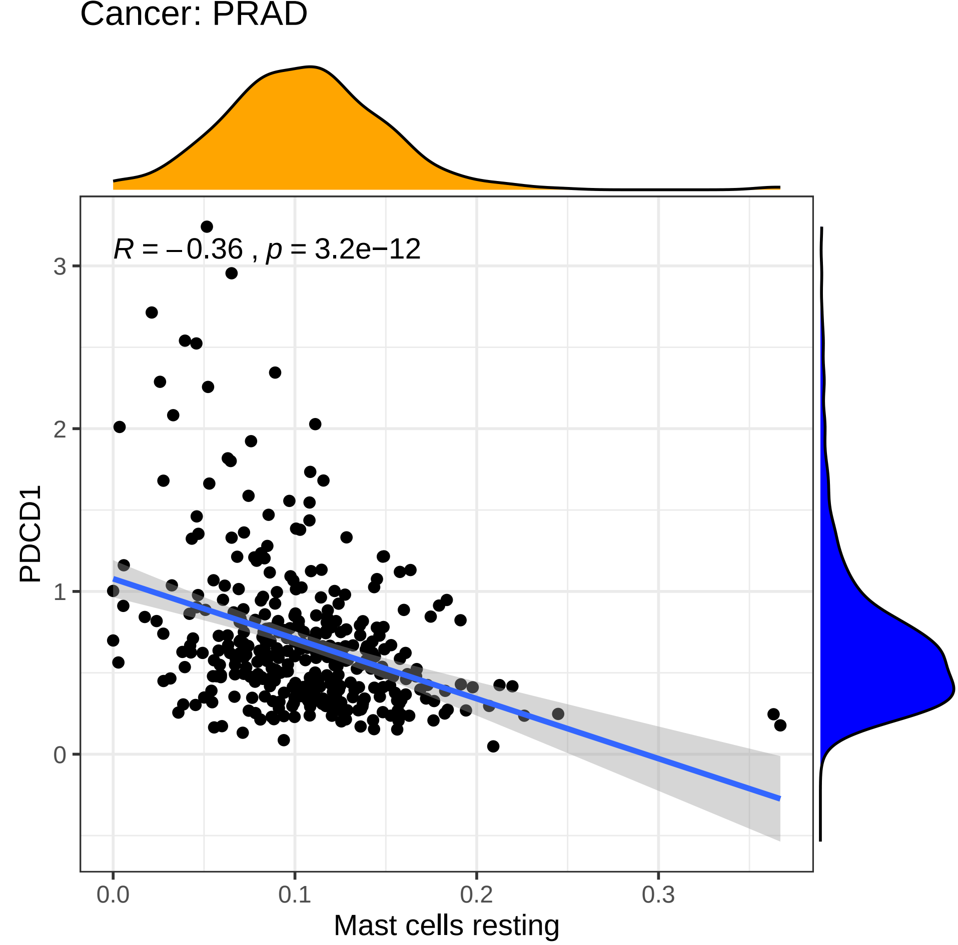

Cancer: PRAD
R = −0.36 , p = 3.2e−12
PDCD1
Mast cells resting
0.0
0.1
0.2
0.3
0
1
2
3

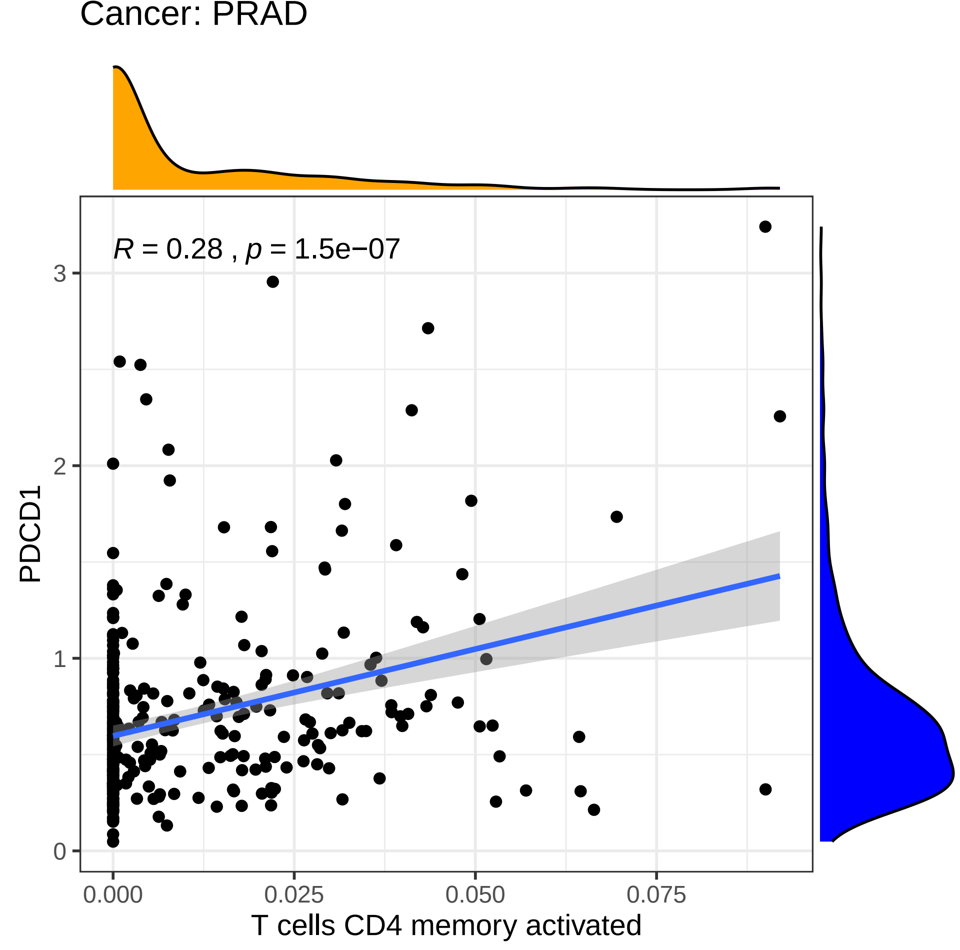

Cancer: PRAD
R = 0.28 , p = 1.5e−07
PDCD1
0
1
2
3
0.000
0.025
0.050
0.075
T cells CD4 memory activated

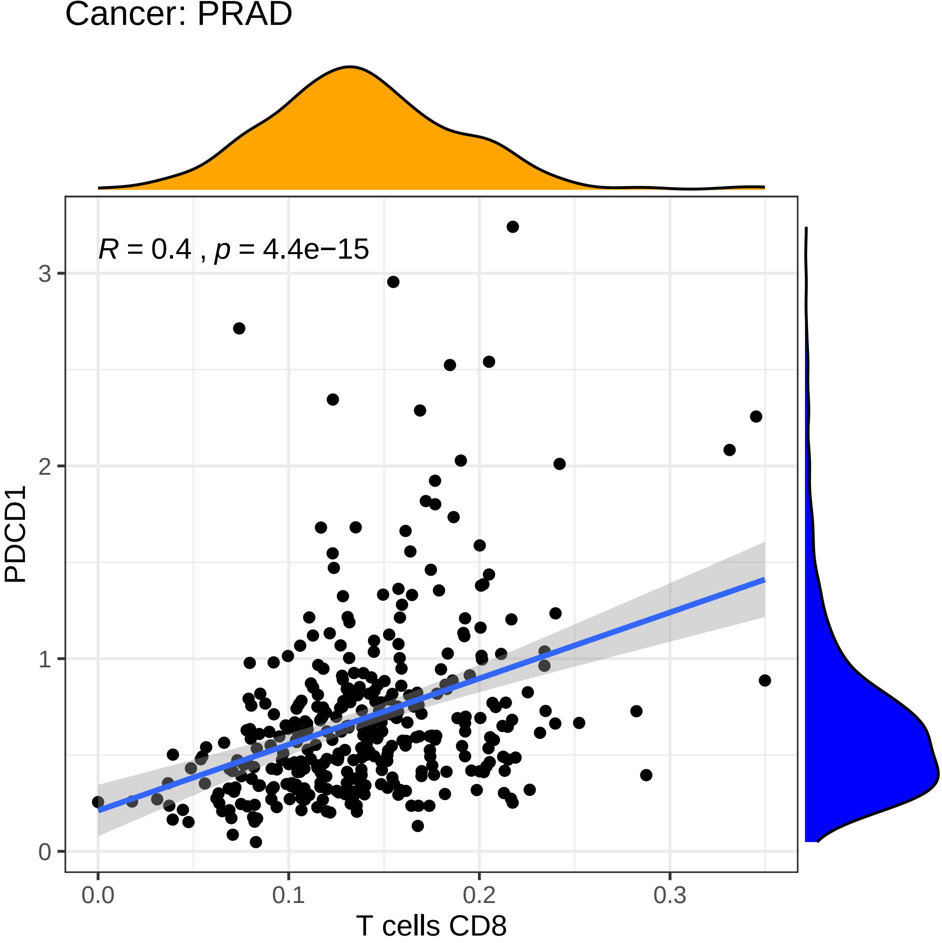
Cancer: PRAD
R = 0.4 , p = 4.4e−15
PDCD1
T cells CD8
0
1
2
3
0.0
0.1
0.2
0.3

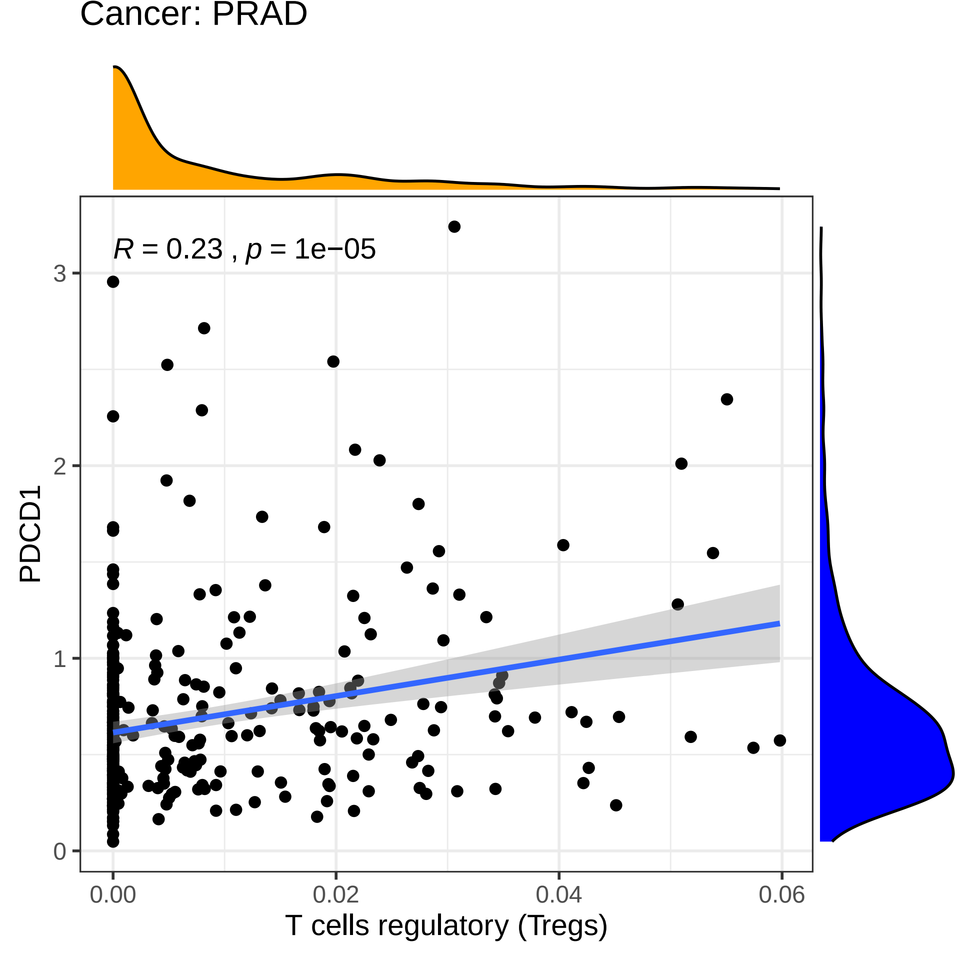

Cancer: PRAD
R = 0.23 , p = 1e−05
PDCD1
0
1
2
3
0.00
0.02
0.04
0.06
T cells regulatory (Tregs)

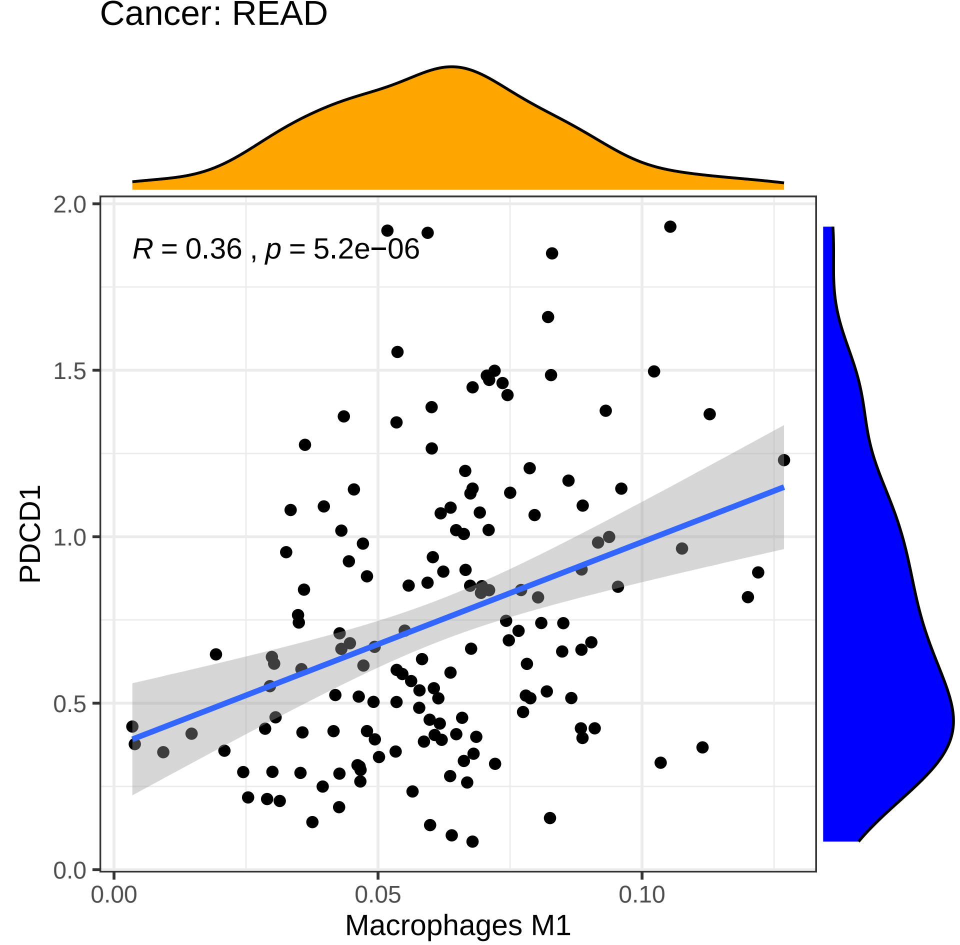

Cancer: READ
R = 0.36 , p = 5.2e−06
2.0
1.5
1.0
0.5
0.0
0.00
0.05
0.10
PDCD1
Macrophages M1

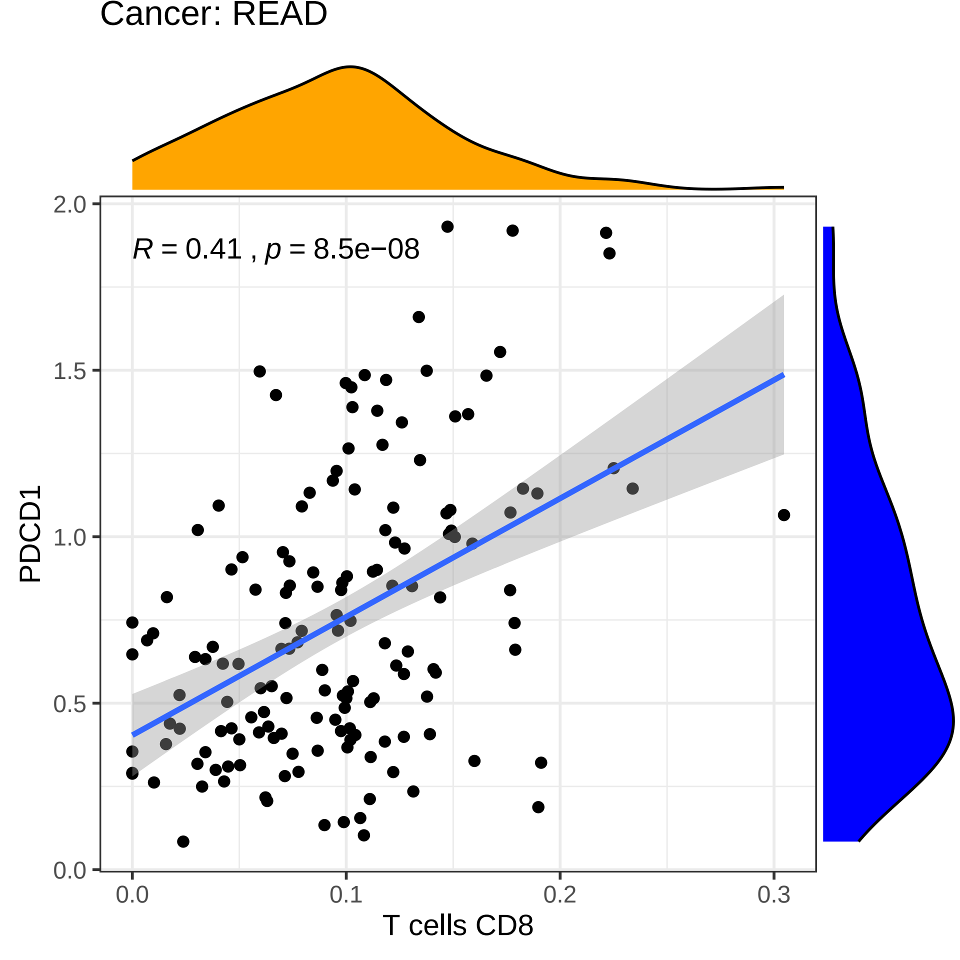
Cancer: READ
R = 0.41 , p = 8.5e−08
PDCD1
T cells CD8
0.0
0.5
1.0
1.5
2.0
0.0
0.1
0.2
0.3

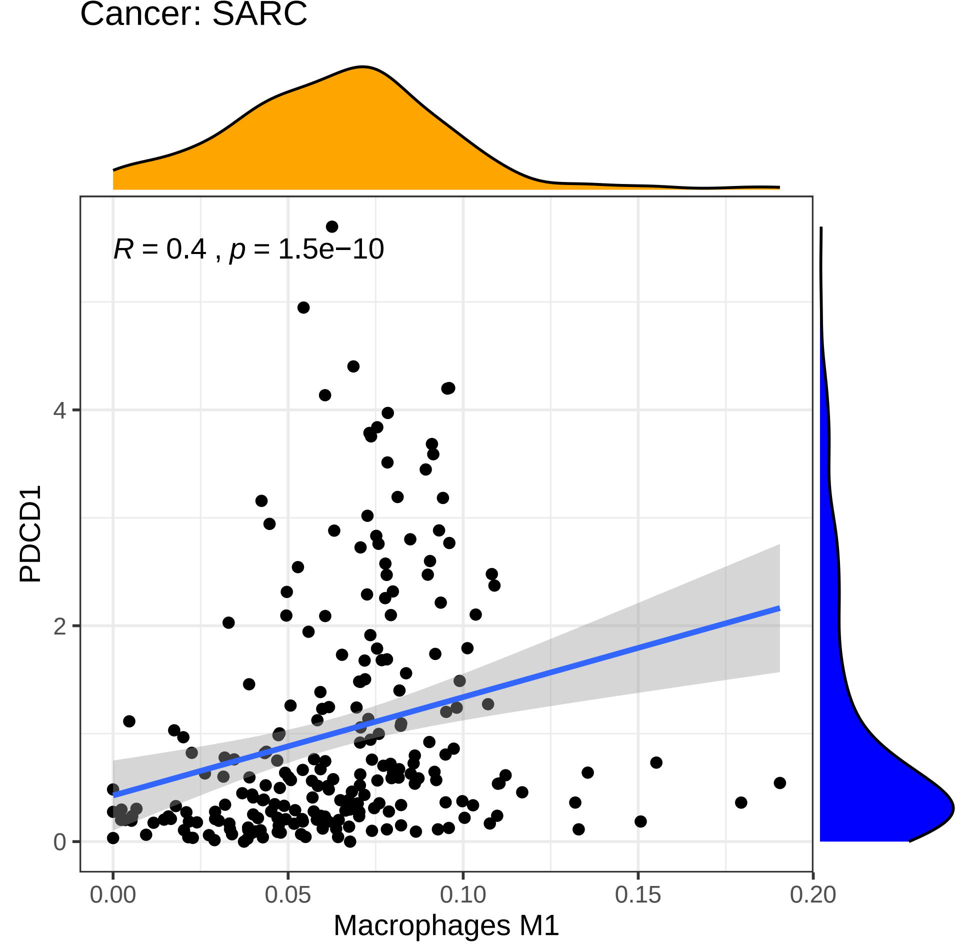

Cancer: SARC
R = 0.4 , p = 1.5e−10
PDCD1
0
2
4
0.00
0.05
0.10
0.15
0.20
Macrophages M1

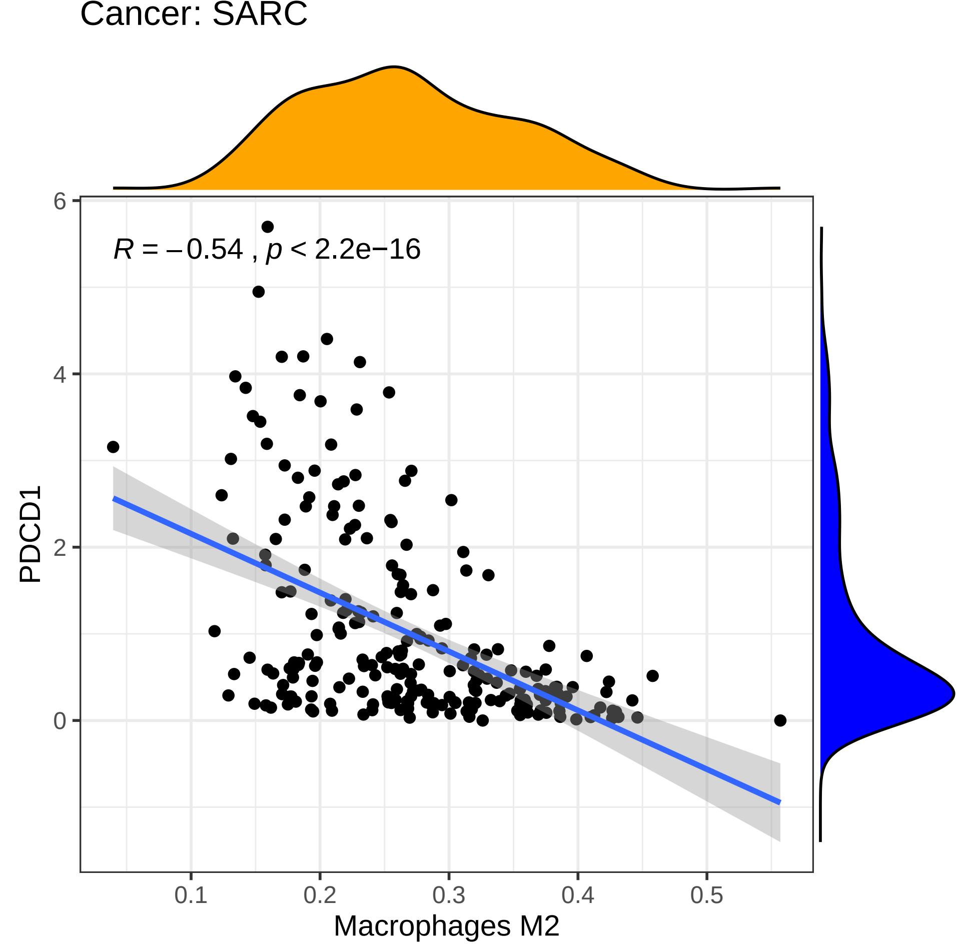
Cancer: SARC
R = −0.54 , p < 2.2e−16
PDCD1
Macrophages M2
0
2
4
6
0.1
0.2
0.3
0.4
0.5

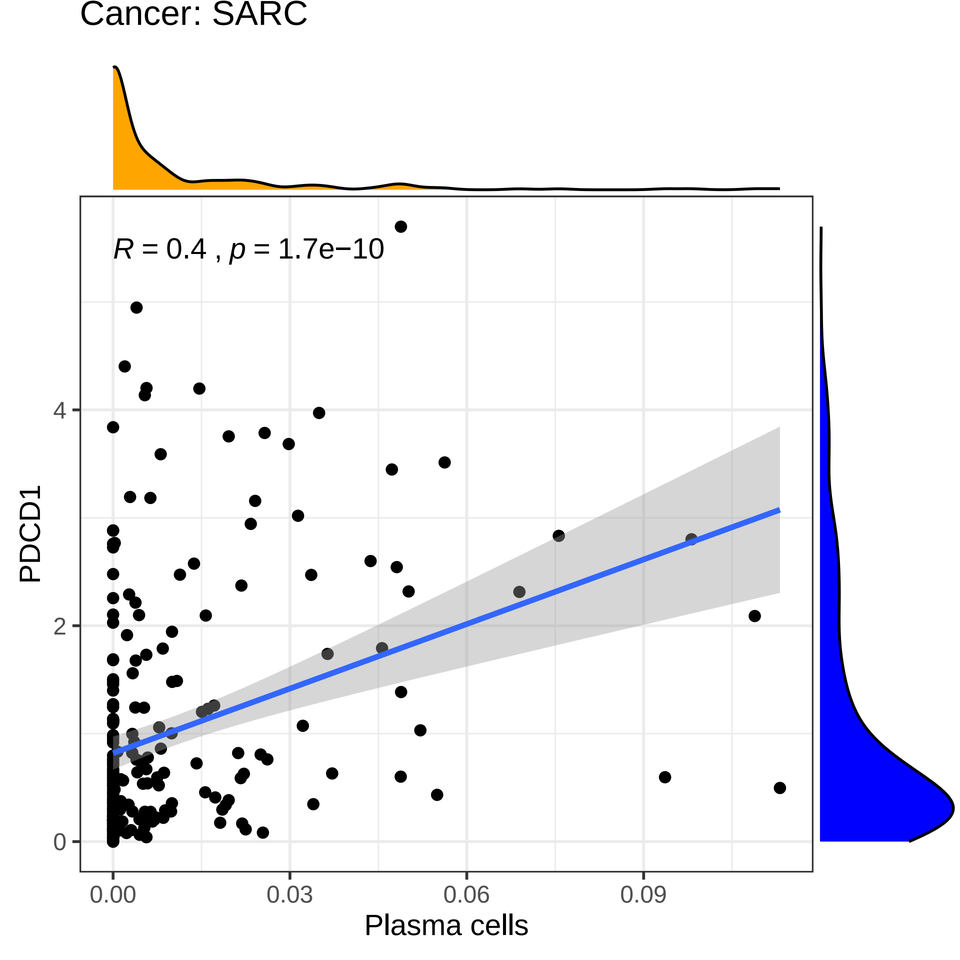
Cancer: SARC
R = 0.4 , p = 1.7e−10
PDCD1
Plasma cells
0
2
4
0.00
0.03
0.06
0.09

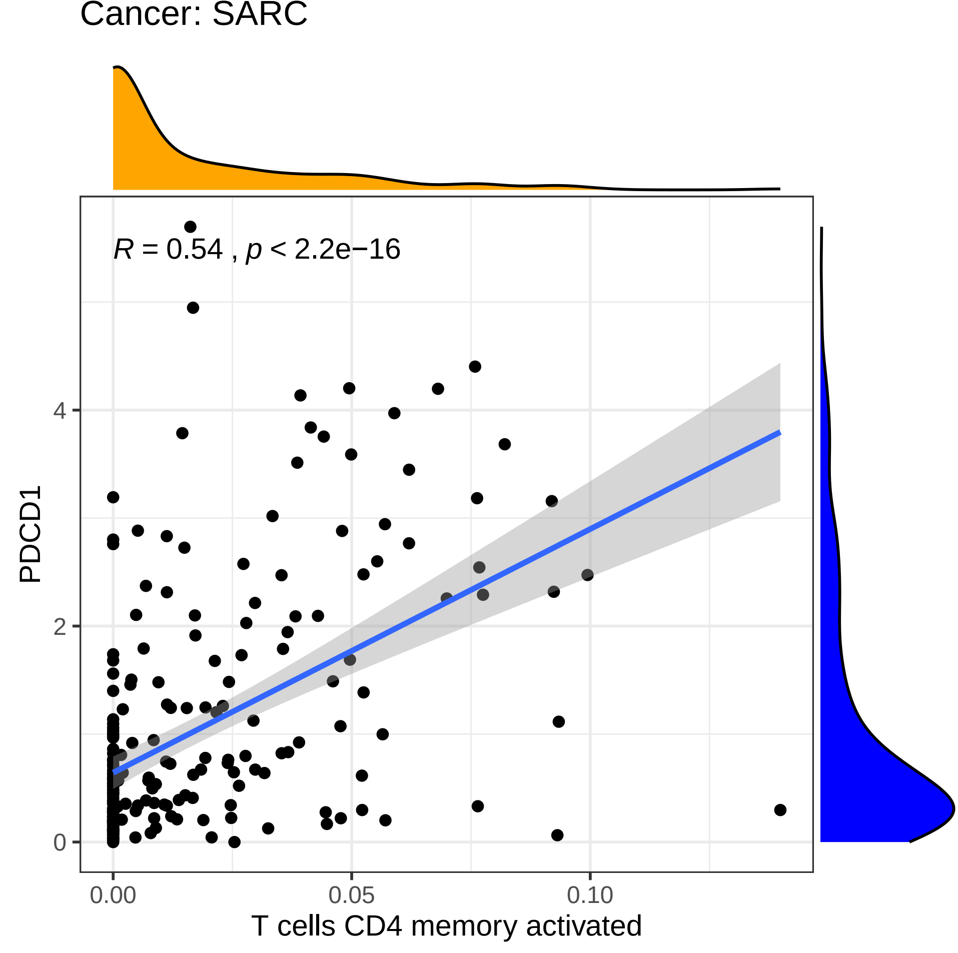

Cancer: SARC
R = 0.54 , p < 2.2e−16
PDCD1
0
2
4
0.00
0.05
0.10
T cells CD4 memory activated

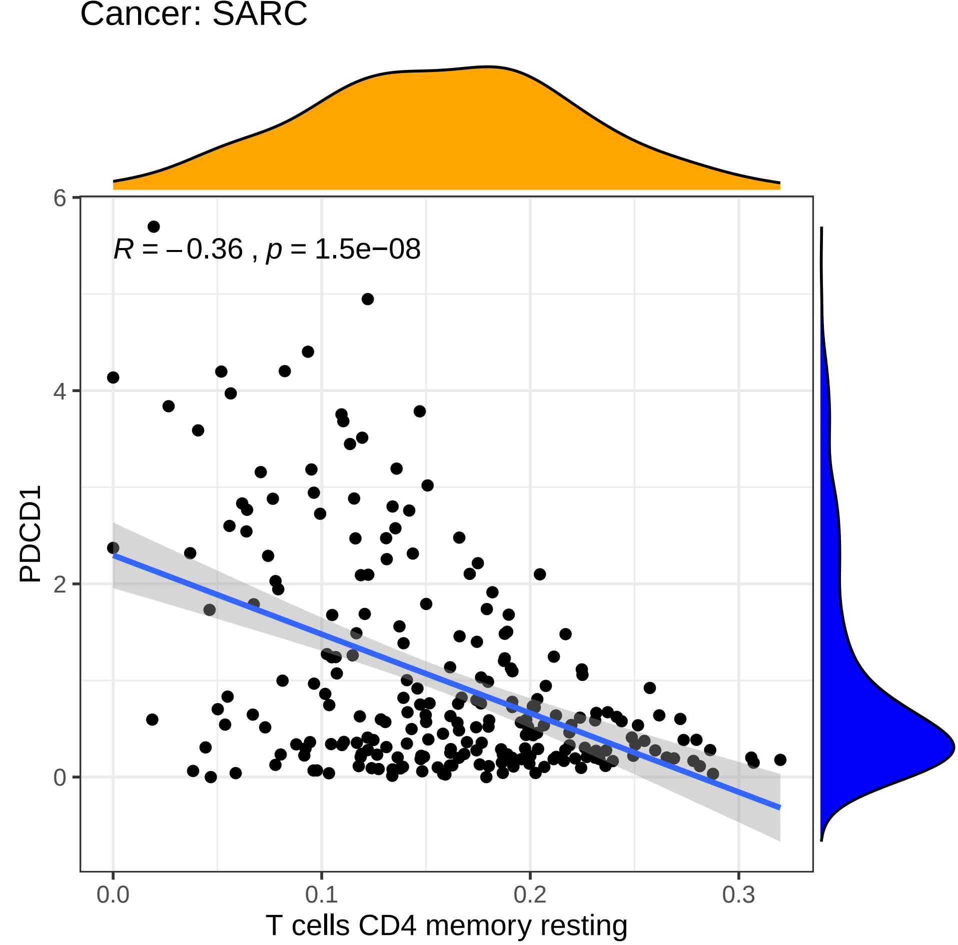

Cancer: SARC
R = −0.36 , p = 1.5e−08
PDCD1
6
4
2
0
0.0
0.1
0.2
0.3
T cells CD4 memory resting

Cancer: SARC

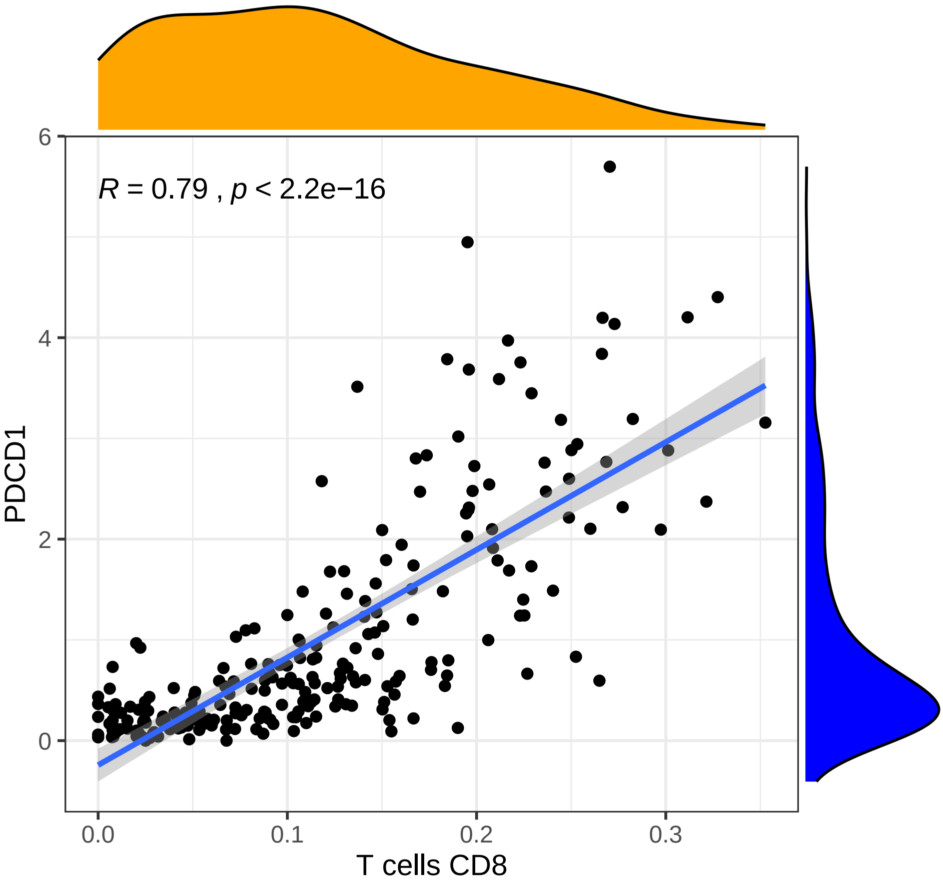

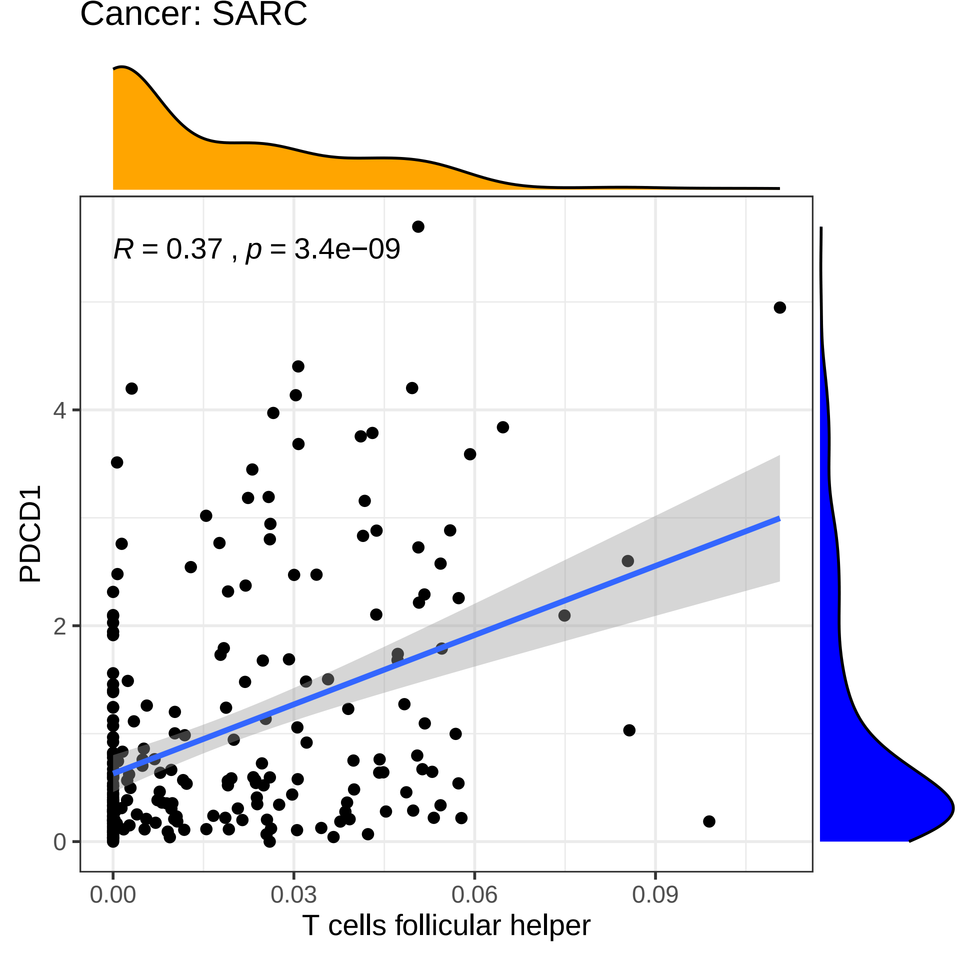
Cancer: SARC
R = 0.37 , p = 3.4e−09
PDCD1
0
2
4
0.00
0.03
0.06
0.09
T cells follicular helper

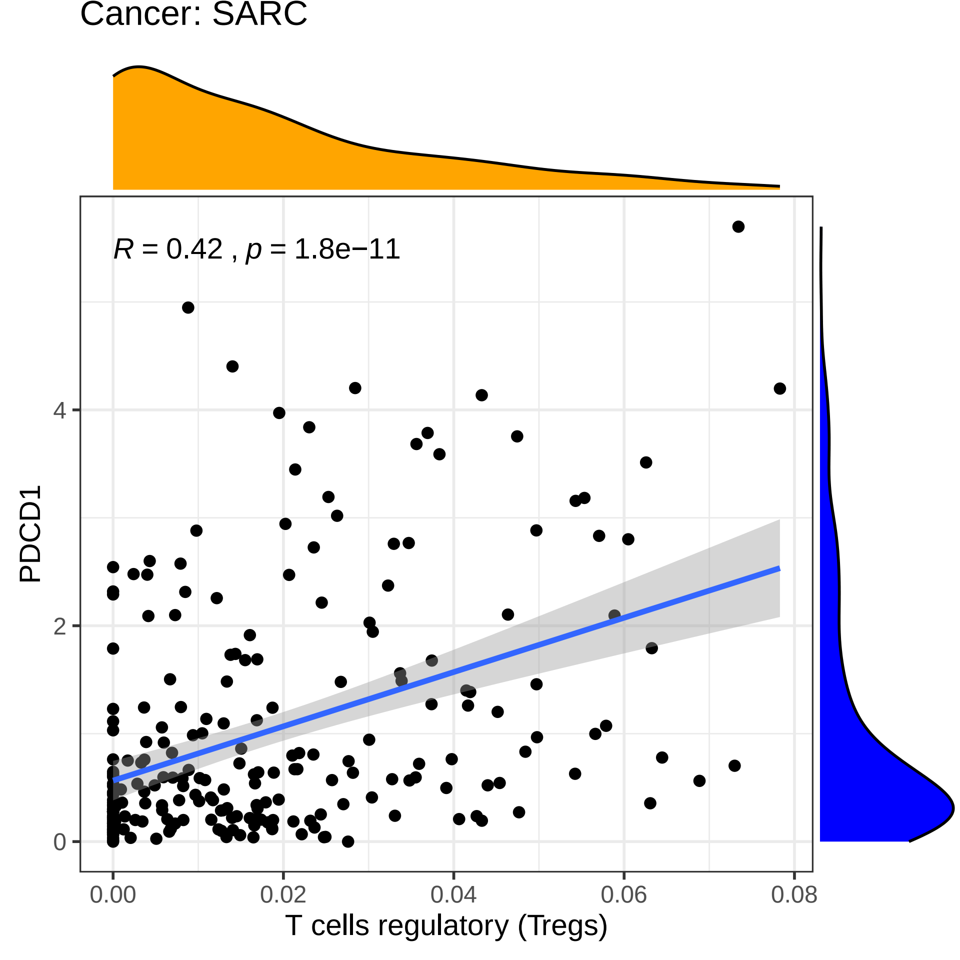
Cancer: SARC
R = 0.42 , p = 1.8e−11
PDCD1
0
2
4
0.00
0.02
0.04
0.06
0.08
T cells regulatory (Tregs)

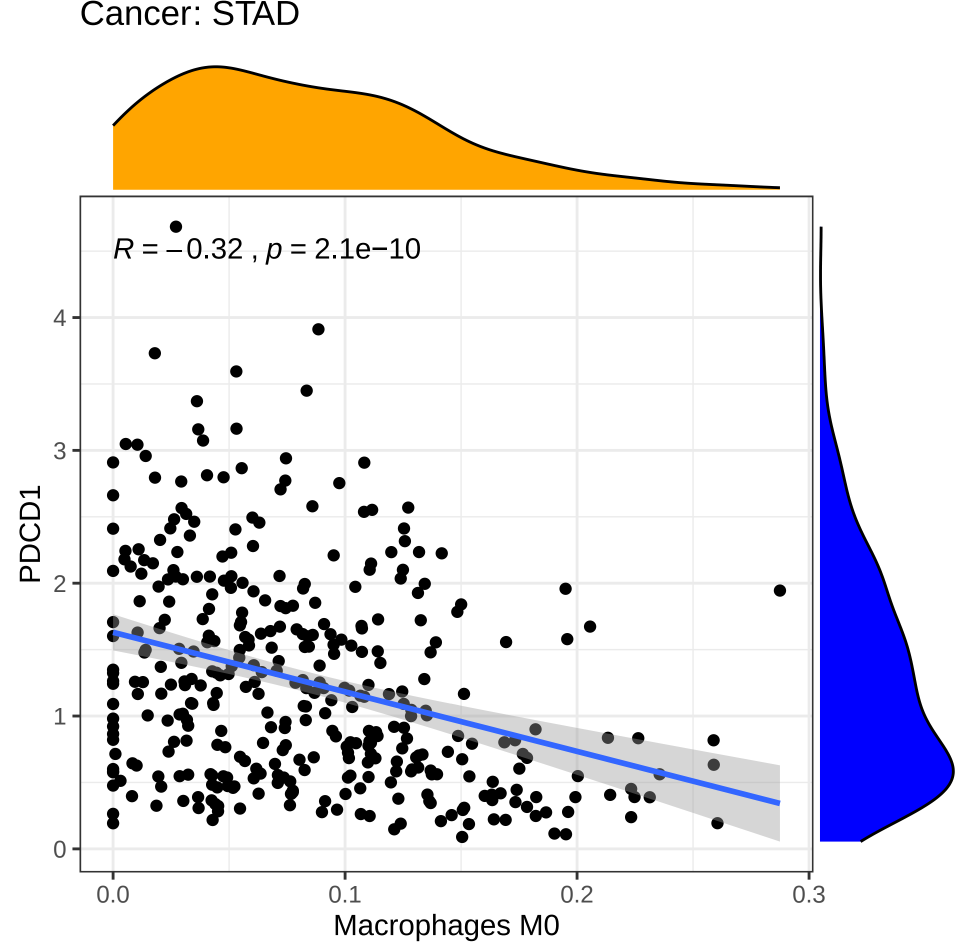

Cancer: STAD
R = −0.32 , p = 2.1e−10
PDCD1
0
1
2
3
4
0.0
0.1
0.2
0.3
Macrophages M0

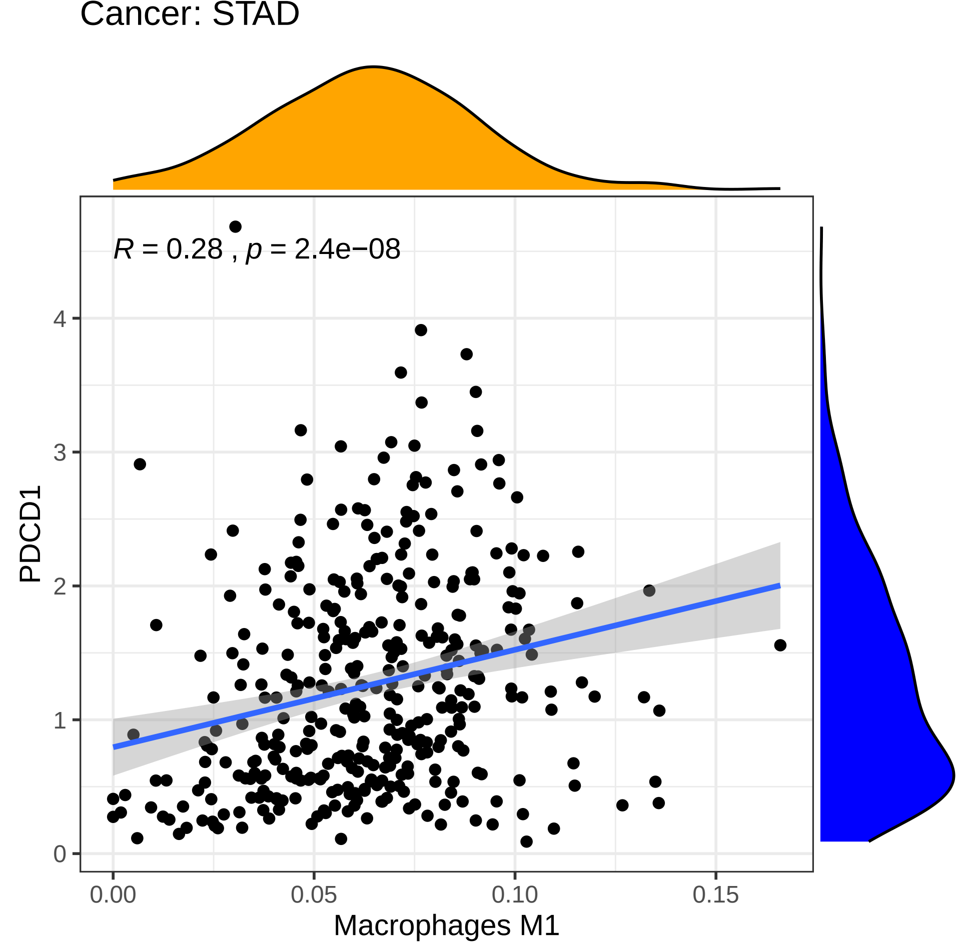

Cancer: STAD
R = 0.28 , p = 2.4e−08
PDCD1
0
1
2
3
4
0.00
0.05
0.10
0.15
Macrophages M1

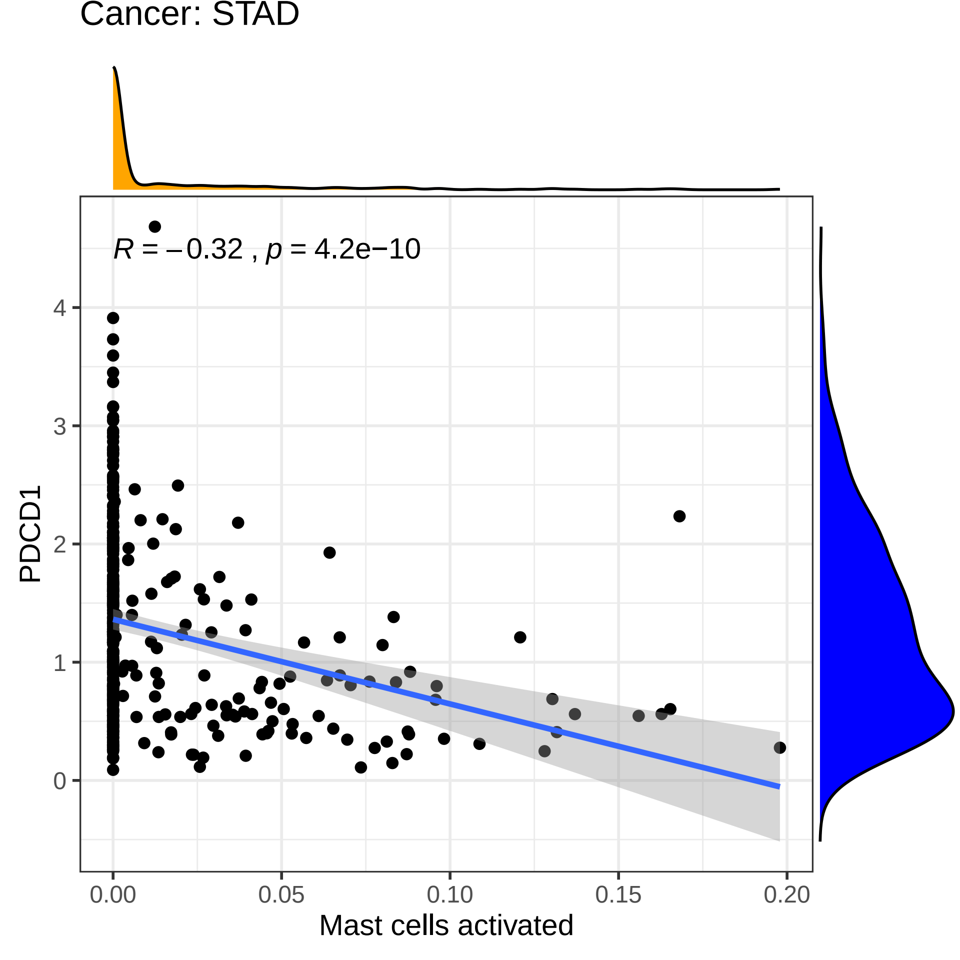

Cancer: STAD
R = −0.32 , p = 4.2e−10
PDCD1
0
1
2
3
4
0.00
0.05
0.10
0.15
0.20
Mast cells activated

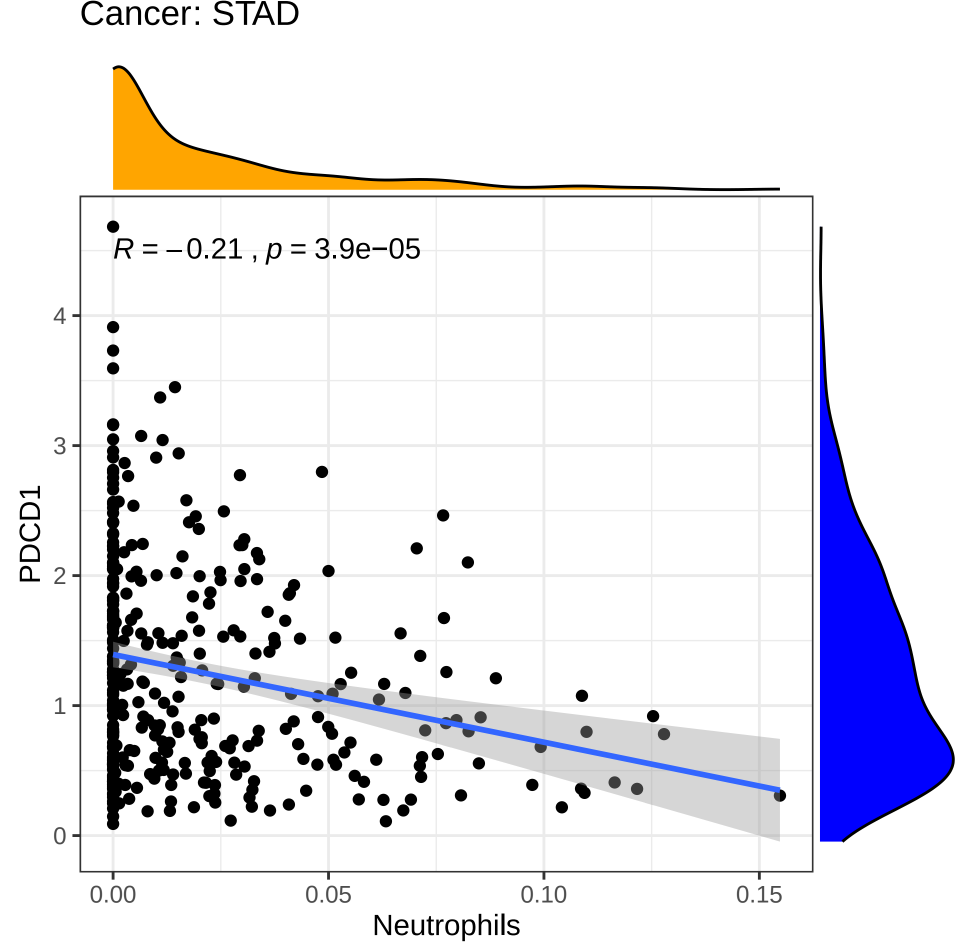

Cancer: STAD
R = −0.21 , p = 3.9e−05
PDCD1
0
1
2
3
4
0.00
0.05
0.10
0.15
Neutrophils

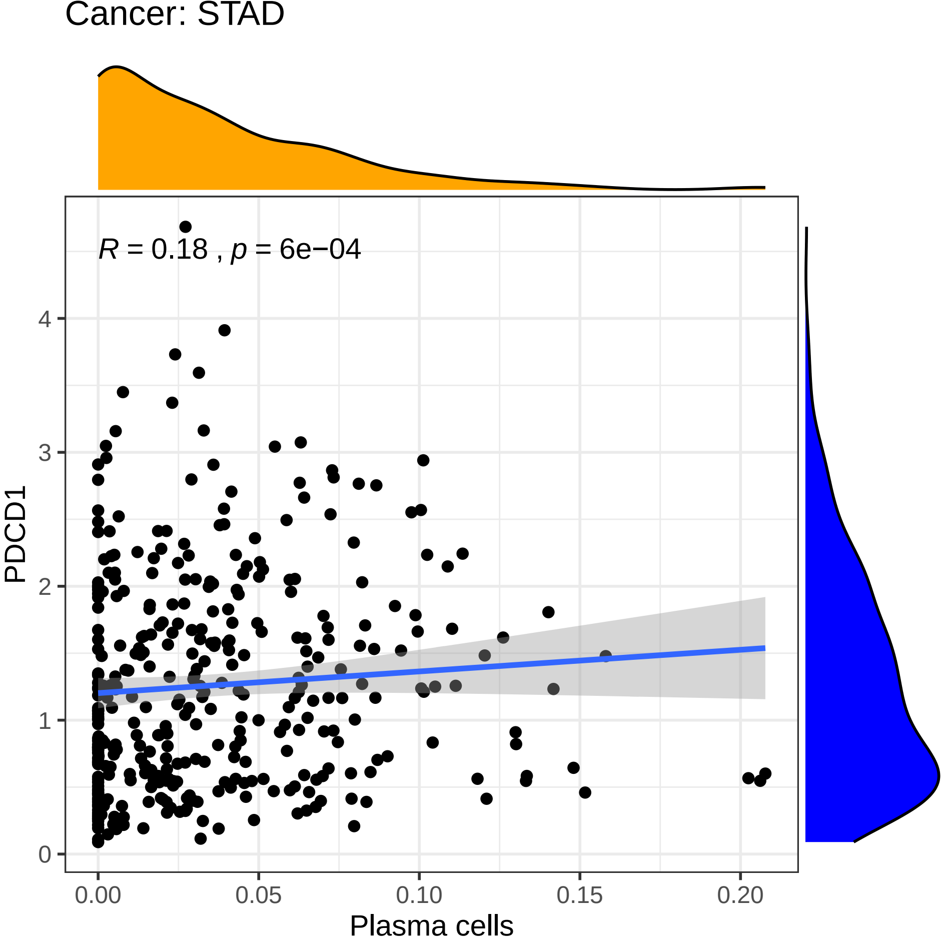
Cancer: STAD
R = 0.18 , p = 6e−04
PDCD1
0
1
2
3
4
0.00
0.05
0.10
0.15
0.20
Plasma cells

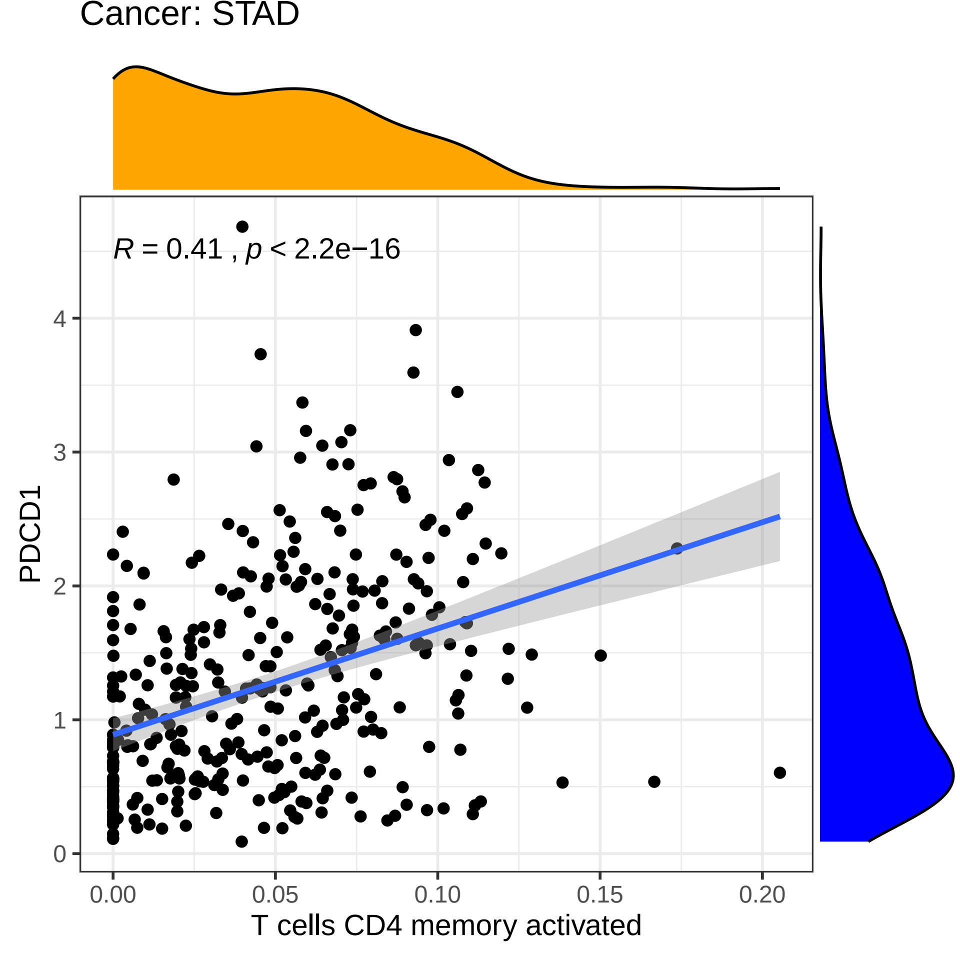
Cancer: STAD
R = 0.41 , p < 2.2e−16
PDCD1
0
1
2
3
4
0.00
0.05
0.10
0.15
0.20
T cells CD4 memory activated

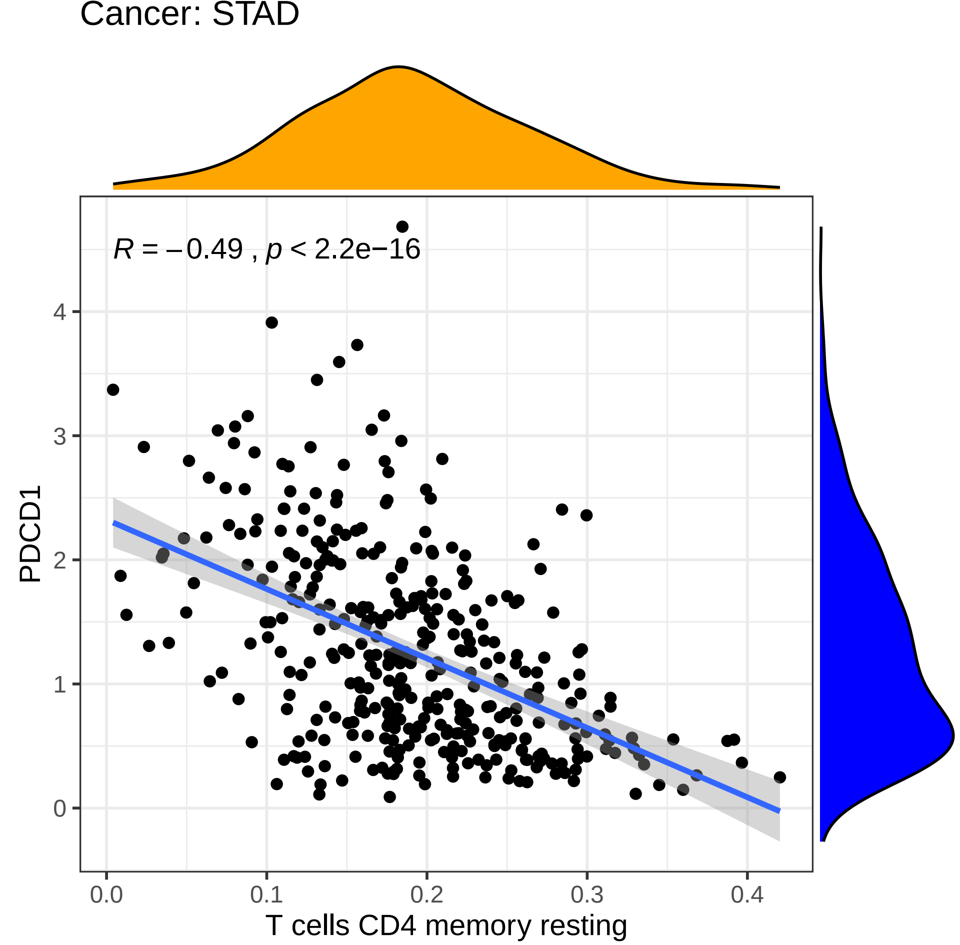

Cancer: STAD
R = −0.49 , p < 2.2e−16
PDCD1
0
1
2
3
4
0.0
0.1
0.2
0.3
0.4
T cells CD4 memory resting

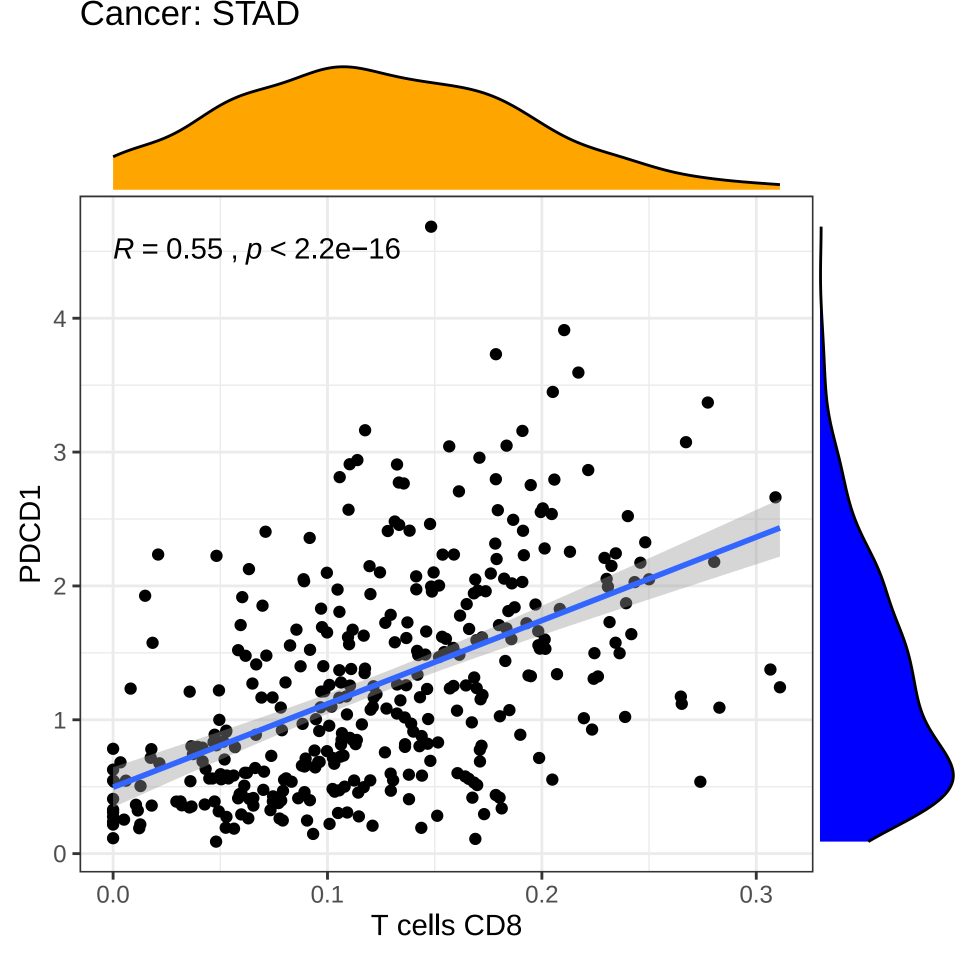

Cancer: STAD
R = 0.55 , p < 2.2e−16
PDCD1
0
1
2
3
4
0.0
0.1
0.2
0.3
T cells CD8

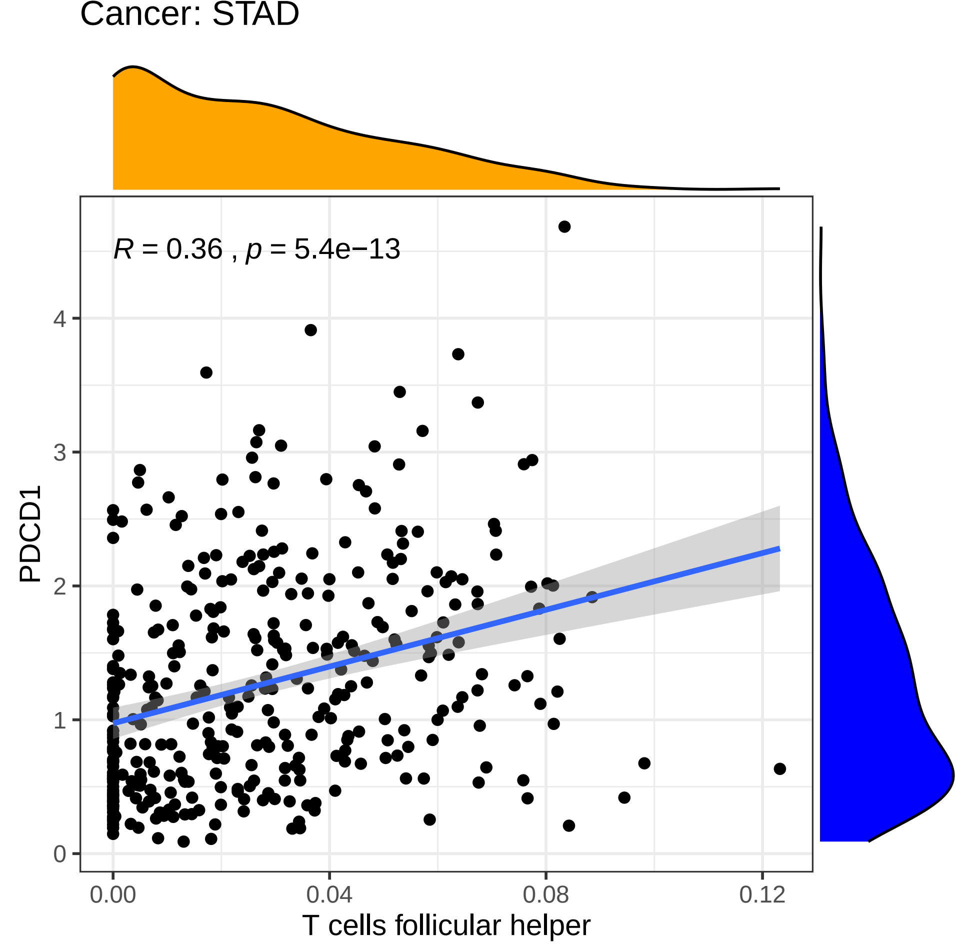

Cancer: STAD
R = 0.36 , p = 5.4e−13
PDCD1
0
1
2
3
4
0.00
0.04
0.08
0.12
T cells follicular helper

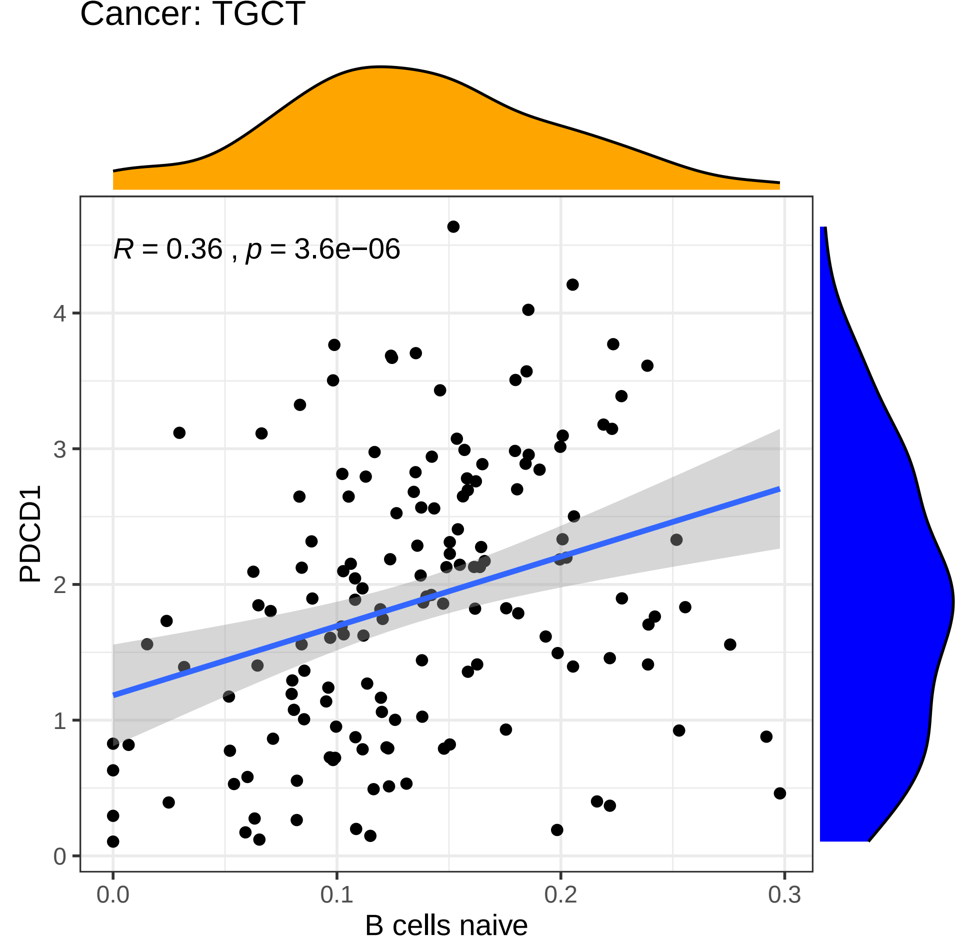
Cancer: TGCT
R = 0.36 , p = 3.6e−06
PDCD1
0
1
2
3
4
0.0
0.1
0.2
0.3
B cells naive

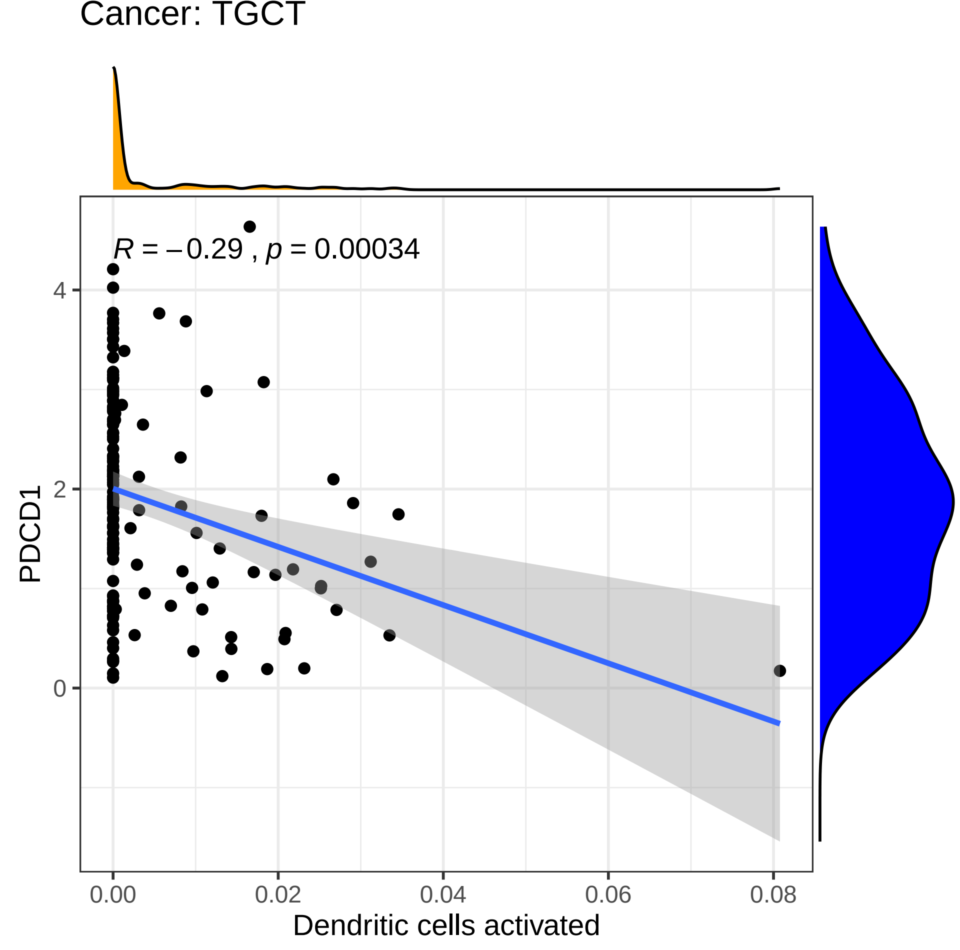

Cancer: TGCT
R = −0.29 , p = 0.00034
PDCD1
0
2
4
0.00
0.02
0.04
0.06
0.08
Dendritic cells activated

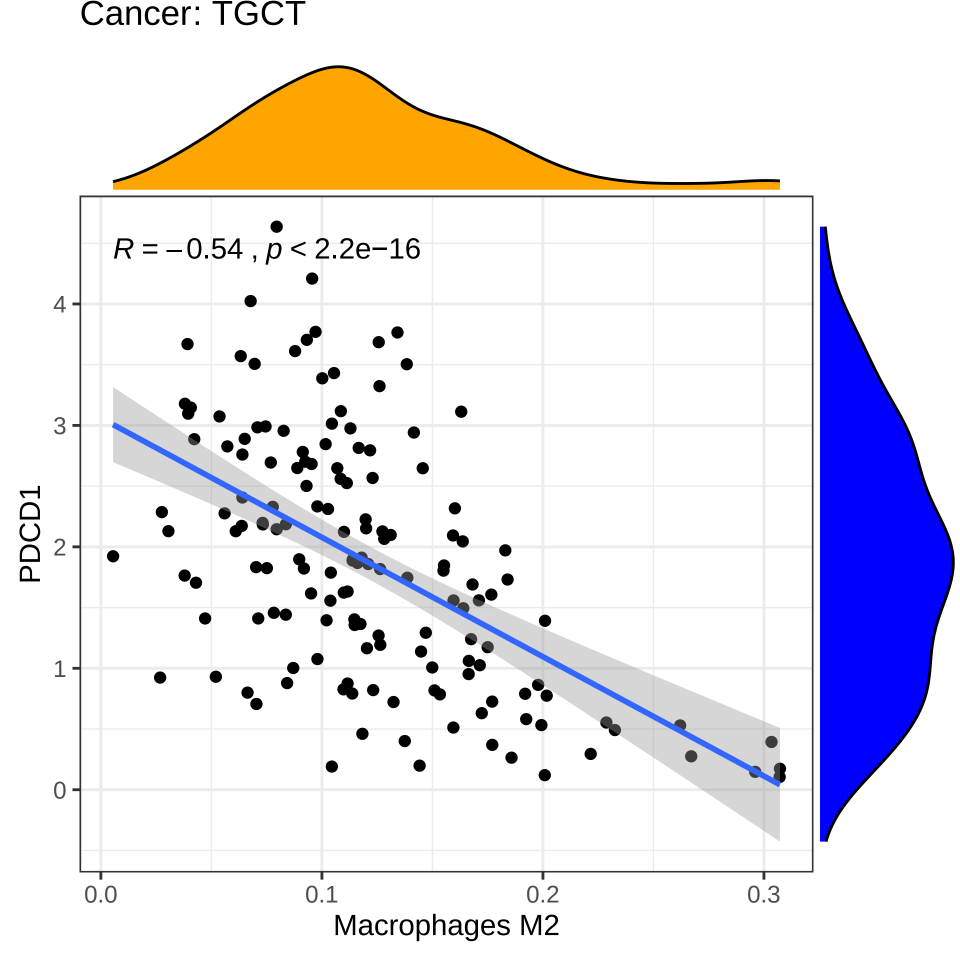

Cancer: TGCT
R = −0.54 , p < 2.2e−16
PDCD1
0
1
2
3
4
0.0
0.1
0.2
0.3
Macrophages M2

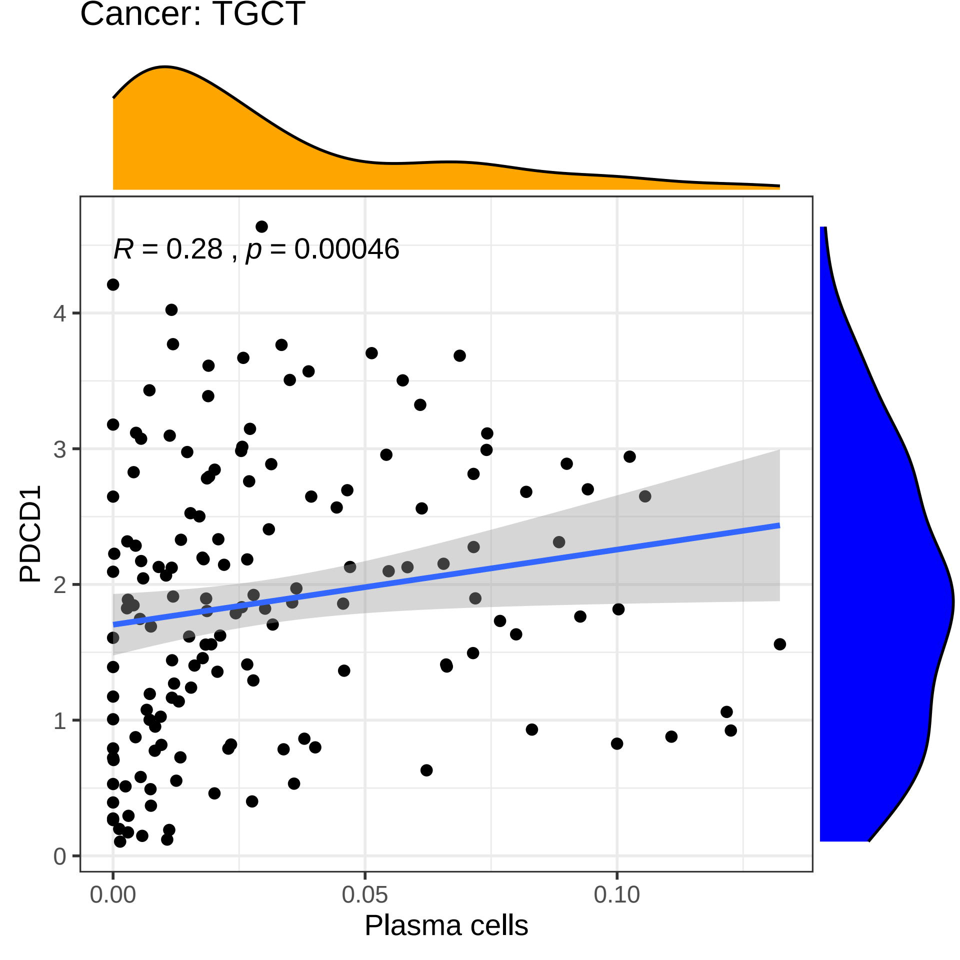

Cancer: TGCT
R = 0.28 , p = 0.00046
PDCD1
0
1
2
3
4
0.00
0.05
0.10
Plasma cells

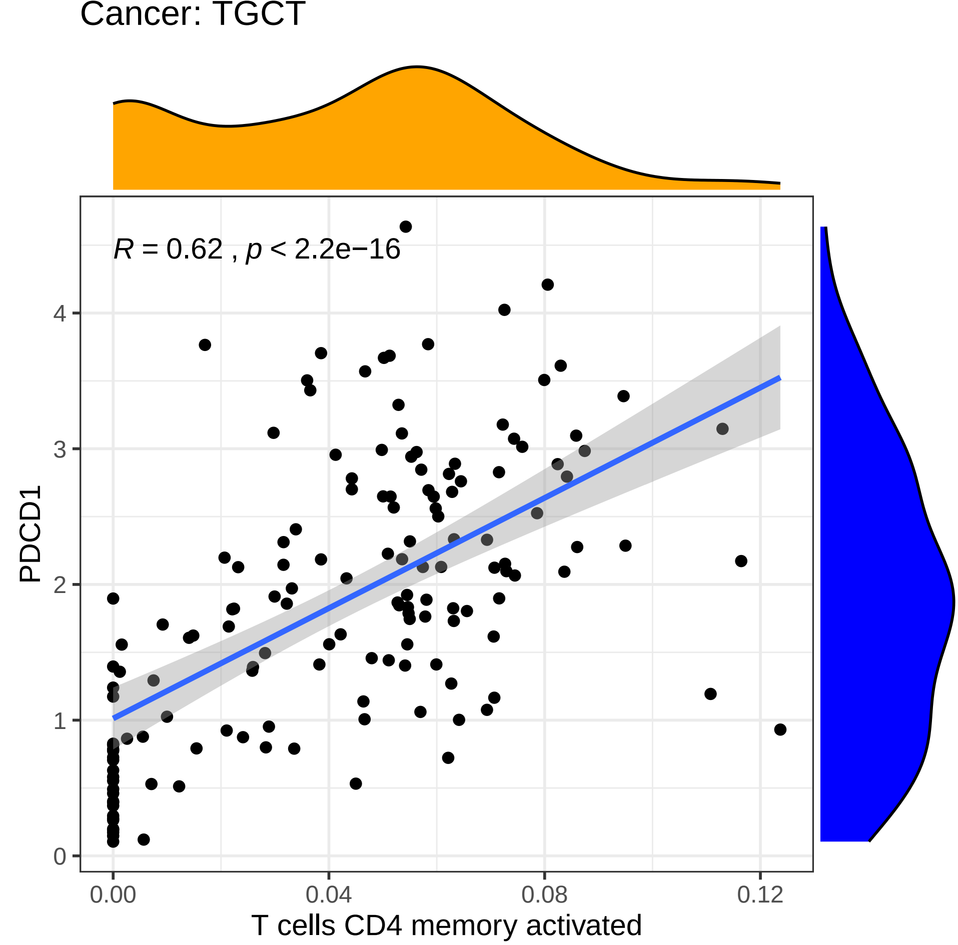

Cancer: TGCT
R = 0.62 , p < 2.2e−16
PDCD1
0
1
2
3
4
0.00
0.04
0.08
0.12
T cells CD4 memory activated

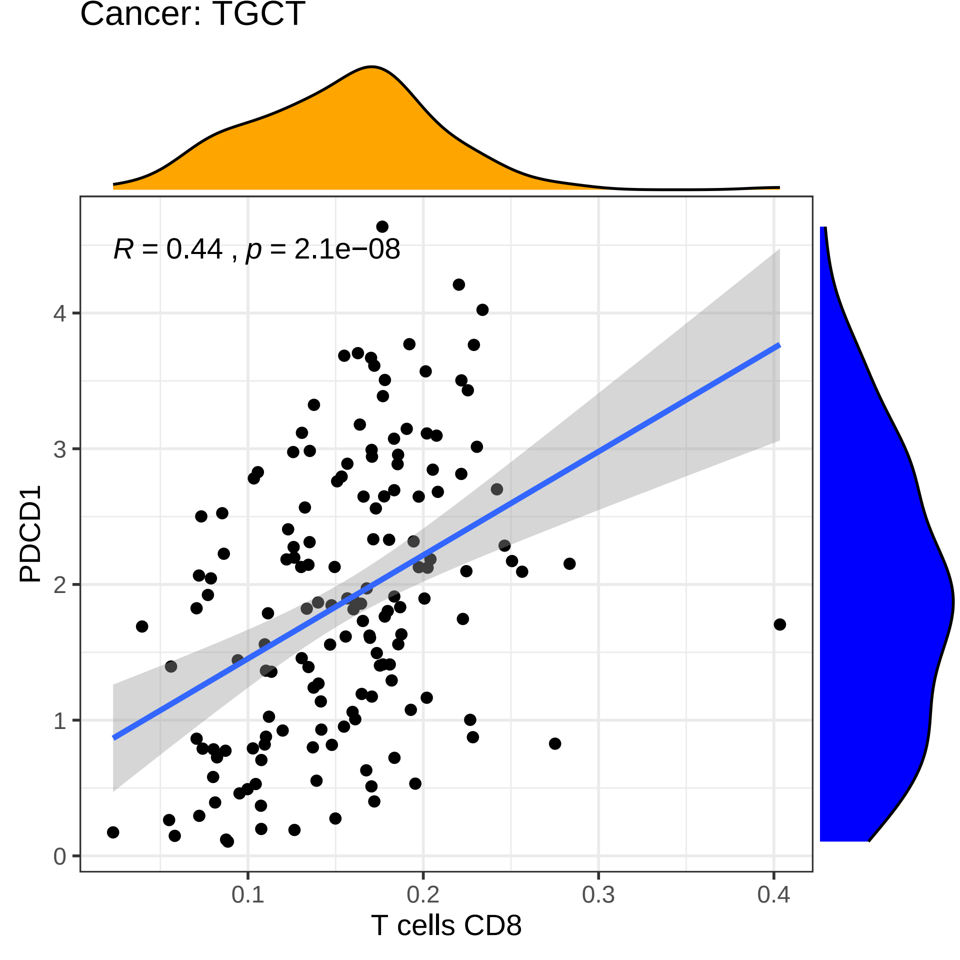
Cancer: TGCT
R = 0.44 , p = 2.1e−08
PDCD1
0
1
2
3
4
0.1
0.2
0.3
0.4
T cells CD8

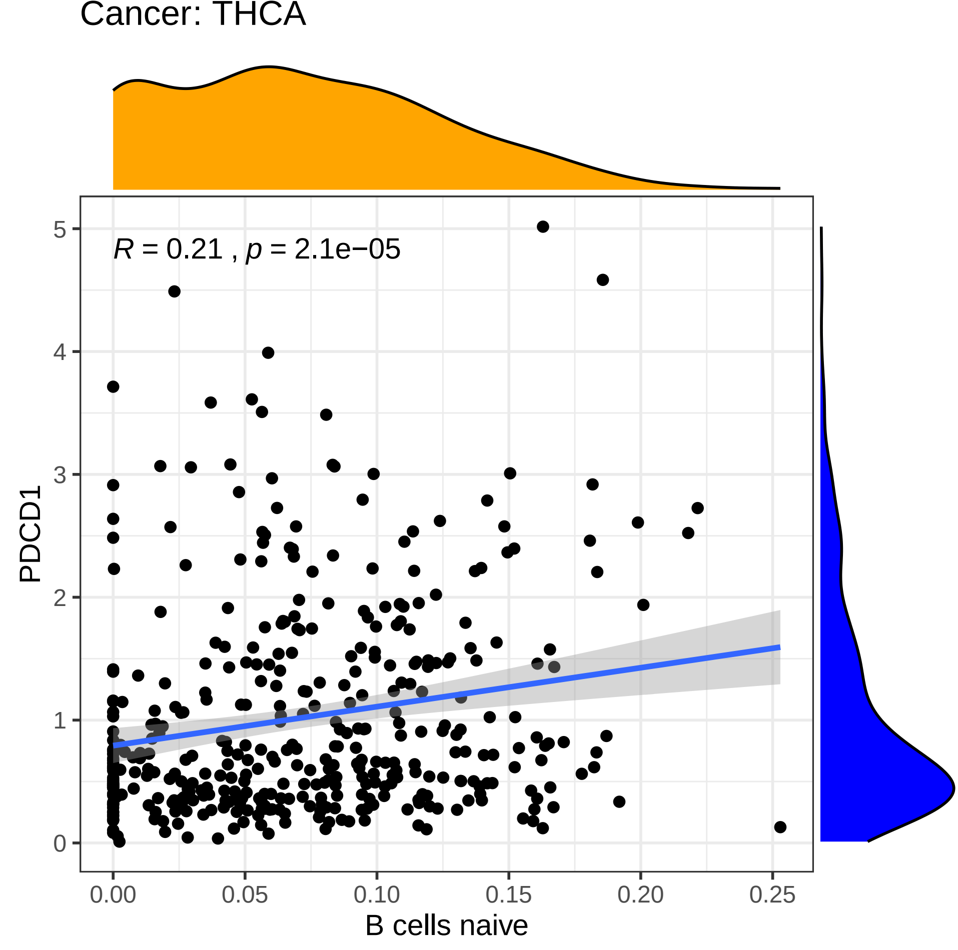

Cancer: THCA
R = 0.21 , p = 2.1e−05
PDCD1
B cells naive
0
1
2
3
4
5
0.00
0.05
0.10
0.15
0.20
0.25

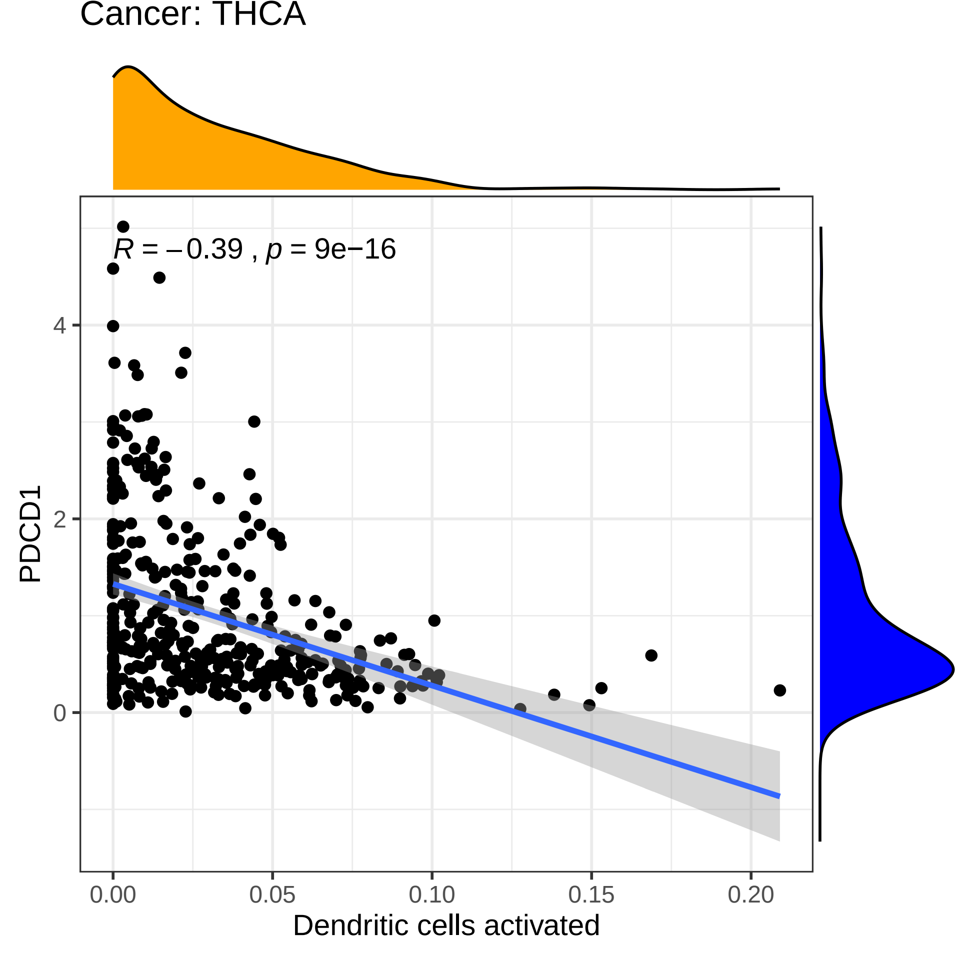
Cancer: THCA
R = −0.39 , p = 9e−16
PDCD1
4
2
0
0.00
0.05
0.10
0.15
0.20
Dendritic cells activated

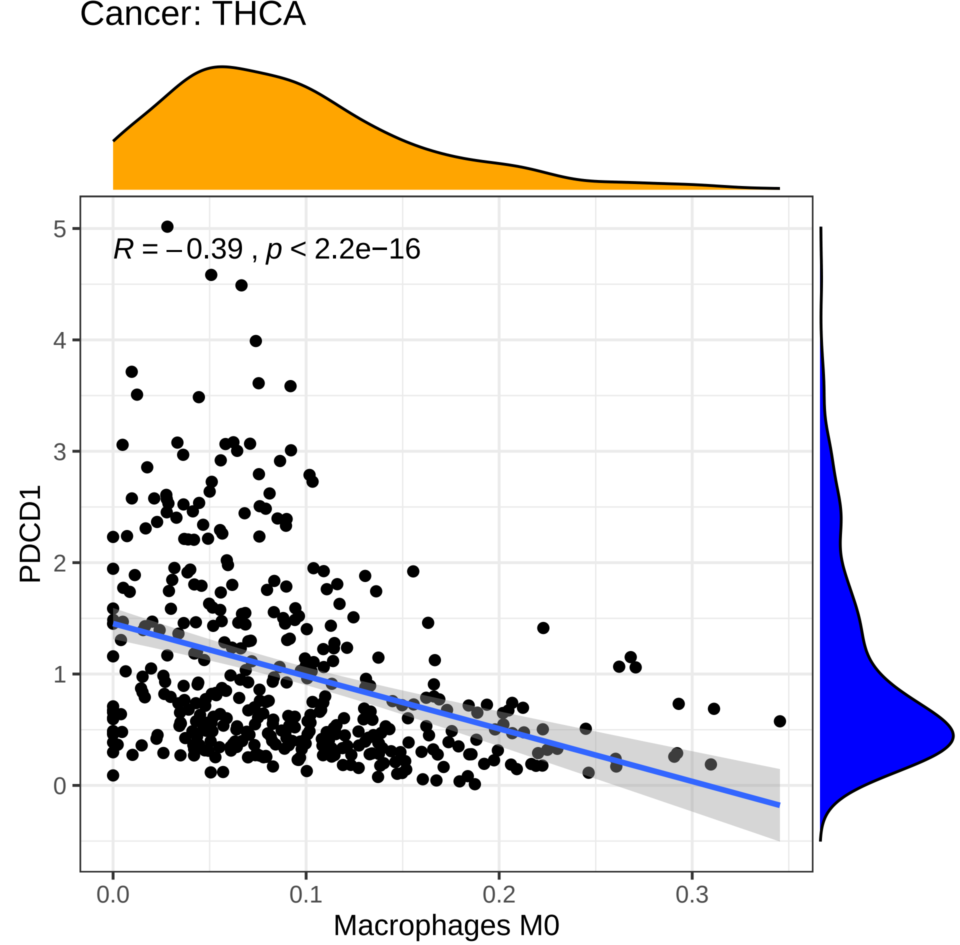

Cancer: THCA
R = −0.39 , p < 2.2e−16
PDCD1
Macrophages M0
0
1
2
3
4
5
0.0
0.1
0.2
0.3

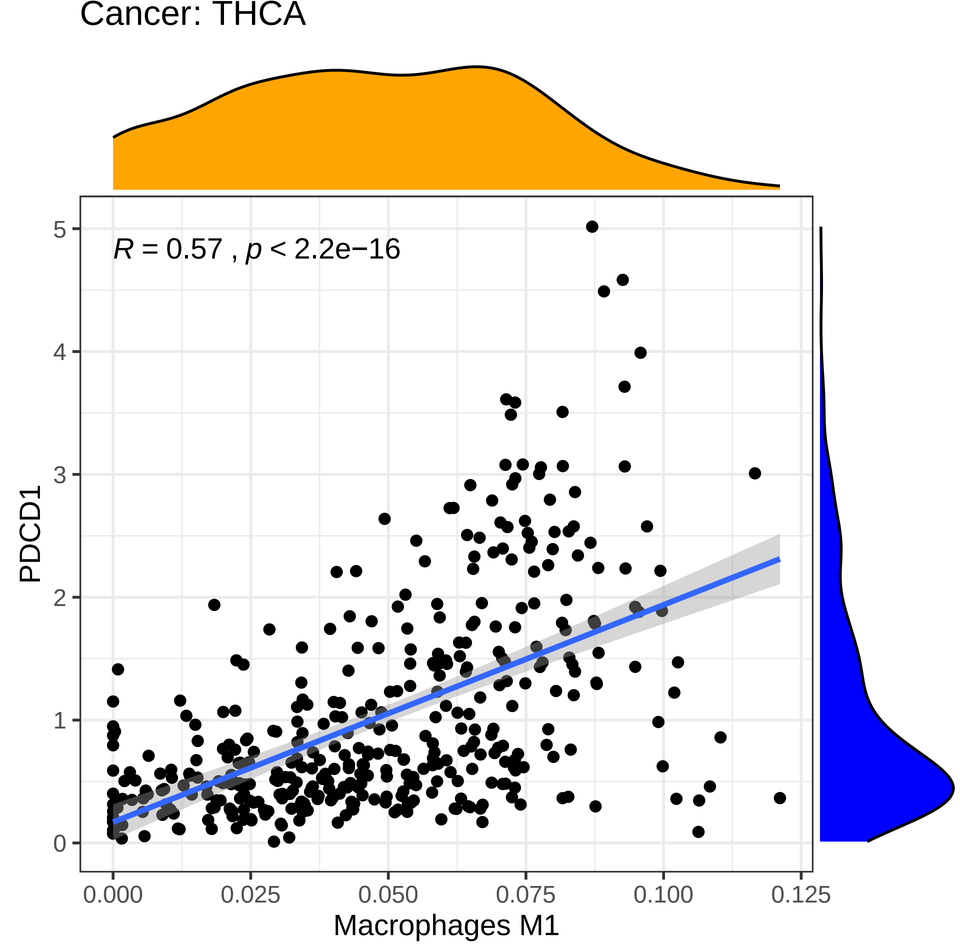

Cancer: THCA
R = 0.57 , p < 2.2e−16
PDCD1
0
1
2
3
4
5
0.000
0.025
0.050
0.075
0.100
0.125
Macrophages M1

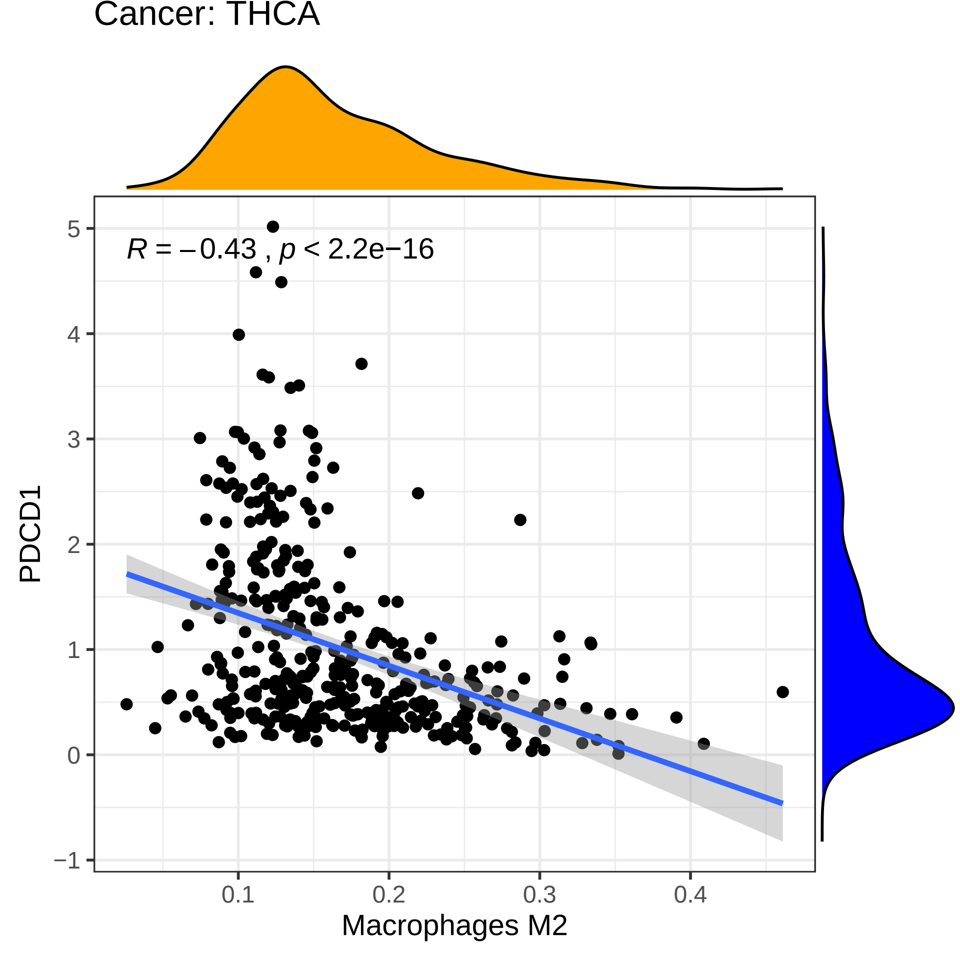

Cancer: THCA
R = −0.43 , p < 2.2e−16
PDCD1
Macrophages M2
5
4
3
2
1
0
−1
0.1
0.2
0.3
0.4

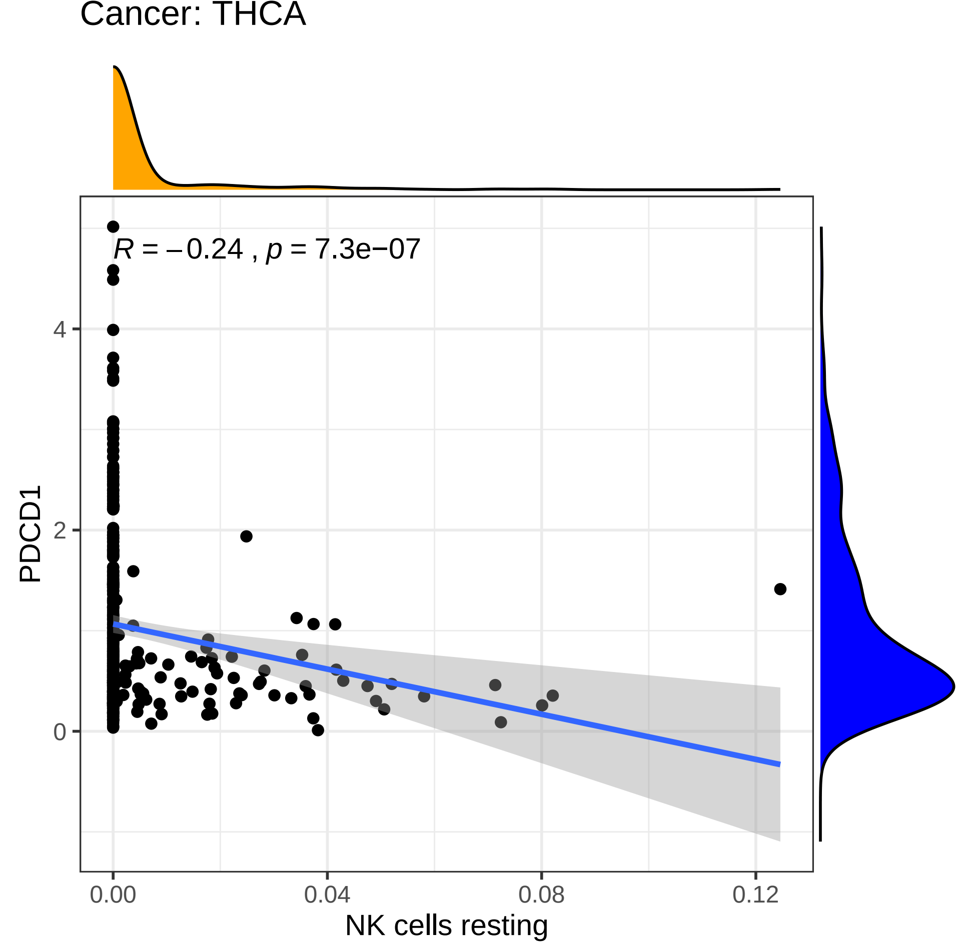
Cancer: THCA
R = −0.24 , p = 7.3e−07
PDCD1
4
2
0
0.00
0.04
0.08
0.12
NK cells resting

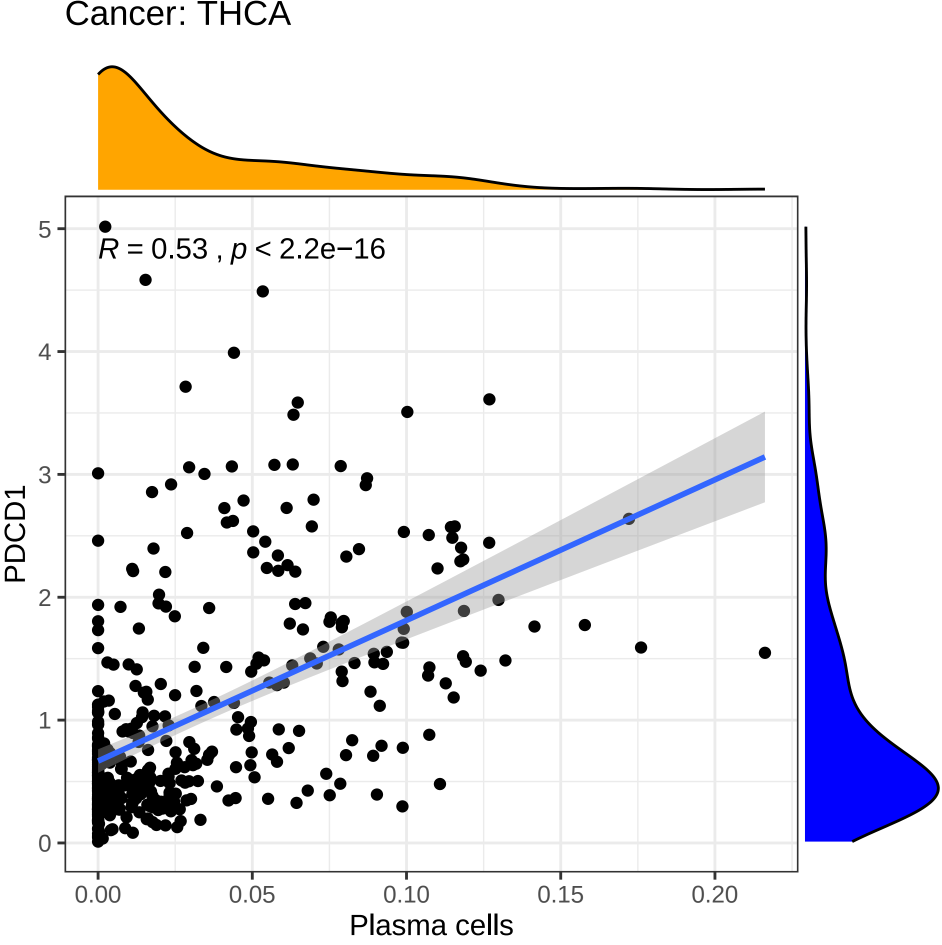

Cancer: THCA
R = 0.53 , p < 2.2e−16
PDCD1
0
1
2
3
4
5
0.00
0.05
0.10
0.15
0.20
Plasma cells

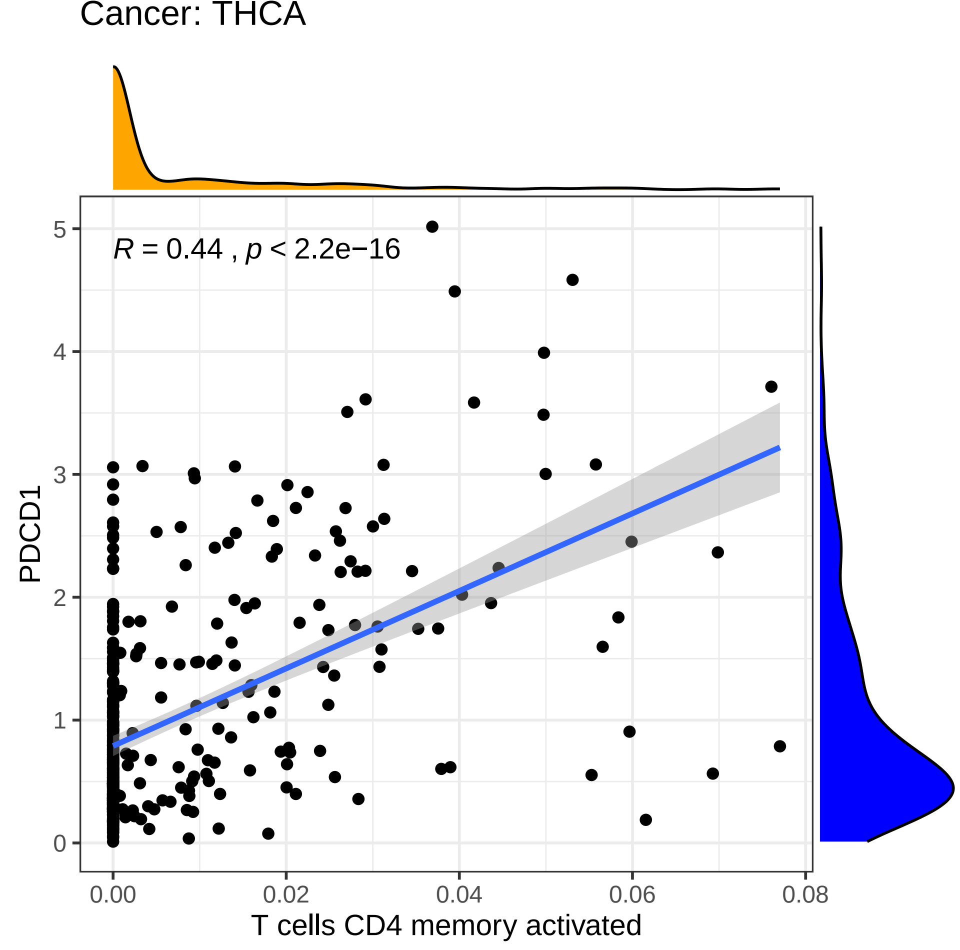

Cancer: THCA
R = 0.44 , p < 2.2e−16
PDCD1
0
1
2
3
4
5
0.00
0.02
0.04
0.06
0.08
T cells CD4 memory activated

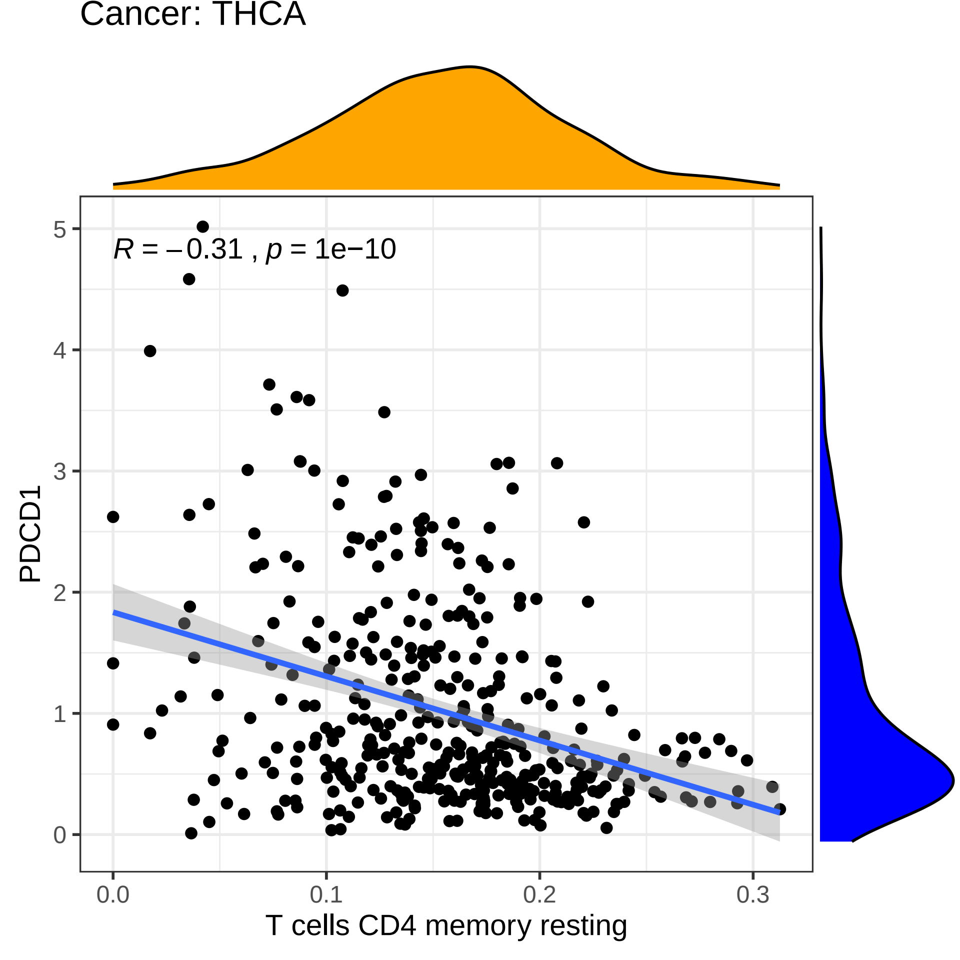
Cancer: THCA
R = −0.31 , p = 1e−10
PDCD1
T cells CD4 memory resting
0.0
0.1
0.2
0.3
0
1
2
3
4
5

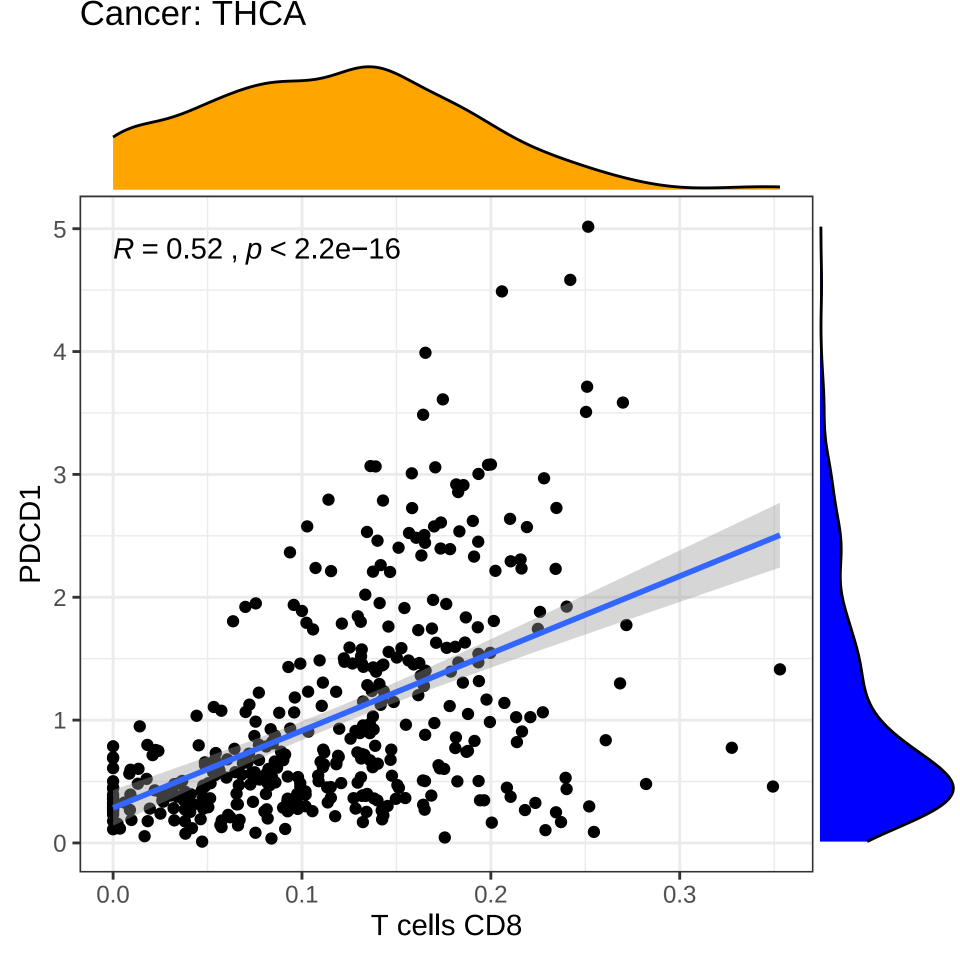

Cancer: THCA
R = 0.52 , p < 2.2e−16
PDCD1
0
1
2
3
4
5
0.0
0.1
0.2
0.3
T cells CD8

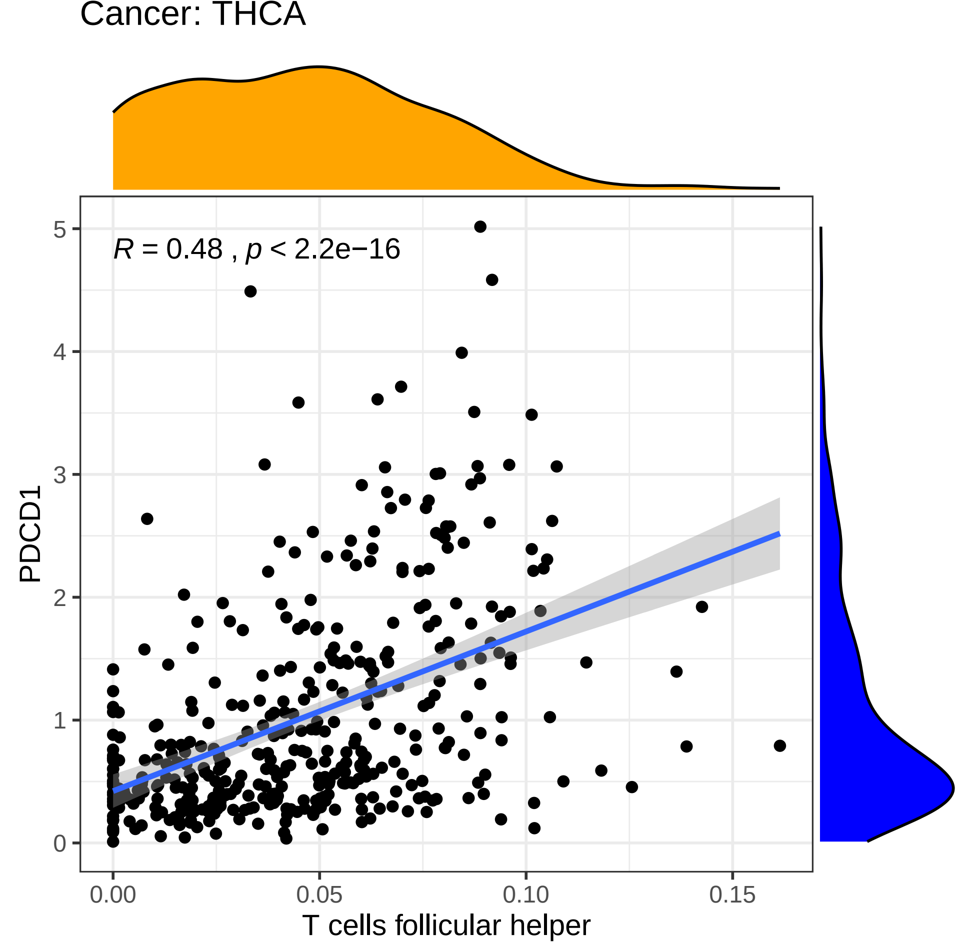

Cancer: THCA
R = 0.48 , p < 2.2e−16
PDCD1
0
1
2
3
4
5
0.00
0.05
0.10
0.15
T cells follicular helper

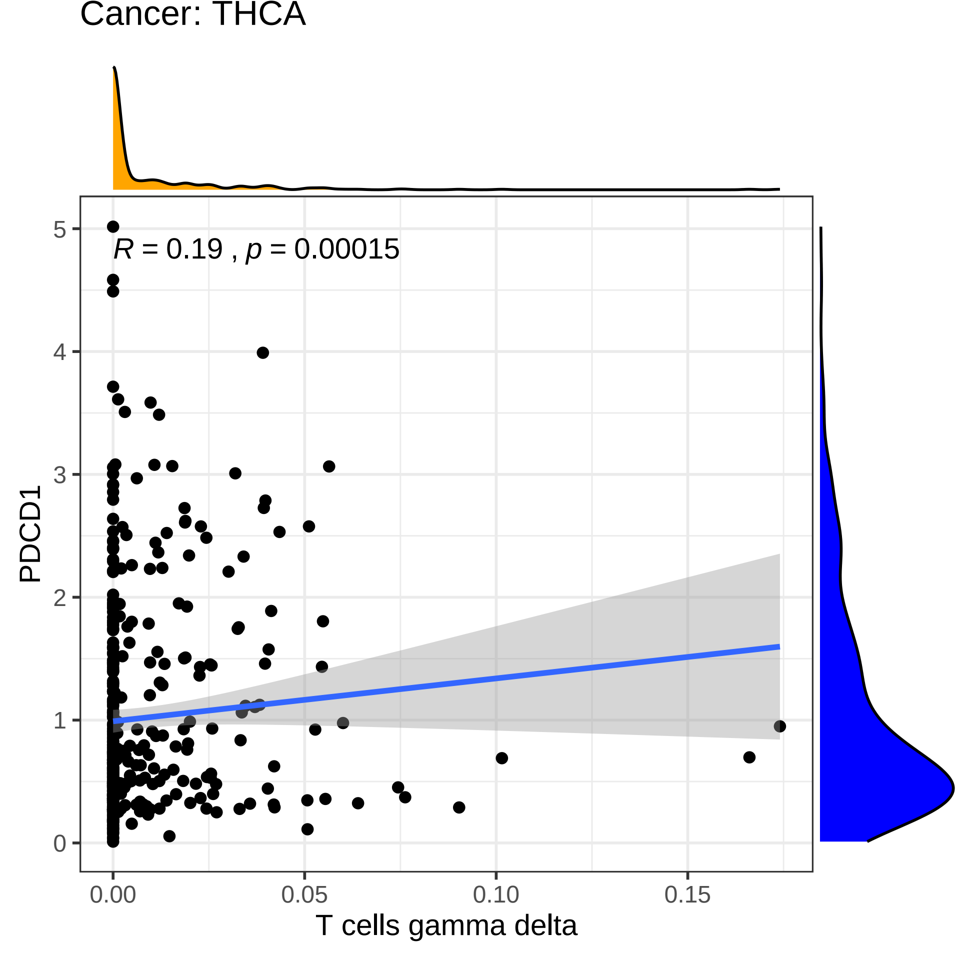

Cancer: THCA
R = 0.19 , p = 0.00015
PDCD1
T cells gamma delta
0.00
0.05
0.10
0.15
0
1
2
3
4
5

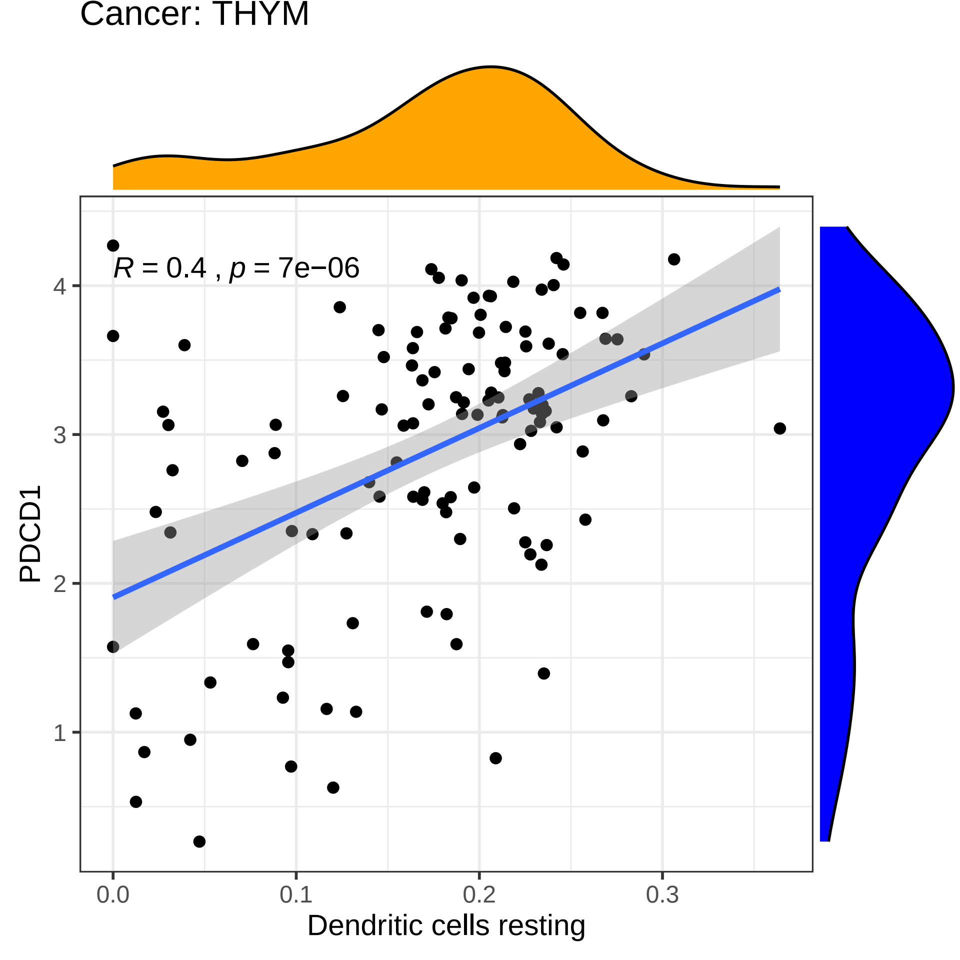
Cancer: THYM
R = 0.4 , p = 7e−06
PDCD1
Dendritic cells resting
0.0
0.1
0.2
0.3
1
2
3
4

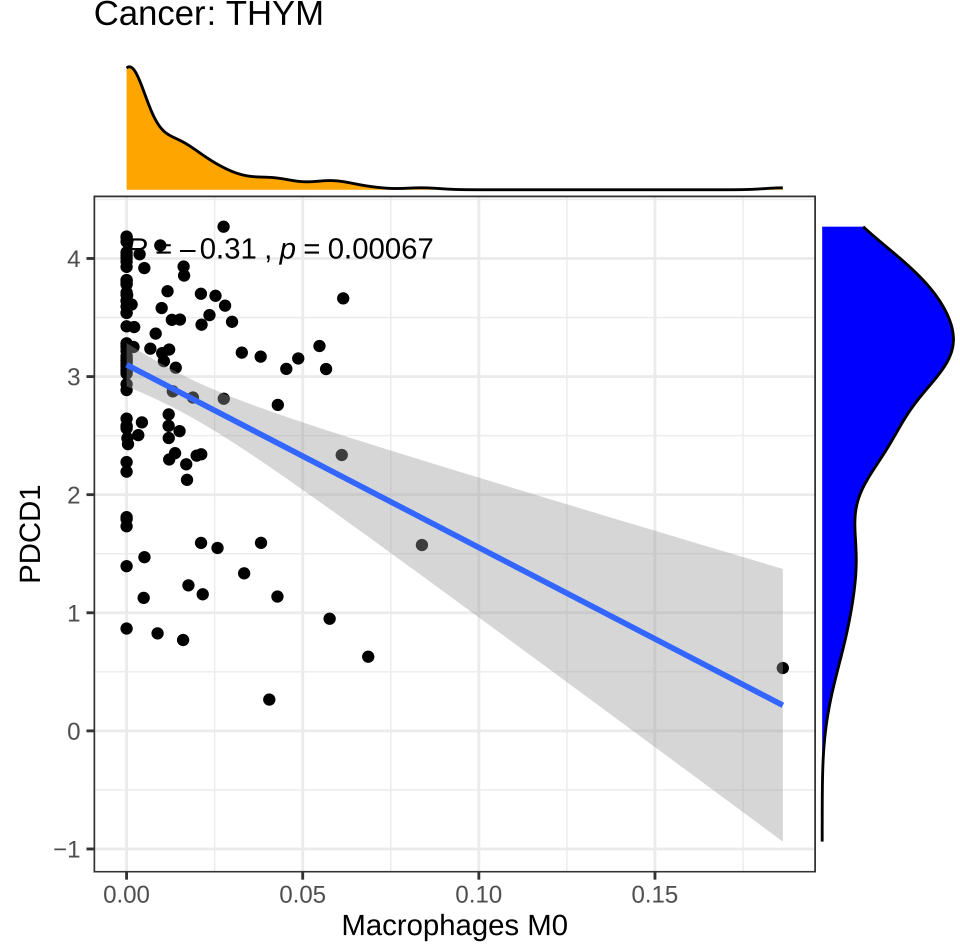

Cancer: THYM
R = −0.31 , p = 0.00067
4
3
2
1
0
−1
PDCD1
0.00
0.05
0.10
0.15
Macrophages M0

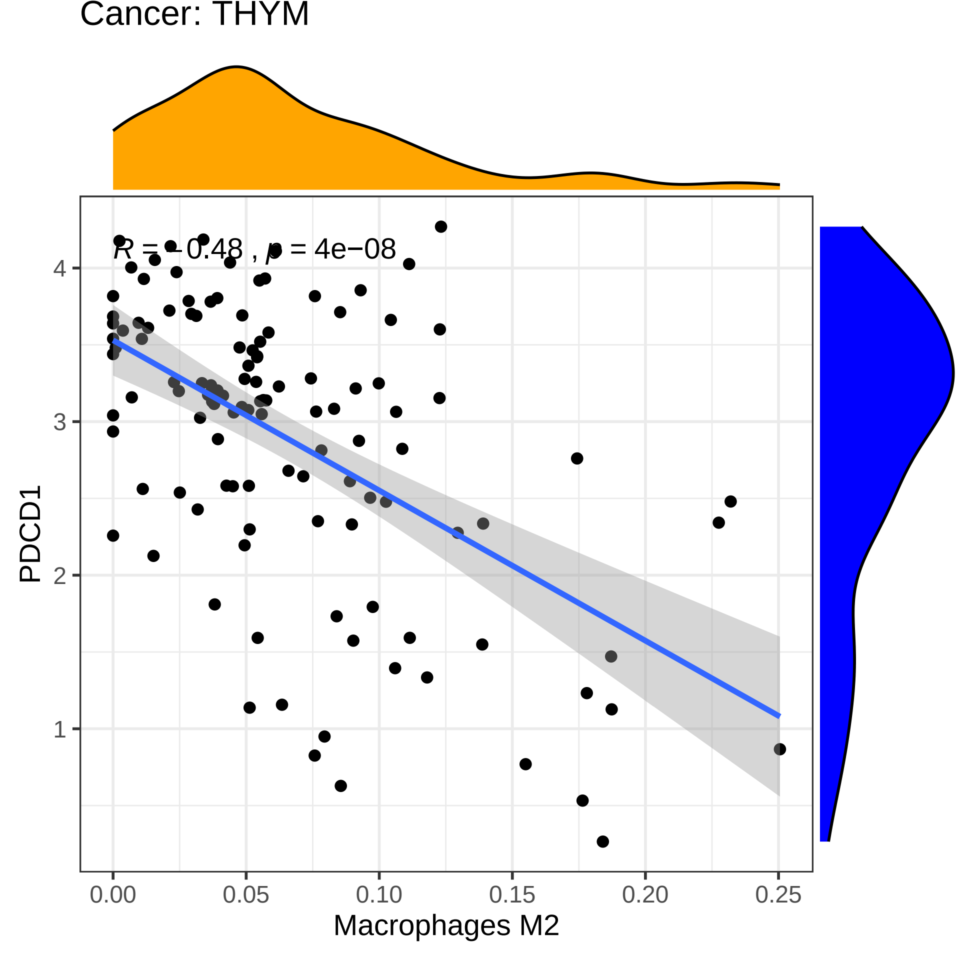
Cancer: THYM
R = −0.48 , p = 4e−08
PDCD1
4
3
2
1
0.00
0.05
0.10
0.15
0.20
0.25
Macrophages M2

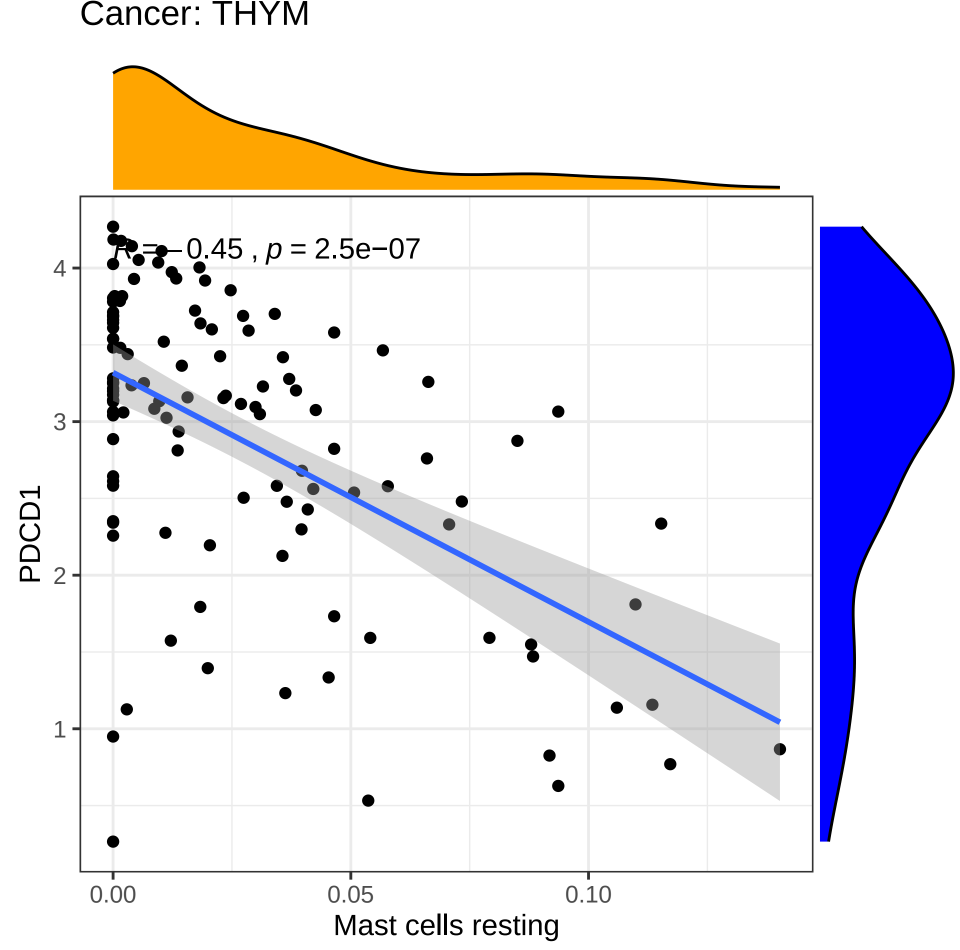

Cancer: THYM
R = −0.45 , p = 2.5e−07
PDCD1
4
3
2
1
0.00
0.05
0.10
Mast cells resting

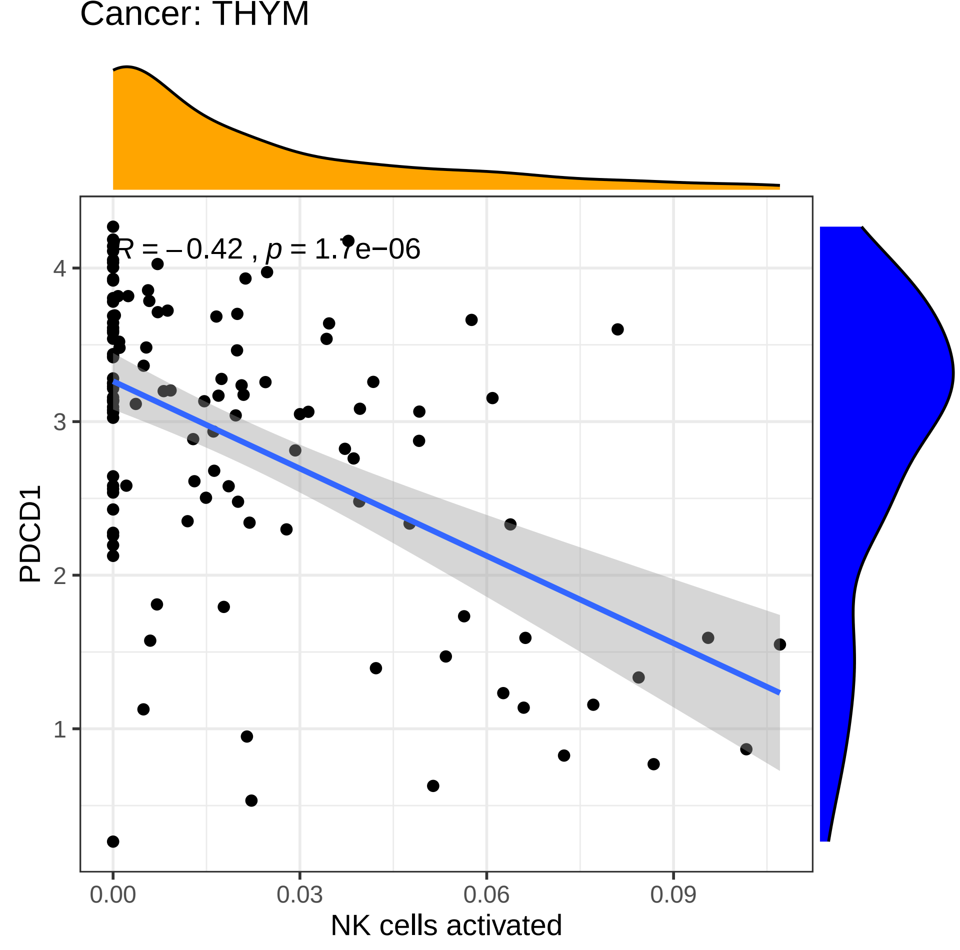
Cancer: THYM
R = −0.42 , p = 1.7e−06
PDCD1
4
3
2
1
0.00
0.03
0.06
0.09
NK cells activated

Supplementary Figure 3. The relationship between ICOS gene expression and immune cell infiltration.
